# Screening of efficient siRNA carriers in a library of surface-engineered dendrimers

**DOI:** 10.1038/srep25069

**Published:** 2016-04-28

**Authors:** Hongmei Liu, Hong Chang, Jia Lv, Cong Jiang, Zhenxi Li, Fei Wang, Hui Wang, Mingming Wang, Chongyi Liu, Xinyu Wang, Naimin Shao, Bingwei He, Wanwan Shen, Qiang Zhang, Yiyun Cheng

**Affiliations:** 1 Shanghai Key Laboratory of Regulatory Biology, School of Life Sciences, East China Normal University, Shanghai 200241, P. R. China

## Abstract

Polymers are widely used as non-viral carriers for siRNA delivery, but concern has also arisen in their limited efficacy and inherent toxicity. Whilst many of previous efforts have been documented towards improving the performance of polymers via chemical modifications, the structure-activity relationships (SAR) of these ligand-modified polymers are not well understood. To address this issue, we systemically prepared a library of surface-engineered dendrimers (>300) as the screening pool to discover efficient siRNA carriers. The modified ligands include alkyls and fluoroalkyls, amino acids, benzene derivatives and heterocyclic compounds. Gene silencing results showed that the lead material shows excellent efficacy even in hard-to-transfect cells such as mesenchymal stem cells. The SAR studies revealed that ligands containing appropriate hydrophobicity, or ligands with both hydrophobic and functional atoms/groups are essential for polymers to achive efficient knockdown efficacy. A second-generation library designed based on the above principles further confirms the proposed design criteria. The results enable the future rational design of potent siRNA carriers.

The discovery of small interfering RNA (siRNA) has offered new avenues in the clinical treatment of various diseases, such as cancers, virus infections, obesity, neurodegenerative diseases, and metabolic skeletal disorders^1^,^2^,^3^,^4^,^5^. siRNAs are relatively hydrophilic molecules with anionic charges. These chemistry features restrict the delivery of siRNAs across the negatively charged cell membrane to the site of action in the cytosol^6^. Therefore, a vector is usually required for efficient siRNA delivery^7^,^8^. In contrast with small molecule drugs with different physicochemical properties, the chemical similarity of siRNAs permits the development of a vector platform for siRNA delivery^9^. Experiences in traditional gene transfection can be adapted to the design of a large number of siRNA carriers, such as amphiphilic lipids, cationic polymers, dendrimers, peptides and nanoparticles^10^,^11^,^12^,^13^,^14^.

Cationic polymers were widely used as siRNA carriers due to their unique properties, such as facile production, biodegradability, flexibility and ease of modification^15^,^16^,^17^,^18^. However, two major obstacles, relatively low delivery efficacy and high toxicity on the transfected cells, currently hinder their utilization in clinical RNA interference^7^,^19^,^20^. To achieve efficient and low toxic siRNA delivery, the polymers need to be functionalized with various ligands including lipids^21^, sugars^22^, peptides^23^, cyclodextrins^24^, fluorous chains^25^, amino acids^26^, and nanoparticles^27^. Despite these impressive efforts, the rational design of efficient siRNA carriers remains challenging. For example, the polymers and ligand-functionalized polymers in siRNA delivery are reported by independent researchers using different polymer scaffolds and cell lines^28^. It is hard to provide structure-function relationships (SAR) of these materials based on current knowledge and experiences, which are essential for the design of ideal polymeric carriers for siRNA delivery. This situation calls for screening approaches to propose a material design principle for efficient siRNA delivery with ligand-modified polymers^10^.

Anderson and others have discovered a large number of amphiphilic lipid materials for efficient siRNA delivery using library screening or rational design strategies^10^,^29^,^30^,^31^,^32^,^33^,^34^,^35^,^36^. A multiparametric approach was successfully used to predict the efficacy of lipid nanoparticles in siRNA delivery^37^. By introduction of alkyl chains onto low molecular weight polymers, they also discovered a list of efficient amphiphilic materials with high siRNA delivery efficacy *in vitro* and *in vivo*^38^,^39^,^40^. However, these discovered siRNA carriers must be formulated with helper lipids such as cholesterol, 1,2-distearoyl-sn-glycero-3-phosphocholine (DSPC) and lipid-poly(ethylene glycol) (PEG) to achieve efficient gene silencing. The use of helper lipids make it less feasible to prepare siRNA formulations with reproducible gene silencing efficacy. In this study, we prepared a library of ligand-modified polymers, which is used as a screening pool to discover siRNA carriers which can achieve efficient gene silencing in the absence of helper lipids. Besides alkyl chains, the ligands in this study include fluoroalkyl chains, benzene derivatives, heterocyclic compounds, amino acids and etc. Dendrimer, a class of synthetic polymers with a well-defined molecular structure^41^,^42^,^43^ was selected as the model polymer due to its globular shape, good solubility and monodispersity^44^,^45^. The use of dendrimer as the polymer scaffold can exclude the effects of polymer structure, conformation and polydispersity on the siRNA delivery efficacy^40^. In addition, cationic dendrimer with primary amine as the surface functionality can be functionalized with various ligands in a multivalent fashion via facile and general chemical reactions^28^. These unique properties make dendrimer an ideal candidate in the study of SAR of ligand-modified polymers.

## Results and Discussion

### Synthesis of surface-engineered dendrimers

**Figure 1:**
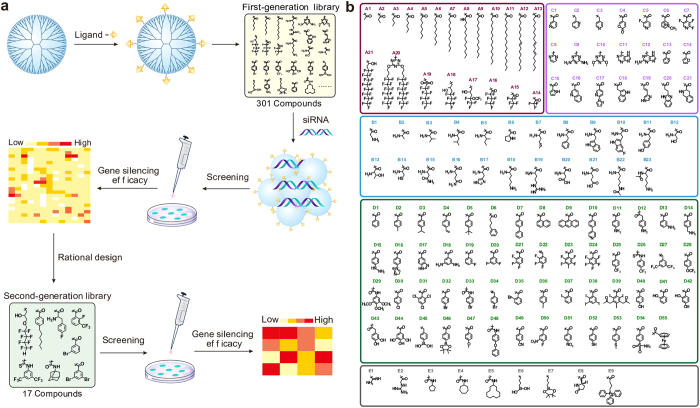
Screening of efficient siRNA carriers in a library of surface-engineered dendrimers. (**a**) Scheme of library construction, material screening and rational design of second-generation library. (**b**) Structures of the ligands conjugated on dendrimer surface. A1-A21: aliphatic and fluorous chains; B1-B23: amino acids; C1-C21: heterocyclic compounds, D1-D55: benzene derivatives and E1-E9: other compounds.

Generation 5 (G5) polyamidoamine (PAMAM) dendrimers with a theoretical number of 128 surface primary amine groups were conjugated with 129 types of ligands listed in Fig. 1. The conjugated ligands include several sub-groups, like alkyl and fluoroalkyl chains, amino acids, heterocyclic compounds, benzene derivatives and other compounds. A total number of 301 surface-engineered dendrimers were obtained by simply reacting the primary amine groups of dendrimer with carboxyl, anhydride, isocyanate, isothiocyanate, halogen or epoxy groups (see Supplementary Table S1).

### Gene silencing efficacies of the surface-engineered dendrimers in the library

**Figure 2:**
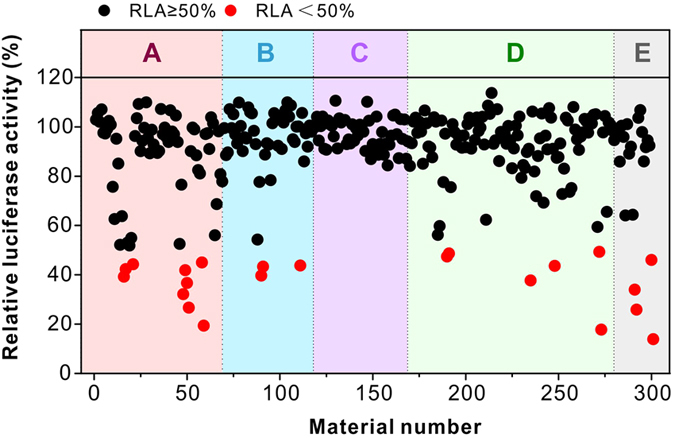
Gene silencing efficacies of the surface-engineered dendrimers in the prepared library. Relative luciferase activities (normalized to controls) of the 301 materials are screened on HeLa-luc cells. 50 nM siLuc is used for each transfection and the material to siLuc weight ratio ranges from 1 to 20. The gene silencing experiments continue for 24 h. About 7.31% of the materials in the library induced >50% gene knockdown efficacy (shown in red dots).

**Figure 3:**
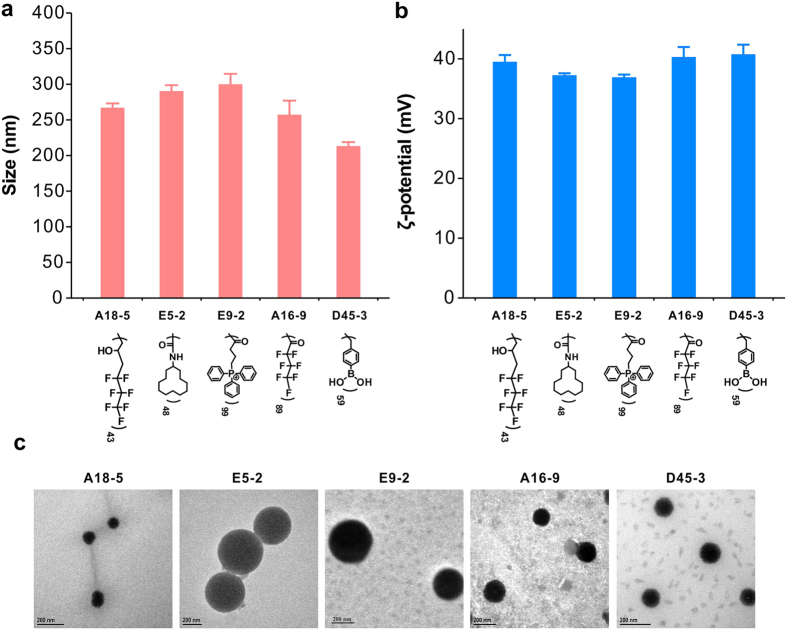
Hydrodynamic size (**a**), zeta potential (**b**) and TEM (**c**) images of the dendrimer/siRNA complexes. The screened top five performing dendrimers were mixed with siLuc at optimal weight ratios. Error bars in a and b represent the s.e. (n = 3).

**Figure 4:**
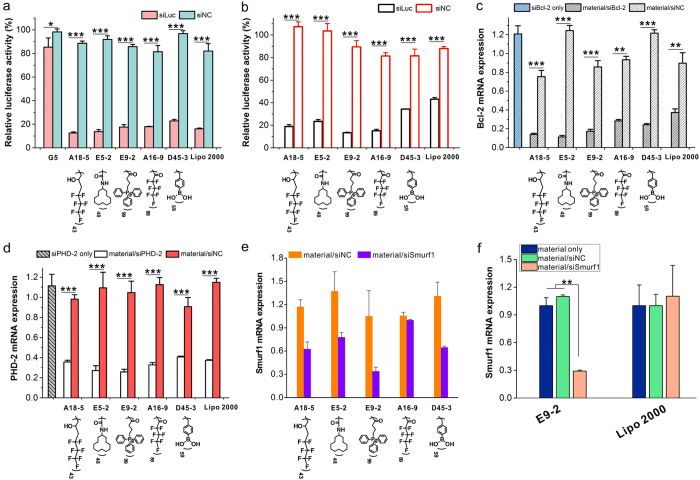
*In vitro* siRNA delivery efficacy of the screened materials. (**a**) Luciferase gene silencing efficacies of the five most efficient materials in HeLa-luc cells. 50 nM siLuc is used for each transfection. (**b**) Luciferase gene silencing in MDA-MB231-luc cells. 10 nM siLuc is used for each transfection. (**c**) Bcl-2 gene silencing in HeLa cells. 50 nM siBcl-2 is used for each transfection. (**d**) PHD-2 gene silencing in NIH3T3 cells. 50 nM siPHD-2 is used for each transfection. (**e**) Smurf1 gene silencing in hard-to-transfect cells. Primary mouse mesenchymal stem cells were transfected with 50 nM siSmurf1. The gene silencing experiments continue for 24 h. (**f**) Efficacies of E9-2 in siSmurf1 delivery into primary mouse mesenchymal stem cells. Lipo 2000 is used as a control. Error bars in a-f represent the s.e. (n = 3). *P < 0.05, **P < 0.01, ***P < 0.005 analyzed by student’s t-test.

Gene silencing efficacy of the resulting materials was first tested on HeLa-luc cells (HeLa cells stably expressing a firefly luciferase gene) using a siRNA specifically targeting luciferase (siLuc). In the screening experiments, the siLuc concentration is fixed at 50 nM and the polymer concentration varies in the polymer/siRNA weight ratios (w/w) of 1 to 20. As shown in Fig. 2, a total number of 22 materials were screened as efficient materials (shown as red dots). About 7.31% of the materials in the library induced more than 50% gene knockdown efficacy on the HeLa-luc cells. We further compared the five most efficient polymers in the library with a commercial transfection reagent Lipofectamine 2000 (Lipo 2000). The screened top 5 performing materials form small-sized polyplexes with favorable biophysical properties, e.g. they are able to form positively charged nanoparticles below 300 nm with siRNA (Fig. 3). Parallel experiments were carried out using scrambled siRNA (siNC) that is inactive in silencing luciferase as negative controls for each material. As shown in Fig. 4a, without surface modification, G5 PAMAM dendrimer only shows extremely low gene silencing efficacy (<10%), while the screened top 5 performing materials show comparable efficacies to Lipo 2000 on the HeLa-luc cells. Additionally, the polymers with siNC show negative gene silencing in the cells, suggesting high specificity in gene silencing mediated by the screened materials. When decreasing the siLuc dose, the gene knockdown efficacies of the screened materials are slightly reduced (see Supplementary Fig. S1). Even at a siRNA dose of 5 nM, the screened polymers still show gene silencing efficacies above 70%. Moreover, the screened materials exhibit high gene silencing efficacies in MDA-MB231-luc cells (MB231 cells stably expressing a firefly luciferase gene) (Fig. 4b). Besides the luciferase gene, the materials can also efficiently and specifically knockdown other genes such as Bcl-2 in HeLa cells (Fig. 4c) and PHD-2 in NIH3T3 cells (Fig. 4d and Supplementary Fig. S2). We also test gene knockdown efficacy of screened materials in hard-to-transfect cells such as primary mouse mesenchymal stem cells (Fig. 4e). The lead material (E9-2, triphenylphosphonium-modified G5 dendrimer) shows high gene silencing efficacy even in hard-to-transfect cells. For example, E9-2 efficiently knockdown the Smurf1 gene in primary mouse mesenchymal stem cells (Fig. 4f), followed by the down-regulation of Smurf1 protein (see Supplementary Fig. S3), but Lipo 2000 shows poor efficacy (<10%) in the delivery of siRNA into primary mouse mesenchymal stem cells. Anderson *et al*. recently also reported that alkyl substitution of regularly branched dendrimers such as PAMAM and polypropylenimine is a facile strategy to prepare efficient siRNA carriers^39^,^40^. However, these amphiphiles should be co-formulated with helper lipids such as DSPC, DSPC-PEG, and cholesterol, which make it difficult to prepare formulations with reproducible siRNA delivery efficacy. In comparison, the discovered materials in our library (A18-5, E5-2, E9-2, A16-8 and D45-3) can efficiently knockdown the target genes in various cell lines without any helper lipids.

### SAR of the surface-engineered dendrimers in siRNA delivery

**Figure 5:**
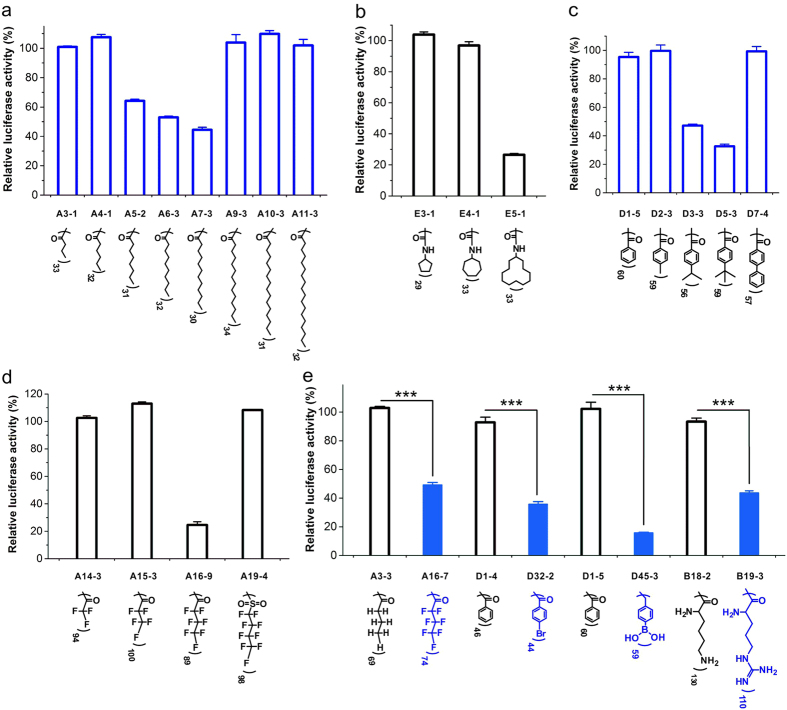
SAR of the surface-engineered dendrimers in siRNA delivery. (**a**) Alkyl chain-modified dendrimers. (**b**) Cycloalkane-modified dendrimers. (**c**) Aromatic ligand-modified dendrimers. (**d**) Fluoroalkyl chain-modified dendrimers. (**e**) Beneficial effect of functional groups in siRNA delivery. The gene silencing efficacies for the materials are conducted at optimal transfection conditions with 50 nM siLuc in HeLa-luc cells. Error bars in a-e represent the s.e. (n = 3). ***P < 0.005 analyzed by student’s t-test.

**Figure 6:**
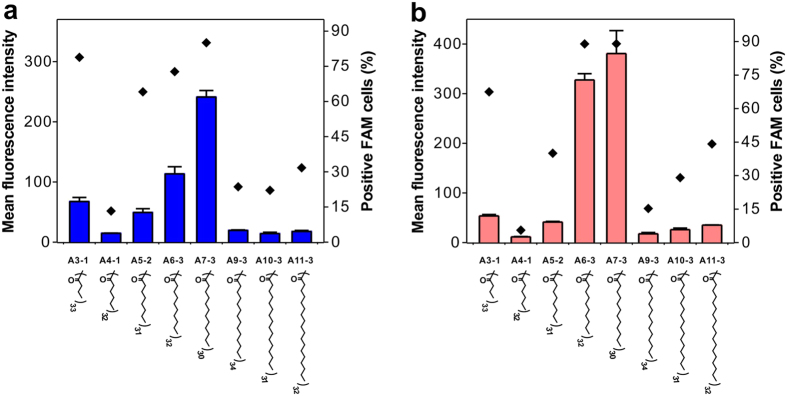
Cellular uptake efficacy of alkyl chain-modified dendrimer/siRNA complexes in HeLa-luc cells. (**a**) At optimal weight ratios as shown in Fig. 5a. The optimal weight ratios for A3-1, A4-1, A5-2, A6-3, A7-3, A9-3, A10-3 and A11-3 are 12, 20, 10, 2, 3, 4, 4 and 4, respectively. (**b**) At an equal polymer molar concentration of 175 nM (175 nM is the optimal concentration for lauric acid-modified G5 PAMAM dendrimer). 0.5 μg siLuc is used for each well and the cellular uptake experiments were conducted for 2 h. Error bars in a-b represent the s.e. (n = 3).

We further investigated SAR of the surface-engineered dendrimers in gene silencing. It is known that hydrophobic modification on polymers could improve serum stability, membrane fusion, cellular uptake and intracellular siRNA disassociation abilities of polymer/siRNA complexes^38^,^39^,^40^,^46^. According to the chemical structures of efficient materials in the library, the modification of hydrophobic ligands on dendrimer is beneficial for efficient siRNA delivery. As shown in Fig. 5a, conjugation of alkyl with proper chain length to G5 dendrimer significantly improves its gene silencing efficacy. In particular, G5 dendrimer modified with lauric acid (A7-3) shows the highest efficacy among the alkyl chain-functionalized dendrimers. The improved gene silencing efficacy can be explained by increased cellular uptake efficacy (Fig. 6). These amphiphilic materials can escape from endolysosomes through a combination of membrane fusion mechanism of the lipids and proton sponge effect of the dendrimers^39^,^47^,^48^. Conjugation of aliphatic acids with longer chains such as myristic acid (A9-3), palmitic acid (A10-3) and fatty acid (A11-3) fails to knockdown the luciferase gene probably due to the decreased charge density on the dendrimer surface (shielding effect of longer alkyl chains, Supplementary Fig. S4)^49^. Similarly, conjugation of cycloaliphatic compounds with higher hydrophobicity shows a beneficial effect in improving the efficacy of G5 dendrimer (see Supplementary Fig. 5b). G5 dendrimer modified with 33 cyclododecyl chains shows the highest efficacy among the polymers in Fig. 5b. The compound that contains an aromatic ring as well as an alkyl group with appropriate hydrophobicity also shows much enhanced gene knockdown efficacy when conjugated onto dendrimer surface (Fig. 5c). Besides, dendrimers modified with fluorous chains show a similar phenomenon in gene silencing (Fig. 5d). G5 dendrimer conjugated with fluoroalkyl chains containing seven fluorine atoms shows the highest efficacy. Considering the more hydrophobic effect of fluoroalkyls compared with alkyls, much shorter fluoroalkyl chains are needed to achieve efficient gene silencing (C4 for fluoroalkyls versus C12 for alkyls). The improved gene knockdown efficacy after fluorination can be explained by enhanced cellular uptake and endosomal escape of the complexes due to the fluorine effect as reported in a recent study^50^. All the highest efficient materials in Fig. 5b–d exhibit the highest cellular uptake among the control materials no matter at optimal transfection conditions or equal molar concentrations (see Supplementary Figs S5–S7).

**Figure 7:**
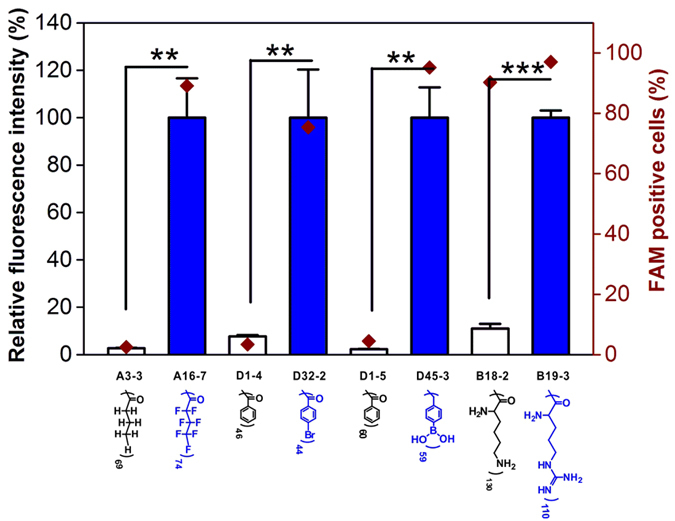
Cellular uptake efficacy of dendrimer/siRNA complexes in Fig. 5e in HeLa-luc cells. 0.5 μg siLuc is used for each well and the cellular uptake experiments were conducted for 2 h. The fluorescence intensity of the cells was normalized to that of cells transfected by materials with functional groups (shown in blue color). The cellular uptake of dendrimer/siRNA complexes for A16-7, D32-2, D45-3 and B19-3 were conducted at optimal weight ratios. The optimal weight ratios for A16-7, D32-2, D45-3 and B19-3 are 4, 20, 20 and 16, respectively. The molar concentrations of the control materials such as A3-3, D1-4, D1-5 and B18-2 equal to those of A16-7, D32-2, D45-3 and B19-3, respectively. Error bars in this figure represent the s.e. (n = 3). **P < 0.01, ***P < 0.005 according to relative fluorescence intensity (%) analyzed by student’s t-test.

Besides the hydrophobic effect, we also found some functional atoms or ligands in improving the siRNA delivery efficacy. In Fig. 5e, the dendrimers conjugated with different numbers of benzoic acid show extremely low gene silencing efficacy (<10%), while significantly improved gene silencing efficacy was observed if the same structures were incorporated with bromine atoms or boronic acid groups. Similarly, butyric acid- and lysine-modified G5 dendrimers show poor gene knockdown efficacies, but performances of the materials can be improved by replacing the hydrogen atoms on butyric acid with fluorine atoms or replacing the amine groups on the side chain of lysine with guanidine groups. These results suggest the beneficial effects of fluorine/bromine atoms, boronic acid and guanidine groups in siRNA delivery. The presence of these atoms or ligands significantly improves the cellular uptake efficacy of the complexes (Fig. 7). The hydrophobic effect of the ligand on dendrimer surface is also essential for the efficient siRNA delivery. For example, G5 dendrimer modified with butylboronic acid with decreased hydrophobicity compared to phenylboronic acid show limited gene silencing efficacy (see Supplementary Fig. S8). A combination of hydrophobic ligands (such as aromatic rings or alkyl chains) and functional ligands (such as boronic acid or guanidine) generates efficient materials for siRNA delivery.

### A second-generation library to confirm the material design principle

**Figure 8:**
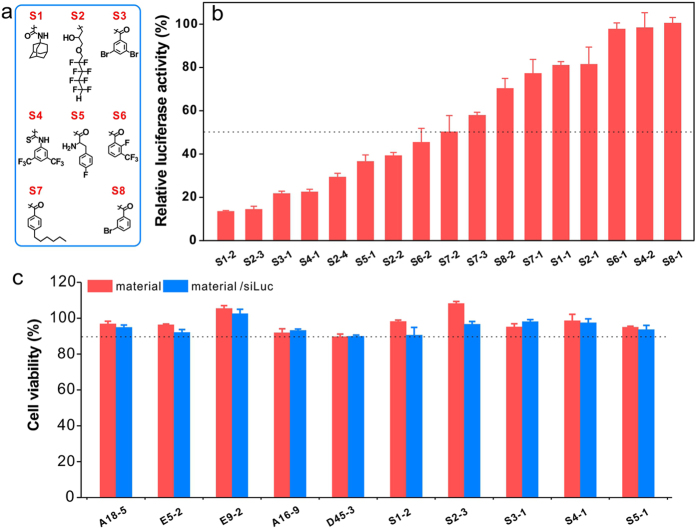
A second-generation library to confirm the SAR of surface-engineered dendrimers. (**a**) 17 second-generation surface-engineered dendrimers were synthesized according to the proposed material design criteria: with appropriate hydrophobicity or combination of hydrophobic ligands and functional ligands. (**b**) *In vitro* siRNA delivery efficacies of the materials in the second-generation library. The gene silencing experiments were conducted in HeLa-luc cells with 50 nM siLuc for each transfection. Error bars in b represent the s.e. (n = 3). (**c**) Viability of cells treated with the efficient materials from the first- and second-generation libraries with or without 50 nM siRNA by an MTT assay at their optimal transfection conditions. Error bars in c represent the s.e. (n = 5).

To further confirm the material design criteria described above, we synthesized a small second-generation library of surface-engineered dendrimers (Fig. 8a and Supplementary Table S2). The ligand in the library either has appropriate hydrophobicity (fluorous chains or aliphatic rings) or combines both hydrophobic ligands (phenyl group) and functional ligands (fluorine and bromine atoms/ligands). In such a way, we could expect a large fraction of this second-generation library to be efficacious in comparison with the first-generation one. When tested the luciferase gene silencing efficacy of the materials on HeLa-luc cells at a siRNA dose of 50 nM, it was found that more than half of the designed materials (52.9%) in the second-generation library show gene silencing efficacy above 50% (Fig. 8b). These results suggest that the proposed material design criteria are able to significantly increase the probability of success in preparing efficient surface-engineered dendrimers for siRNA delivery. For cationic polymers, there is usually a transfection efficacy-cytotoxicity correlation associated with the carriers. We further evaluated the cytotoxicity of the efficient materials from the first- and second-generation libraries with or without siRNA by an MTT assay. All the materials show minimal toxicity on the transfected cells (>90% viability) at their optimal transfection conditions (Fig. 8c), suggesting that the materials feature high efficacy and low cytotoxicity when delivering various siRNA into different cell lines. Just like most of the pioneer work by Anderson *et al*.^31^,^32^,^34^,^35^,^51^, we need to further investigate the *in vivo* gene silencing efficacy of the discovered materials for better understanding the structure-function relationships of surface-engineered dendrimers in siRNA delivery.

## Conclusions

In summary, we have synthesized a library of surface-engineered dendrimers via facile and simple chemical reactions and screened their performances in siRNA delivery. The screened efficient materials can specifically knockdown several target genes in different cell lines. Efficacy of the lead material E9-2 is much superior to commercial lipid carriers such as Lipo 2000 in hard-to-transfect stem cells. SAR studies demonstrated that hydrophobic ligands such as aliphatic chains and aromatic rings, as well as functional ligands such as fluorine and bromine atoms, boronic acid and guanidine groups are essential for the high gene silencing efficacy of screened materials. Above all, the screened materials in the library enable efficient delivery of various siRNA into different cell lines with minimal cytotoxicity. The SAR results in this study successfully paved the way towards the design of a second-generation library of surface-engineered dendrimers with high gene silencing efficacy.

## Materials and Methods

### Materials

Ethylenediamine-cored G5 PAMAM dendrimer with primary amine groups was purchased from Dendritech, Inc. (Midland, MI). The received dendrimer was characterized by ^13^C NMR and polyacrylamide gel electrophoresis to confirm its quality. Lipo 2000 was purchased from Invitrogen (Carlsbad, CA).

### Chemicals

Propionic anhydride, hexanoic anhydride, decanoic anhydride, dodecanoic anhydride, myristic acid, palmitic acid, stearic acid, oleic acid, linoleic acid, trifluoroacetic anhydride, pentafluoropropionic anhydride, heptafluorobutyric anhydride, [2,3,3,3-Tetrafluoro-2-(trifluoromethoxy)propyl]oxirane, 3-(perfluoropropyl)-1,2-propenoxide, glycidyl 2,2,3,3,4,4,5,5-octafluoropentyl ether, nonafluorobutanesulfonic anhydride, 2-chloro-4,6-bis[3-(perfluorohexyl)propyloxy]-1,3,5-triazine, (2,2,3,3,4,4,5,5,6,6,7,7,8,8,9,9,9-heptadecafluorononyl)oxirane, cyclopentyl isocyanate, cycloheptyl isocyanate , cyclododecyl isocyanate, 3-bromopropylboronic acid pinacol ester, (3-carboxypropyl)triphenylphosphonium bromide, nicotinic acid, coumalic acid, 2-thiophenecarboxylic acid, 2-furoic acid, pyrrole-2-carboxylic acid, 3-quinolinecarboxylic acid, 3-indoleacetic acid, 4-imidazoleacrylic acid, 2-benzimidazolepropionic acid, guanidineacetic acid, 3-fluoro-4-pyridinecarboxylic acid, 2,3,5-trifluoropyridine-4-carboxylic acid, benzoic acid, p-toluic acid, 4-phenylbutyric acid, 4-hydrazinobenzoic acid, 2,3,4-trifluorobenzaldehyde, 2,3,5,6-tetrafluoro-p-toluic acid, 2,3,4,5,6-pentafluorobenzoic acid, 3,5-bis(trifluoromethyl)benzaldehyde, 4-(trifluoromethyl)phenyl isothiocyanate, 3,4,5-trimethoxyphenyl isocyanate, 4-bromophenyl isocyanate, 4-bromobenzaldehyde, 4-iodobenzoic acid, salicylic acid, 2,4-dihydroxybenzoic acid, 3,4-dihydroxyphenylacetic acid, 4-methoxybenzoic acid, 4-phenoxyphenyl isocyanate, 4-cyanobenzoic acid, 2-iodobenzoic acid, 2,3,5-triiodobenzoic, adamantyl isocyanate, 4-mercaptobenzoic acid, 4-(bromomethyl)phenylboronic acid, 4-(methylthio)phenyl isocyanate, 4-chlorobenzoic acid, 4-bromobenzoic acid, dicyclohex-ylcarbodiimide (DCC) and N-hydroxysuccinimide (NHS) were purchased from Sigma-Aldrich (St. Louis, MO). 4-Isopropylbenzoic acid, 4-vinylbenzoic acid, 4-tert-Butylbenzoic acid, 4-hexylbenzoic acid, biphenyl-4-carboxylic acid, 1-naphthoic acid, 9-anthroic acid, 4-(4-pyridyl)benzoic acid, 3-fluorobenzoic acid, 3,5-difluorobenzoic acid, 2,3,4-trifluorobenzoic acid, 4-(trifluoromethyl)benzoic acid, 4-(trifluoromethoxy)benzoic acid, 2,4,6-trichlorobenzoic acid, 5-benzimidazolecarboxylic acid, diatrizoic acid, gallic acid monohydrate, 4-carboxybenzeneboronic acid pinacol ester, 4-nitrobenzoic acid, 4-fluoro-3-nitrobenzoic acid, 2-bromobenzoic aicd, 4-sulfamylbenzoic acid butyric anhydride, 6-(1H-pyrazol-1-yl) nicotinic acid, 2-(chloromethyl)pyridine hydrochloride, 4-(chloromethyl)pyridine hydrochloride, trigonelline hydrochloride, 2-bromopyrimidine, 6-chloropurine, 2-amino-6-chloropurine, 2-chloro-4,6-diamino-1,3,5-triazine, 2-chloro-4,6-dimethoxy-1,3,5-triazine and Fmoc-L-4-fluorophe were purchased from Aladdin (Shanghai, China). Octanoic anhydride, dodecyl isocyanate, 3-(perfluoropropyl)-1,2-propenoxide, 5-fluorotryptophan, Boc-4-Abz-OH, Boc-4-aminophenylacetic acid, Boc-3-Abz-OH, 4-(Boc-aminomethyl)benzoic acid, 4-(1H-imidazol-1-yl)benzoic acid, Di-Fmoc-3.5-diaminobenzic acid, ferrocenecarboxylic acid and Fmoc-L-1,2,3,4-tetrahydroisoquinoline-3-carboxylic acid were obtained from J&K Scientific (Shanghai, China). Triethylamine (TEA), trifluoroacetic acid (TFA) and dimethyl sulfoxide (DMSO), N,N-dimethylformamide (DMF), N, N-diisopropylethylamine (DIPEA), methanol and ethanol were purchased from Sinopharm Chemical Reagent Co., Ltd (Shanghai, China). Boc-Gly-OH, Boc-Ala-OH, Boc-Val-OH, Boc-Leu-OH, Boc-Ile-OH, Boc-Pro-OH, Boc-Met-OH, Boc-Phe-OH, Boc-Trp(Boc)-OH, Boc-Tyr(tBu)-OH, Boc-Ser(tBu)-OH, Boc-Thr(tBu)-OH, Boc-Cys(Acm)-OH, Boc-Asn(Trt)-OH, Boc-Gln(Trt)-OH, Boc-His(Trt)-OH, Boc-Lys-OH, Boc-Arg(Pbf)-OH, Boc-Asp(otBu)-OH, Boc-Glu(otBu)-OH, Boc-Lys(Ac)-OH and Ac-Lys(Boc)-OH were purchased from GL Biochem (Shanghai, China). 4-Bromobutylboronic acid was purchased from Santa Cruz Biotechnology (Dallas TX). The chemicals were used as received without further purification.

### siRNAs

All siRNAs including fluorescently labeled siLuc (with a FAM conjugated at the 5′ end of siLuc, siLuc-FAM) and scrambled siRNA non-specific to any human gene (siNC) were synthesized by GenePharma Co. Ltd. (Shanghai, China). The sequences for the sense and antisense strands of siRNAs are as follows:

The siLuc (targeting firefly luciferase)

Sense: 5′-CCCUAUUCUCCUUCUUCGCdTdT-3′;

Antisense: 5′-GCGAAGAAGGAGAAUAGGGdTdT-3′;

siBcl-2 (targeting Bcl-2)

Sence: 5′-CCGGGAGAUAGUGAUGAAGdTdT-3′;

Antisense: 5′-CUUCAUCACUAUCUCCCGGdTdT-3′;

siPHD-2 (targeting prolyl hydroxylase)

Sense: 5′-UCACGUUGAUAACCCAAAUdTdT-3′;

Antisense: 5′- AUUUGGGUUAUCAACGUGAdTdT-3′;

siSmurf1 (targeting Smurf1)

Sence: 5′-CCUUGCAAAGAAAGAGdTdT-3′;

Antisense: 5′-CUCUUUCUUUGCAAGGdTdT-3′.

### Synthesis and characterization of surface-engineered dendrimers

G5 PAMAM dendrimer was reacted with the compounds via facile reactions (interacting of amine groups of G5 PAMAM dendrimers with carboxyl, anhydride, isocyanate, isothiocyanate, halogen or epoxy groups at different feeding ratios). The detailed feeding ratios and reaction conditions for the synthesized materials are listed in Supplementary Table S1. The synthesized materials were purified by intensive dialysis against distilled water or dimethyl sulfoxide according to their solubility, and characterized by ^1^H NMR, ninhydrin assay or fluorine element analysis to calculate the number of conjugated ligands. Detailed synthesis and characterizations on the surface-engineered dendrimers can be found in Supplementary Table S1 and Table S2. The ^1^H NMR spectra of synthesized materials are listed in Supplementary Appendix A.

### Characterizations of the complexes

The synthesized materials were mixed with siRNA in diethylpyrocarbonate-treated water at different weight ratios (w/w) for 30 min. The size and zeta potential of the complexes were measured by Zetasizer Nano ZS (Malvern Instrument) and transmission electron microscope (HT7700, HITACHI, Japan). The binding of siRNA with the alkyl chain-modified dendrimers was determined by agarose gel electrophoresis assay. The weight ratio of dendrimer to siRNA ranges from 2 to 6 (10 μL of the sample containing 1–3 μg polymer and 0.5 μg siRNA). The polyplexes were diluted with DNA loading buffer and the samples were run on a 1% (w/v) agarose gel at 90 V for 20 min.

### Cell culture and gene silencing experiments

HeLa, HeLa-luc (HeLa cells stably expressing a firefly luciferase gene) and NIH3T3 cells were cultured in 24-well plates with DMEM media containing 10% fetal bovine serum (FBS), 100 U/mL penicillin, and 100 μg/mL streptomycin at 37 °C in humidified atmosphere containing 5% CO_2_. MDA-MB231-luc cells (MB231 cells stably expressing a firefly luciferase gene) were cultured with MEM media containing 10% fetal bovine serum (FBS). Primary mouse mesenchymal stem cells were cultured with DMEM media containing 15% fetal bovine serum (FBS). The cells were cultured for 24 h before gene silencing experiments.

The polymer/siRNA complexes (w/w = 1–20) were diluted with 100 μL serum-free media and equilibrated for 30 min at room temperature. Before incubation with the cells, the polyplex solution was further diluted with 150 μL serum-free media. The cells were then incubated with the polyplex solutions for 6 h and replenished with 500 μL serum-containing media (10% FBS). The silencing experiments of luciferase gene were further continued for 18 h. Luciferase expressions in the cells were analyzed according to the manufacturer’s protocols (Promega). Protein concentration in each well was determined using a BCA Protein Assay Kit (TIANGEN, China). The data was normalized to relative luciferase light unit per mg protein (RLU/mg protein), and relative to that of untreated cells (see Supplementary Appendix B). The silencing experiments of PHD-2 gene in NIH3T3 cells and Bcl-2 gene in HeLa cells were conducted for 48 h. The silencing experiments of Smurf1 gene in primary mouse mesenchymal stem cells were conducted for 24 h. The expressions of Bcl-2 mRNA in HeLa cells, PHD-2 mRNA in NIH3T3 cells or Smurf1 mRNA in primary mouse mesenchymal stem cells was analyzed by RT-PCR using specific primers described as follows.

Unmodified G5 PAMAM dendrimer and commercial transfection reagent Lipo 2000 were tested as controls. Three repeats were conducted for each material. Students’t-test was used to analyze statistically significant differences.

### Real-time PCR

PCR combined with reserve transcription was used to quantify the expressions of Bcl-2 mRNA in HeLa cells, PHD-2 mRNA in NIH3T3 cells or Smurf1 mRNA in primary mouse mesenchymal stem cells. The specific primers used are as follows:

1)  Bcl-2, forward: 5′-GGACACGGACAGGATTGACA-3′; reverse: 5′-GACATCTAAGGGCATCACAG-3′;

2)  PHD-2, forward: 5′-CCACTGGCACTCAACTAACTCA-3′; reverse: 5′-CCGAGTTCATTTAGTGCCCGTCA-3′;

3)  Smurf1, forward: 5′-CTACCAGCGTTTGGATCTAT-3′; reverse: 5′-TGTCTCGGGTCTGTAAACT -3′.

Total RNA was isolated from the transfected cells and reverse-transcribed into cDNA. The cDNA was subjected to RT-PCR analysis targeting Bcl-2, PHD-2 or Smurf1 using SYBR Green PCR Mix (TOYOBO, Osaka, Japan). The data were normalized to an endogenous reference, and relative to that of untreated cells.

### Western-blot

Western-blot was performed to evaluate the levels of PHD-2 protein expression in NIH3T3 cells and the Smurf1 protein expression in primary mouse mesenchymal stem cells. The silencing experiments of PHD-2 gene in NIH3T3 cells and Smurf1 gene in primary mouse mesenchymal stem cells were conducted for 72 h and 48 h, respectively. Transfected cells were washed three times with cold PBS and lysed in a lysis buffer. The cell lysates were separated on 10% SDS-PAGE gels and transferred onto a PVDF membrane. The membrane was incubated with rabbit monoclonal antibodies against PHD-2 and HIF-1α or Smurf1 and MEKK2 overnight, and further incubated with HRP-linked IgG antibody (goat anti-rabbit) for 1 h. The protein bands were visualized using the Odyssey CLx Infrared Imaging System (LI-COR, USA). β-Actin was measured as the endogenous reference.

### Cellular uptake

HeLa-luc cells were plated in 24-well plates and grown until 50% confluent. The cells were then incubated with the polymer/siLuc-FAM complexes at concentrations equal to those used in transfection experiments. After incubation for 2 h at 37 °C, the cells were washed with cold PBS buffer for three times and trypsinized. The mean fluorescence intensity of incubated cells was quantitatively analyzed using flow cytometry (BD FACSCalibur, San Jose). Three repeats were conducted for each material.

### Cytotoxicity analysis

Cytotoxicity of cells treated with the polymers was determined by a 3-(4,5-Dimethylthiazol-2-yl)-2,5-diphenyltetrazolium bromide (MTT) assay according to well-established protocols. Generally, HeLa cells were plated in 96-well plates at a density around 10^4^ cells per well and cultured overnight. The cells were incubated with the materials at concentrations equal to those used in transfection experiments with or without 50 nM siRNA for 24 h. After that, 100 μL MTT containing medium (0.5 mg/mL) was added to each well. After incubation for 2 h at 37 °C, the medium was removed and 150 μL DMSO was added to dissolve the insoluble purple formazan. Absorbance of the solution in each well was detected at 490 nm by a microplate reader (Thermo Fisher Scientific, U.S.). Five repeats were conducted for each sample and the data were given as means ± S.E.M.

## Additional Information

**How to cite this article**: Liu, H. *et al*. Screening of efficient siRNA carriers in a library of surface-engineered dendrimers. *Sci. Rep*. **6**, 25069; doi: 10.1038/srep25069 (2016).

## Supplementary Material

Supplementary Information
