# Supplementary material for: Screening of efficient siRNA carriers in a library of surface-engineered dendrimers
Source: Sci Rep. 2016 Apr 28;6:25069. doi: 10.1038/srep25069 (PMC4848513; doi:10.1038/srep25069)
Supplement: Supplementary Information [file srep25069-s1.pdf]

Supplementary Information

Screening of efficient siRNA carriers in a library of surface-engineered dendrimers

Hongmei Liu,[†] *Hong Chang,*[†] *Jia Lv,*[†] *Cong Jiang, Zhenxi Li, Fei Wang, Hui Wang, Mingming Wang, Chongyi Liu, Xinyu Wang, Naimin Shao, Bingwei He, Wanwan Shen, Qiang Zhang, Yiyun Cheng**

Shanghai Key Laboratory of Regulatory Biology, School of Life Sciences, East China Normal University, Shanghai, 200241, P.R. China

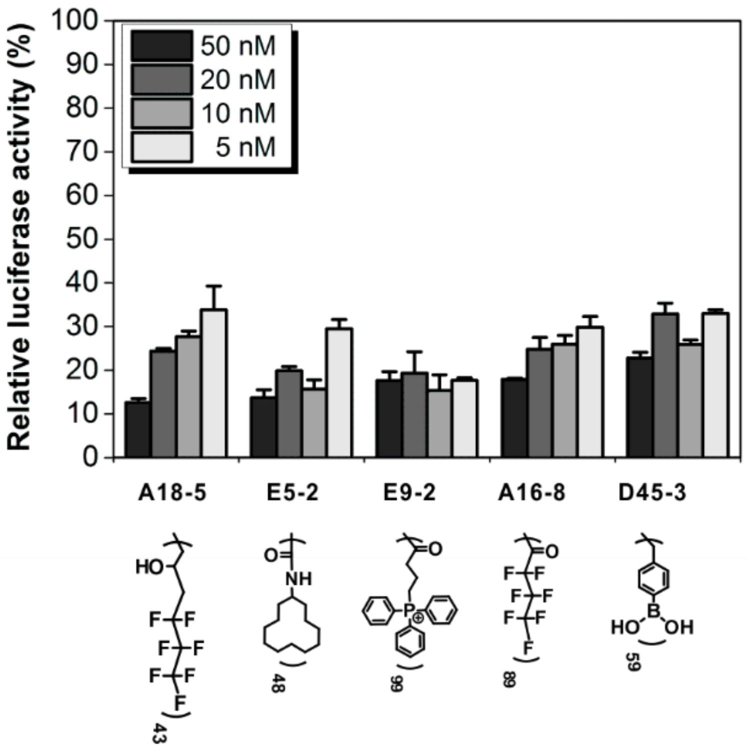

Fig. S1. siRNA dose-dependent efficacies of the screened materials. The used siRNA doses are 5, 10, 20 and 50 nM, respectively. Error bars represent the s.e. (n =3).

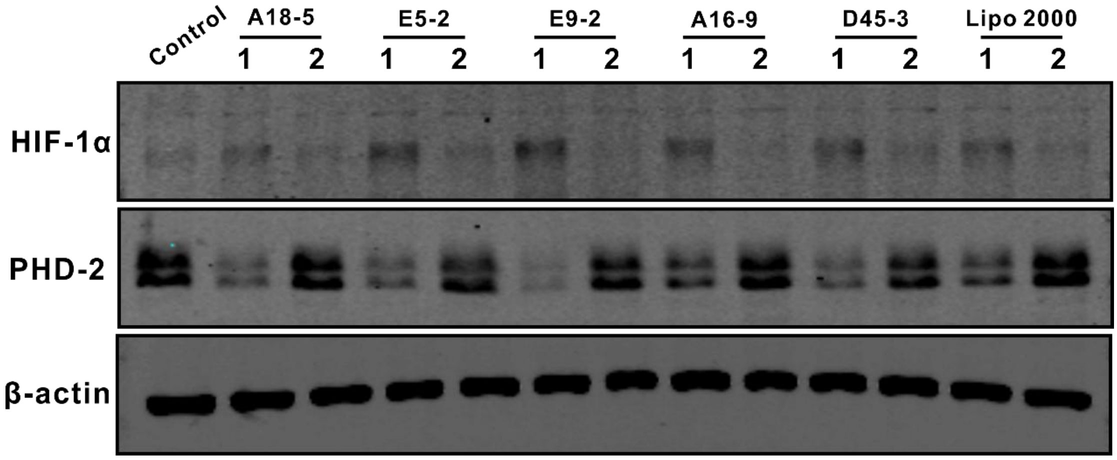

Fig. S2. Western blotting analysis for detection of PHD2 and HIF-1α in the transfected NIH3T3 cells. 50 nM siPHD2 were used. Non-transfected cells were shown as “Control”. “1” and “2’’ in above figure mean the cells were transfected with materials/siPHD2 and materials/siNC, respectively.

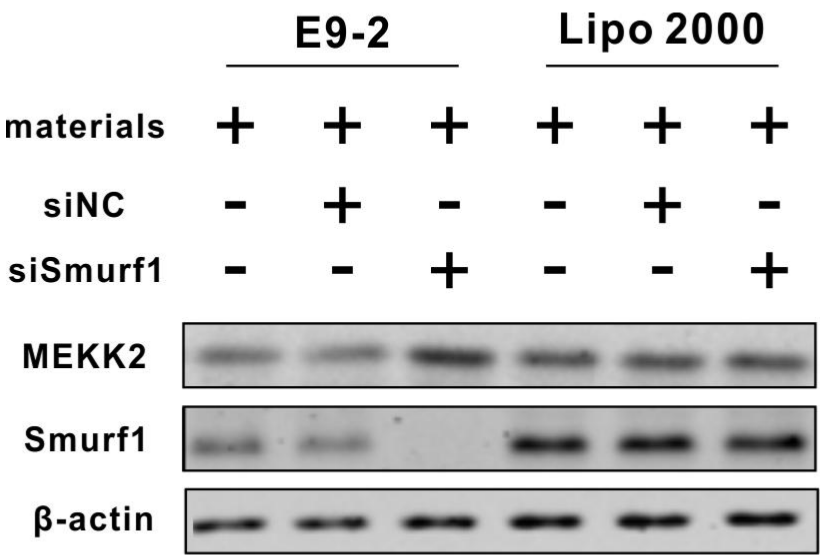

Fig. S3. Western blotting analysis for detection of Smurf1 and MEKK2 in the transfected primary mouse mesenchymal stem cells. 50 nM siSmurf1 were used.

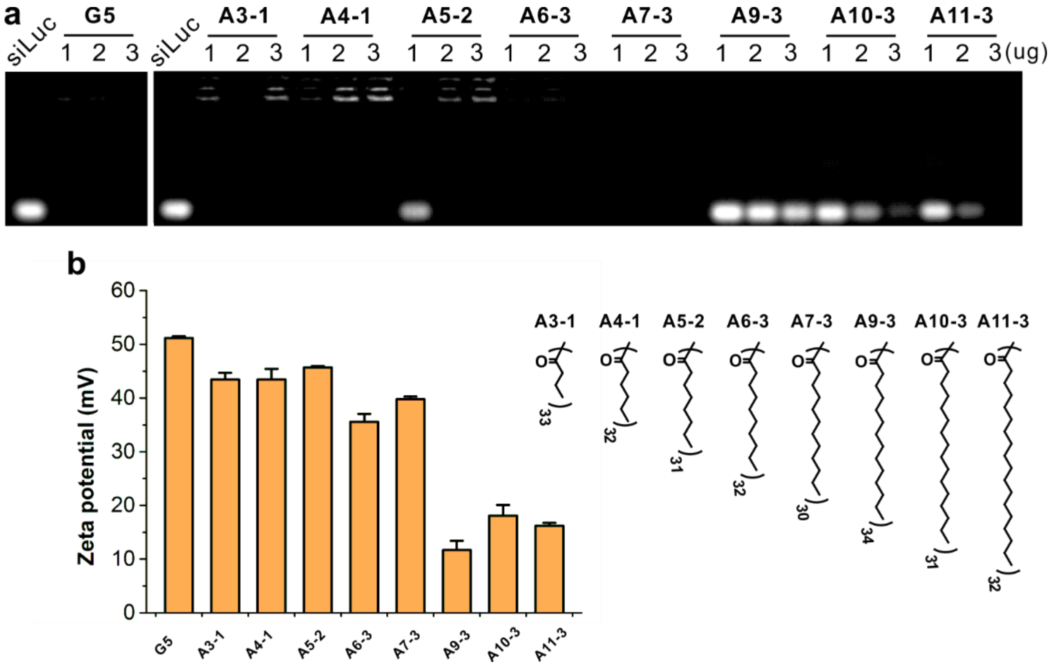

Fig. S4. Comparisons of the alkyl chain-modified dendrimers on siRNA binding capacity. (a) Agarose gel electrophoresis assay. (b) Zeta potential analysis. The zeta potentials of alkyl chain-modified dendrimers were measured at their optimal transfection concentrations. The optimal weight ratios for G5, A3-1, A4-1, A5-2, A6-3, A7-3, A9-3, A10-3 and A11-3 are 12, 12, 20, 10, 2, 3, 4, 4 and 4, respectively. 0.5 μg siLuc is used for each complex. Error bars in b represent the s.e. (n =3).

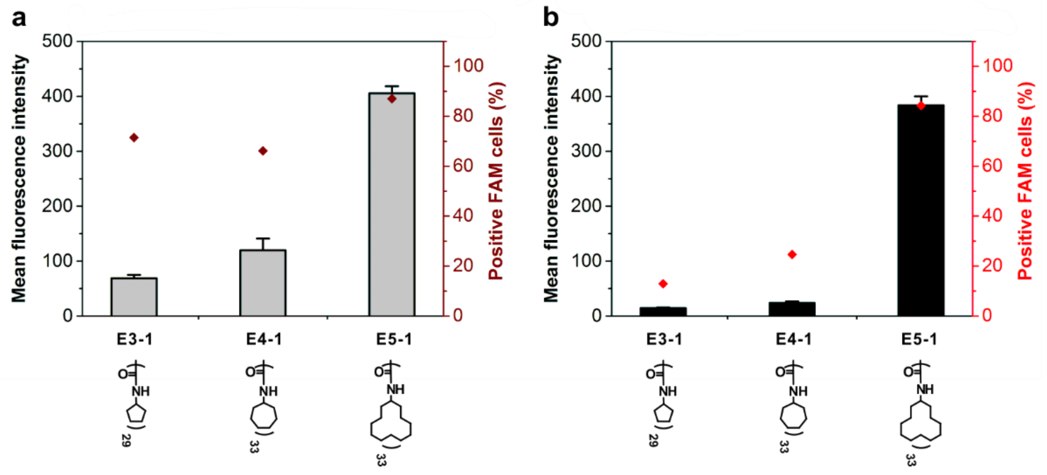

Fig. S5. Cellular uptake efficacy of cycloalkane-modified dendrimer/siRNA complexes in HeLa-Luc cells. (a) At optimal weight ratios as shown in Figure 5b. The optimal weight ratios for E3-1, E4-1 and E5-1 are 12, 8 and 2, respectively. (b) At an equal polymer molar concentration of 112 nM (112 nM is the optimal concentration for cyclododecane-modified G5 PAMAM dendrimer). 0.5 μg siLuc is used for each well and the cellular uptake experiments were conducted for 2 h. Error bars in a and b represent the s.e. (n=3).

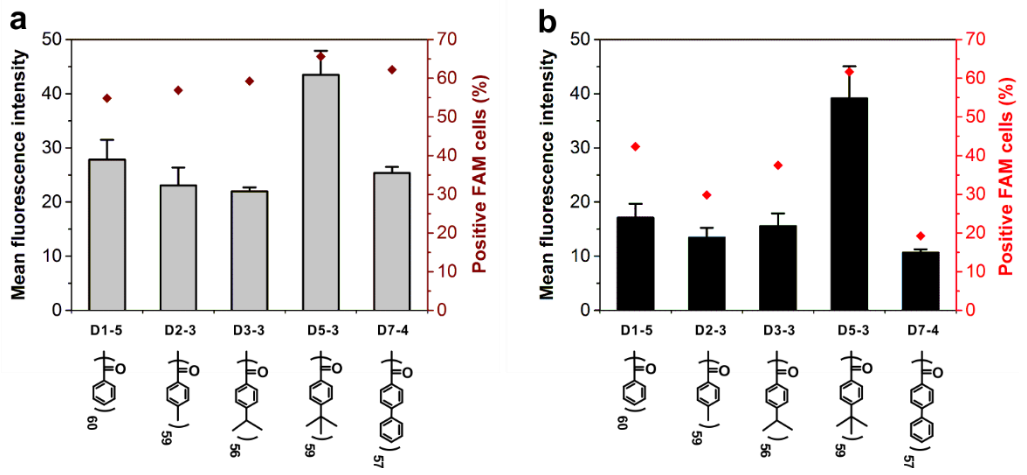

Fig. S6. Cellular uptake efficacy of aromatic ligand-modified dendrimer/siRNA complexes in HeLa-Luc cells. (a) At optimal weight ratios as shown in Figure 5c. The optimal weight ratios for D1-5, D2-3, D3-3, D5-3 and D7-4 are 12, 16, 8, 3 and 16, respectively. (b) At an equal polymer molar concentration of 157 nM (157 nM is the optimal concentration for 4-tert-butylbenzoic acid-modified G5 PAMAM dendrimer). 0.5 μg siLuc is used for each well and the cellular uptake experiments were conducted for 2 h. Error bars in a and b represent the s.e. (n=3).

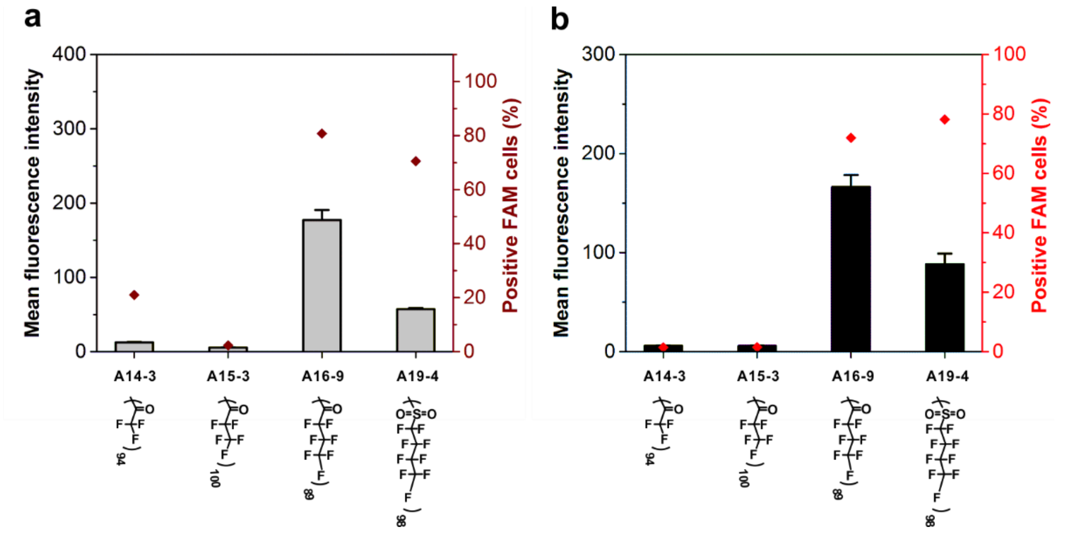

Fig. S7. Cellular uptake efficacy of fluoroalkyl chain-modified dendrimer/siRNA complexes in HeLa-Luc cells. (a) At optimal weight ratios as shown in Figure 5d. The optimal weight ratios for A14-3, A15-3, A16-9 and A19-4 are 20, 8, 3 and 20, respectively. (b) At an equal polymer molar concentration of 130 nM (130 nM is the optimal concentration for heptafluorobutyric acid-modified G5 PAMAM dendrimer). 0.5 μg siLuc is used for each well and the cellular uptake experiments were conducted for 2 h. Error bars in a and b represent the s.e. (n=3).

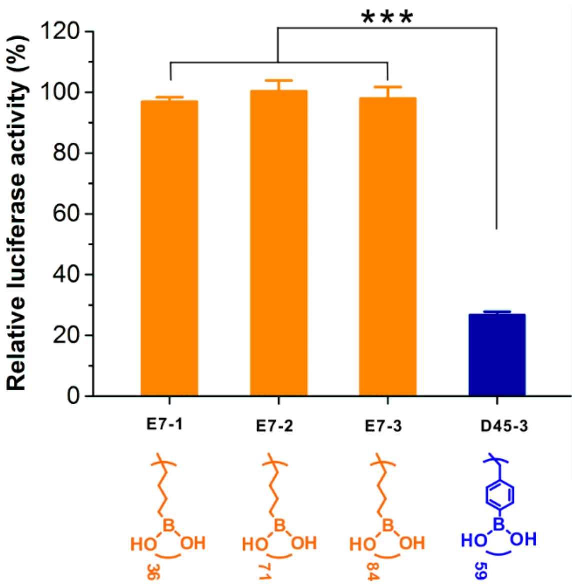

Fig. S8. Gene silencing efficacy of phenylboronic acid- and butylboronic acid-modified G5 PAMAM dendrimers. The gene silencing experiments were conducted at the optimal transfection condition for each material in HeLa-Luc cells. 50 nM siLuc is used for each transfection. The gene silencing experiments continue for 24 h. The optimal weight ratios for E7-1, E7-2, E7-3 and D45-3 are 8, 16, 4 and 20, respectively. Error bars represent the s.e. (n=3). ***P < 0.005 analyzed by students't-test.

Table S1. Characterization of surface-engineered G5 PAMAM dendrimers.

Ligand	Chemical	Reaction condition	Fed[#]	Conjugated	RLA*	NO.	Name
A1	Acetic anhydride 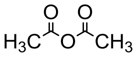	Methanol/TEA	32	33[a]	102.93	1	A1-1
			64	54[a]	105.60	2	A1-2
A2	Propionic anhydride 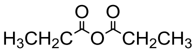	Methanol/TEA	32	30[a]	102.02	3	A2-1
			64	59[a]	107.05	4	A2-2
A3	Butyric anhydride 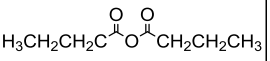	Methanol/TEA	32	33[a]	97.59	5	A3-1
			64	55[a]	97.30	6	A3-2
			68	69[a]	99.14	7	A3-3
A4	Hexanoic anhydride 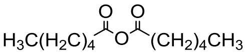	Methanol/TEA	32	32[a]	102.39	8	A4-1
			64	60[a]	100.88	9	A4-2
A5	Octanoic anhydride 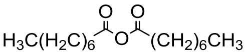	Methanol/TEA	16	15[a]	75.64	10	A5-1
			32	31[a]	62.57	11	A5-2
A6	Decanoic anhydride 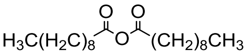	Methanol/TEA	8	8[a]	95.18	12	A6-1
			16	12[a]	85.06	13	A6-2
			32	32[a]	52.12	14	A6-3
A7	Dodecanoic anhydride 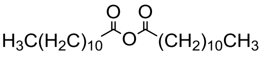	Methanol/TEA	8	9[a]	63.76	15	A7-1
			16	14[a]	39.21	16	A7-2
			32	30[a]	42.32	17	A7-3
A8	Dodecyl isocyanate $CH_3(CH_2)_{10}CH_2$-N=C=O	Methanol	8	10[a]	53.03	18	A8-1
			16	17[a]	51.89	19	A8-2
			32	32[a]	54.93	20[d]	A8-3
			48	48[a]	44.24	21[d]	A8-4

A9	Myristic acid $CH_3(CH_2)_{12}COOH$	DCC/NHS/TEA	16	3[a]	96.29	22	A9-1
			32	14[a]	103.49	23	A9-2
			48	34[a]	109.28	24[d]	A9-3
			64	41[a]	90.14	25[d]	A9-4
A10	Palmitic acid $CH_3(CH_2)_{14}COOH$	DCC/NHS/TEA	16	4[a]	94.28	26	A10-1
			32	16[a]	99.6	27	A10-2
			48	31[a]	109.90	28[d]	A10-3
A11	Stearic acid $CH_3(CH_2)_{16}COOH$	DCC/NHS/TEA	16	4[a]	95.56	29	A11-1
			32	12[a]	89.24	30	A11-2
			48	32[a]	98.79	31[d]	A11-3
A12	Oleic acid $CH_3(CH_2)_7CH{=}CH(CH_2)_7COOH$	DCC/NHS/TEA	16	4[a]	92.81	32	A12-1
			32	15[a]	90.73	33	A12-2
			48	22[a]	89.39	34	A12-3
			64	42[a]	90.22	35[d]	A12-4
A13	Linoleic acid $CH_3(CH_2)_4CH{=}CHCH_2CH{=}CH(CH_2)_7COOH$	DCC/NHS/TEA	128	43[a]	107.22	36[d]	A13-1
			256	NA	/	/	/
A14	Trifluoroacetic anhydride 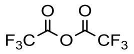	Methanol/TEA	32	26[b]	97	37	A14-1
			64	47[b]	99.66	38	A14-2
			128	94[b]	95.78	39	A14-3
A15	Pentafluoropropionic anhydride 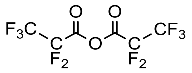	Methanol/TEA	32	36[b]	93.88	40	A15-1
			64	61[b]	106.72	41	A15-2
			128	100[b]	98.10	42	A15-3
A16	Heptafluorobutyric anhydride 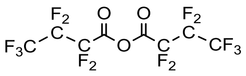	Methanol/TEA	32	24[b]	96.97	43	A16-1
			50	40[b]	104.60	44	A16-2

			55	44[b]	93.88	45	A16-3
			60	53[b]	52.49	46	A16-4
			66	59[b]	76.57	47	A16-5
			70	66[b]	32.11	48	A16-6
			72	74[b]	41.82	49	A16-7
			82	82[b]	36.68	50	A16-8
			85	89[b]	26.63	51	A16-9
			96	NA	/	/	/
A17	[2,3,3,3-Tetrafluoro-2-(trifluoromethoxy)propyl]oxirane 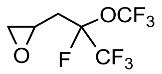	50% Ethanol	32	19[b]	90.13	52	A17-1
			64	25[b]	99.7	53	A17-2
			96	29[b]	98.93	54	A17-3
A18	3-(Perfluoropropyl)-1,2-propenoxide 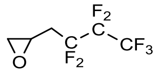	50% Ethanol	96	6[a]	88.44	55	A18-1
			128	23[a]	82.42	56	A18-2
			160	25[a]	81.09	57	A18-3
			256	34[a]	44.99	58	A18-4
			384	43[a]	19.39	59	A18-5
A19	Nonafluorobutanesulfonic anhydride 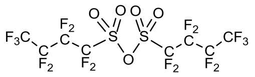	Ethanol/TEA	32	32[b]	97.05	60	A19-1
			64	63[b]	97.43	61	A19-2
			96	82[b]	90.98	62	A19-3
			128	98[b]	103.89	63	A19-4
A20	2-Chloro-4,6-bis[3-(perfluorohexyl)propyloxy]-1,3,5-triazine 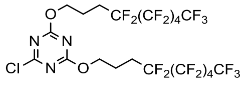	50% Ethanol 90℃	2	1.3[c]	101.47	64	A20-1
			8	8.5[c]	56.05	65	A20-2
			10	9.5[c]	68.62	66	A20-3

			20	NA	/	/	/
A21	(2,2,3,3,4,4,5,5,6,6,7,7,8,8,9,9,9-Heptadecafluorononyl)oxirane 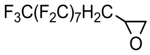	DMSO	32	5[b]	98.15	67	A21-1
			64	14[b]	80.76	68	A21-2
			88	18[b]	77.84	69	A21-3
B1	Boc-Gly-OH 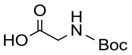	(1)DCC/NHS /TEA (2)TFA	83	52[a]	99.44	70	B1-1
			166	108[a]	88.51	71	B1-2
B2	Boc-Ala-OH 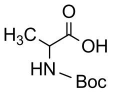	(1)DCC/NHS /TEA (2)TFA	83	73[a]	89.64	72	B2-1
			166	114[a]	107.13	73	B2-2
B3	Boc-Val-OH 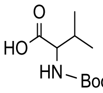	(1)DCC/NHS /TEA (2)TFA	83	77[a]	99.44	74	B3-1
			166	113[a]	105.08	75	B3-2
B4	Boc-Leu-OH 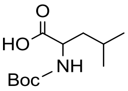	(1)DCC/NHS /TEA (2)TFA	71	71[a]	96.88	76	B4-1
			83	81[a]	93.23	77	B4-2
			166	128[a]	109.82	78	B4-3
B5	Boc-Ile-OH 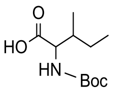	(1)DCC/NHS /TEA (2)TFA	71	67[a]	100.35	79	B5-1
			83	83[a]	90.09	80	B5-2
			166	118[a]	95.77	81	B5-3
B6	Boc-Pro-OH 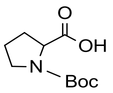	(1)DCC/NHS /TEA (2)TFA	83	41[a]	95.24	82	B6-1
			102	82[a]	107.89	83	B6-2
			166	98[a]	105.99	84	B6-3
B7	Boc-Met-OH 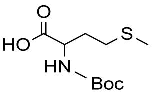	(1)DCC/NHS /TEA (2)TFA	83	68[a]	105.45	85	B7-1
			166	111[a]	98.38	86	B7-2
B8	Boc-Phe-OH 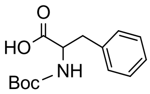	(1)DCC/NHS /TEA (2)TFA	83	71[a]	92.99	87	B8-1
			166	99[a]	54.3	88	B8-2

B9	Boc-Trp(Boc)-OH 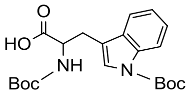	(1)DCC/NHS /TEA (2)TFA	83	55[a]	77.63	89	B9-1
			166	89[a]	39.69	90	B9-2
			256	107[a]	43.30	91	B9-3
B10	5-Fluorotryptophan 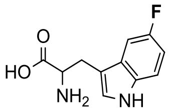	DCC/NHS/TEA	83	3[a]	88.76	92	B10-1
			166	23[a]	93.34	93[d]	B10-2
B11	Boc-Tyr(tBu)-OH 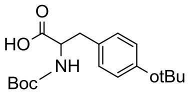	(1)DCC/NHS /TEA (2)TFA	83	68[a]	92.75	94	B11-1
			166	98[a]	78.38	95	B11-2
B12	Boc-Ser(tBu)-OH 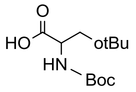	(1)DCC/NHS /TEA (2)TFA	83	74[a]	101.44	96	B12-1
			166	113[a]	105.55	97	B12-2
B13	Boc-Thr(tBu)-OH 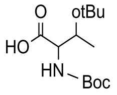	(1)DCC/NHS /TEA (2)TFA	71	75[a]	104.71	98	B13-1
			83	88[a]	92.34	99	B13-2
			166	126[a]	103.05	100	B13-3
B14	Boc-Cys(Acm)-OH 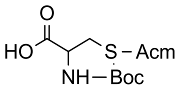	(1)DCC/NHS /TEA (2)I_2/Methanol	83	N/A	/	/	/
			166	N/A	/	/	/
B15	Boc-Asn(Trt)-OH 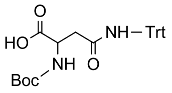	(1)DCC/NHS /TEA (2)TFA	166	75[a]	90.97	101	B15-1
			512	88[a]	101.71	102	B15-2
B16	Boc-Gln(Trt)-OH 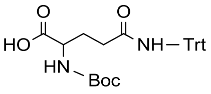	(1)DCC/NHS /TEA (2)TFA	166	71[a]	106.72	103	B16-1
			256	86[a]	109.94	104	B16-2
B17	Boc-His(Trt)-OH 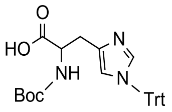	(1)DCC/NHS /TEA (2)TFA	83	65[a]	104.12	105	B17-1
			166	113[a]	108.68	106	B17-2
B18	Boc-Lys-OH 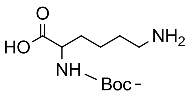	(1)DCC/NHS /TEA (2)TFA	83	67[a]	94.96	107	B18-1
			166	130[a]	93.83	108	B18-2

B19	Boc-Arg(Pbf)-OH 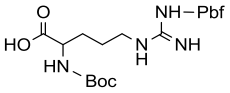	(1)DCC/NHS /TEA (2)TFA	45	33[a]	100.09	109	B19-1
			83	64[a]	100.16	110	B19-2
			166	110[a]	43.73	111	B19-3
B20	Boc-Asp(otBu)-OH 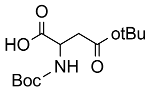	(1)DCC/NHS /TEA (2)TFA	83	54[a]	105.61	112	B20-1
			166	98[a]	85.96	113	B20-2
B21	Boc-Glu(otBu)-OH 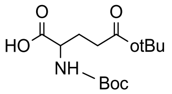	(1)DCC/NHS /TEA (2)TFA	71	48[a]	97.36	114	B21-1
			134	81[a]	91.94	115	B21-2
B22	Boc-Lys(Ac)-OH 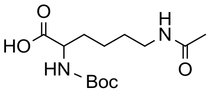	(1)DCC/NHS /TEA (2)TFA	192	124[a]	100.52	116	B22-1
B23	Ac-Lys(Boc)-OH 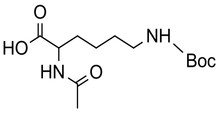	(1)DCC/NHS /TEA (2)TFA	96	84[a]	98.03	117	B23-1
			192	121[a]	100.53	118	B23-2
C1	Nicotinic acid 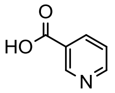	DCC/NHS/TEA	32	22[a]	99.89	119	C1-1
			64	50[a]	104.06	120[d]	C1-2
C2	2-(Chloromethyl)pyridine hydrochloride 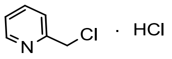	50% Ethanol 80℃	64	16[a]	94.46	121	C2-1
			96	30[a]	100.27	122	C2-2
			128	51[a]	104.17	123	C2-3
C3	4-(Chloromethyl)pyridine hydrochloride 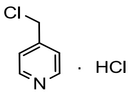	50% Ethanol 80℃	64	22[a]	91.06	124	C3-1
			96	24 [a]	90.69	125	C3-2
			128	26 [a]	102.54	126	C3-3
C4	Coumalic acid 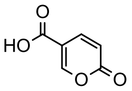	DCC/NHS/TEA	32	NA	/		
			64	NA	/		
C5	3-Fluoro-4-pyridinecarboxylic acid	DCC/NHS/TEA	32	11[a]	100.98	127	C5-1

	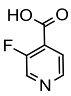		64	27[a]	100.44	128	C5-2
			96	34[a]	96.09	129	C5-3
			128	40[a]	110.60	130	C5-4[d]
C6	Trigonelline hydrochloride 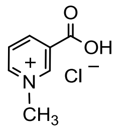	EDC/NHS	64	NA	/		
C7	2,3,5-Trifluoropyridine-4-carboxylic acid 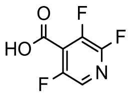	DCC/NHS/TEA	32	4[a]	91.22	131	C7-1
			64	7[a]	103.45	132	C7-2
C8	2-Bromopyrimidine 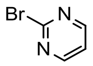	50% Ethanol 80℃	32	16[a]	101.76	133	C8-1
			64	33[a]	102.63	134	C8-2
			128	46[a]	102.74	135	C8-3
C9	2-Chloro-4,6-diamino-1,3,5-triazine 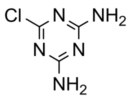	50% Ethanol 80℃	32	29[a]	92.97	136	C9-1
			51	46[a]	94.86	137	C9-2
			64	55[a]	93.23	138	C9-3
			77	60[a]	101.02	139	C9-4
			96	78[a]	94.37	140[d]	C9-5
C10	2-Chloro-4,6-dimethoxy-1,3,5-triazine 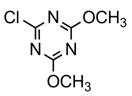	50% Ethanol 80℃	32	25[a]	99.39	141	C10-1
			64	55[a]	96.50	142	C10-2
C11	6-Chloropurine 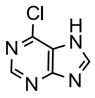	50% Ethanol 80℃	32	15[a]	100.86	143	C11-1
			64	27[a]	100.51	144	C11-2
			128	48[a]	98.04	145	C11-3

C12	2-Amino-6-chloropurine 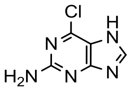	50% Ethanol 80℃	32	14[a]	90.21	146	C12-1
			64	26[a]	110.11	147	C12-2
			128	32[a]	102.49	148	C12-3
C13	2-Thiophenecarboxylic acid 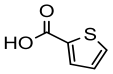	DCC/NHS/TEA	32	16[a]	86.90	149	C13-1
			64	47[a]	92.97	150[d]	C13-2
C14	2-Furoic acid 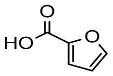	DCC/NHS/TEA	32	14[a]	90.52	151	C14-1
			64	37[a]	104.12	152[d]	C14-2
C15	Pyrrole-2-carboxylic acid 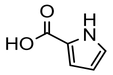	DCC/NHS/TEA	32	11[a]	88.65	153	C15-1
			64	31[a]	93.03	154	C15-2
C16	3-Quinolinecarboxylic acid 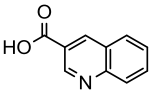	DCC/NHS/TEA	32	5[a]	97.87	155	C16-1
			64	49[a]	88.63	156[d]	C16-2
C17	6-(1H-Pyrazol-1-yl)nicotinic acid 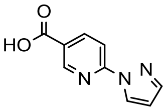	DCC/NHS/TEA	32	24[a]	93.12	157[d]	C17-1
			64	40[a]	84.37	158[d]	C17-2
C18	3-Indoleacetic acid 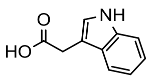	DCC/NHS/TEA	32	22[a]	92.77	159	C18-1
			64	42[a]	96.42	160[d]	C18-2
			142	94[a]	104.56	161[d]	C18-3
			160	100[a]	104.92	162[d]	C18-4
			172	106[a]	97.00	163[d]	C18-5
C19	4-Imidazoleacrylic acid 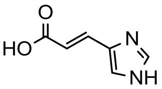	DCC/NHS/TEA	32	8[a]	87.31	164	C19-1
			64	46[a]	103.09	165[d]	C19-2
C20	2-Benzimidazolepropionic acid	DCC/NHS/TEA	32	21[a]	95.07	166	C20-1

	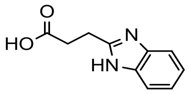		64	50[a]	103.01	167[d]	C20-2
C21	Fmoc-L-1,2,3,4-Tetrahydroisoquinoline-3-carboxylic acid 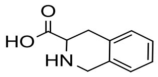	DCC/NHS/TEA	32	19[a]	87.06	168[d]	C21-1
		DCC/NHS/TEA	64	40[a]	94.06	169[d]	C21-2
D1	Benzoic acid 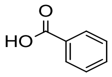	DCC/NHS/TEA	19	7[a]	84.13	170	D1-1
		DCC/NHS/TEA	38	19[a]	93.31	171	D1-2
		DCC/NHS/TEA	48	27[a]	102.10	172	D1-3
		DCC/NHS/TEA	58	46[a]	103.69	173[d]	D1-4
		DCC/NHS/TEA	64	60[a]	97.56	174[d]	D1-5
		DCC/NHS/TEA	70	67[a]	102.10	175[d]	D1-6
		DCC/NHS/TEA	82	77[a]	98.10	176[d]	D1-7
		DCC/NHS/TEA	96	85[a]	84.99	177[d]	D1-8
		DCC/NHS/TEA	128	99[a]	106.61	178[d]	D1-9
D2	p-Toluic acid 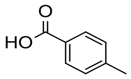	DCC/NHS/TEA	61	24[a]	101.42	179	D2-1
		DCC/NHS/TEA	76	42[a]	92.01	180[d]	D2-2
		DCC/NHS/TEA	96	59[a]	91.60	181[d]	D2-3
		DCC/NHS/TEA	102	62[a]	87.65	182[d]	D2-4
		DCC/NHS/TEA	128	103[a]	103.85	183[d]	D2-5
D3	4-Isopropylbenzoic acid 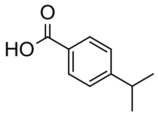	DCC/NHS/TEA	32	15[a]	102.09	184	D3-1
		DCC/NHS/TEA	76	45[a]	56.14	185[d]	D3-2
		DCC/NHS/TEA	86	56[a]	59.77	186[d]	D3-3

D4	4-Vinylbenzoic acid 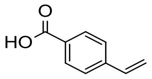	DCC/NHS/TEA	32	16[a]	102.51	187	D4-1
		DCC/NHS/TEA	64	53[a]	77.59	188[d]	D4-2
D5	4-tert-Butylbenzoic acid 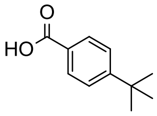	DCC/NHS/TEA	32	17[a]	97.19	189	D5-1
		DCC/NHS/TEA	50	37[a]	47.43	190[d]	D5-2
		DCC/NHS/TEA	64	59[a]	48.63	191[d]	D5-3
D6	4-Phenylbutyric acid 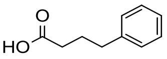	DCC/NHS/TEA	64	51[a]	75.5	192[d]	D6-1
		DCC/NHS/TEA	128	105[a]	101.08	193[d]	D6-2
D7	Biphenyl-4-carboxylic acid 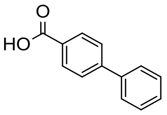	DCC/NHS/TEA	13	3[a]	97.85	194	D7-1
		DCC/NHS/TEA	26	10[a]	92.76	195	D7-2
		DCC/NHS/TEA	64	29[a]	96.46	196	D7-3
		DCC/NHS/TEA	96	57[a]	96.30	197[d]	D7-4
D8	1-naphthoic acid 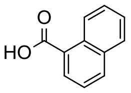	DCC/NHS/TEA	32	26[a]	91.49	198	D8-1
		DCC/NHS/TEA	64	40[a]	96.48	199	D8-2
D9	9-Anthroic acid 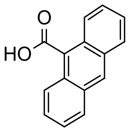	DCC/NHS/TEA	32	NA	/	/	/
		DCC/NHS/TEA	64	NA	/	/	/
D10	4-(4-Pyridyl)benzoic acid 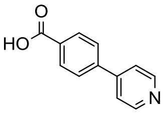	DCC/NHS/TEA	32	9[a]	91.71	200	D10-1
D11	Boc-4-Abz-OH 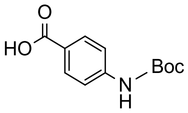	DCC/NHS/TEA	32	14[a]	101.05	201	D11-1
		DCC/NHS/TEA	64	44[a]	103.70	202	D11-2
D12	Boc-4-aminophenylacetic acid	DCC/NHS/TEA	26	8[a]	91.11	203	D12-1

	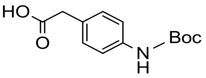	DCC/NHS/TEA	51	22[a]	102.41	204	D12-2
D13	Boc-3-Abz-OH 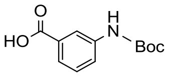	DCC/NHS/TEA	26	7[a]	94.65	205	D13-1
		DCC/NHS/TEA	51	18[a]	103.81	206	D13-2
		DCC/NHS/TEA	64	34[a]	98.30	207	D13-3
		DCC/NHS/TEA	128	NA	/	/	/
D14	4-(Boc-aminomethyl)benzoic acid 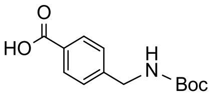	DCC/NHS/TEA	26	8[a]	99.71	208	D14-1
		DCC/NHS/TEA	51	21[a]	104.10	209	D14-2
		DCC/NHS/TEA	64	40[a]	92.80	210	D14-3
		DCC/NHS/TEA	128	103[a]	62.33	211	D14-4
D15	4-Hydrazinobenzoic acid 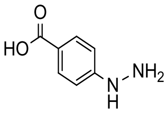	DCC/NHS/TEA	32	7[a]	108.63	212	D15-1
		DCC/NHS/TEA	64	NA	/	/	/
D16	4-(1H-Imidazol-1-yl)benzoic acid 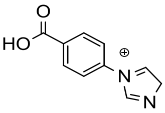	DCC/NHS/TEA	32	11[a]	105.21	213	D16-1
		DCC/NHS/TEA	64	38[a]	113.73	214[d]	D16-2
		DCC/NHS/TEA	96	66[a]	93.10	215[d]	D16-3
D17	5-Benzimidazolecarboxylic acid 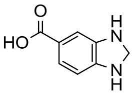	DCC/NHS/TEA	32	21[a]	94.47	216[d]	D17-1
		DCC/NHS/TEA	64	52[a]	107.11	217[d]	D17-2
D18	Di-Fmoc-3.5-diaminobenzic acid 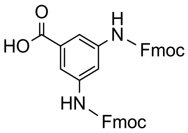	DCC/NHS/TEA	26	5[a]	86.12	218	D18-1
		DCC/NHS/TEA	51	17[a]	97.94	219	D18-2
		DCC/NHS/TEA	64	26[a]	95.62	220	D18-3
D19	3-fluorobenzoic acid 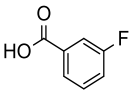	DCC/NHS/TEA	26	27[a]	97.21	221	D19-1
		DCC/NHS/TEA	64	30[a]	93.11	222	D19-2

D20	3,5-Difluorobenzoic acid<br>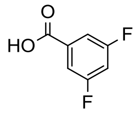	DCC/NHS/TEA	32	25[a]	100.35	223	D20-1
		DCC/NHS/TEA	64	59[a]	94.47	224[d]	D20-2
D21	2,3,4-Trifluorobenzoic acid<br>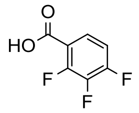	DCC/NHS/TEA	16	6[a]	83.16	225	D21-1
		DCC/NHS/TEA	32	14[a]	91.22	226	D21-2
		DCC/NHS/TEA	64	28[a]	100.51	227	D21-3
		DCC/NHS/TEA	96	34[a]	96.54	228	D21-4
		DCC/NHS/TEA	256	107[a]	104.14	229[d]	D21-5
D22	2,3,4-Trifluorobenzaldehyde<br>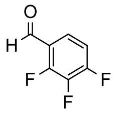	DMSO	32	2[a]	79.31	230	D22-1
		DMSO	64	4[a]	84.48	231	D22-2
D23	2,3,5,6-Tetrafluoro-p-toluic acid<br>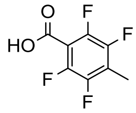	DCC/NHS/TEA	16	13[a]	83.36	232	D23-1
		DCC/NHS/TEA	24	19[a]	89.73	233	D23-2
		DCC/NHS/TEA	32	25[a]	91.09	234	D23-3
		DCC/NHS/TEA	64	44[a]	37.72	235[d]	D23-4
D24	2,3,4,5,6-Pentafluorobenzoic acid<br>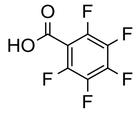	DCC/NHS/TEA	8	NA	/	/	/
		DCC/NHS/TEA	17	NA	/	/	/
		DCC/NHS/TEA	24	NA	/	/	/
D25	4-(Trifluoromethyl)benzoic acid<br>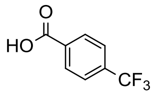	DCC/NHS/TEA	32	18[a]	106.17	236	D25-1
D26	4-(Trifluoromethyl)phenyl isothiocyanate<br>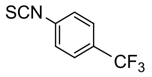	DMSO	32	31[a]	81.85	237	D26-1
		DMSO	64	63[a]	71.88	238[d]	D26-2
D27	3,5-Bis(trifluoromethyl)benzaldehyde	DMSO	32	27[b]	88.15	239	D27-1

	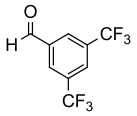	DMSO	64	43[b]	95.32	240	D27-2
D28	4-(Trifluoromethoxy)benzoic acid 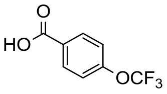	DCC/NHS/TEA	32	17[a]	105.37	241	D28-1
		DCC/NHS/TEA	64	47[a]	69.21	242[d]	D28-2
D29	3,4,5-Trimethoxyphenyl isocyanate 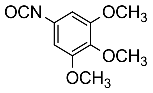	DMSO/TEA	64	NA	/	/	/
D30	4-Chlorobenzoic acid 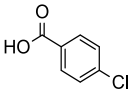	DCC/NHS/TEA	32	12[a]	96.44	243[d]	D30-1
		DCC/NHS/TEA	64	32[a]	90.85	244[d]	D30-2
D31	2,4,6-Trichlorobenzoic acid 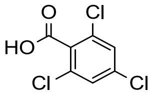	DCC/NHS/TEA	32	9[a]	107.55	245	D31-1
		DCC/NHS/TEA	64	18[a]	103.70	246[d]	D31-2
D32	4-Bromobenzoic acid 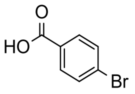	DCC/NHS/TEA	32	11[a]	91.10	247	D32-1
		DCC/NHS/TEA	64	44[a]	43.61	248[d]	D32-2
D33	4-Bromophenyl isocyanate 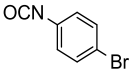	DMSO	32	32[a]	87.44	249[d]	D33-1
D34	4-Bromobenzaldehyde 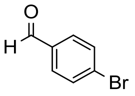	DMSO	32	NA	/	/	/
		DMSO	64	21[a]	87.71	250[d]	D34-1
D35	2-Bromobenzoic aicd 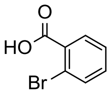	DCC/NHS/TEA	32	14[a]	93.28	251[d]	D35-1
		DCC/NHS/TEA	64	41[a]	72.72	252[d]	D35-2
		DCC/NHS/TEA	96	53[a]	83.03	253[d]	D35-3
		DCC/NHS/TEA	128	119[a]	103.74	254[d]	D35-4
D36	4-Iodobenzoic acid	DCC/NHS/TEA	32	16[a]	102.55	255	D36-1

	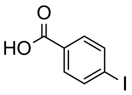	DCC/NHS/TEA	64	46[a]	73.62	256[d]	D36-2
		DCC/NHS/TEA	96	75[a]	75.18	257[d]	D36-3
		DCC/NHS/TEA	128	121[a]	108.01	258[d]	D36-4
D37	2-Iodobenzoic acid 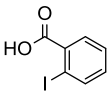	DCC/NHS/TEA	32	NA	/	/	/
		DCC/NHS/TEA	64	NA	/	/	/
D38	2,3,5-Triiodobenzoic acid 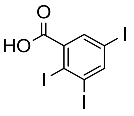	DCC/NHS/TEA	32	NA	/	/	/
		DCC/NHS/TEA	64	NA	/	/	/
D39	Diatrizoic acid 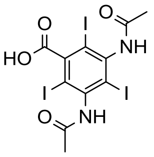	DCC/NHS/TEA	32	29[b]	98.91	259	D39-1
		DCC/NHS/TEA	64	34[b]	94.98	260	D39-2
D40	4-Hydroxybenzoic acid 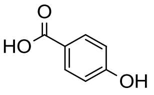	DCC/NHS/TEA	48	14[a]	90.65	261[d]	D40-1
		DCC/NHS/TEA	64	25[a]	96.03	262[d]	D40-2
		DCC/NHS/TEA	82	39[a]	97.73	263[d]	D40-3
D41	Salicylic acid 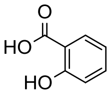	DCC/NHS/TEA	32	16[a]	103.19	264	D41-1
		DCC/NHS/TEA	64	42[a]	102.27	265[d]	D41-2
D42	2,4-Dihydroxybenzoic acid 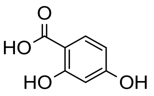	DCC/NHS/TEA	32	6[a]	97.64	266	D42-1
		DCC/NHS/TEA	64	12[a]	103.39	267	D42-2
		DCC/NHS/TEA	128	14[a]	101.37	268	D42-3[d]
D43	3,4-Dihydroxyphenylacetic acid 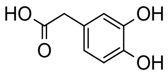	DCC/NHS/TEA	32	8[a]	104.89	269	D43-1
		DCC/NHS/TEA	64	40[a]	105.11	270[d]	D43-2
D44	Gallic acid monohydrate 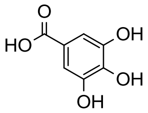	DCC/NHS/TEA	32	NA	/	/	/
		DCC/NHS/TEA	64	NA	/	/	/

D45	4-(Bromomethyl)phenylboronic acid 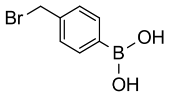	DMSO 80℃	64	46[a]	59.38	271	D45-1
		DMSO 80℃	96	56[a]	49.30	272	D45-2
		DMSO 80℃	128	59[a]	17.74	273	D45-3
D46	4-Carboxybenzeneboronic acid pinacol ester 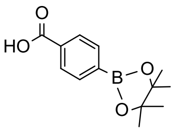	DMSO 80℃	32	NA	/	/	/
		DMSO 80℃	64	NA	/	/	/
D47	4-Methoxybenzoic acid 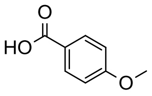	DCC/NHS/TEA	32	14[a]	97.72	274	D47-1
		DCC/NHS/TEA	64	50[a]	104.35	275[d]	D47-2
D48	4-Phenoxyphenyl isocyanate 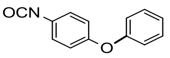	DMSO	32	35[a]	65.53	276[d]	D48-1
D49	4-Cyanobenzoic acid 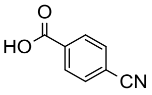	DCC/NHS/TEA	32	13[a]	101.84	277	D49-1
		DCC/NHS/TEA	64	29[a]	100.85	278[d]	D49-2
D50	4-Fluoro-3-nitrobenzoic Acid 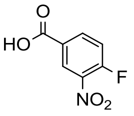	DCC/NHS/TEA	16	NA	/	/	/
		DCC/NHS/TEA	32	NA	/	/	/
		DCC/NHS/TEA	64	NA	/	/	/
D51	4-Nitrobenzoic acid 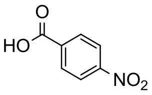	DCC/NHS/TEA	32	12[a]	98.46	279	D51-1
D52	4-Mercaptobenzoic acid 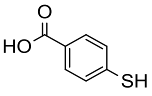	DCC/NHS/TEA	32	NA	/	/	/
		DCC/NHS/TEA	64	NA	/	/	/
D53	4-(Methylthio)phenyl isocyanate 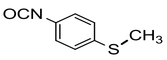	DMSO/TEA	64	NA	/	/	/

D54	4-Sulfamylbenzoic acid 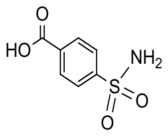	DCC/NHS/TEA	32	14[a]	94.59	280	D54-1
D55	Ferrocenecarboxylic acid 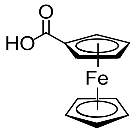	DCC/NHS/TEA	32	NA	/	/	/
		DCC/NHS/TEA	64	NA	/	/	/
E1	1-Amidinopyrazole Hydrochloride 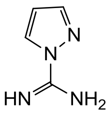	DIPEA/H_2O	66	42[b]	99.27	281	E1-1
			110	60[b]	96.77	282	E1-2
E2	Guanidineacetic acid 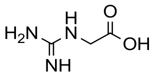	DCC/NHS/TEA	32	12[b]	85.88	283	E2-1
			64	19[b]	100.42	284	E2-2
			83	57[b]	95.88	285	E2-3
			166	32[b]	64.02	286	E2-4
E3	Cyclopentyl isocyanate 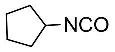	DMSO	32	29[a]	101.08	287	E3-1
			64	60[a]	89.09	288	E3-2
E4	Cycloheptyl isocyanate 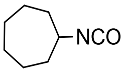	DMSO	32	33[a]	91.93	289	E4-1
			64	64[a]	64.31	290[d]	E4-2
E5	Cyclododecyl isocyanate 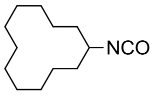	DMSO	32	33[a]	33.97	291[d]	E5-1
			48	48[a]	25.87	292[d]	E5-2
E6	3-bromopropylboronic acid pinacol ester 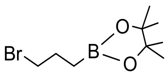	DMSO, 80℃	64	9[a]	103.61	293	E6-1
			128	20[a]	106.75	294	E6-2
E7	4-bromobutylboronic acid 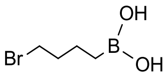	DMSO, 80℃	64	36[a]	97.90	295	E7-1
			96	71[a]	85.96	296	E7-2

			128	84[a]	91.74	297	E7-3
E8	R-(+)-2-Pyrrolidone-5-carbo 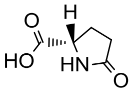 xylic acid	DCC/NHS/TEA	32	16[a]	94.53	298	E8-1
			64	28[a]	92.37	299	E8-2
E9	(3-Carboxypropyl)triphenylphosphonium bromide 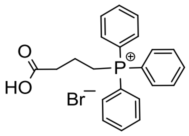	DCC/NHS/TEA	90	35[a]	46.0	300	E9-1
			180	99[a]	13.9	301	E9-2

TEA is abbreviation of triethylamine; DCC is abbreviation of dicyclohex-ylcarbodiimide; NHS is abbreviation of N-hydroxysuccinimide; DMSO is abbreviation of dimethyl sulfoxide; TFA is abbreviation of trifluoroacetic acid; DIPEA is abbreviation of N, N-diisopropylethylamine.

Fed[#] is molar ratio of the chemical to G5 PAMAM dendrimer during the synthesis of surface-engineered dendrimers. In this manuscript, most of the surface-engineered dendrimers were synthesized at feeding molar ratios of 32 and 64. Amino acid-modified dendrimers were synthesized at feeding molar ratios of 83 and 166 according to a previous reference (*Biomaterials*, 2014, 35, 9187-9198). Depending on different conjugation efficacies of the reactions in Table S1, the feeding ratios were varied in a proper range. For example, if the conjugation efficacy is low, larger feeding ratios will be chosen, and if the conjugated product is insoluble in water or DMSO, lower feeding ratios will be chosen.

RLA* is relative luciferase activity to untreated cells.

NO. means product number.

[a] means the products are characterized by ^{1}H NMR; [b] means the products are characterized by the ninhydrin assay and [c] means the products are characterized by fluorine element analysis (CAS Shanghai Institute of Organic Chemistry, China); [d] means the products are dissolved in DMSO; The solvent DMSO within the concentration range of 0-18.77 mM has no effect on luciferase gene silencing.

NA means the products are insoluble in water or DMSO.

Table S2. Characterization of surface-engineered G5 PAMAM dendrimers in the second-generation library.

Ligand	Chemical	Reaction condition	Fed[#]	Conjugated	RLA*	Name
S1	Adamantyl isocyanate 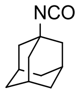	DMSO	32	33[a]	85.86	S1-1
			64	63[a]	16.28	S1-2[d]
S2	Glycidyl 2,2,3,3,4,4,5,5-octafluoropentyl ether 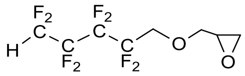	50% Ethanol	64	22[a]	97.05	S2-1
			96	41[a]	34.44	S2-2
			128	71[a]	13.77	S2-3[d]
			160	93[a]	34.57	S2-4[d]
S3	3,5-Dibromobenzoic acid 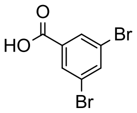	DCC/NHS/TEA	64	46[b]	11.56	S3-1[d]
S4	3,5-Bis(trifluoromethyl)phenyl isothiocyanate 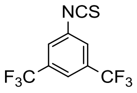	DMSO	32	26[a]	19.57	S4-1[d]
			64	58[a]	87.97	S4-2[d]
S5	Fmoc-L-4-Fluorophe 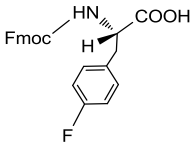	DCC/NHS/ Piperidine	64	68[a]	39.35	S5-1
S6	2-Fluoro-3-(trifluoromethyl)benz 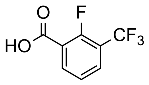 oic acid	DCC/NHS/TEA	32	17[a]	94.59	S6-1[d]
			64	40[a]	56.07	S6-2[d]
S7	4-Hexylbenzoic acid 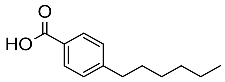	DCC/NHS/TEA	64	24[a]	68.83	S7-1[d]
			82	64[a]	49.94	S7-2[d]
			96	83[a]	69.84	S7-3[d]

S8	3-Bromobenzoic acid 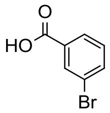	DCC/NHS/TEA	64	39[a]	98.95	S8-1[d]
			96	83[a]	59.65	S8-2[d]

DMSO is abbreviation of dimethyl sulfoxide; DCC is abbreviation of dicyclohex-ylcarbodiimide; NHS is abbreviation of N-hydroxysuccinimide; TEA is abbreviation of Triethylamine.

Fed# is molar ratio of the chemical to G5 PAMAM dendrimer during the synthesis of surface-engineered dendrimers;

RLA* is relative luciferase activity to untreated cells (normalized to relative luciferase light unit per mg protein, and relative to that of untreated cells);

[a] means the products are characterized by ^{1}H NMR; [b] means the products are characterized by the ninhydrin assay and [c] means the products are characterized by fluorine element analysis (CAS Shanghai Institute of Organic Chemistry, China); [d] means the products are dissolved in dimethyl sulphoxide. The solvent DMSO within the concentration range of 0-18.77 mM has no effect on luciferase gene silencing.

Appendix A. 1H NMR spectra of the surface-engineered dendrimers in the library.

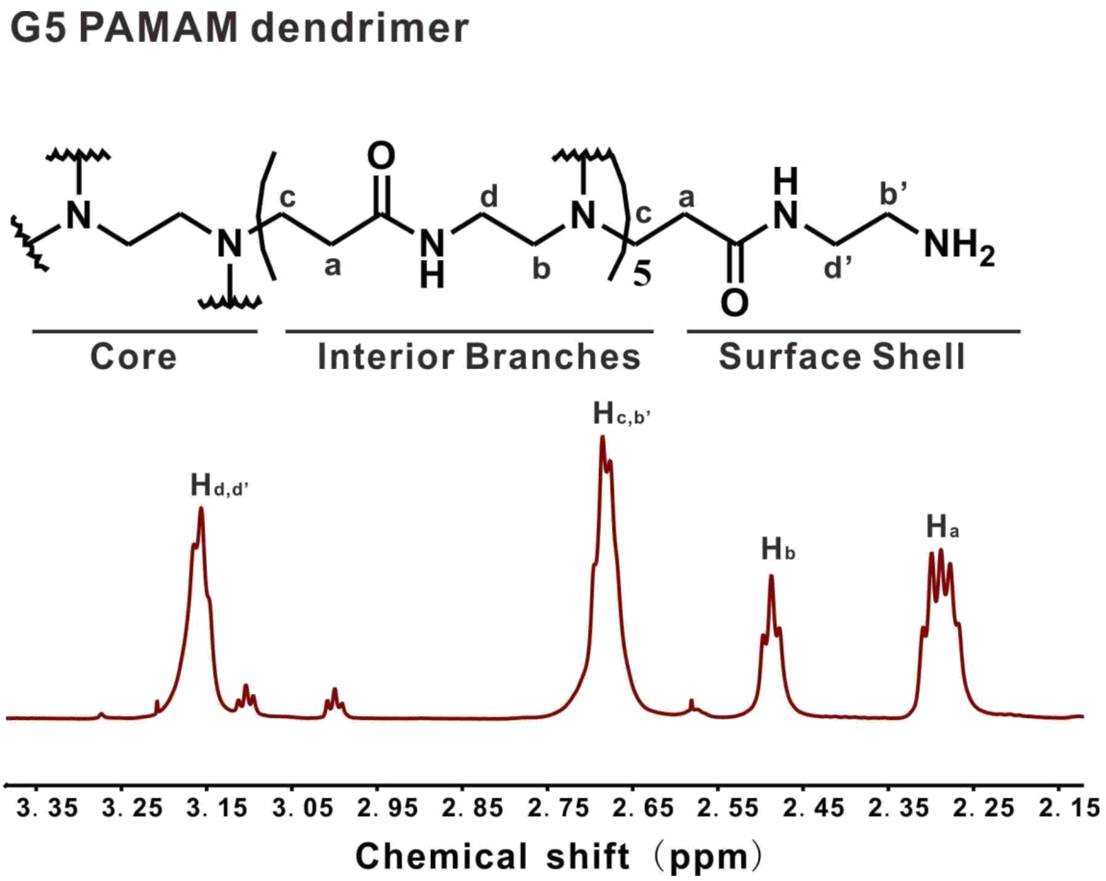

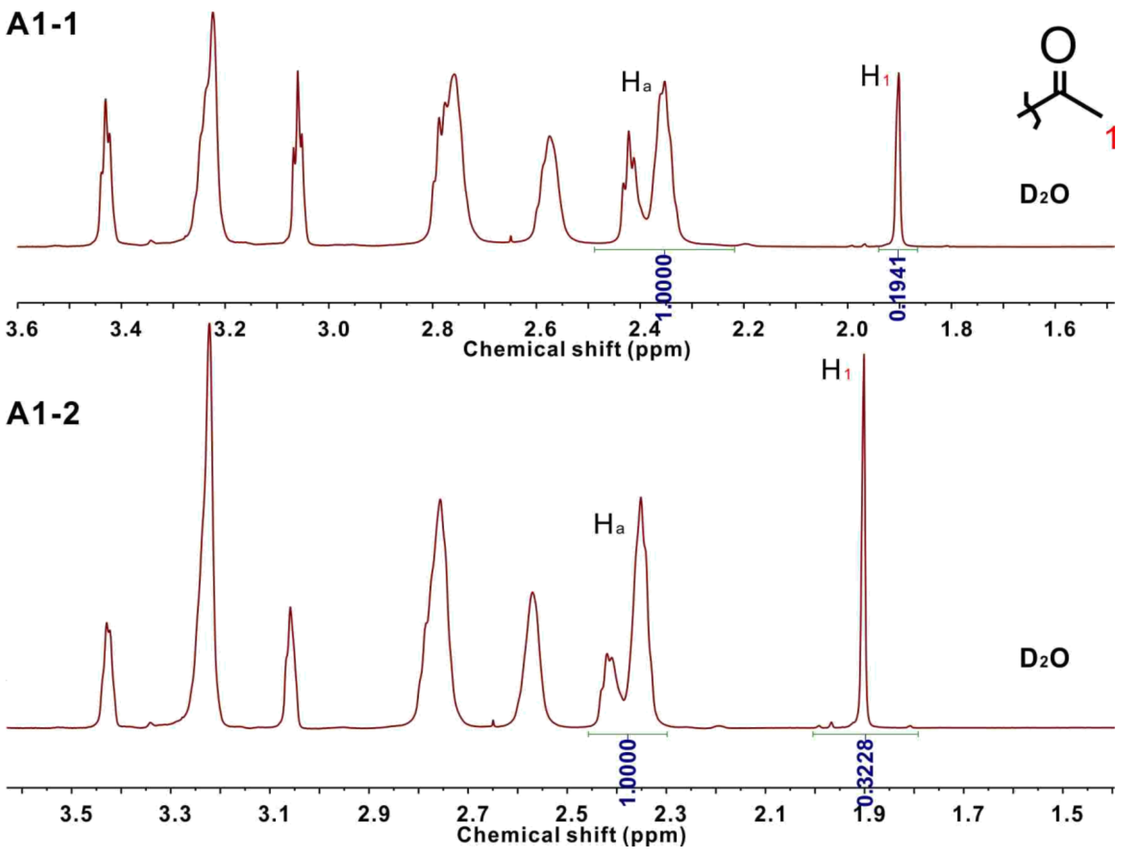
A1-1
Ha
H1
1
D2O
1.0000
0.1941
3.6 3.4 3.2 3.0 2.8 2.6 2.4 2.2 2.0 1.8 1.6
Chemical shift (ppm)
A1-2
Ha
H1
D2O
1.0000
0.3228
3.5 3.3 3.1 2.9 2.7 2.5 2.3 2.1 1.9 1.7 1.5
Chemical shift (ppm)

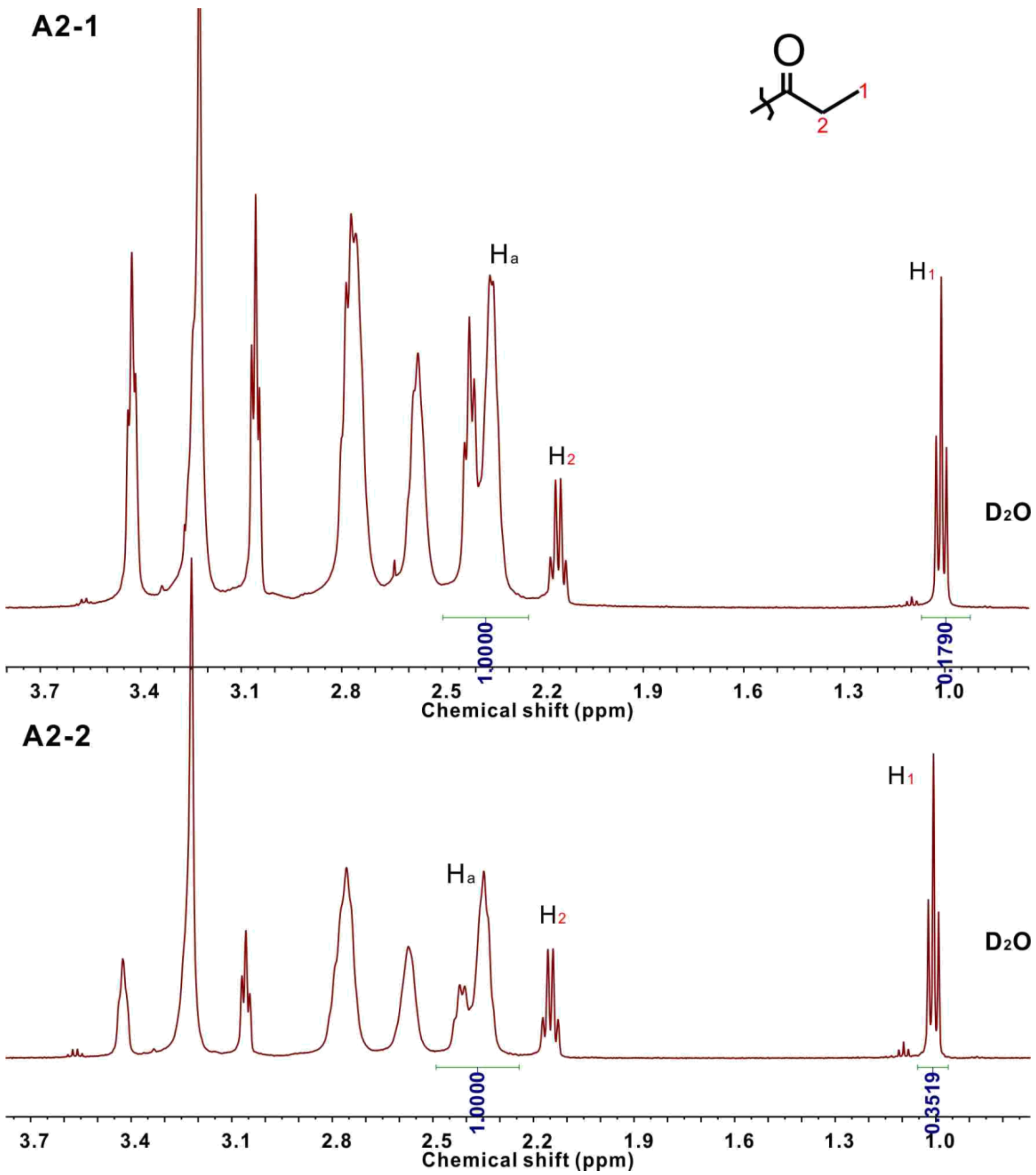
A2-1
O
1
2
Ha
H1
H2
D2O
1.0000
0.1790
3.7
3.4
3.1
2.8
2.5
2.2
1.9
1.6
1.3
1.0
Chemical shift (ppm)
A2-2
H1
Ha
H2
D2O
1.0000
0.3519
3.7
3.4
3.1
2.8
2.5
2.2
1.9
1.6
1.3
1.0
Chemical shift (ppm)

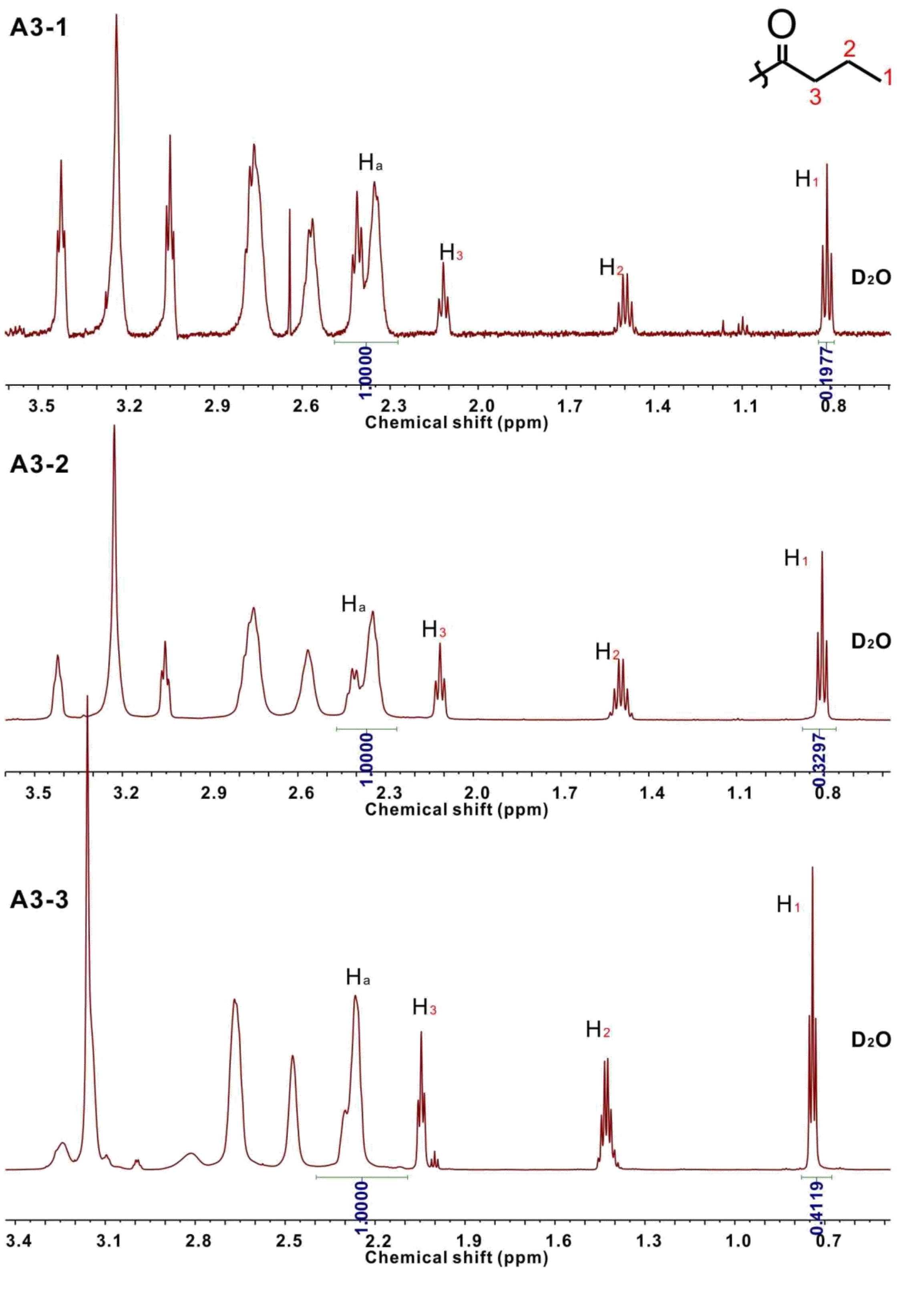
A3-1
H a
H 3
H 2
H 1
D2O
1.0000
0.1977
3.5 3.2 2.9 2.6 2.3 2.0 1.7 1.4 1.1 0.8
Chemical shift (ppm)
A3-2
H a
H 3
H 2
H 1
D2O
1.0000
0.3297
3.5 3.2 2.9 2.6 2.3 2.0 1.7 1.4 1.1 0.8
Chemical shift (ppm)
A3-3
H a
H 3
H 2
H 1
D2O
1.0000
0.4119
3.4 3.1 2.8 2.5 2.2 1.9 1.6 1.3 1.0 0.7
Chemical shift (ppm)
1
2
3

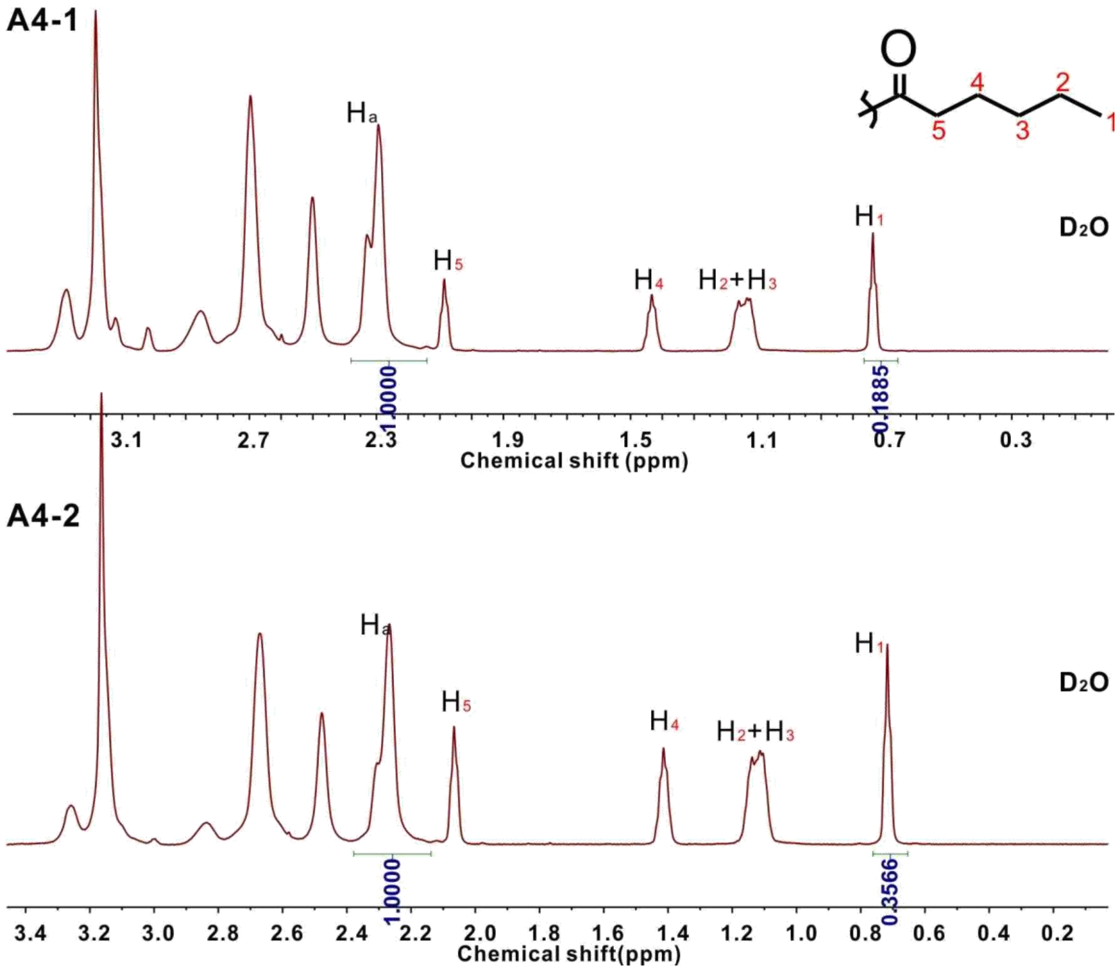
A4-1
Ha
H5
H4
H2+H3
H1
D2O
1.0000
0.1885
3.1
2.7
2.3
1.9
1.5
1.1
0.7
0.3
Chemical shift (ppm)
A4-2
Ha
H5
H4
H2+H3
H1
D2O
1.0000
0.3566
3.4
3.2
3.0
2.8
2.6
2.4
2.2
2.0
1.8
1.6
1.4
1.2
1.0
0.8
0.6
0.4
0.2
Chemical shift(ppm)

A5-1

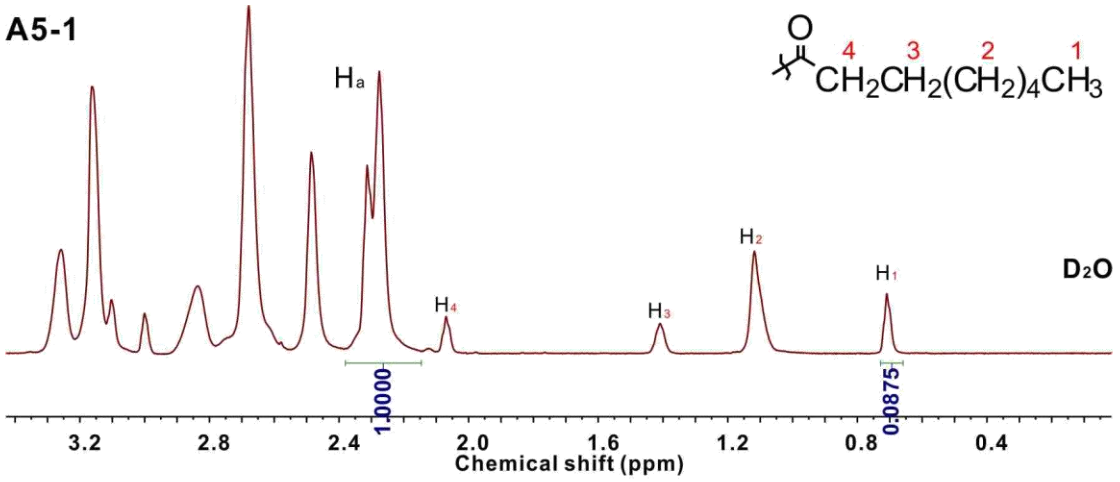
H$_a$
$CH_2CH_2(CH_2)_4CH_3$
4
3
2
1
H$_2$
H$_1$
D$_2$O
H$_4$
H$_3$
1.0000
0.0875
3.2
2.8
2.4
2.0
1.6
1.2
0.8
0.4
Chemical shift (ppm)

A5-2

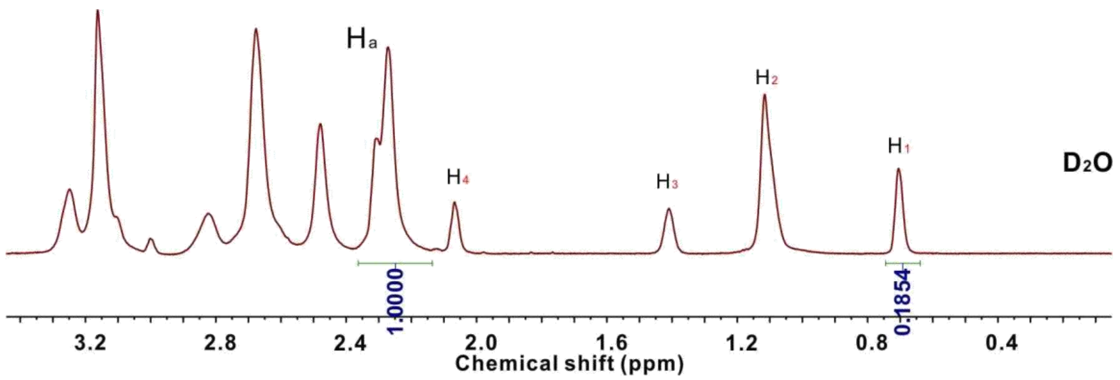
H$_a$
H$_2$
H$_1$
D$_2$O
H$_4$
H$_3$
1.0000
0.1854
3.2
2.8
2.4
2.0
1.6
1.2
0.8
0.4
Chemical shift (ppm)

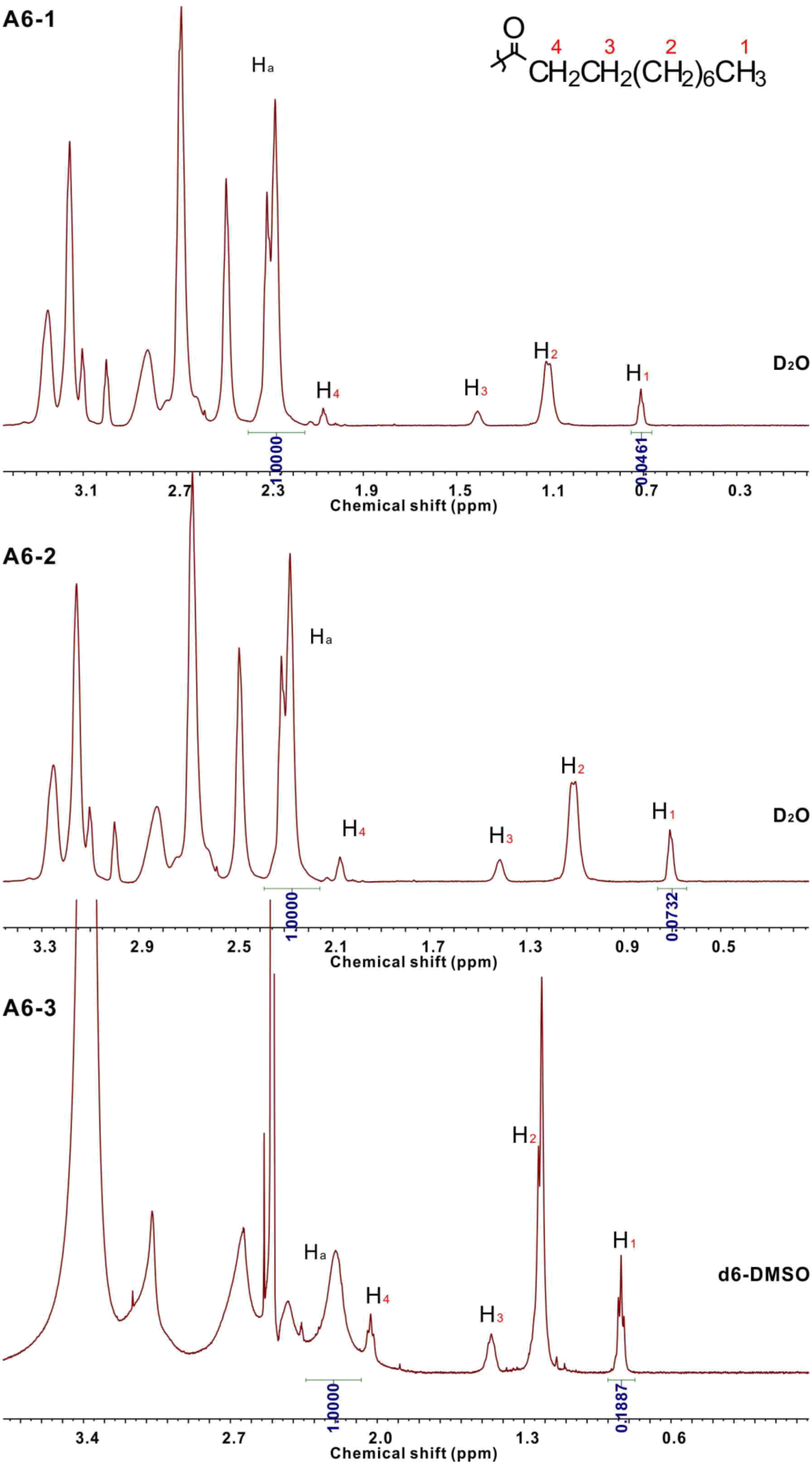
A6-1
O
4 3 2 1
CH2CH2(CH2)6CH3
Ha
H2
H1
H4
H3
D2O
1.0000
0.0461
3.1
2.7
2.3
1.9
1.5
1.1
0.7
0.3
Chemical shift (ppm)
A6-2
Ha
H2
H1
H4
H3
D2O
1.0000
0.0732
3.3
2.9
2.5
2.1
1.7
1.3
0.9
0.5
Chemical shift (ppm)
A6-3
H2
H1
Ha
H4
H3
d6-DMSO
1.0000
0.1887
3.4
2.7
2.0
1.3
0.6
Chemical shift (ppm)

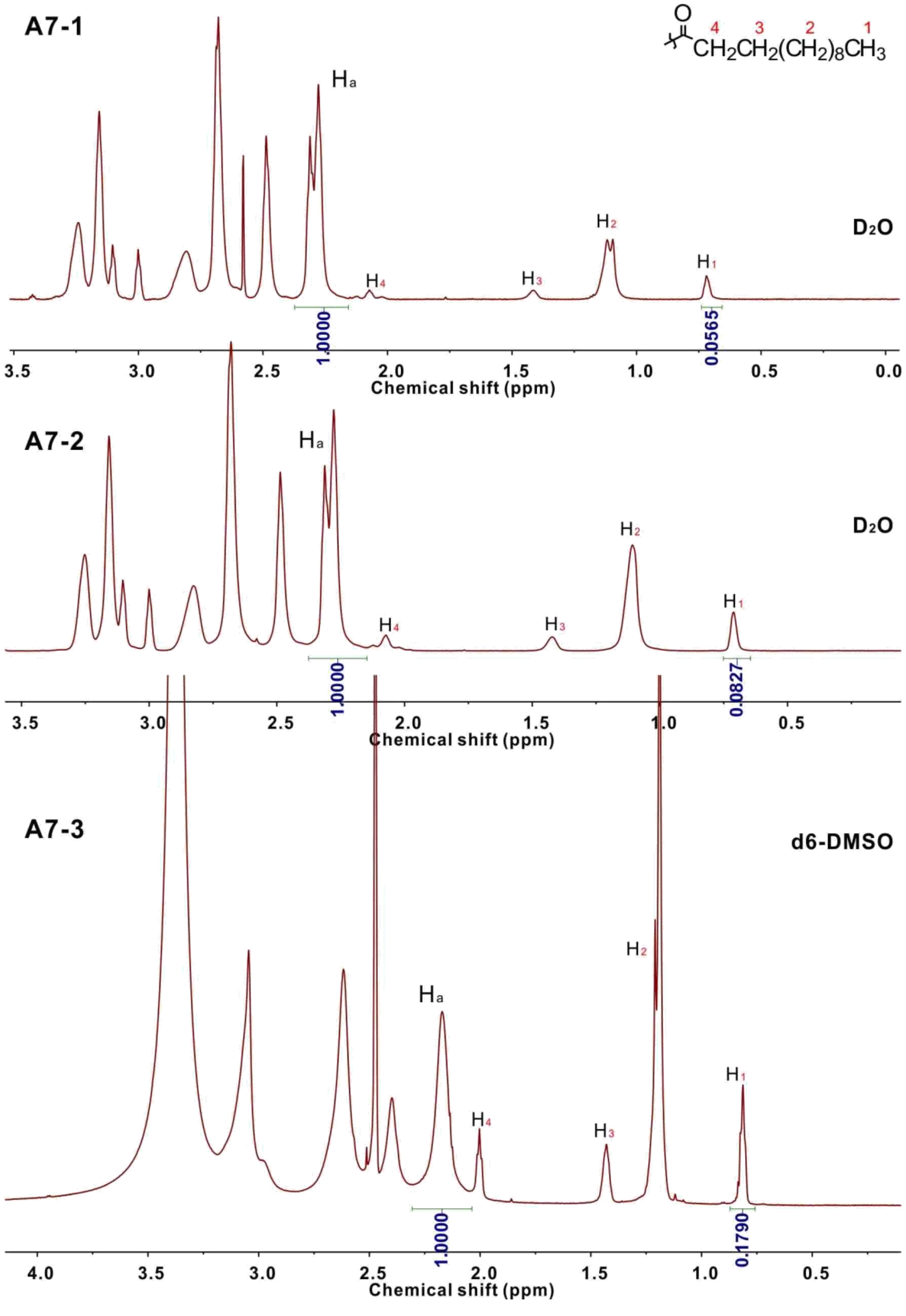
A7-1
CH2CH2(CH2)8CH3
4 3 2 1
Ha
H4
H3
H2
H1
D2O
1.0000
0.0565
Chemical shift (ppm)
A7-2
Ha
H4
H3
H2
H1
D2O
1.0000
0.0827
Chemical shift (ppm)
A7-3
d6-DMSO
Ha
H4
H3
H2
H1
1.0000
0.1790
Chemical shift (ppm)

A8-1

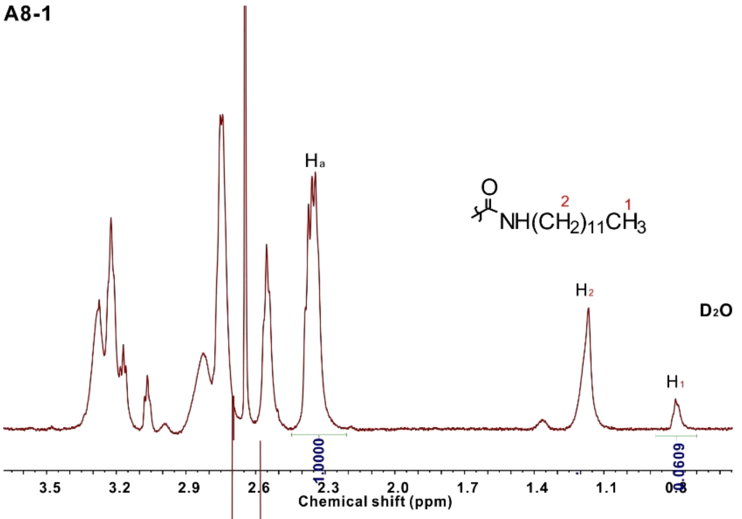

A8-2

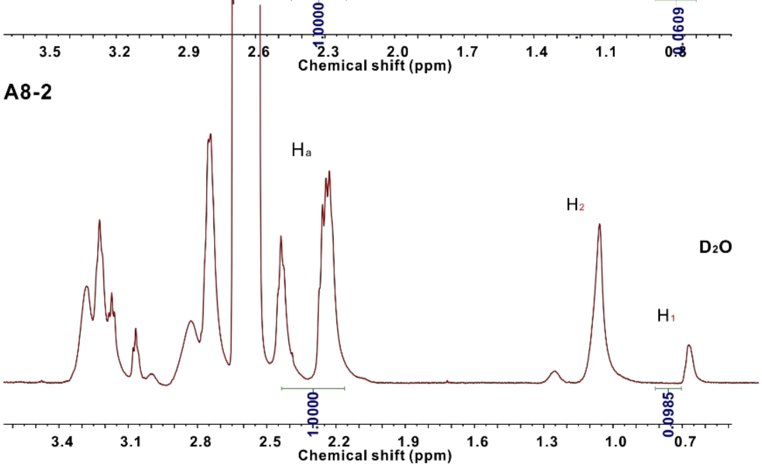

A8-3

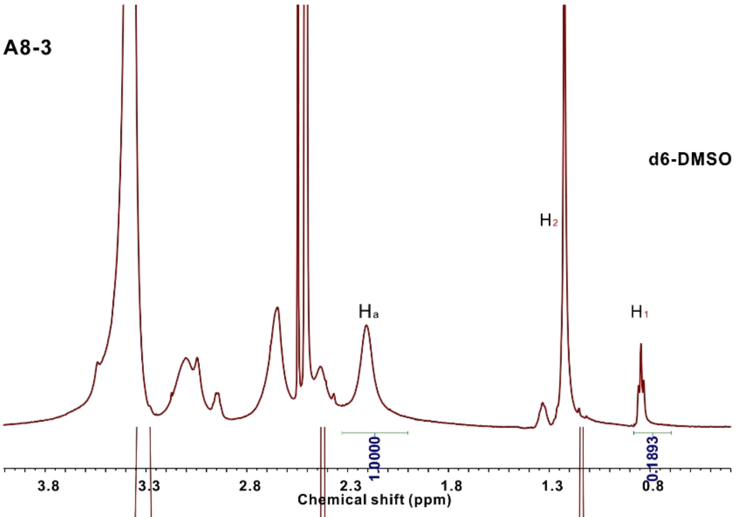

A8-4

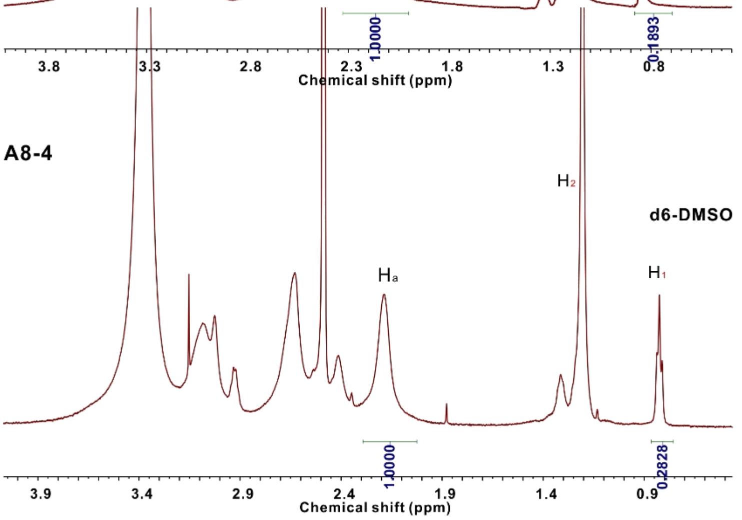

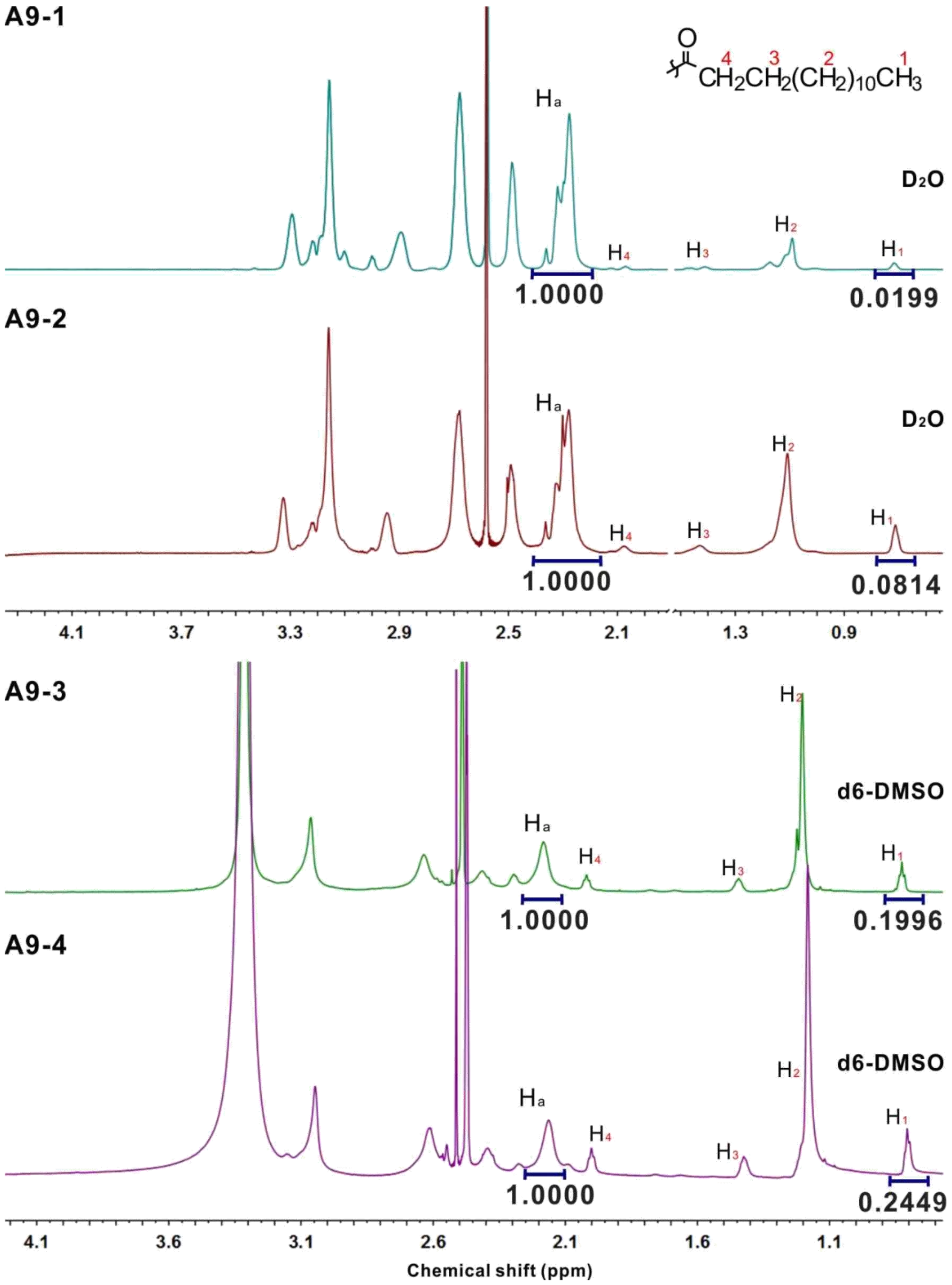
A9-1
A9-2
A9-3
A9-4
D2O
D2O
d6-DMSO
d6-DMSO
CH2CH2(CH2)10CH3
Ha
H4
H3
H2
H1
1.0000
0.0199
0.0814
0.1996
0.2449
4.1
3.7
3.3
2.9
2.5
2.1
1.3
0.9
4.1
3.6
3.1
2.6
2.1
1.6
1.1
Chemical shift (ppm)

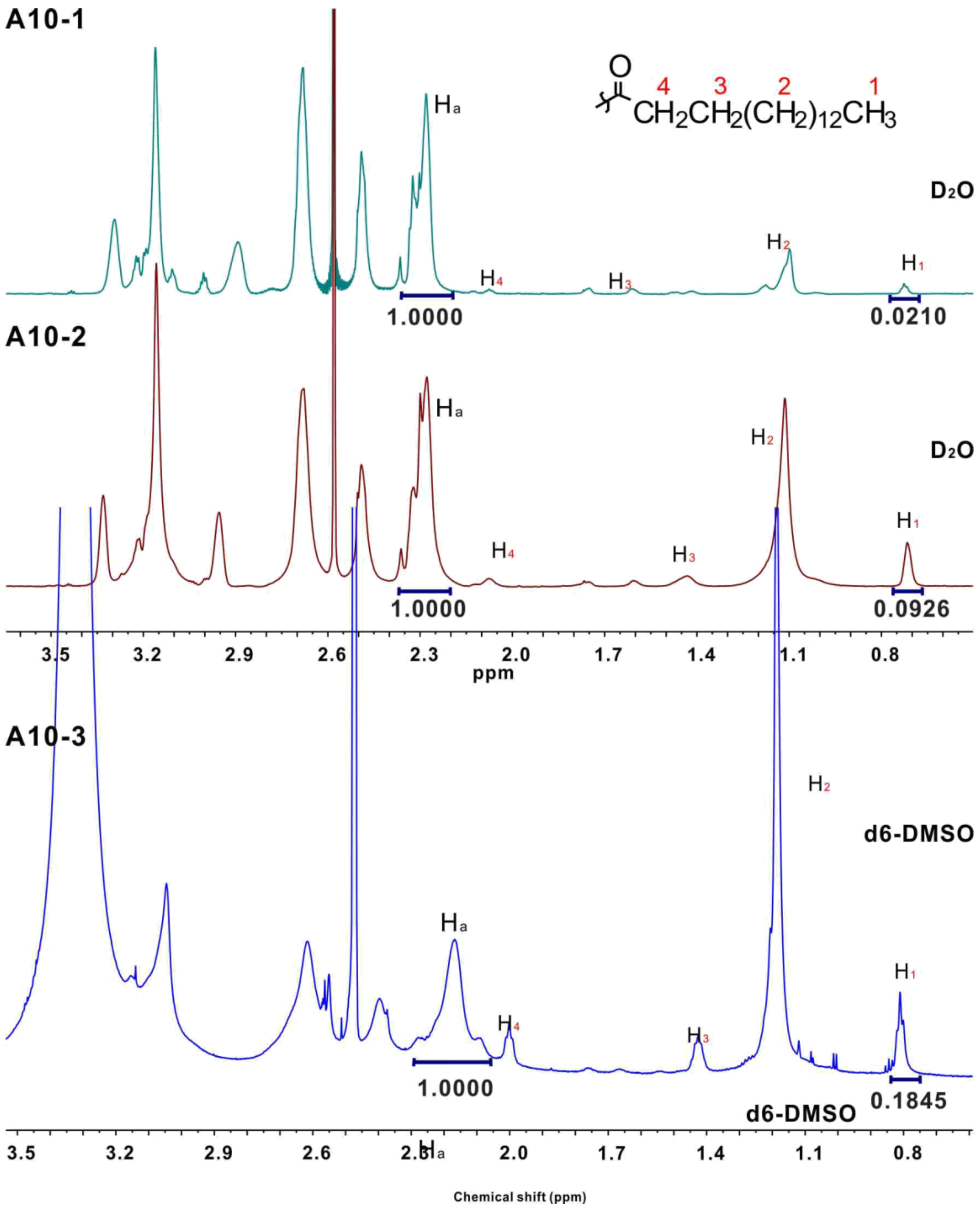
A10-1
A10-2
A10-3
$CH_2CH_2(CH_2)_{12}CH_3$
4 3 2 1
D2O
D2O
d6-DMSO
d6-DMSO
Ha
H4
H3
H2
H1
1.0000
0.0210
0.0926
0.1845
3.5 3.2 2.9 2.6 2.3 2.0 1.7 1.4 1.1 0.8
ppm
Chemical shift (ppm)

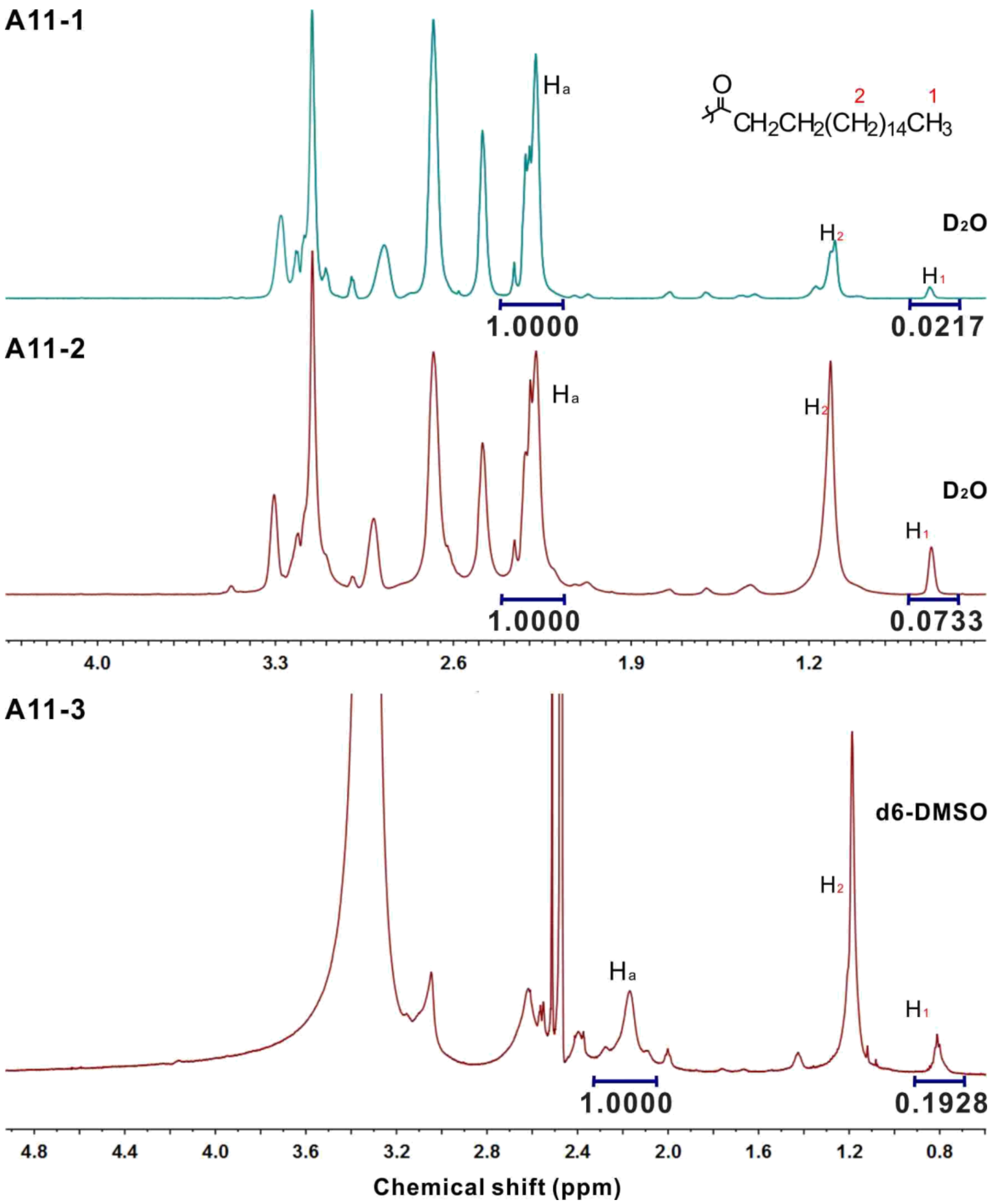
A11-1
Ha
O
CH2CH2(CH2)14CH3
2
1
D2O
H2
H1
1.0000
0.0217
A11-2
Ha
H2
D2O
H1
1.0000
0.0733
4.0
3.3
2.6
1.9
1.2
A11-3
d6-DMSO
H2
Ha
H1
1.0000
0.1928
4.8
4.4
4.0
3.6
3.2
2.8
2.4
2.0
1.6
1.2
0.8
Chemical shift (ppm)

A12-1

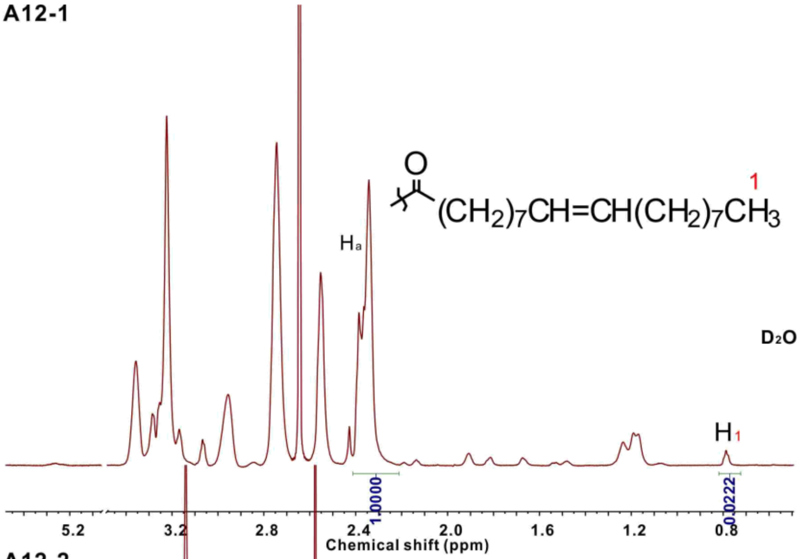

A12-2

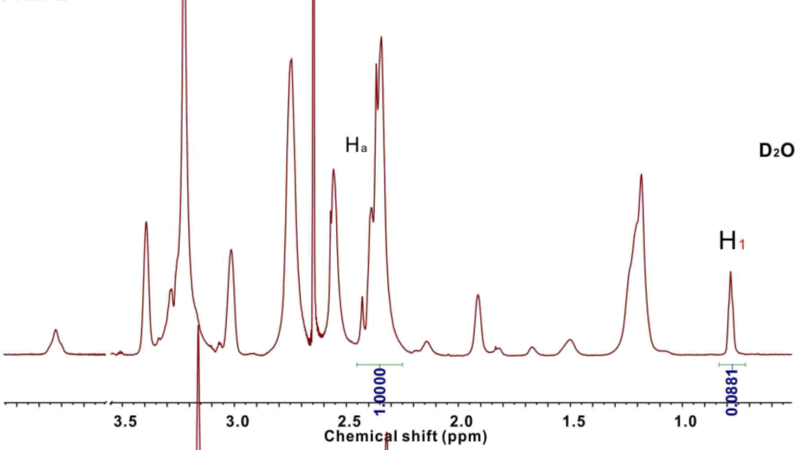

A12-3

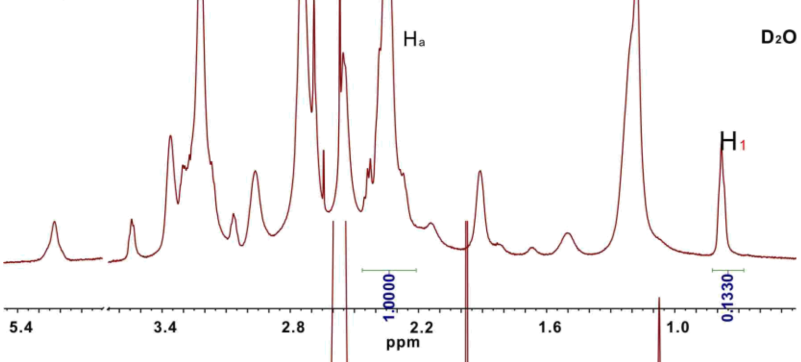

A12-4

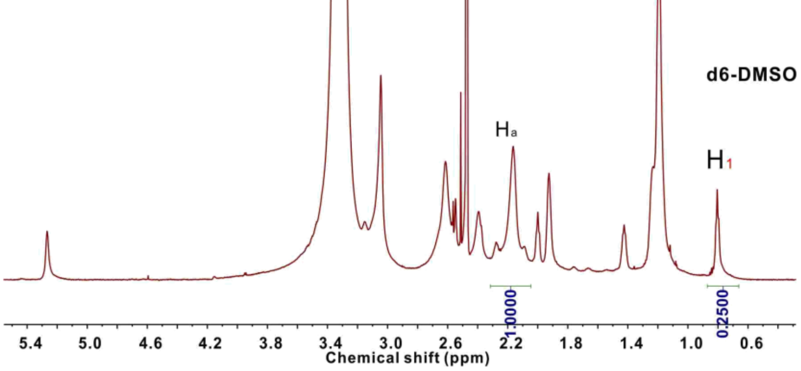

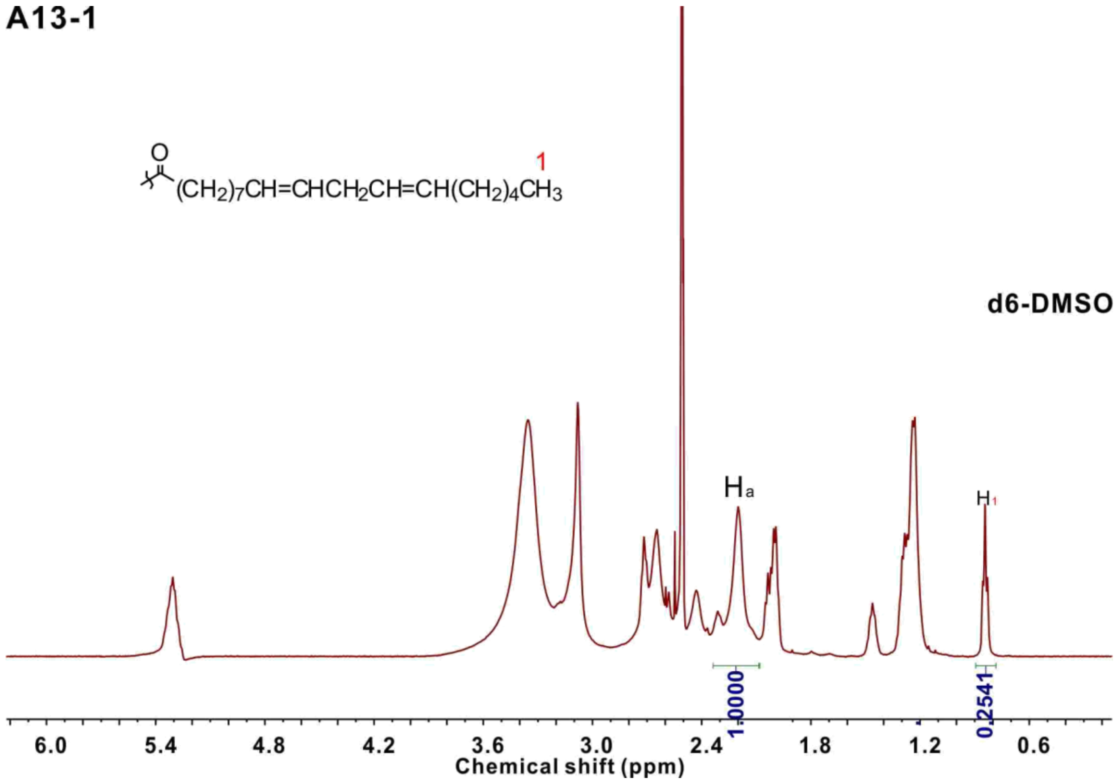
A13-1
(CH2)7CH=CHCH2CH=CH(CH2)4CH3
1
d6-DMSO
Ha
H1
1.0000
0.2541
6.0
5.4
4.8
4.2
3.6
3.0
2.4
1.8
1.2
0.6
Chemical shift (ppm)

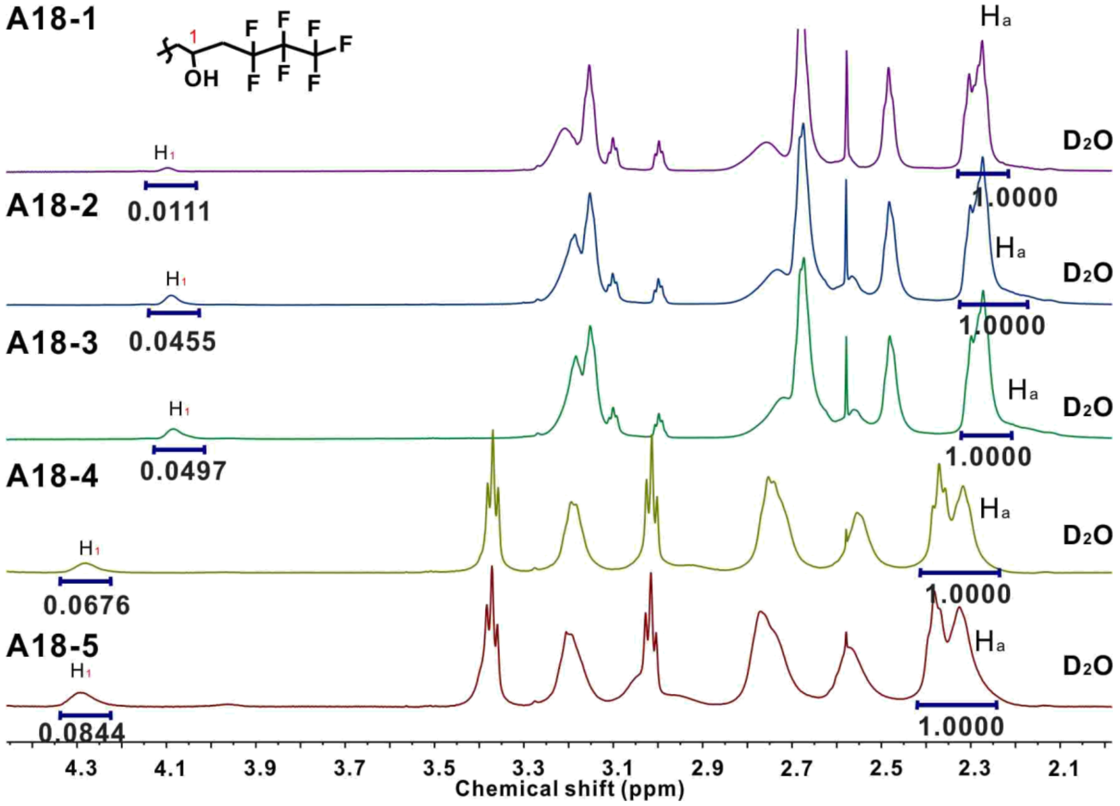
A18-1
OH
F
Ha
H1
D2O
0.0111
1.0000
A18-2
0.0455
A18-3
0.0497
A18-4
0.0676
A18-5
0.0844
4.3
4.1
3.9
3.7
3.5
3.3
3.1
2.9
2.7
2.5
2.3
2.1
Chemical shift (ppm)

B1-1

B1-2

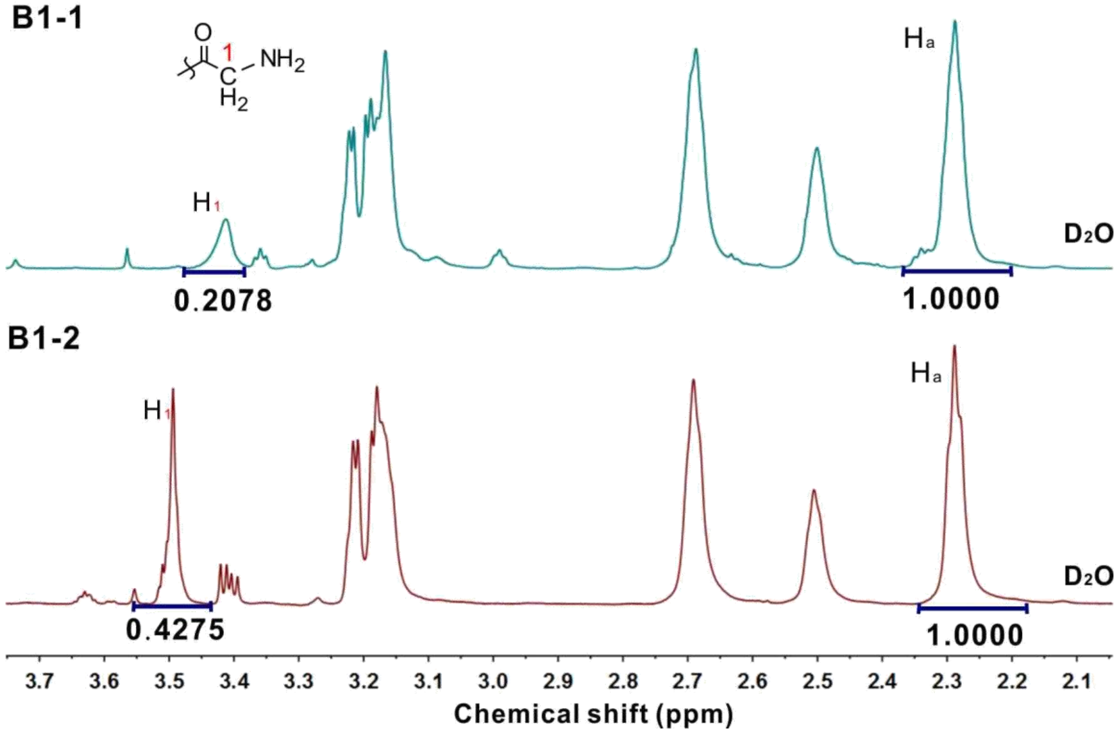

B2-1

B2-2

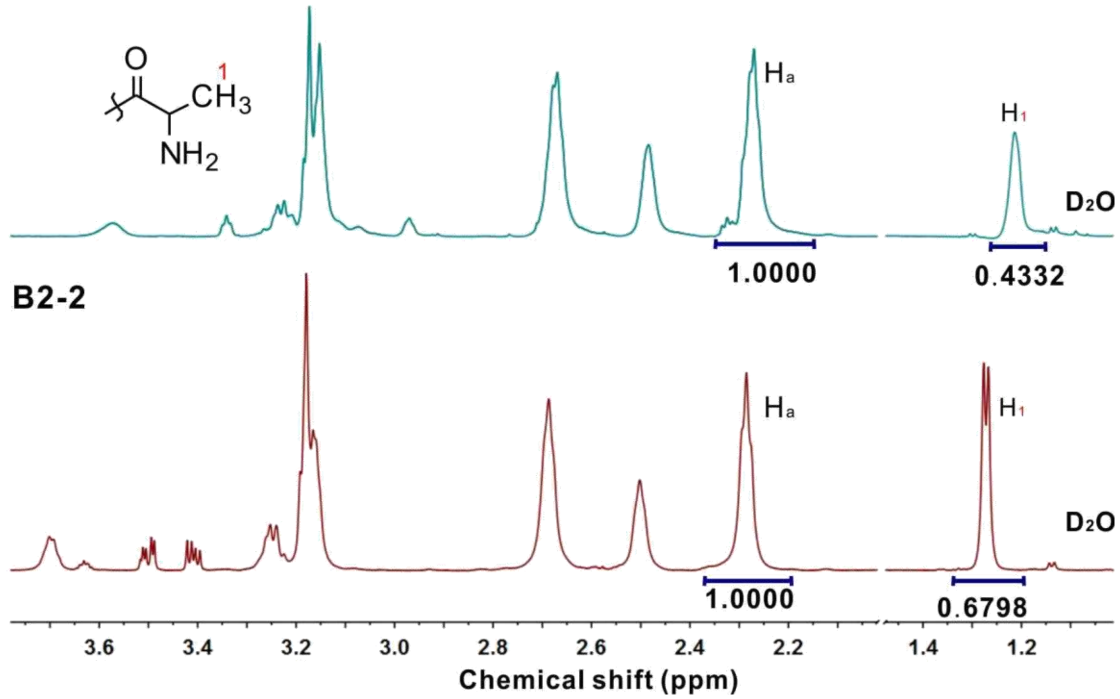

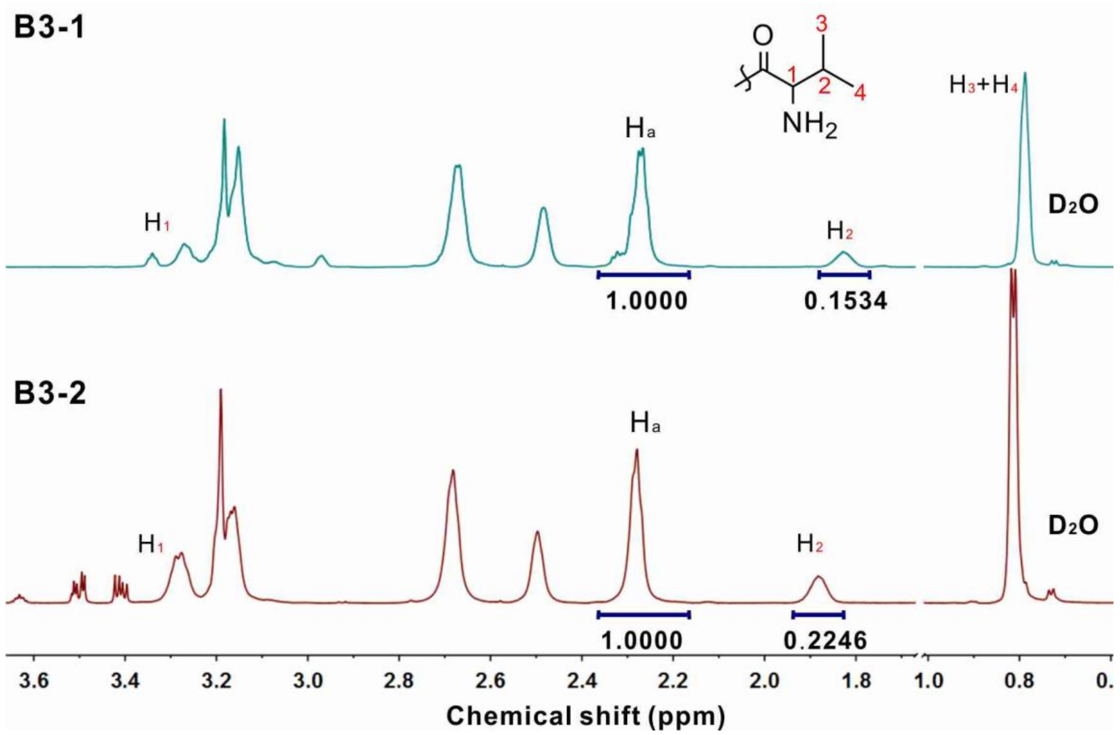
B3-1
H1
Ha
H2
H3+H4
D2O
1.0000
0.1534
B3-2
H1
Ha
H2
D2O
1.0000
0.2246
3.6 3.4 3.2 3.0 2.8 2.6 2.4 2.2 2.0 1.8 1.0 0.8 0.
Chemical shift (ppm)

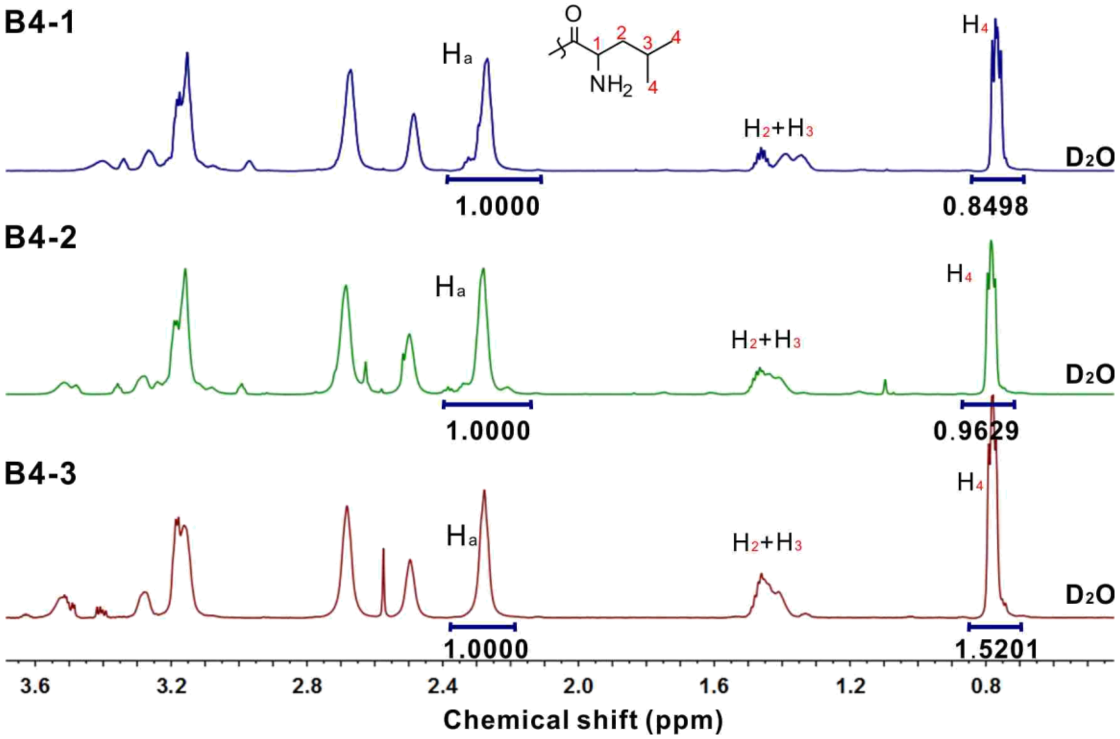
B4-1
Ha
H2+H3
H4
D2O
1.0000
0.8498
B4-2
Ha
H2+H3
H4
D2O
1.0000
0.9629
B4-3
Ha
H2+H3
H4
D2O
1.0000
1.5201
3.6 3.2 2.8 2.4 2.0 1.6 1.2 0.8
Chemical shift (ppm)

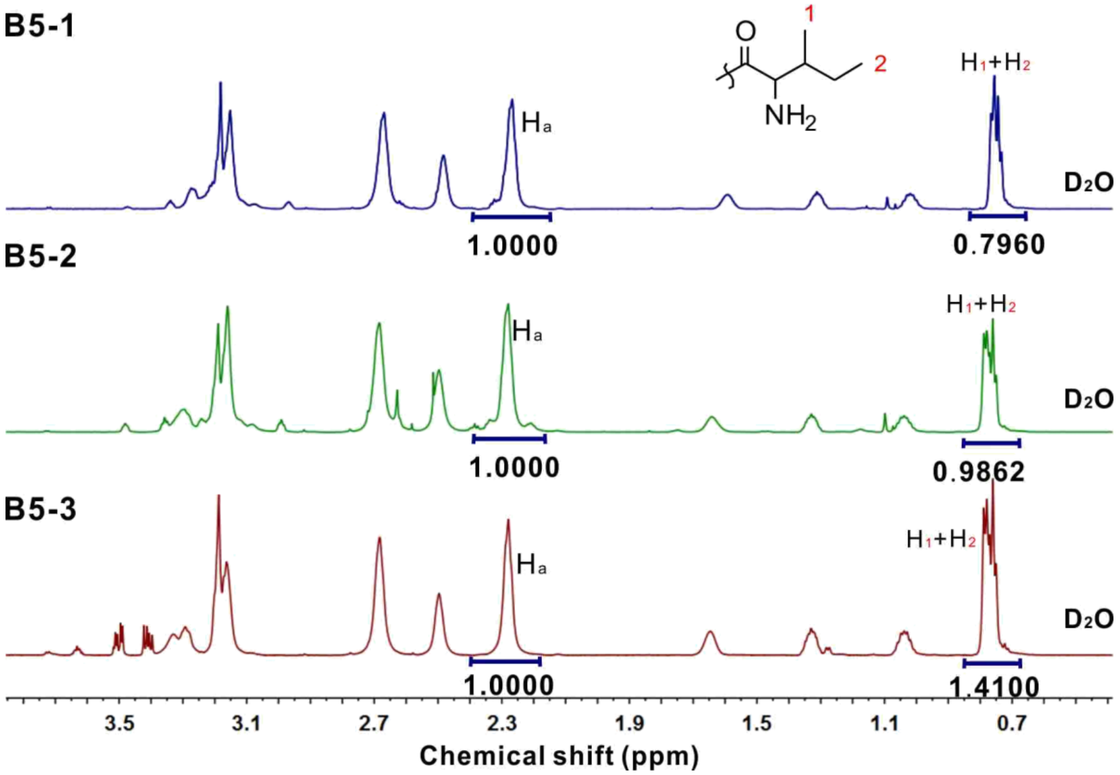
B5-1
B5-2
B5-3
O
NH2
1
2
Ha
H1+H2
D2O
1.0000
0.7960
0.9862
1.4100
3.5
3.1
2.7
2.3
1.9
1.5
1.1
0.7
Chemical shift (ppm)

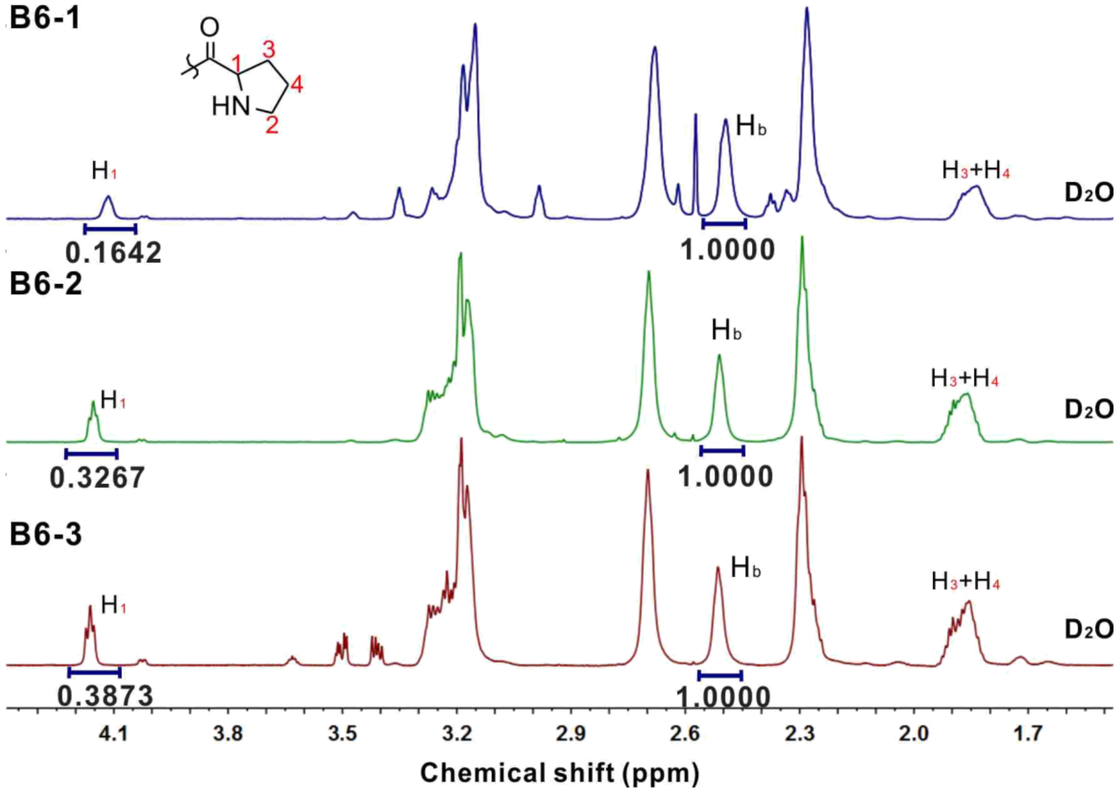
B6-1
B6-2
B6-3
O
HN
1
2
3
4
H1
Hb
H3+H4
D2O
0.1642
0.3267
0.3873
1.0000
4.1
3.8
3.5
3.2
2.9
2.6
2.3
2.0
1.7
Chemical shift (ppm)

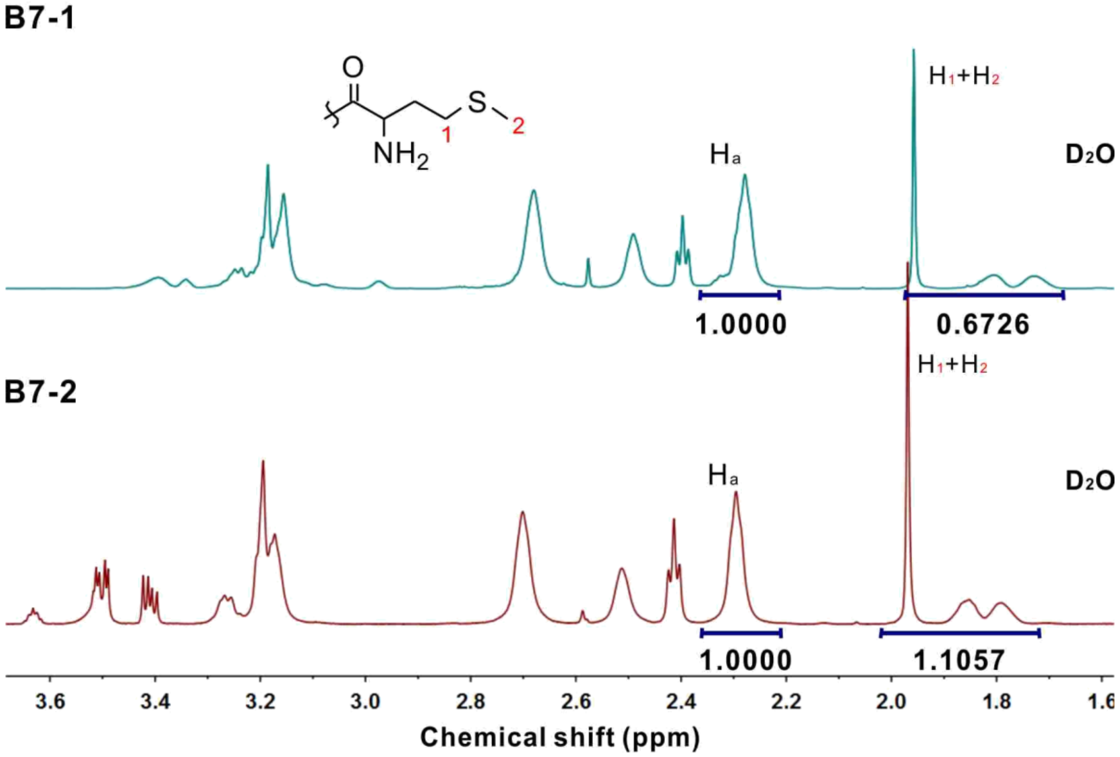

B7-1
O
S
1
2
NH2
Ha
H1+H2
D2O
1.0000
0.6726
B7-2
H1+H2
Ha
D2O
1.0000
1.1057
3.6
3.4
3.2
3.0
2.8
2.6
2.4
2.2
2.0
1.8
1.6
Chemical shift (ppm)

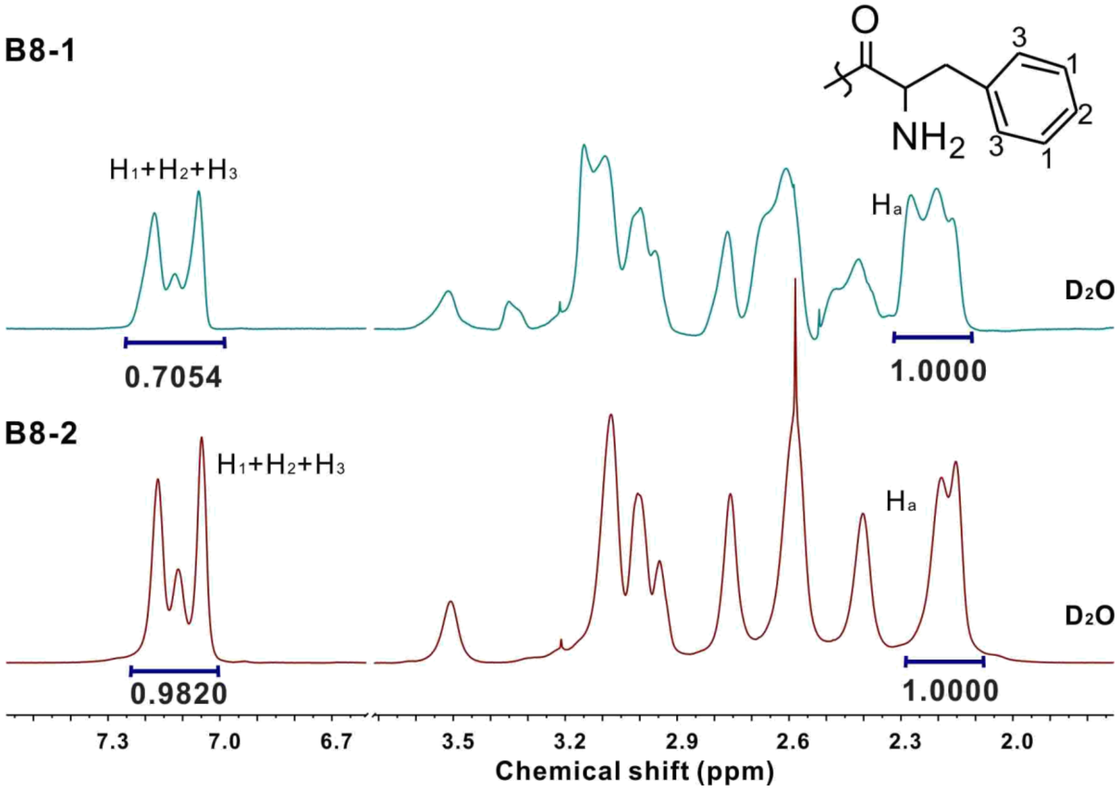

B8-1
O
3
1
2
NH2
3
1
H1+H2+H3
Ha
D2O
0.7054
1.0000
B8-2
H1+H2+H3
Ha
D2O
0.9820
1.0000
7.3
7.0
6.7
3.5
3.2
2.9
2.6
2.3
2.0
Chemical shift (ppm)

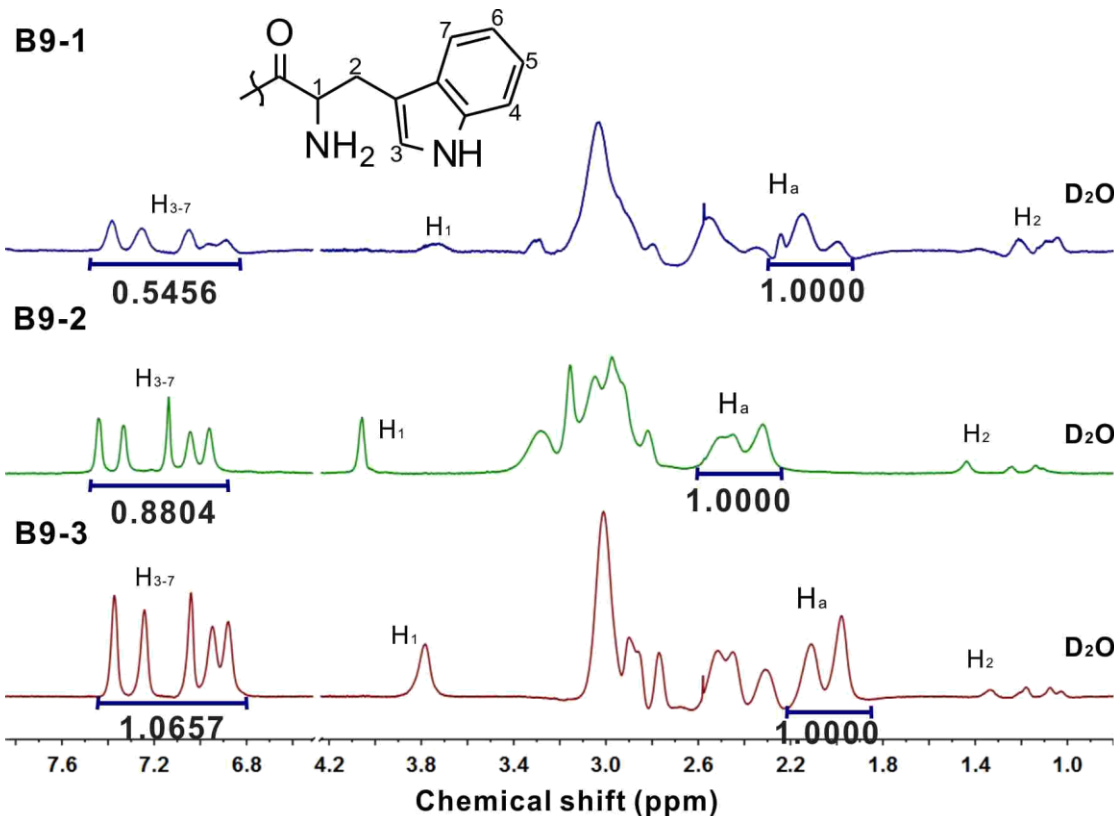
B9-1
B9-2
B9-3
H3-7
H1
Ha
H2
D2O
0.5456
1.0000
0.8804
1.0000
1.0657
1.0000
7.6
7.2
6.8
4.2
3.8
3.4
3.0
2.6
2.2
1.8
1.4
1.0
Chemical shift (ppm)

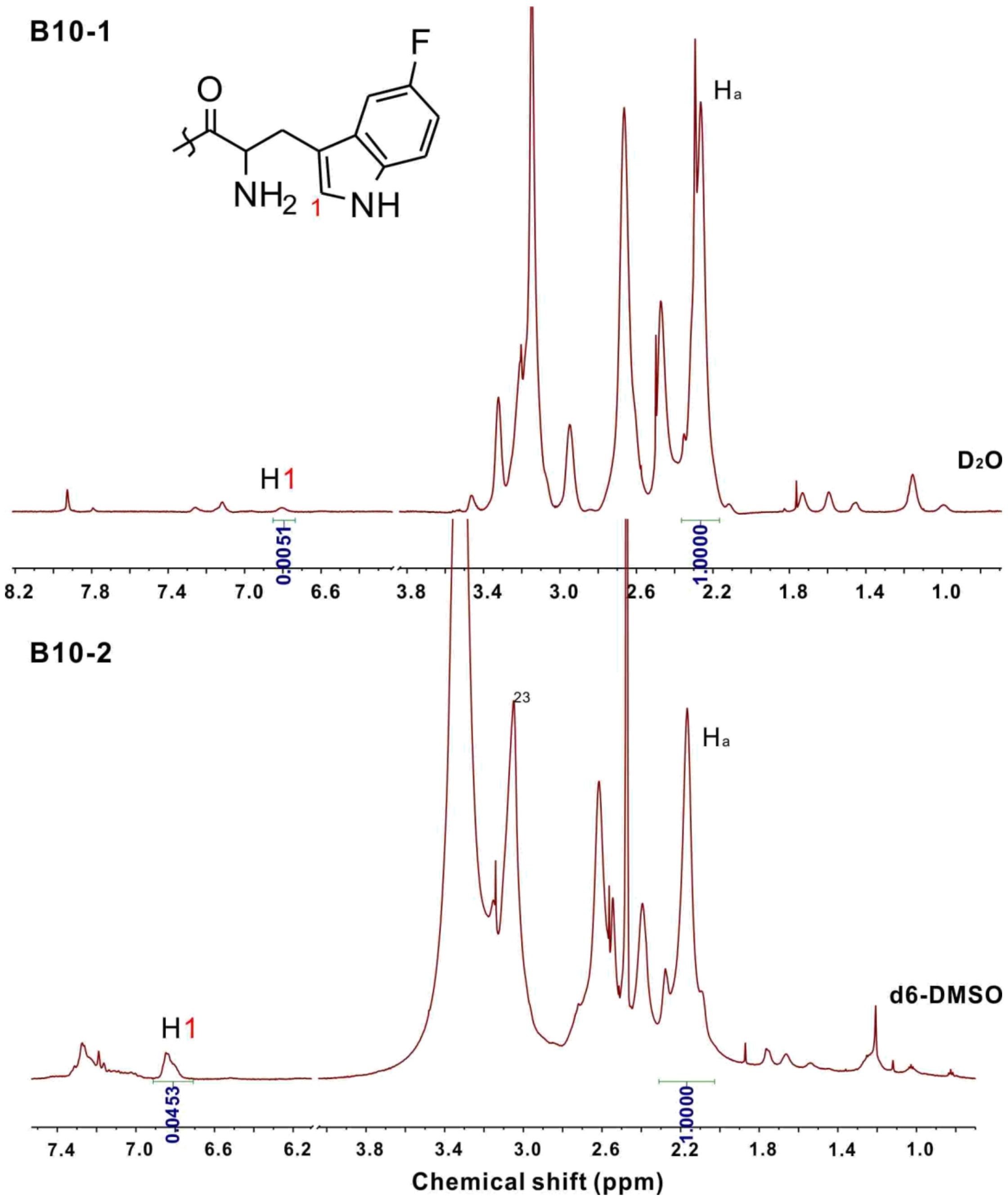
B10-1
F
O
NH2
1
NH
Ha
H1
D2O
0.0051
1.0000
8.2 7.8 7.4 7.0 6.6 3.8 3.4 3.0 2.6 2.2 1.8 1.4 1.0
B10-2
23
Ha
d6-DMSO
H1
0.0453
1.0000
7.4 7.0 6.6 6.2 3.8 3.4 3.0 2.6 2.2 1.8 1.4 1.0
Chemical shift (ppm)

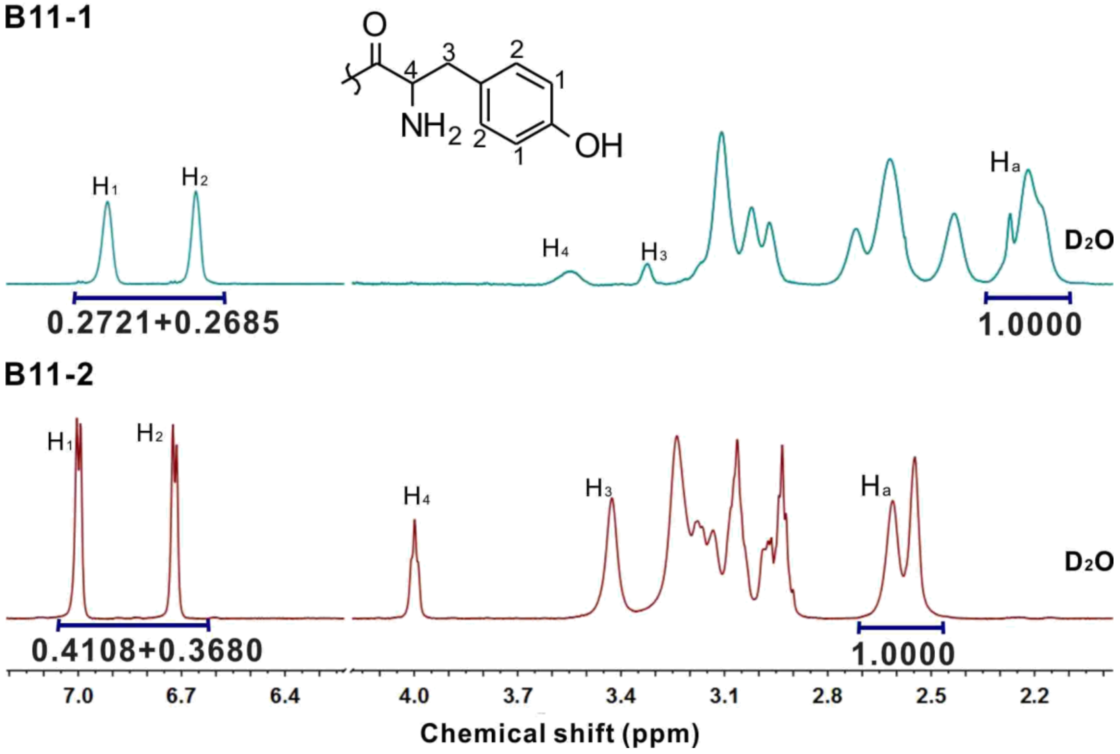
B11-1
O
3
2
4
1
NH2
2
1
OH
H1
H2
H4
H3
Ha
D2O
0.2721+0.2685
1.0000
B11-2
H1
H2
H4
H3
Ha
D2O
0.4108+0.3680
1.0000
7.0
6.7
6.4
4.0
3.7
3.4
3.1
2.8
2.5
2.2
Chemical shift (ppm)

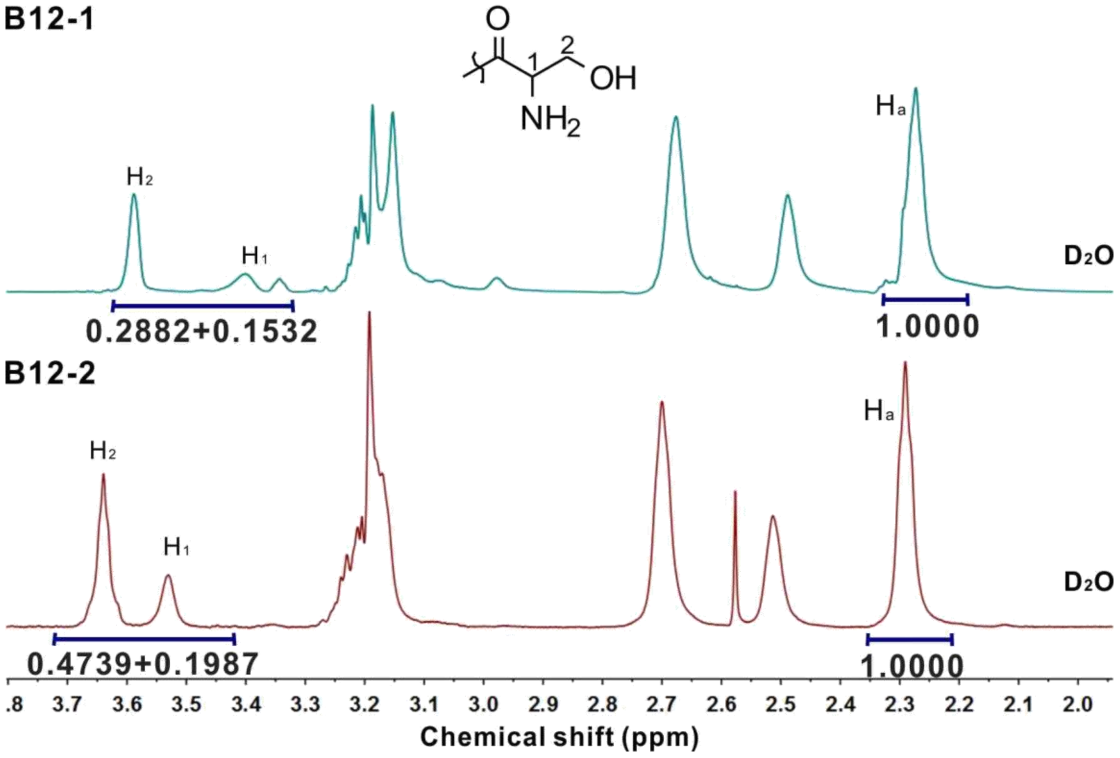
B12-1
O
2
1
OH
NH2
H2
H1
Ha
D2O
0.2882+0.1532
1.0000
B12-2
H2
H1
Ha
D2O
0.4739+0.1987
1.0000
.8
3.7
3.6
3.5
3.4
3.3
3.2
3.1
3.0
2.9
2.8
2.7
2.6
2.5
2.4
2.3
2.2
2.1
2.0
Chemical shift (ppm)

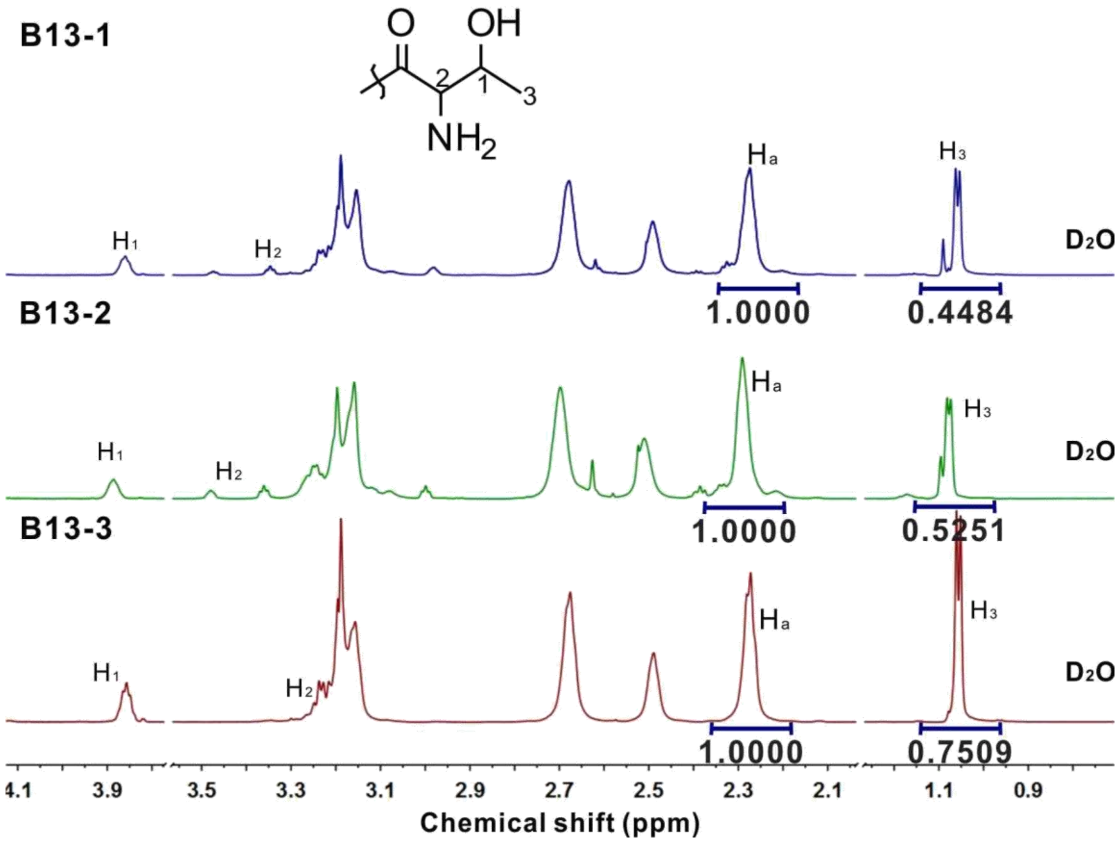
B13-1
B13-2
B13-3
OH
O
NH2
H1
H2
Ha
H3
D2O
1.0000
0.4484
0.5251
0.7509
4.1
3.9
3.5
3.3
3.1
2.9
2.7
2.5
2.3
2.1
1.1
0.9
Chemical shift (ppm)

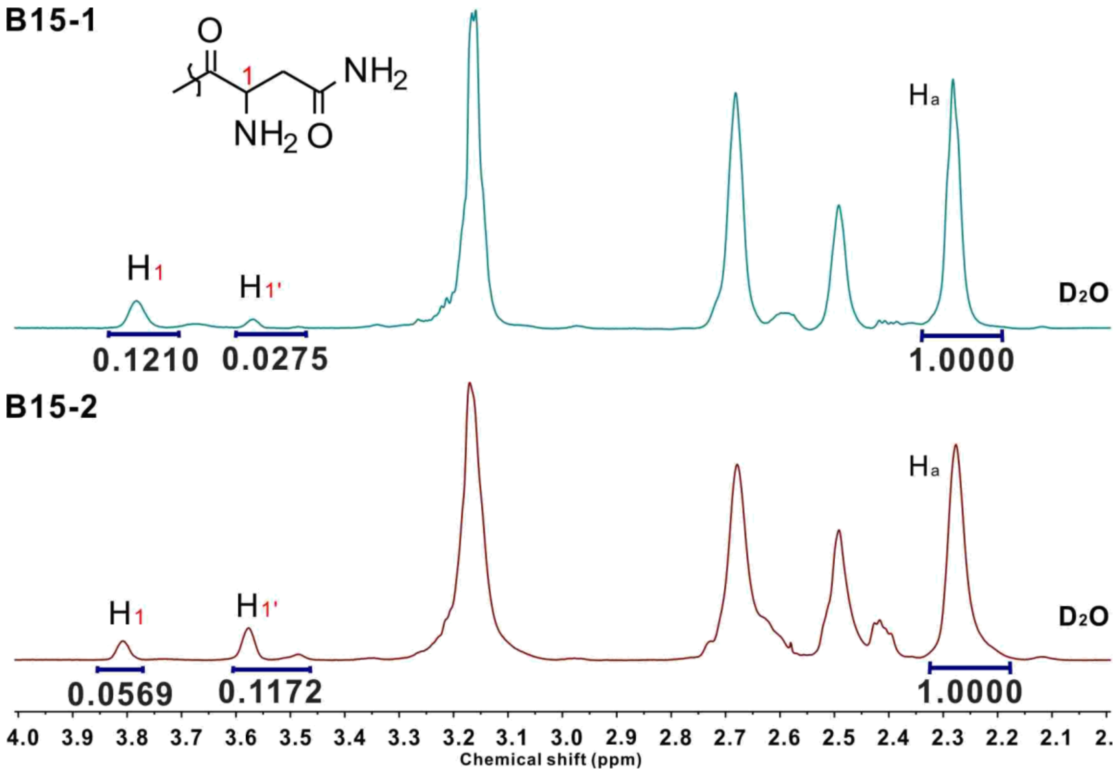
B15-1
B15-2
O
NH2
NH2 O
H1
H1'
Ha
D2O
0.1210
0.0275
1.0000
0.0569
0.1172
4.0
3.9
3.8
3.7
3.6
3.5
3.4
3.3
3.2
3.1
3.0
2.9
2.8
2.7
2.6
2.5
2.4
2.3
2.2
2.1
Chemical shift (ppm)

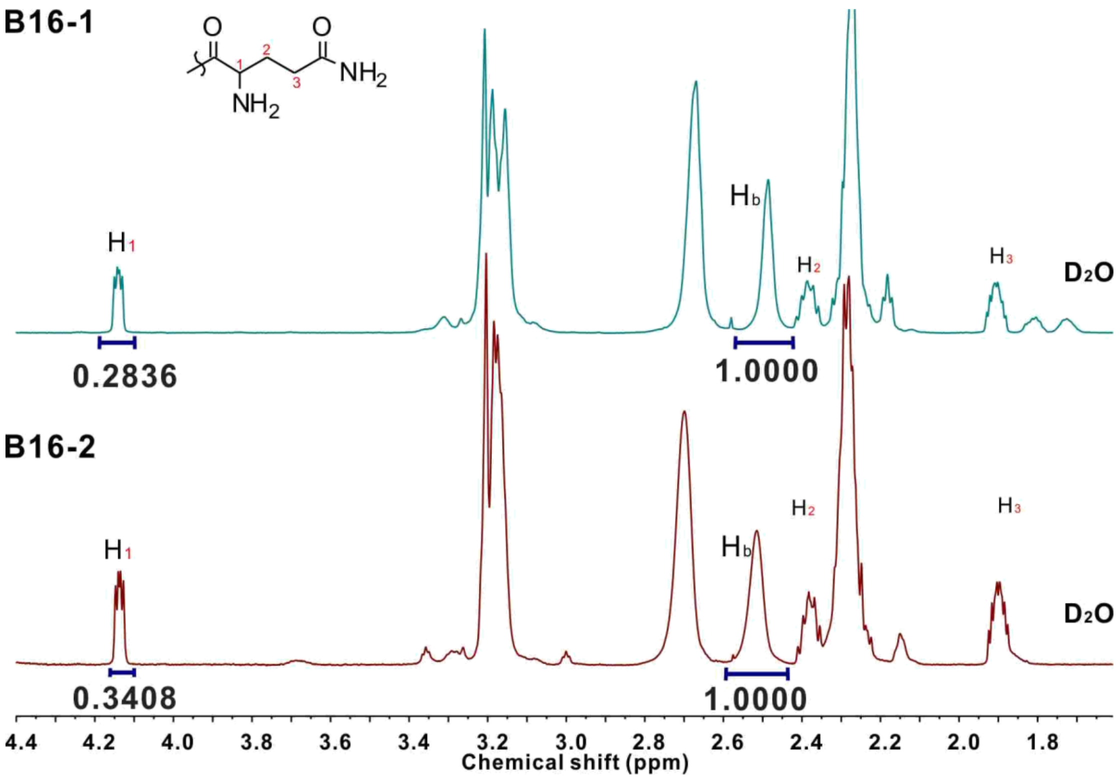
B16-1
B16-2
H1
Hb
H2
H3
D2O
0.2836
1.0000
0.3408
1.0000
4.4 4.2 4.0 3.8 3.6 3.4 3.2 3.0 2.8 2.6 2.4 2.2 2.0 1.8
Chemical shift (ppm)

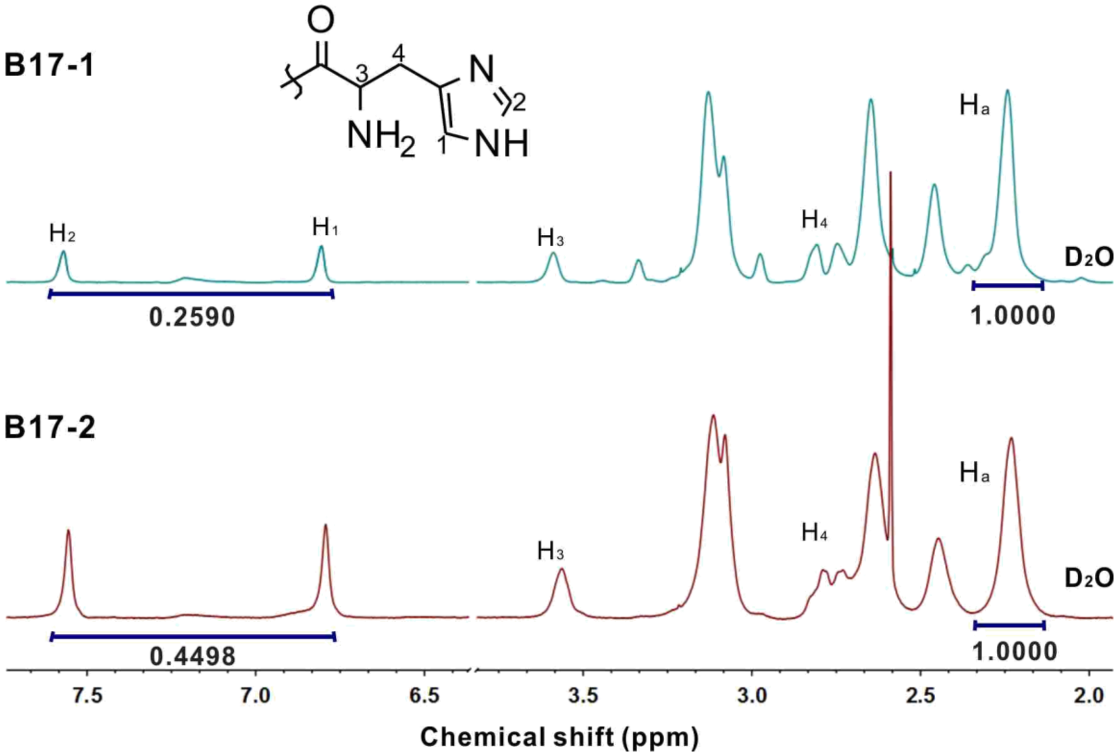
B17-1
B17-2
H2
H1
H3
H4
Ha
D2O
0.2590
1.0000
0.4498
1.0000
7.5 7.0 6.5 3.5 3.0 2.5 2.0
Chemical shift (ppm)

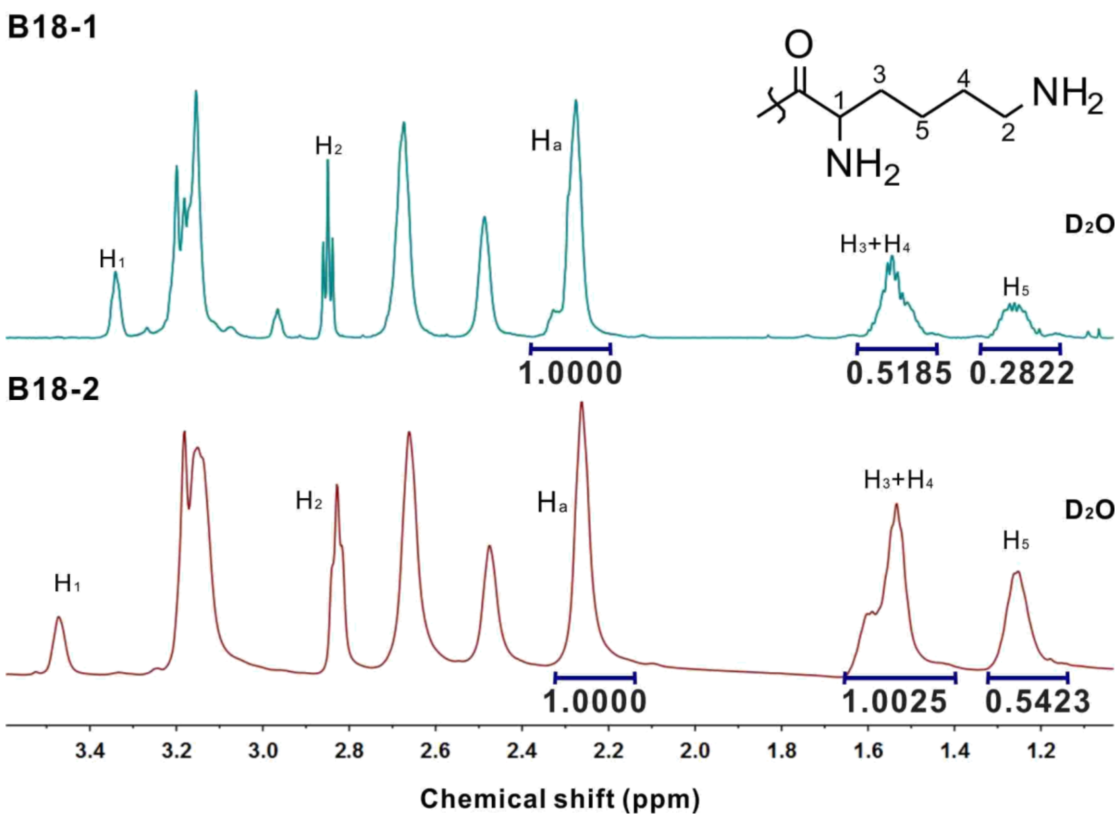

B18-1
B18-2
NH2
NH2
D2O
H1
H2
Ha
H3+H4
H5
1.0000
0.5185
0.2822
1.0025
0.5423
3.4
3.2
3.0
2.8
2.6
2.4
2.2
2.0
1.8
1.6
1.4
1.2
Chemical shift (ppm)

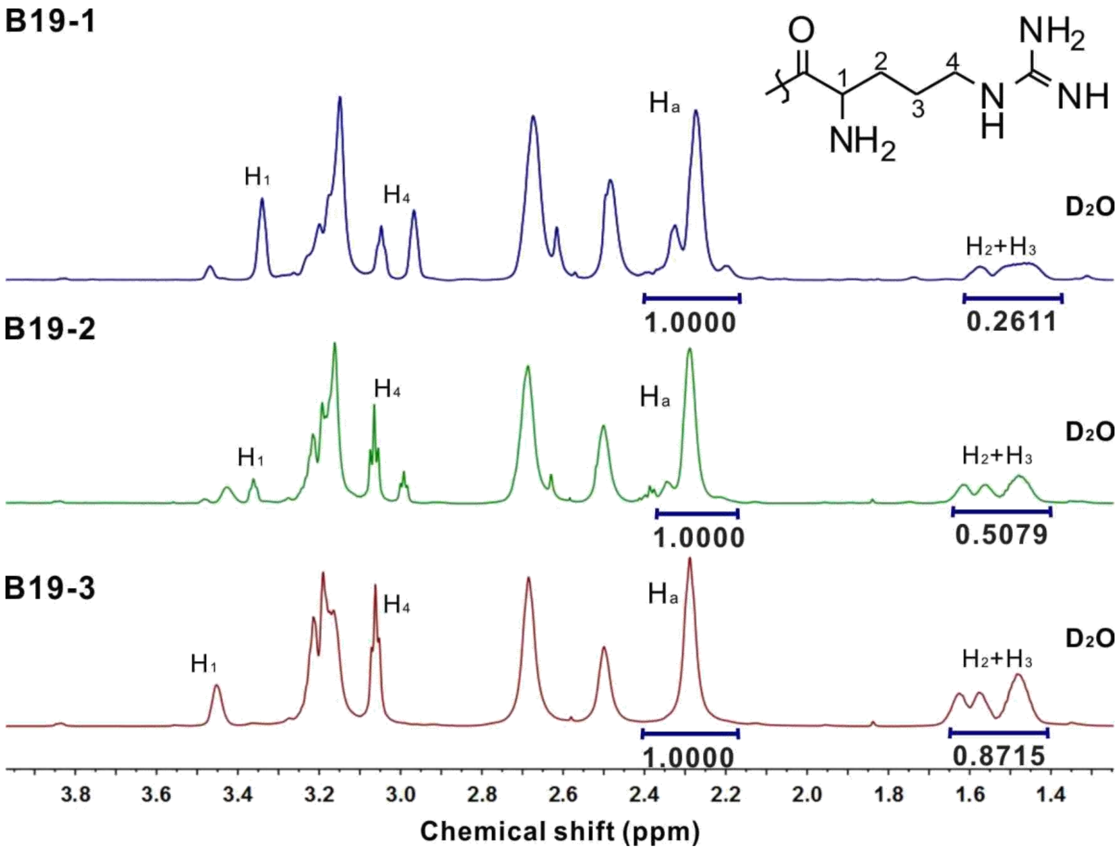

B19-1
B19-2
B19-3
NH2
NH
D2O
H1
H4
Ha
H2+H3
1.0000
0.2611
0.5079
0.8715
3.8
3.6
3.4
3.2
3.0
2.8
2.6
2.4
2.2
2.0
1.8
1.6
1.4
Chemical shift (ppm)

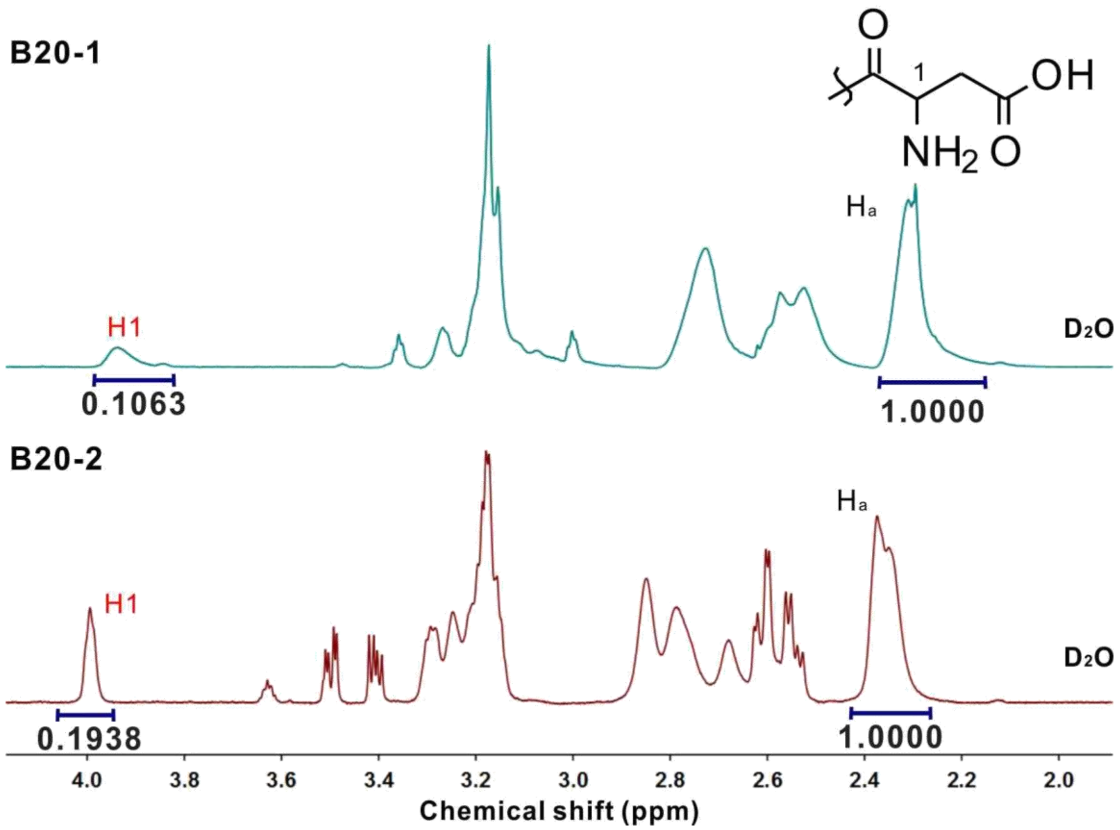
B20-1
O
1
OH
NH_2 O
Ha
H1
D_2O
0.1063
1.0000
B20-2
Ha
H1
D_2O
0.1938
1.0000
4.0
3.8
3.6
3.4
3.2
3.0
2.8
2.6
2.4
2.2
2.0
Chemical shift (ppm)

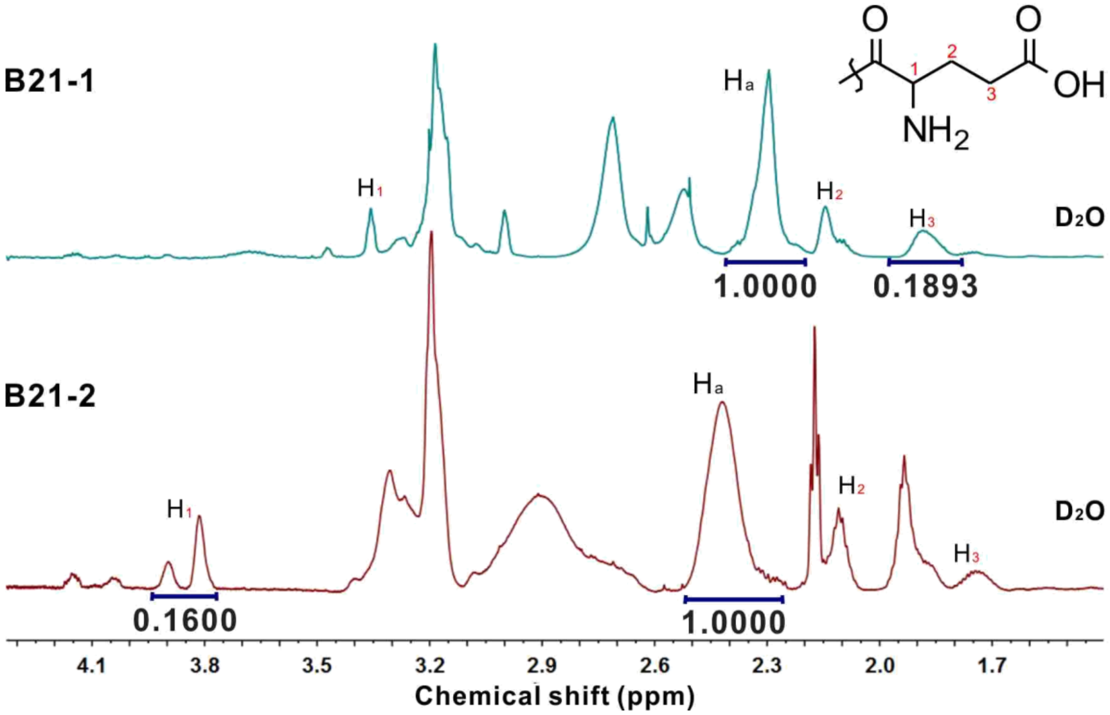
B21-1
O
O
1
2
3
OH
NH_2
Ha
H1
H2
H3
D_2O
1.0000
0.1893
B21-2
Ha
H1
H2
H3
D_2O
0.1600
1.0000
4.1
3.8
3.5
3.2
2.9
2.6
2.3
2.0
1.7
Chemical shift (ppm)

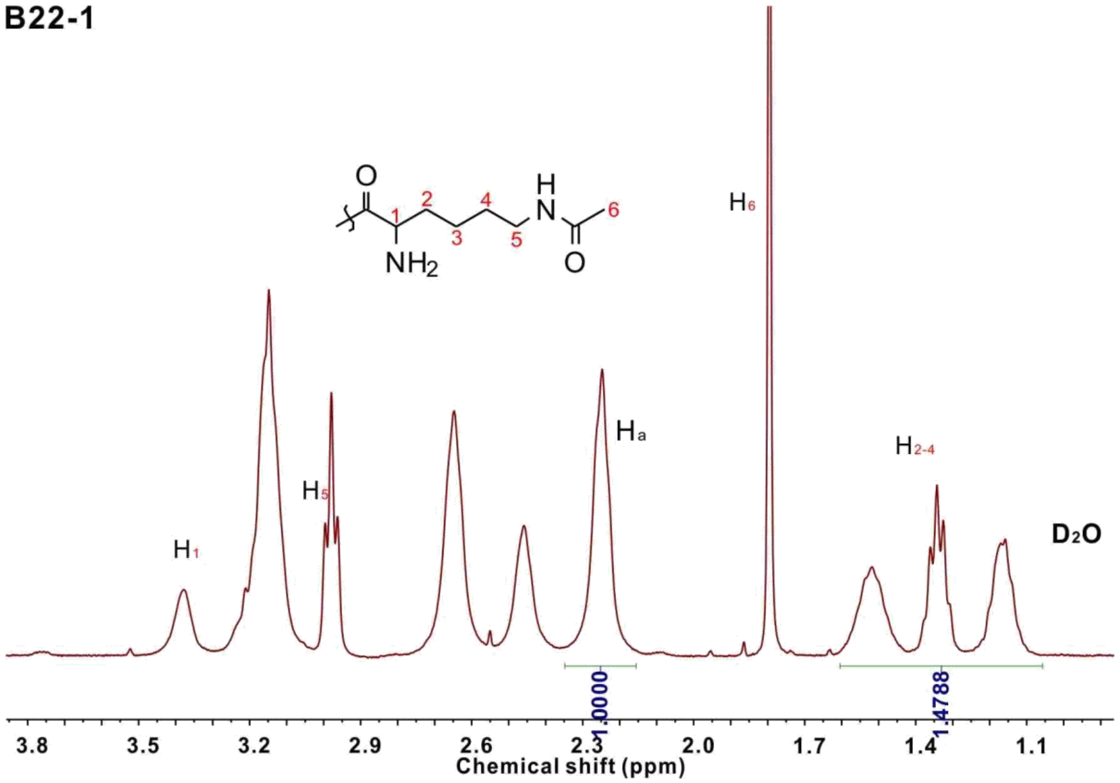
B22-1
H6
Ha
H5
H1
H2-4
D2O
1.0000
1.4788
3.8
3.5
3.2
2.9
2.6
2.3
2.0
1.7
1.4
1.1
Chemical shift (ppm)

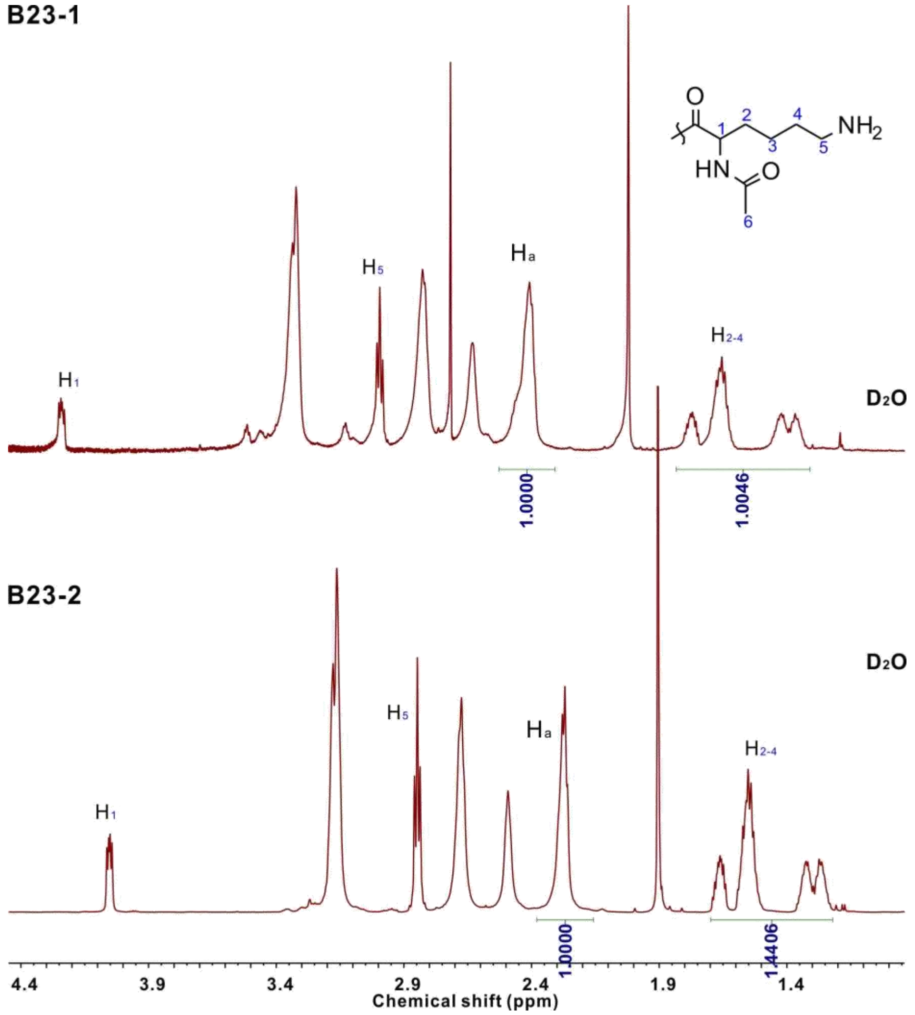
B23-1
H1
H5
Ha
H2-4
D2O
1.0000
1.0046
B23-2
H1
H5
Ha
H2-4
D2O
1.0000
1.4406
4.4
3.9
3.4
2.9
2.4
1.9
1.4
Chemical shift (ppm)

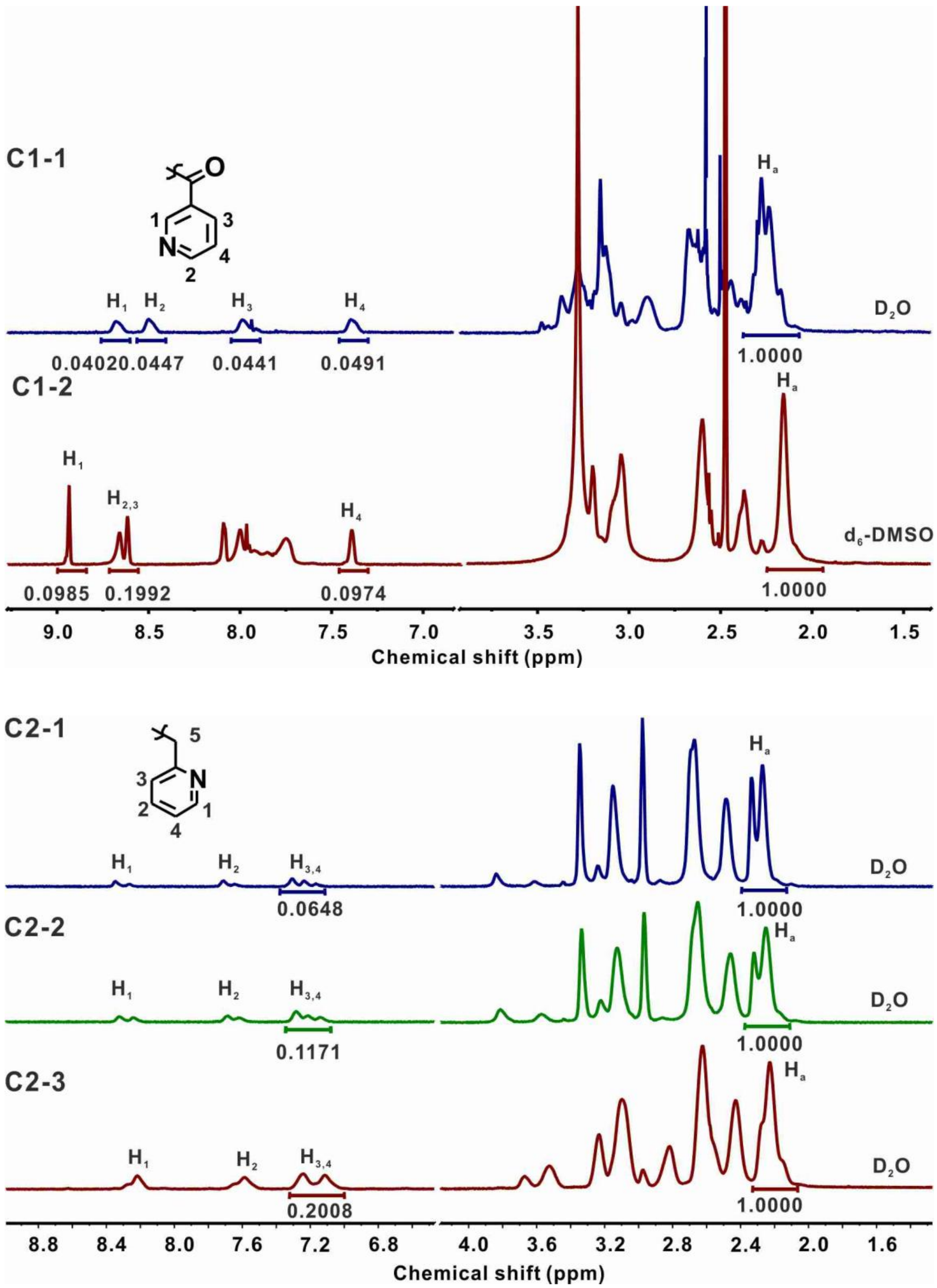
C1-1
1
3
N
4
2
H1 H2 H3 H4
Ha
D2O
0.04020.0447 0.0441 0.0491
1.0000
C1-2
H1
H2,3
H4
Ha
d6-DMSO
0.0985 0.1992 0.0974
1.0000
9.0 8.5 8.0 7.5 7.0 3.5 3.0 2.5 2.0 1.5
Chemical shift (ppm)
C2-1
5
3
N
2
1
4
H1 H2 H3,4
Ha
D2O
0.0648
1.0000
C2-2
H1 H2 H3,4
Ha
D2O
0.1171
1.0000
C2-3
H1 H2 H3,4
Ha
D2O
0.2008
1.0000
8.8 8.4 8.0 7.6 7.2 6.8 4.0 3.6 3.2 2.8 2.4 2.0 1.6
Chemical shift (ppm)

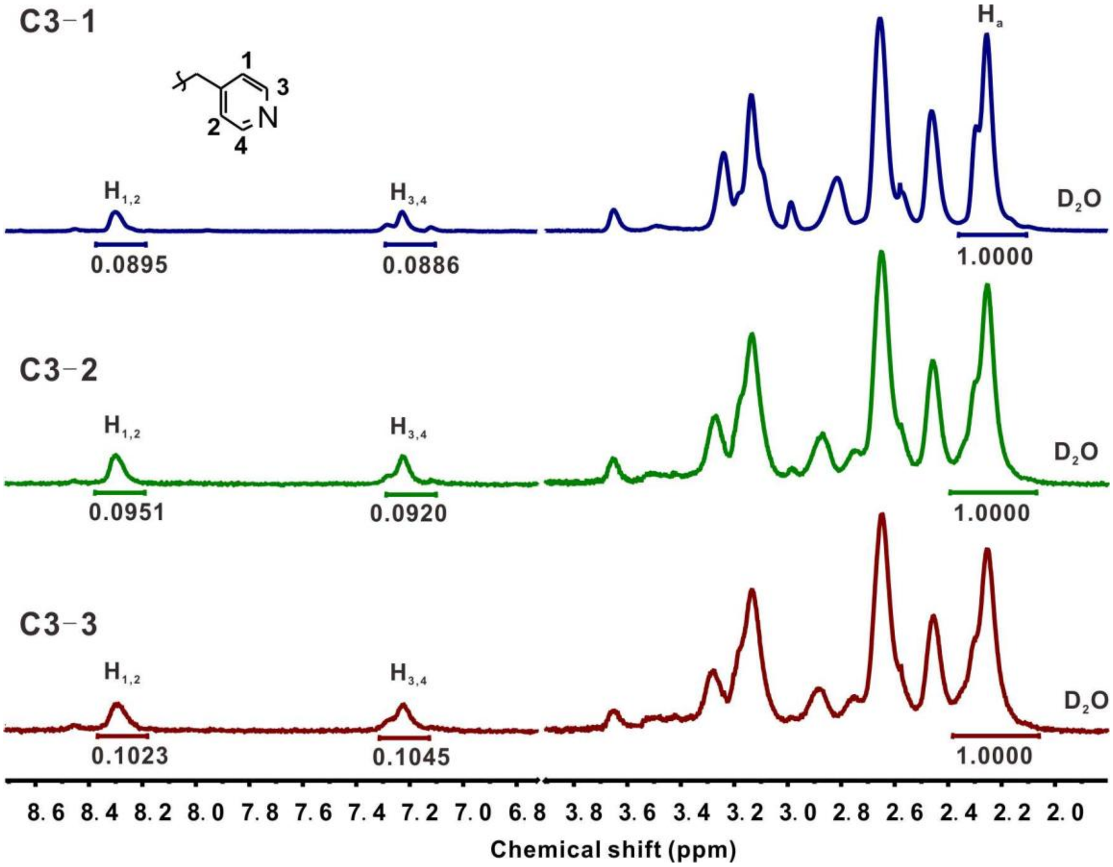

C3−1
C3−2
C3−3
1
2
3
4
N
$H_{1,2}$
$H_{3,4}$
H_a
D_2O
0.0895
0.0886
1.0000
0.0951
0.0920
1.0000
0.1023
0.1045
1.0000
8.6 8.4 8.2 8.0 7.8 7.6 7.4 7.2 7.0 6.8 3.8 3.6 3.4 3.2 3.0 2.8 2.6 2.4 2.2 2.0
Chemical shift (ppm)

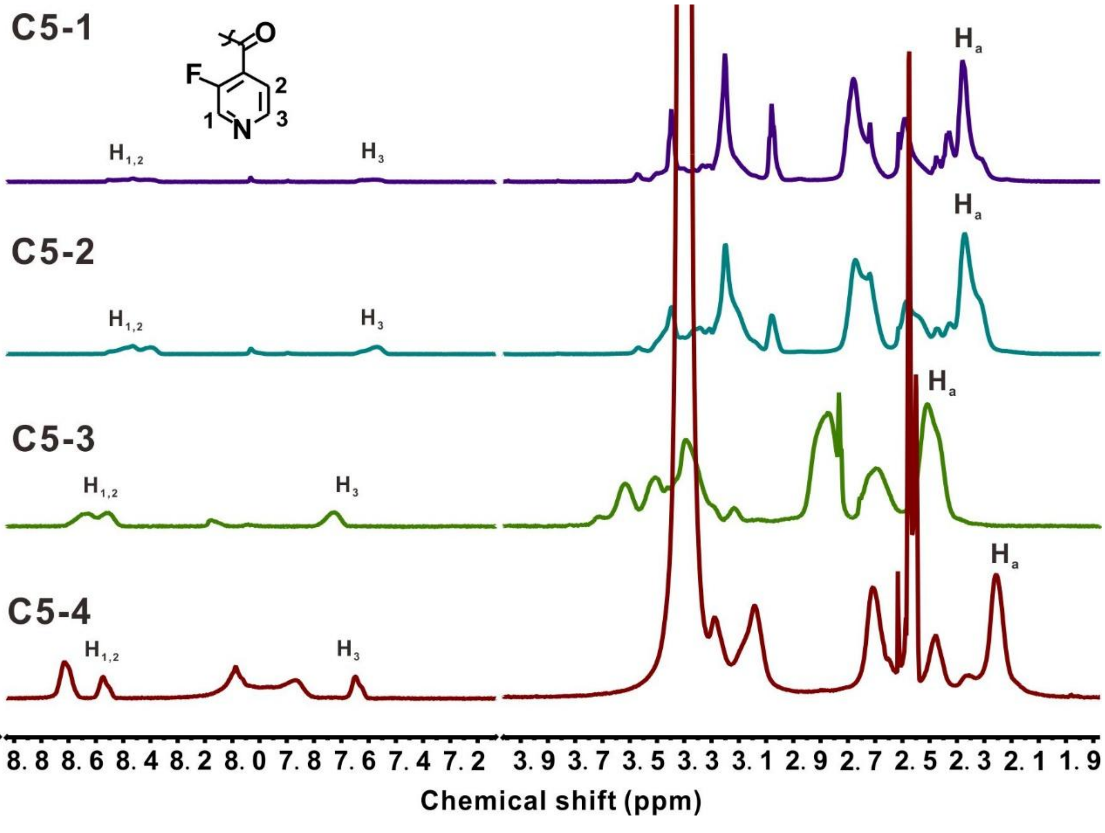

C5-1
C5-2
C5-3
C5-4
O
F
N
1
2
3
$H_{1,2}$
H_3
H_a
8.8 8.6 8.4 8.2 8.0 7.8 7.6 7.4 7.2 3.9 3.7 3.5 3.3 3.1 2.9 2.7 2.5 2.3 2.1 1.9
Chemical shift (ppm)

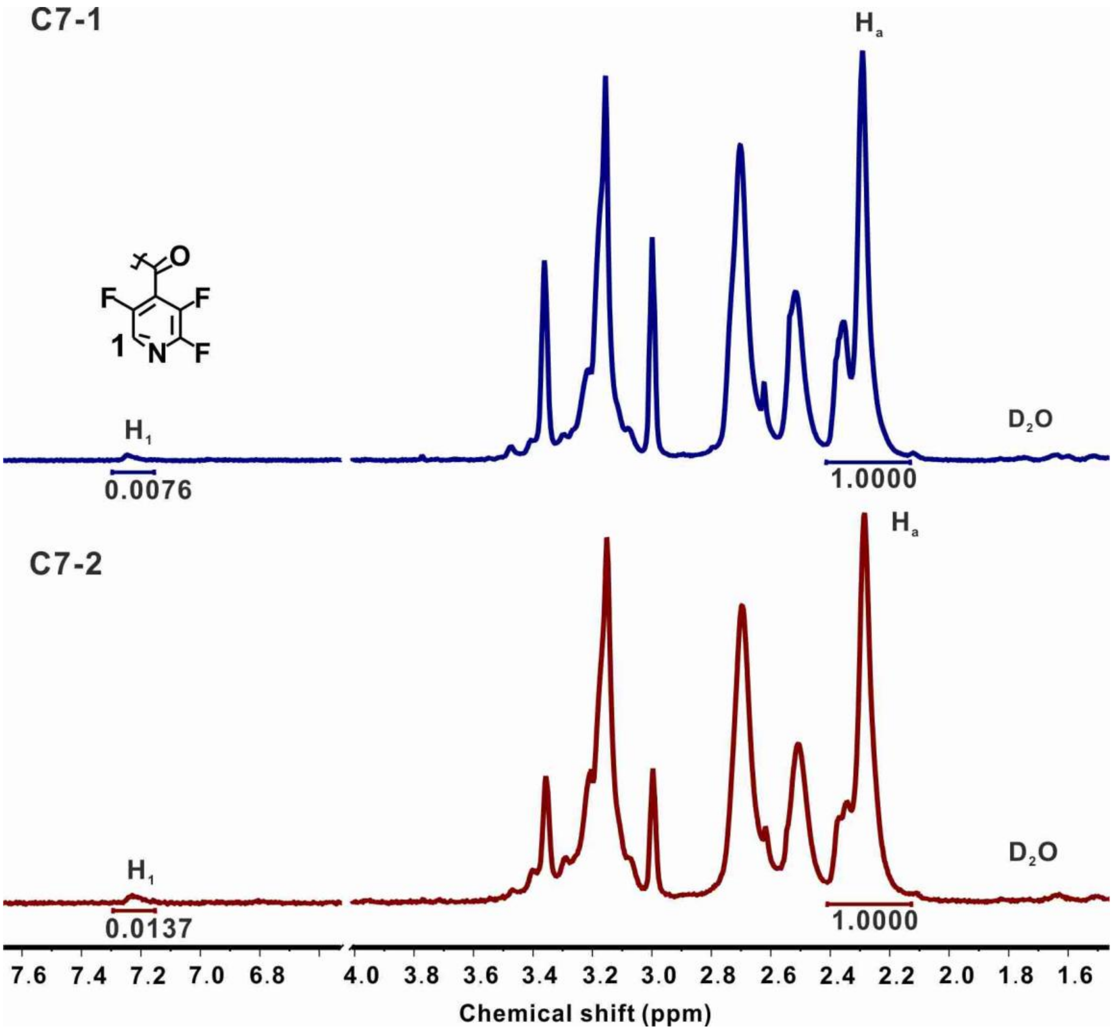

C7-1
C7-2
1
F
O
N
H_a
H_1
D_2O
0.0076
1.0000
0.0137
1.0000
7.6 7.4 7.2 7.0 6.8 4.0 3.8 3.6 3.4 3.2 3.0 2.8 2.6 2.4 2.2 2.0 1.8 1.6
Chemical shift (ppm)

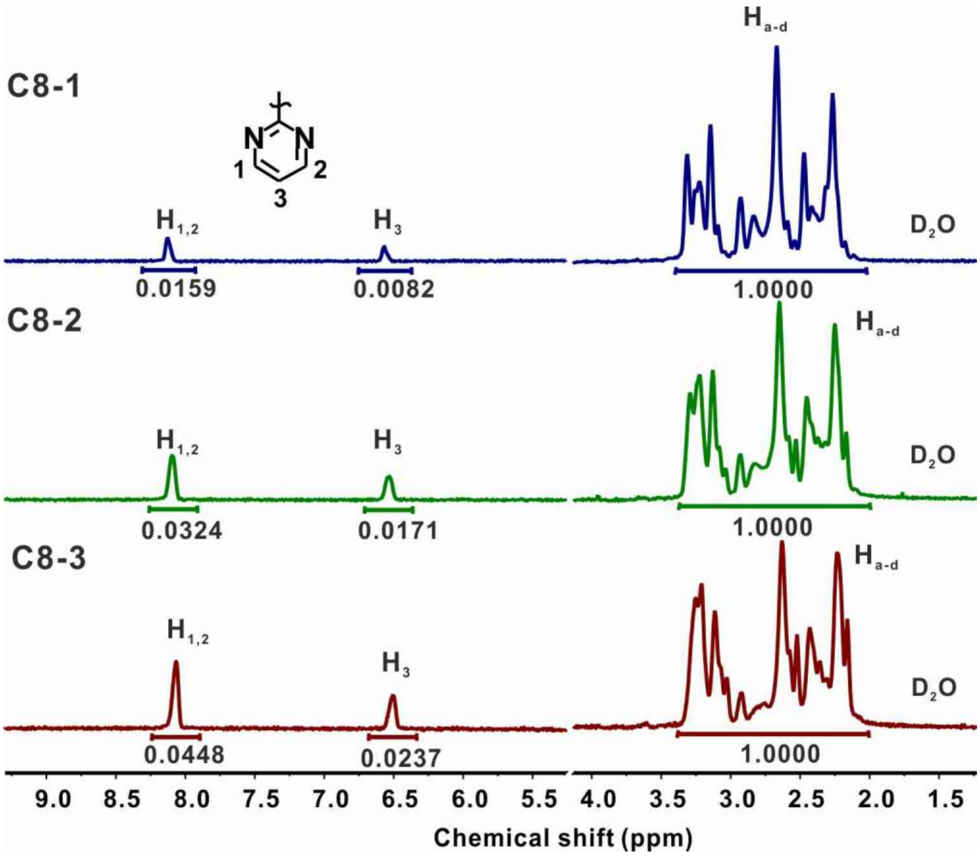
C8-1
C8-2
C8-3
1
2
3
H1,2
H3
Ha-d
D2O
0.0159
0.0082
1.0000
0.0324
0.0171
0.0448
0.0237
9.0 8.5 8.0 7.5 7.0 6.5 6.0 5.5 4.0 3.5 3.0 2.5 2.0 1.5
Chemical shift (ppm)

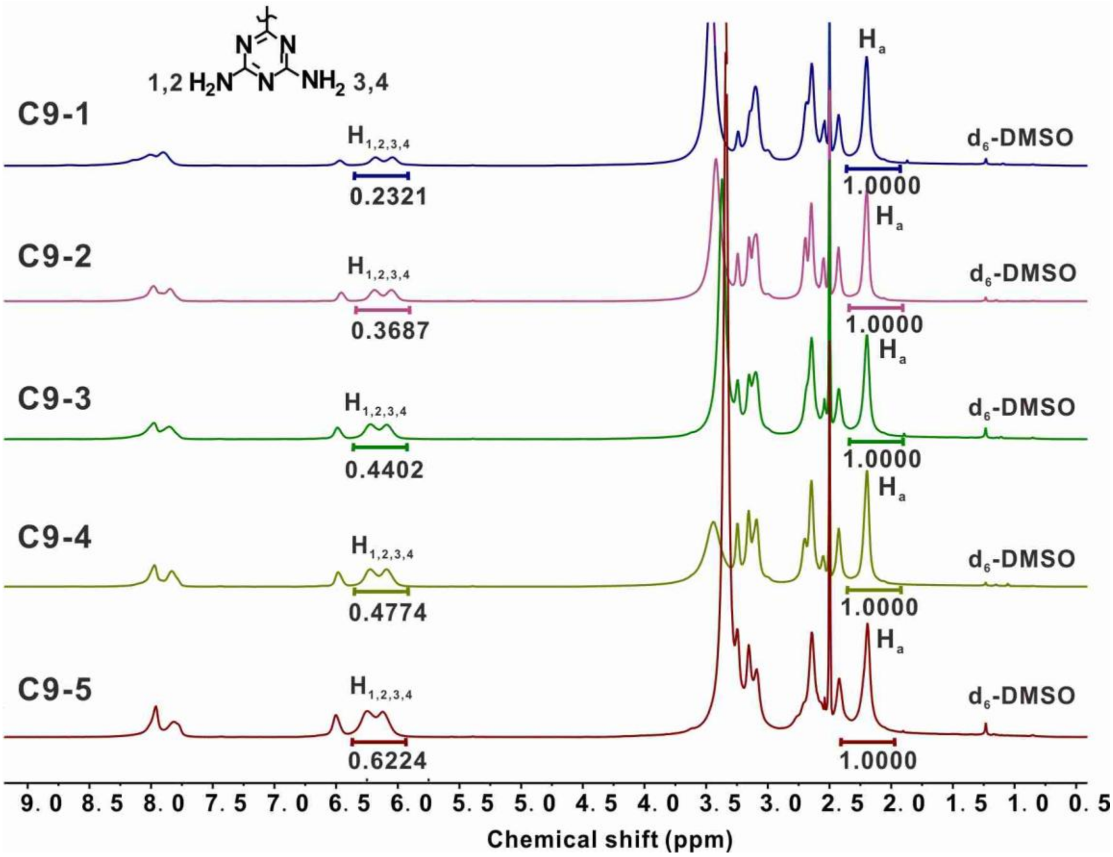
1,2 H2N NH2 3,4
C9-1
C9-2
C9-3
C9-4
C9-5
H1,2,3,4
Ha
d6-DMSO
0.2321
0.3687
0.4402
0.4774
0.6224
1.0000
9.0 8.5 8.0 7.5 7.0 6.5 6.0 5.5 5.0 4.5 4.0 3.5 3.0 2.5 2.0 1.5 1.0 0.5
Chemical shift (ppm)

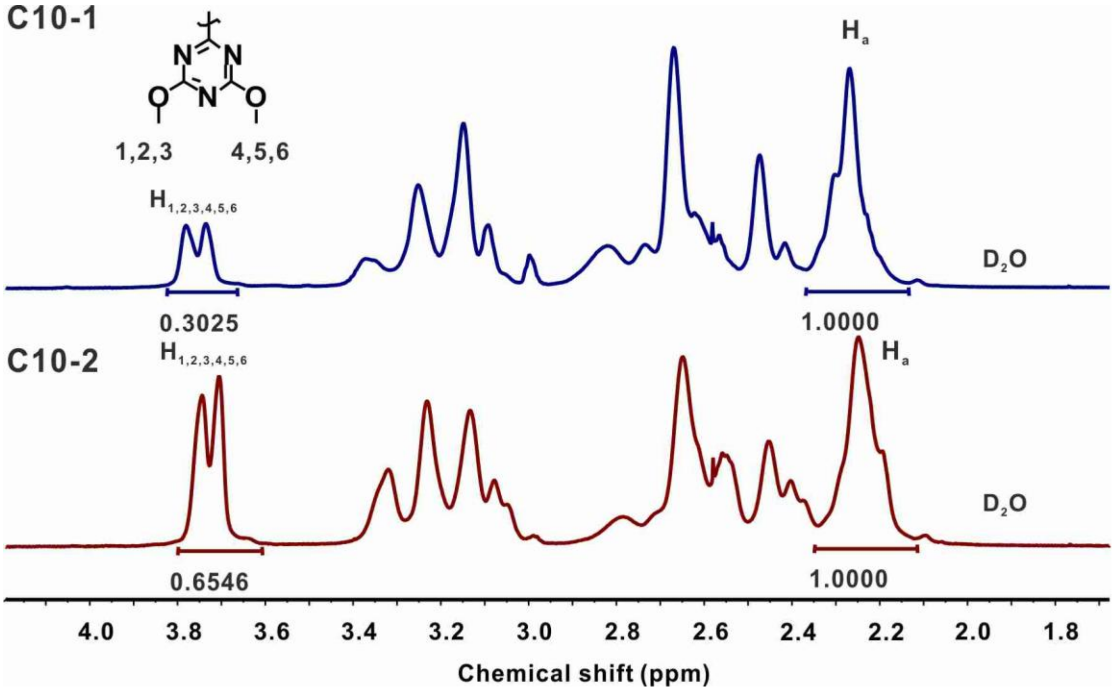
C10-1
1,2,3
4,5,6
H1,2,3,4,5,6
Ha
D2O
0.3025
1.0000
C10-2
H1,2,3,4,5,6
Ha
D2O
0.6546
1.0000
4.0 3.8 3.6 3.4 3.2 3.0 2.8 2.6 2.4 2.2 2.0 1.8
Chemical shift (ppm)

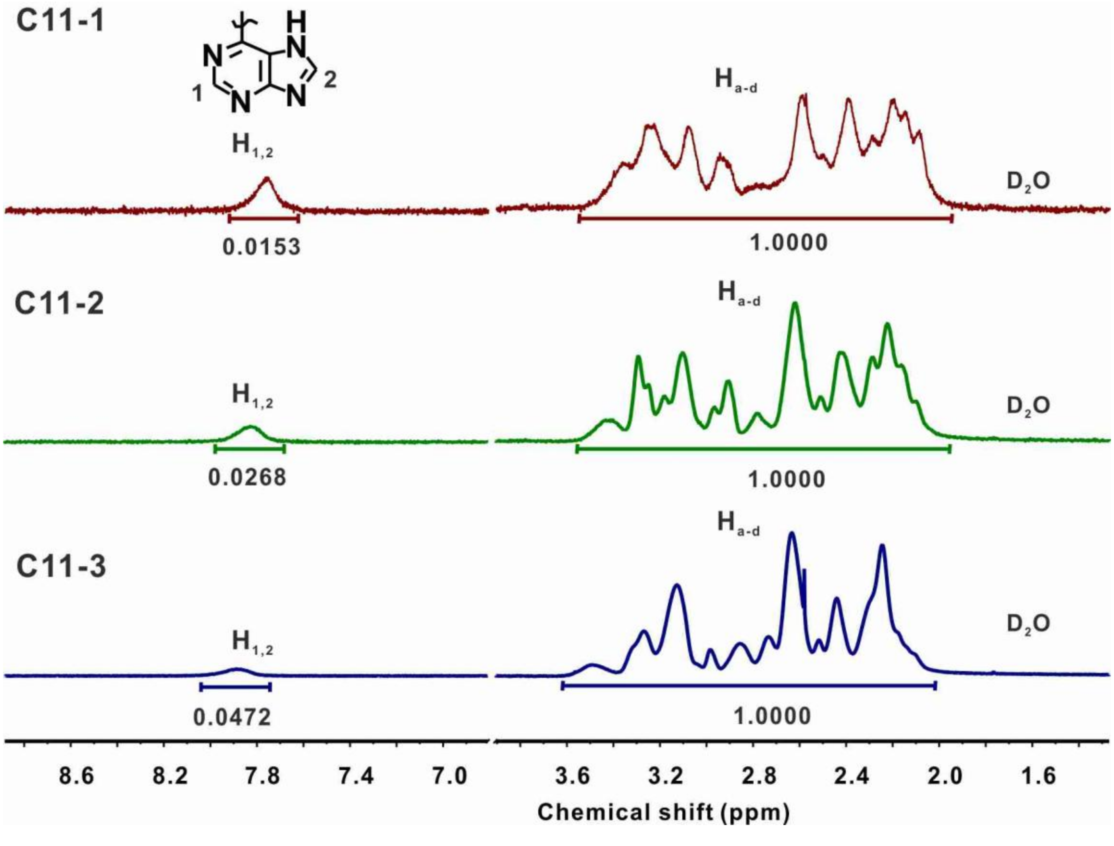
C11-1
1
2
H1,2
Ha-d
D2O
0.0153
1.0000
C11-2
H1,2
Ha-d
D2O
0.0268
1.0000
C11-3
H1,2
Ha-d
D2O
0.0472
1.0000
8.6 8.2 7.8 7.4 7.0 3.6 3.2 2.8 2.4 2.0 1.6
Chemical shift (ppm)

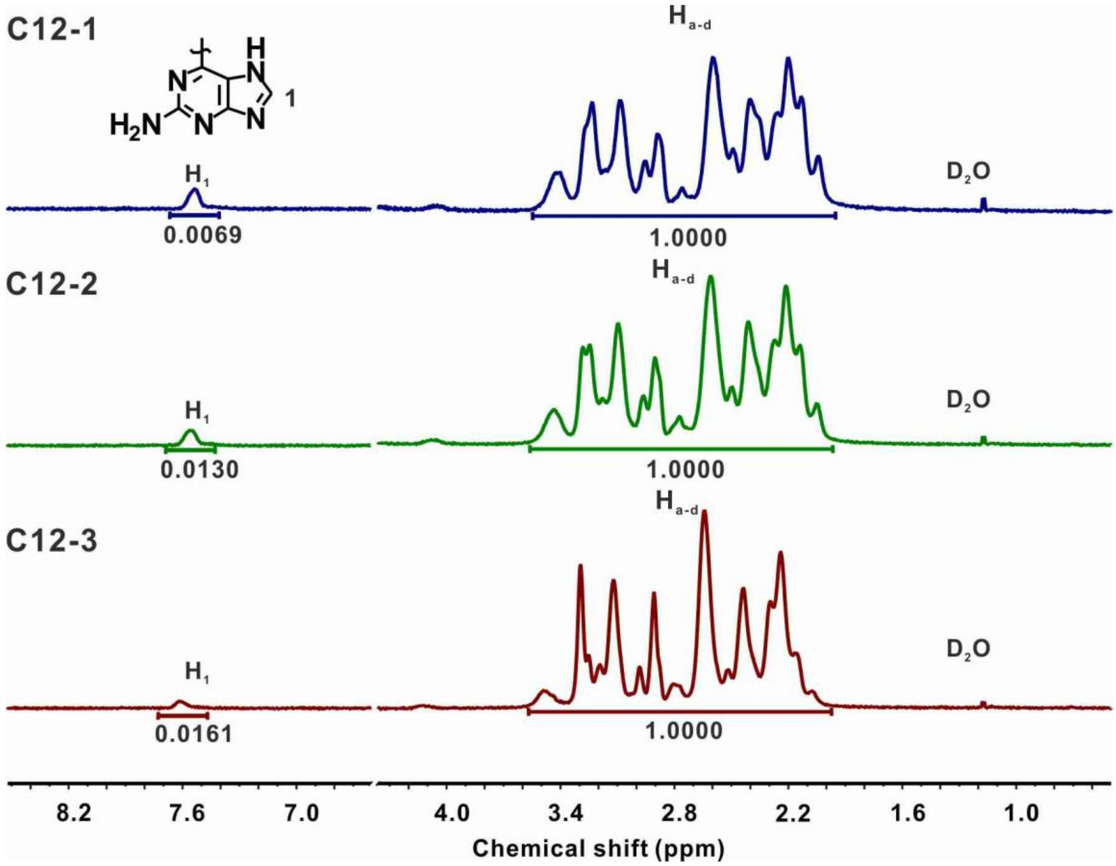

C12-1
H
N
N
N
H₂N
N
N
1
H_1
0.0069
H_{a-d}
1.0000
D_2O
C12-2
H_1
0.0130
H_{a-d}
1.0000
D_2O
C12-3
H_1
0.0161
H_{a-d}
1.0000
D_2O
8.2
7.6
7.0
4.0
3.4
2.8
2.2
1.6
1.0
Chemical shift (ppm)

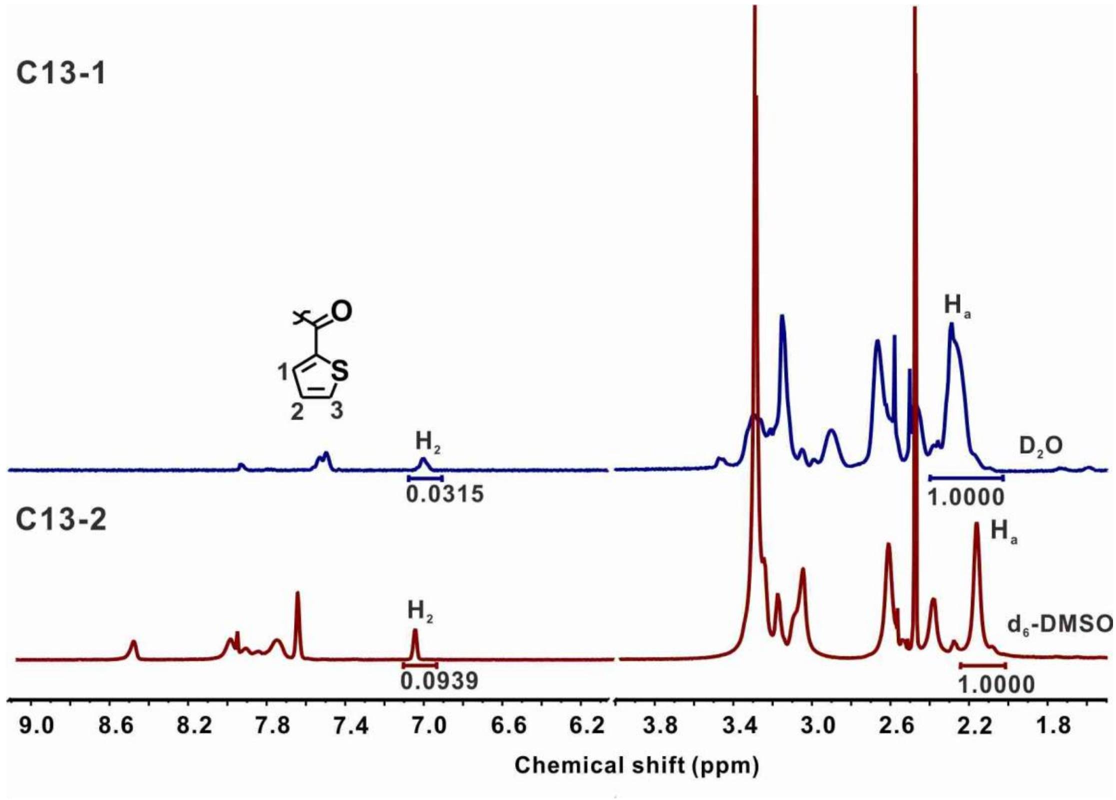

C13-1
O
1
S
2
3
H_2
0.0315
H_a
1.0000
D_2O
C13-2
H_2
0.0939
H_a
1.0000
d_6-DMSO
9.0
8.6
8.2
7.8
7.4
7.0
6.6
6.2
3.8
3.4
3.0
2.6
2.2
1.8
Chemical shift (ppm)

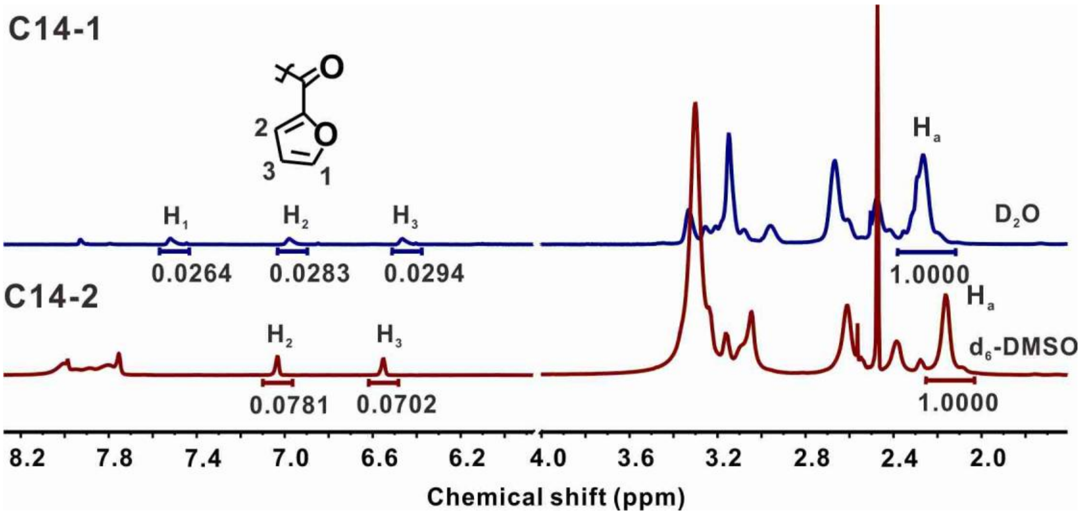
C14-1
2
3
1
O
O
H1
H2
H3
0.0264
0.0283
0.0294
Ha
D2O
1.0000
C14-2
H2
H3
0.0781
0.0702
Ha
d6-DMSO
1.0000
8.2 7.8 7.4 7.0 6.6 6.2 4.0 3.6 3.2 2.8 2.4 2.0
Chemical shift (ppm)

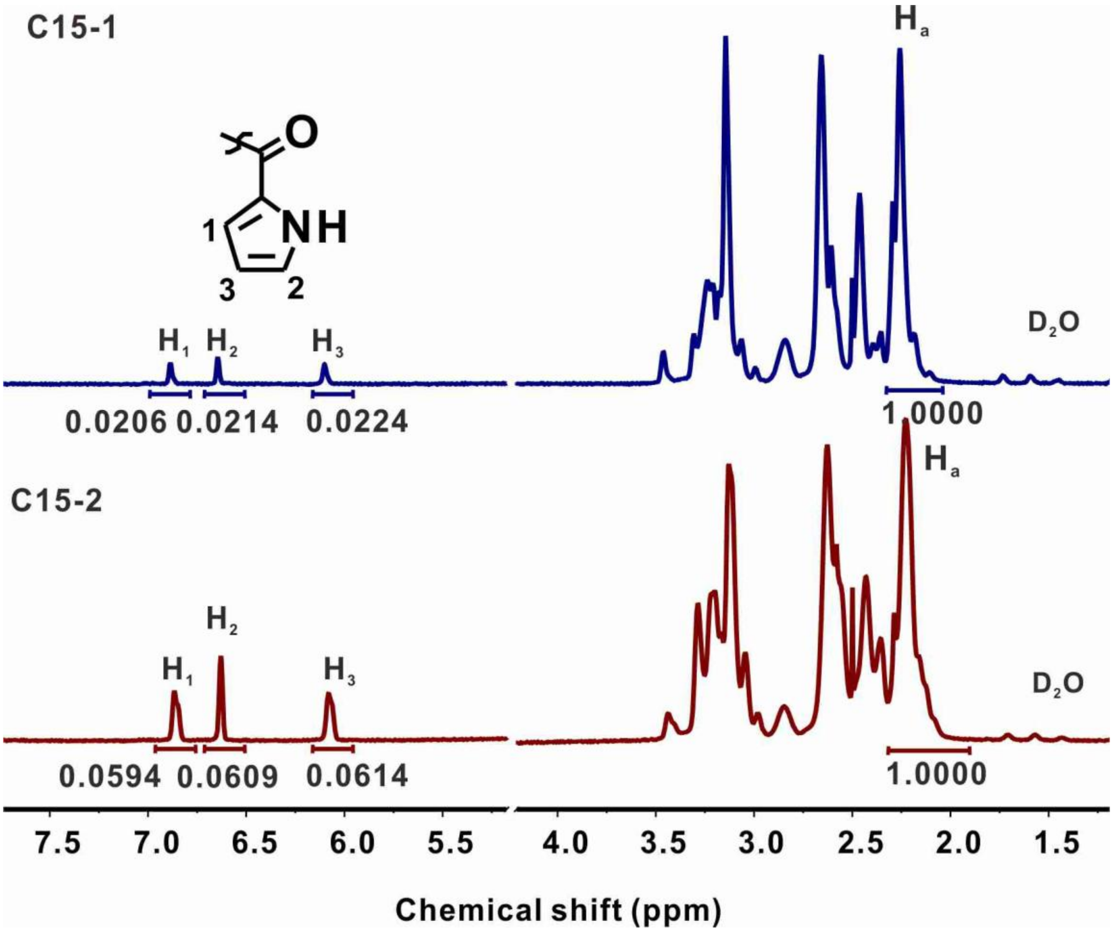
C15-1
O
1
NH
3
2
H1
H2
H3
0.0206
0.0214
0.0224
Ha
D2O
1.0000
C15-2
H1
H2
H3
0.0594
0.0609
0.0614
Ha
D2O
1.0000
7.5 7.0 6.5 6.0 5.5 4.0 3.5 3.0 2.5 2.0 1.5
Chemical shift (ppm)

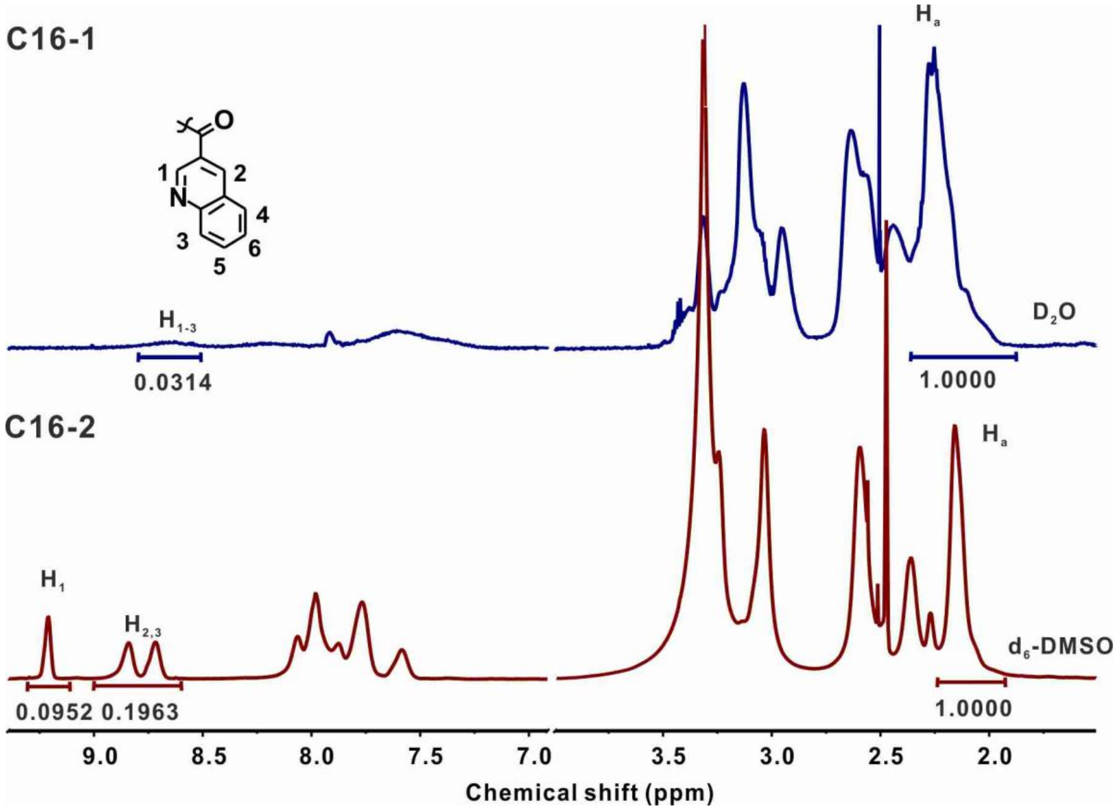

C16-1
O
1
2
N
4
3
6
5
H1-3
0.0314
Ha
D2O
1.0000
C16-2
H1
H2,3
0.0952 0.1963
Ha
d6-DMSO
1.0000
9.0
8.5
8.0
7.5
7.0
3.5
3.0
2.5
2.0
Chemical shift (ppm)

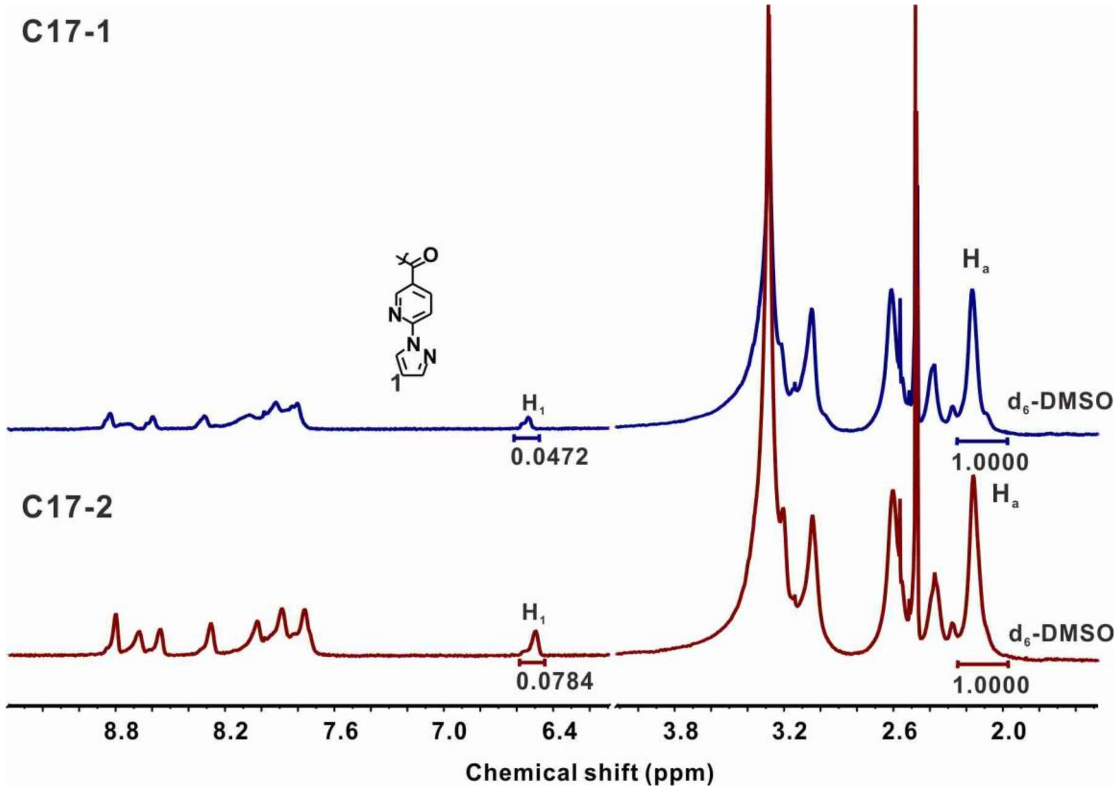

C17-1
O
N
N
N
1
H1
0.0472
Ha
d6-DMSO
1.0000
C17-2
H1
0.0784
Ha
d6-DMSO
1.0000
8.8
8.2
7.6
7.0
6.4
3.8
3.2
2.6
2.0
Chemical shift (ppm)

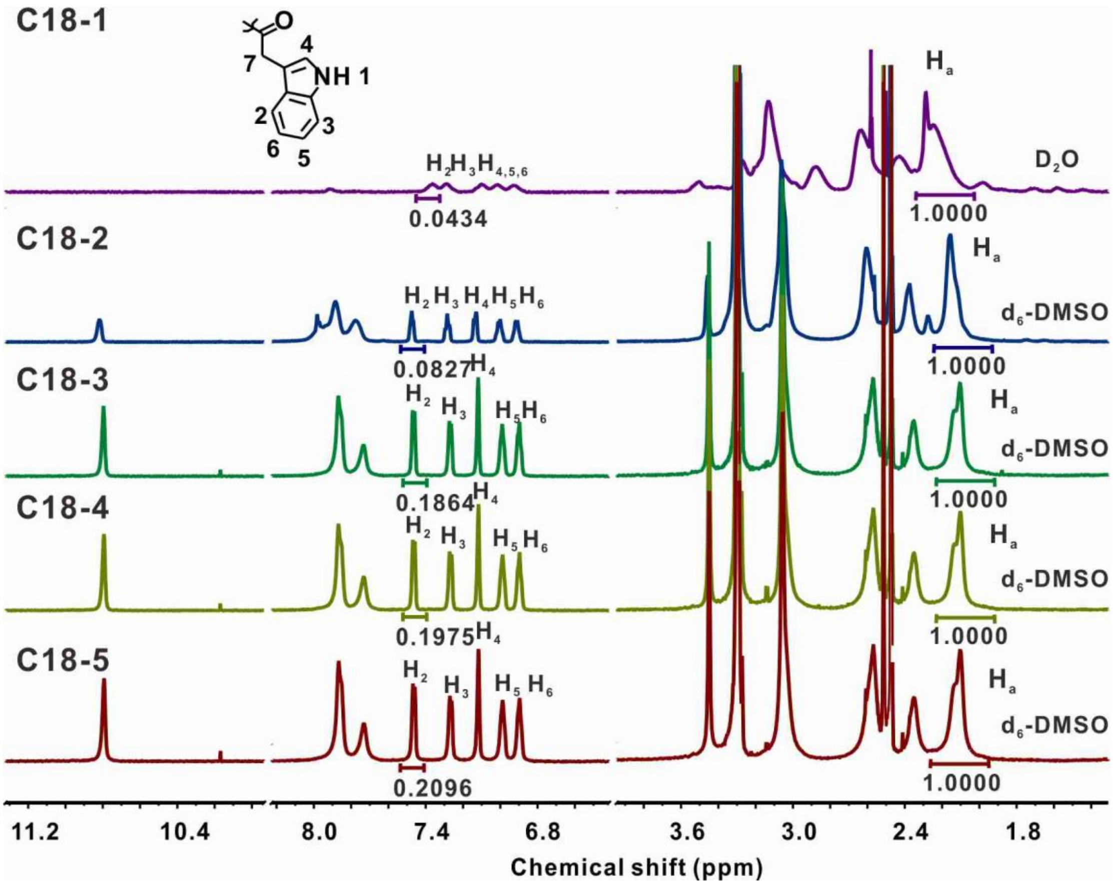
C18-1
C18-2
C18-3
C18-4
C18-5
D2O
d6-DMSO
0.0434
0.0827
0.1864
0.1975
0.2096
1.0000
11.2
10.4
8.0
7.4
6.8
3.6
3.0
2.4
1.8
Chemical shift (ppm)

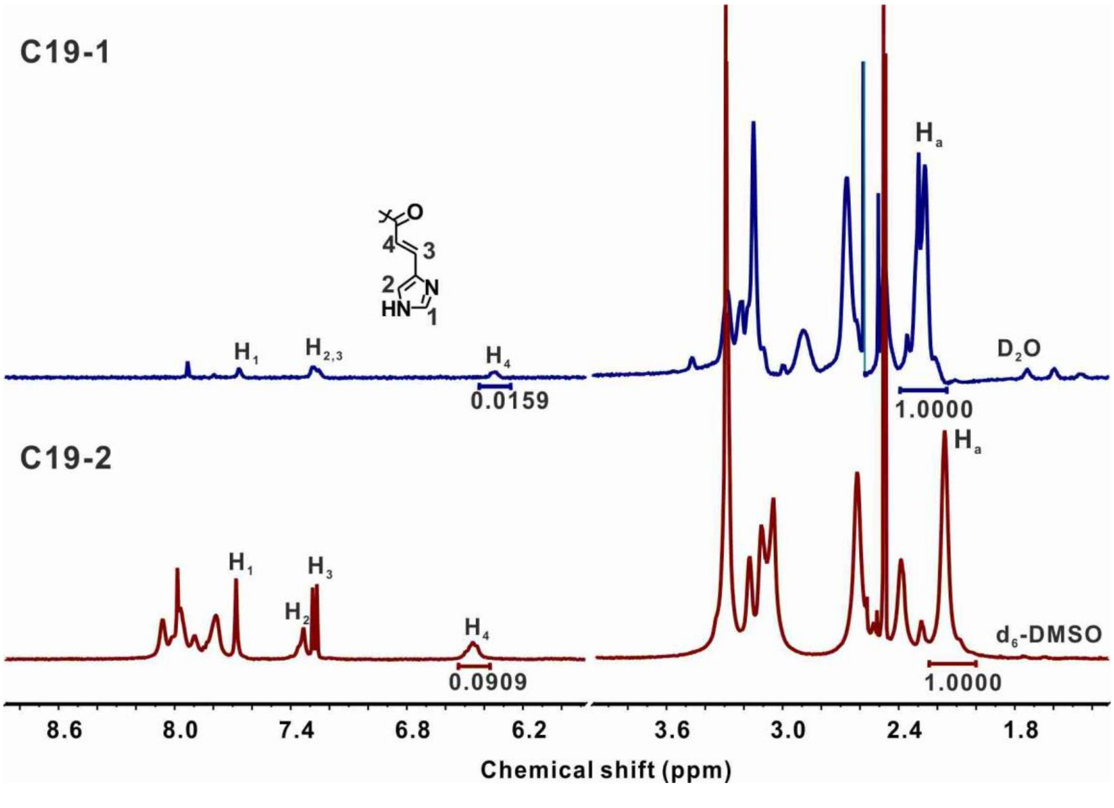
C19-1
C19-2
D2O
d6-DMSO
0.0159
0.0909
1.0000
8.6
8.0
7.4
6.8
6.2
3.6
3.0
2.4
1.8
Chemical shift (ppm)

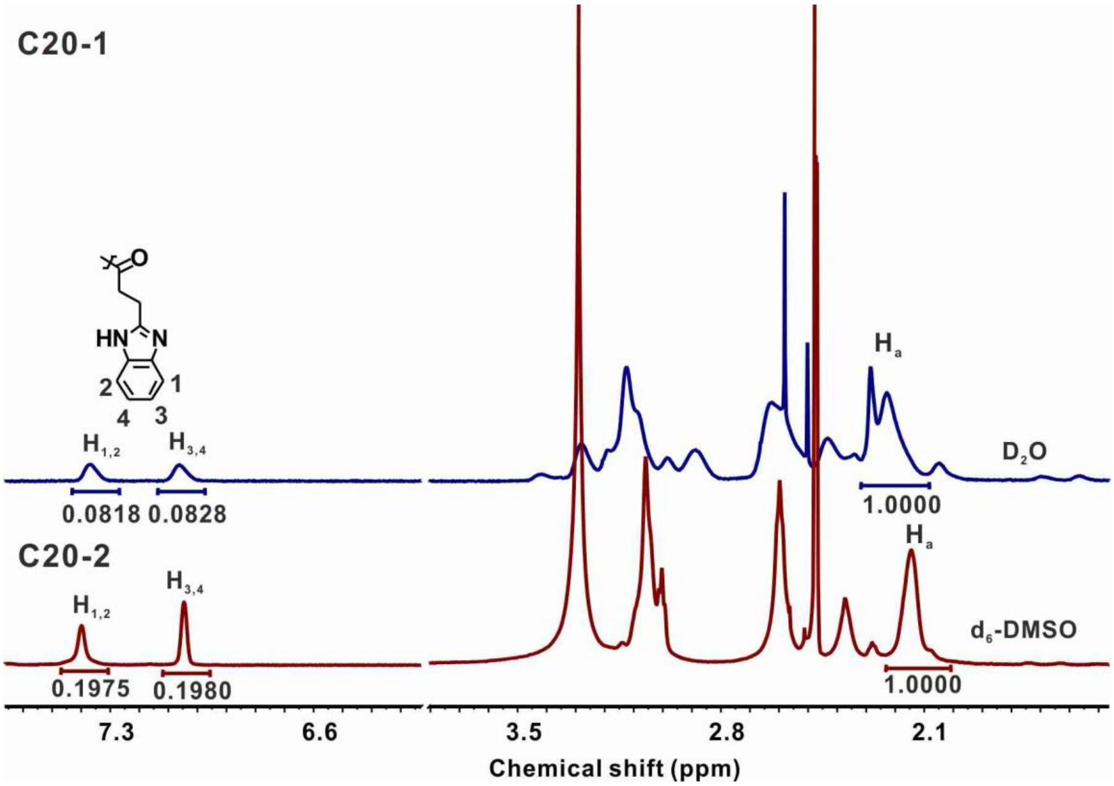
C20-1
HN
N
2
1
4
3
H1,2
H3,4
0.0818
0.0828
Ha
D2O
1.0000
C20-2
H1,2
H3,4
0.1975
0.1980
Ha
d6-DMSO
1.0000
7.3
6.6
3.5
2.8
2.1
Chemical shift (ppm)

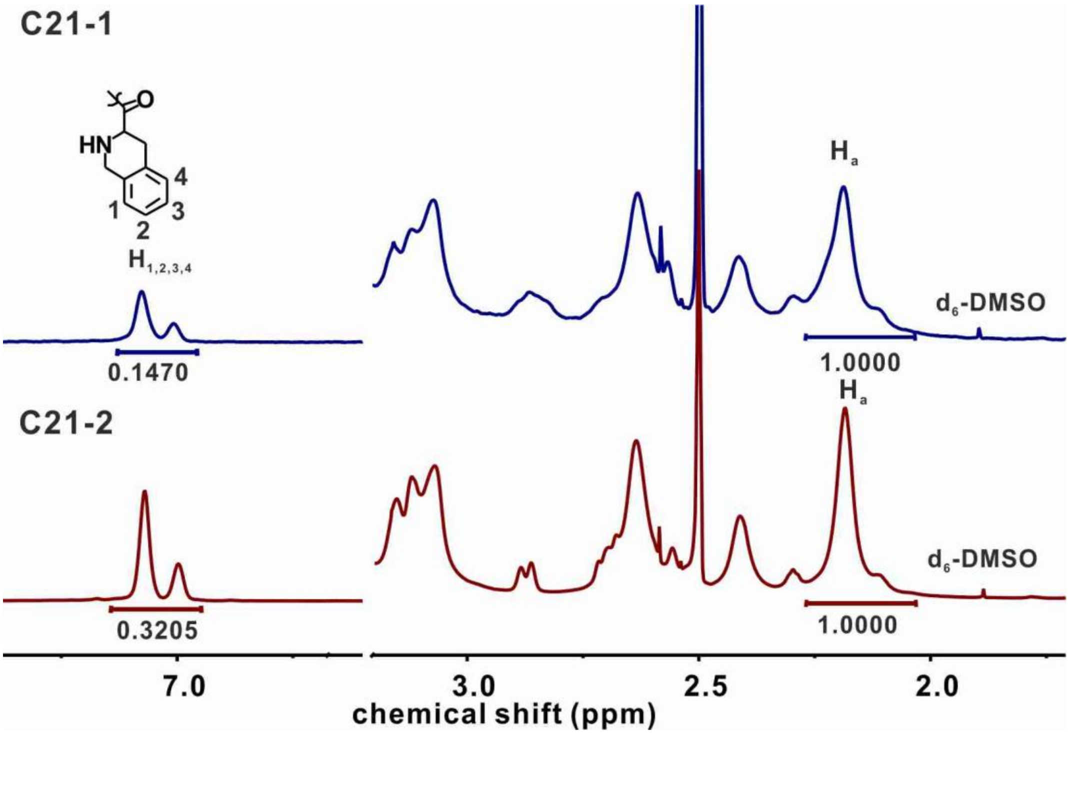
C21-1
HN
4
1
3
2
H1,2,3,4
0.1470
Ha
d6-DMSO
1.0000
C21-2
Ha
0.3205
d6-DMSO
1.0000
7.0
3.0
2.5
2.0
chemical shift (ppm)

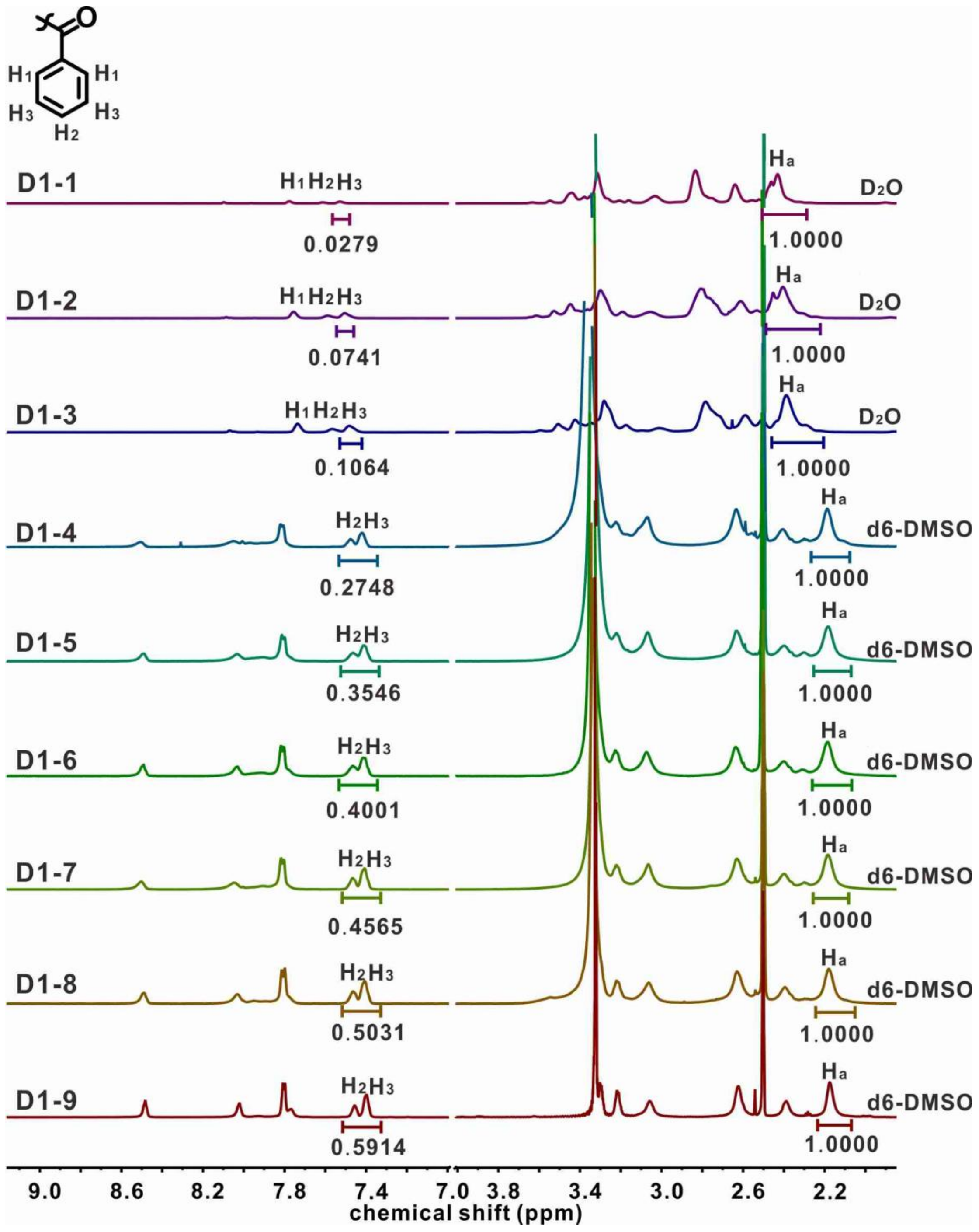

H1 H1 H3 H3 H2
D1-1
H1 H2 H3
0.0279
Ha
D2O
1.0000
D1-2
H1 H2 H3
0.0741
Ha
D2O
1.0000
D1-3
H1 H2 H3
0.1064
Ha
D2O
1.0000
D1-4
H2 H3
0.2748
Ha
d6-DMSO
1.0000
D1-5
H2 H3
0.3546
Ha
d6-DMSO
1.0000
D1-6
H2 H3
0.4001
Ha
d6-DMSO
1.0000
D1-7
H2 H3
0.4565
Ha
d6-DMSO
1.0000
D1-8
H2 H3
0.5031
Ha
d6-DMSO
1.0000
D1-9
H2 H3
0.5914
Ha
d6-DMSO
1.0000
9.0 8.6 8.2 7.8 7.4 7.0 3.8 3.4 3.0 2.6 2.2
chemical shift (ppm)

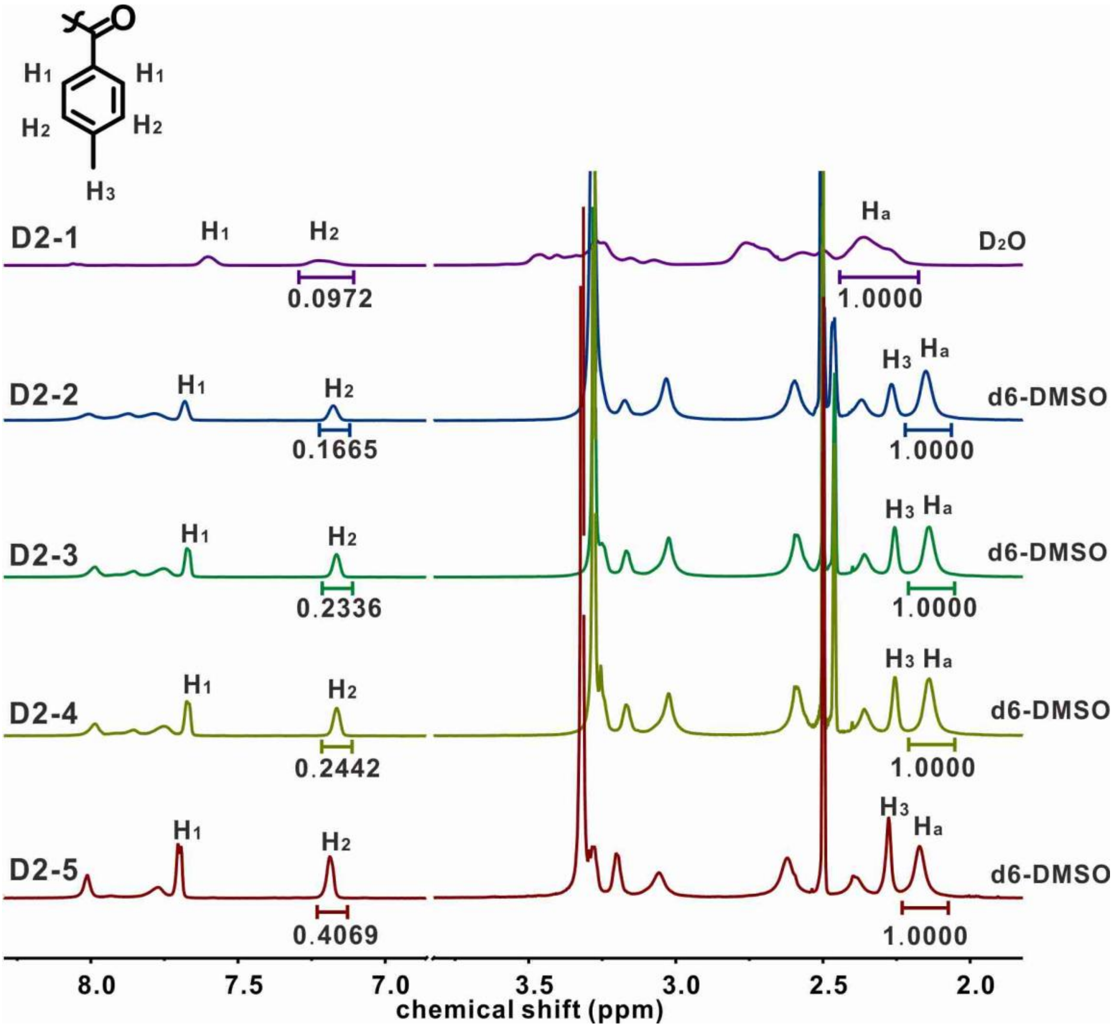
H1
H1
H2
H2
H3
D2-1
H1
H2
Ha
D2O
0.0972
1.0000
D2-2
H1
H2
H3
Ha
d6-DMSO
0.1665
1.0000
D2-3
H1
H2
H3
Ha
d6-DMSO
0.2336
1.0000
D2-4
H1
H2
H3
Ha
d6-DMSO
0.2442
1.0000
D2-5
H1
H2
H3
Ha
d6-DMSO
0.4069
1.0000
8.0
7.5
7.0
3.5
3.0
2.5
2.0
chemical shift (ppm)

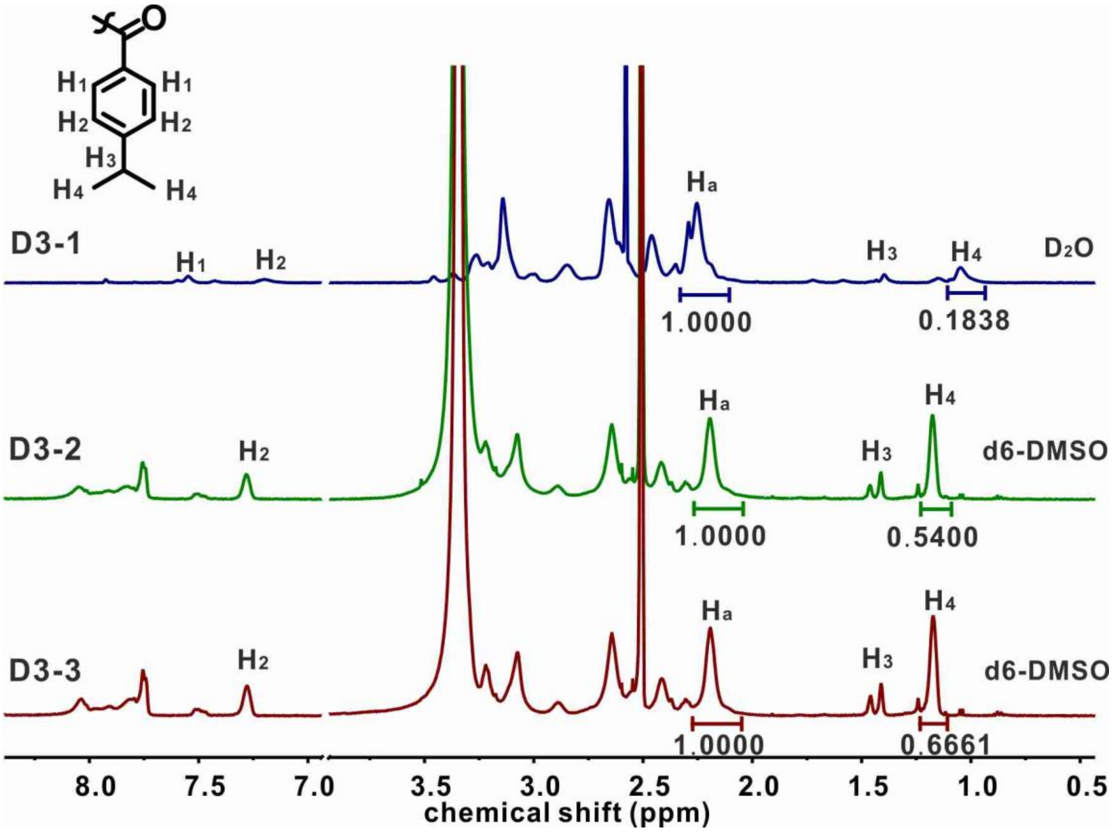

H1
H2
H3
H4
D3-1
D3-2
D3-3
Ha
D2O
d6-DMSO
1.0000
0.1838
0.5400
0.6661
8.0
7.5
7.0
3.5
3.0
2.5
2.0
1.5
1.0
0.5
chemical shift (ppm)

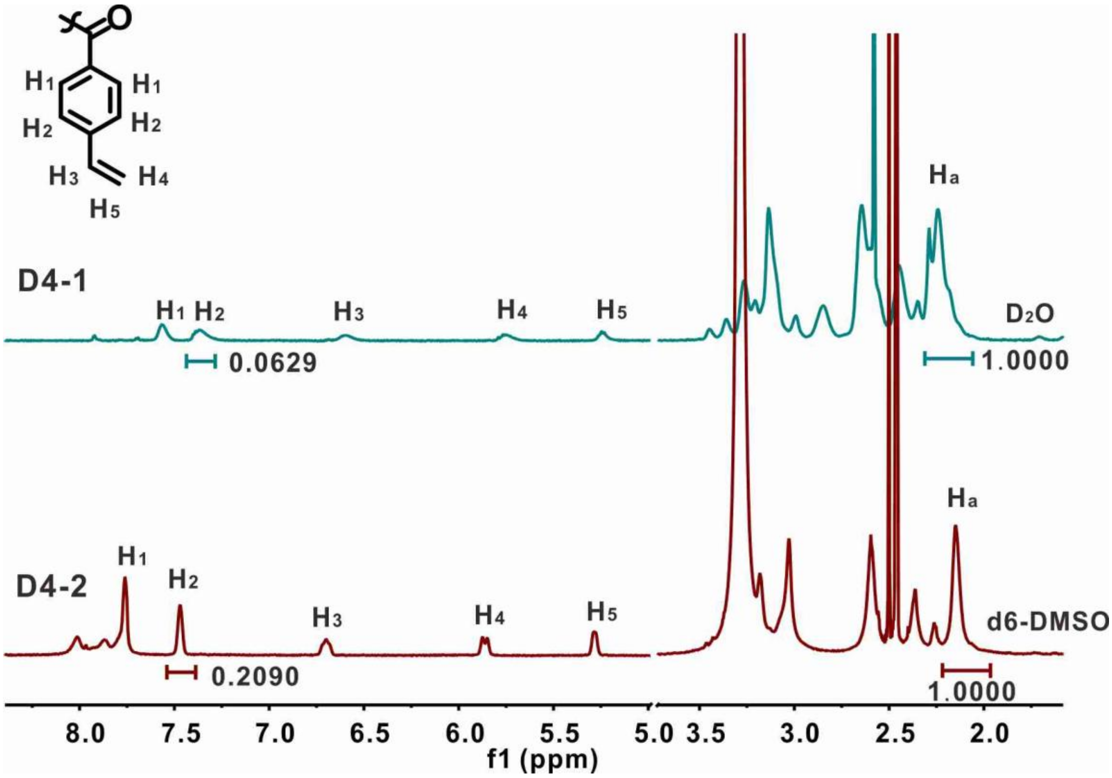

H1
H2
H3
H4
H5
D4-1
D4-2
Ha
D2O
d6-DMSO
0.0629
1.0000
0.2090
8.0
7.5
7.0
6.5
6.0
5.5
5.0
3.5
3.0
2.5
2.0
f1 (ppm)

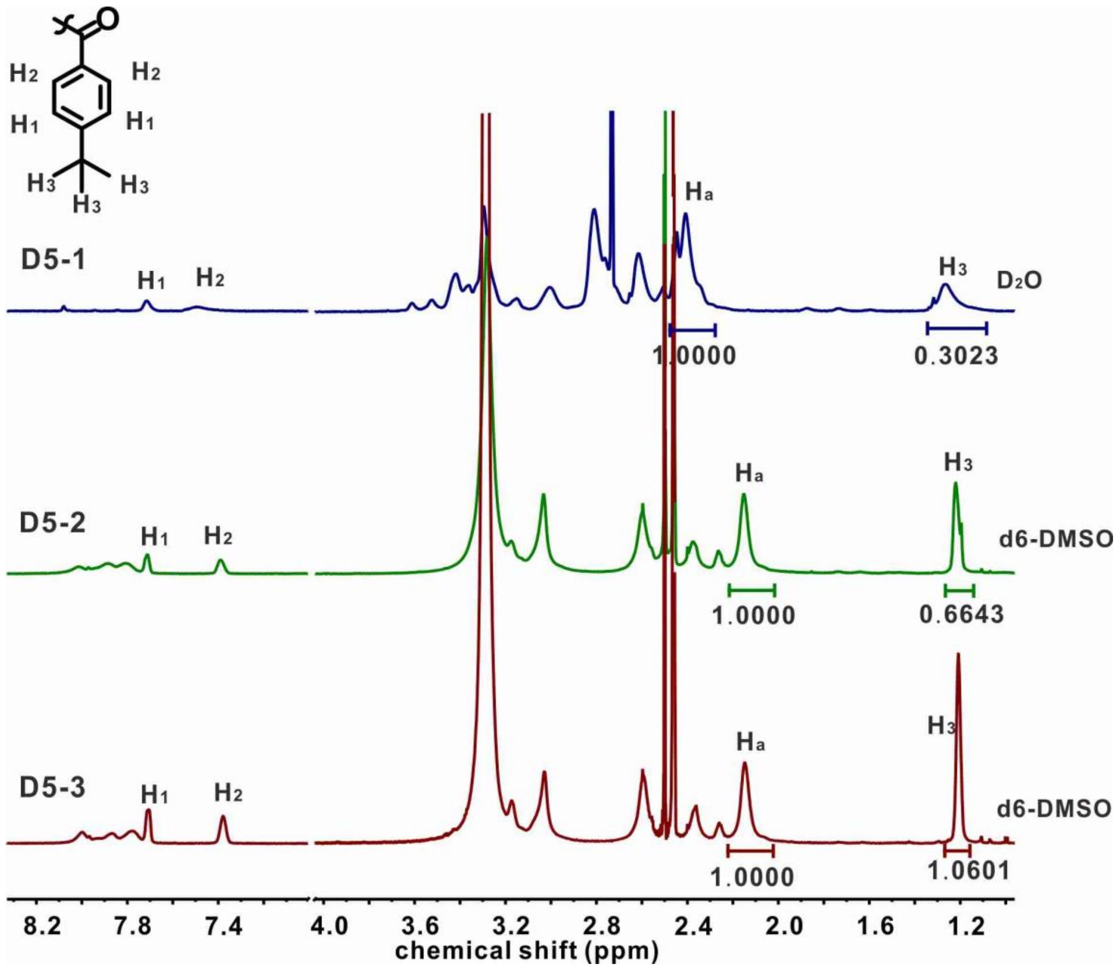
D5-1
H1
H2
Ha
H3
D2O
1.0000
0.3023
D5-2
H1
H2
Ha
H3
d6-DMSO
1.0000
0.6643
D5-3
H1
H2
Ha
H3
d6-DMSO
1.0000
1.0601
8.2
7.8
7.4
4.0
3.6
3.2
2.8
2.4
2.0
1.6
1.2
chemical shift (ppm)

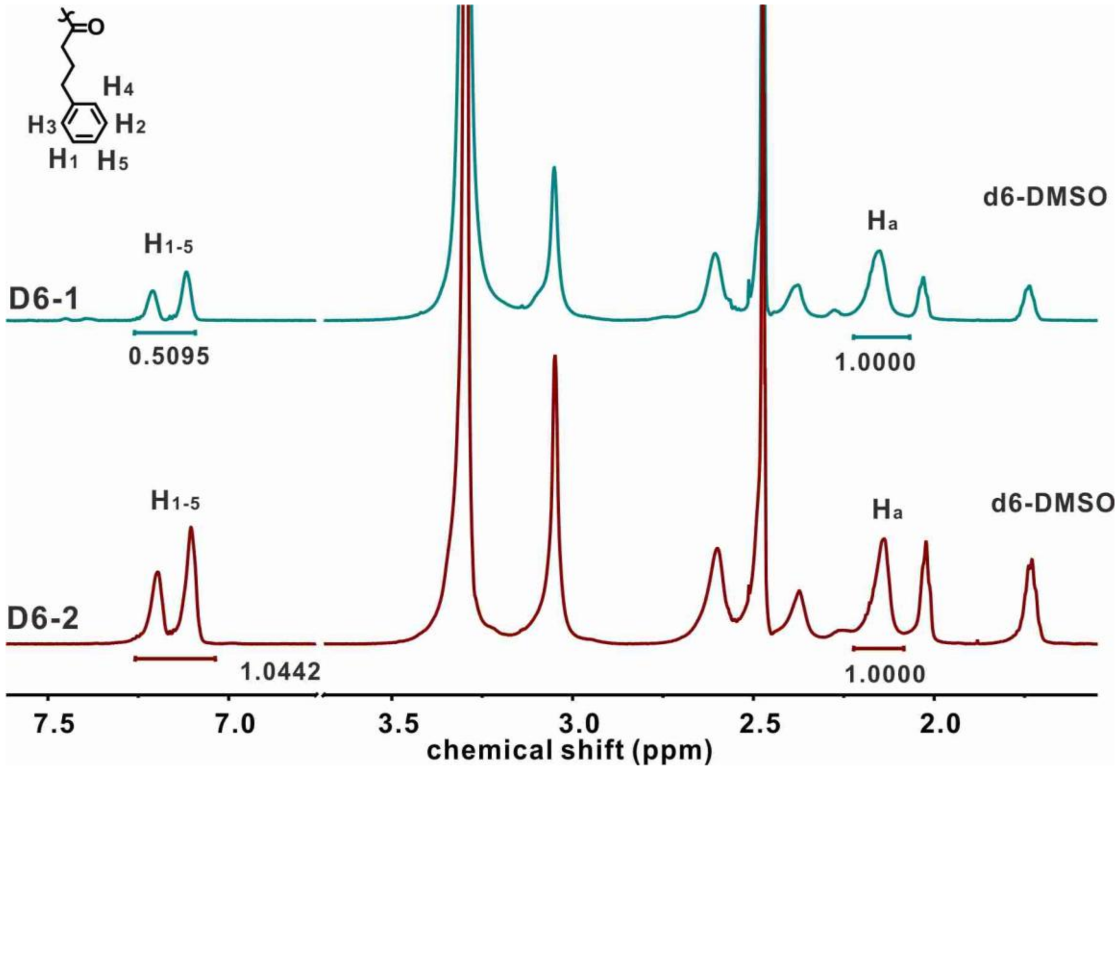
H4
H3
H2
H1
H5
d6-DMSO
H1-5
Ha
D6-1
0.5095
1.0000
H1-5
Ha
d6-DMSO
D6-2
1.0442
1.0000
7.5
7.0
3.5
3.0
2.5
2.0
chemical shift (ppm)

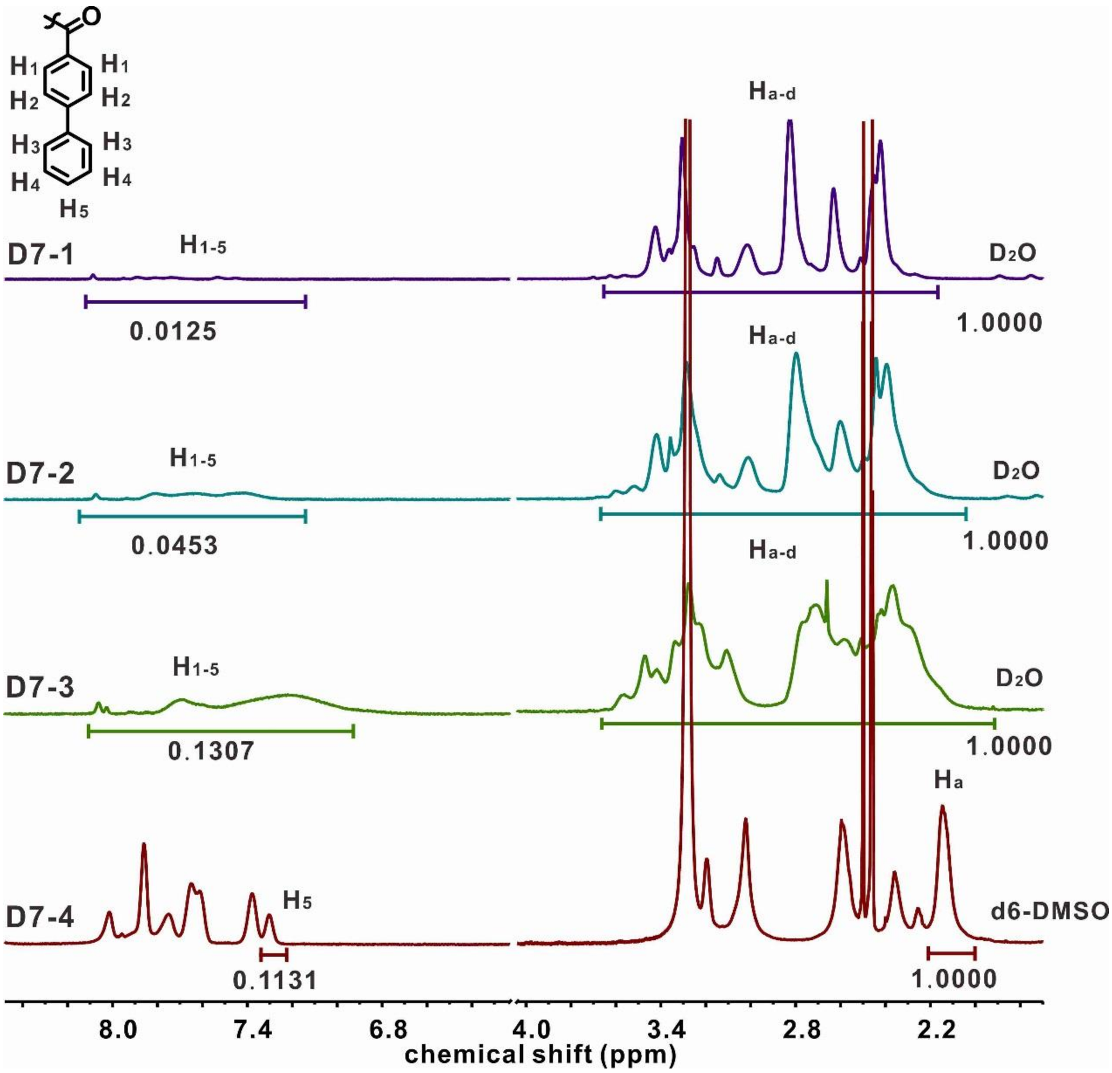

H1
H1
H2
H2
H3
H3
H4
H4
H5
D7-1
H1-5
0.0125
Ha-d
D2O
1.0000
D7-2
H1-5
0.0453
Ha-d
D2O
1.0000
D7-3
H1-5
0.1307
Ha-d
D2O
1.0000
D7-4
H5
0.1131
Ha
d6-DMSO
1.0000
8.0
7.4
6.8
4.0
3.4
2.8
2.2
chemical shift (ppm)

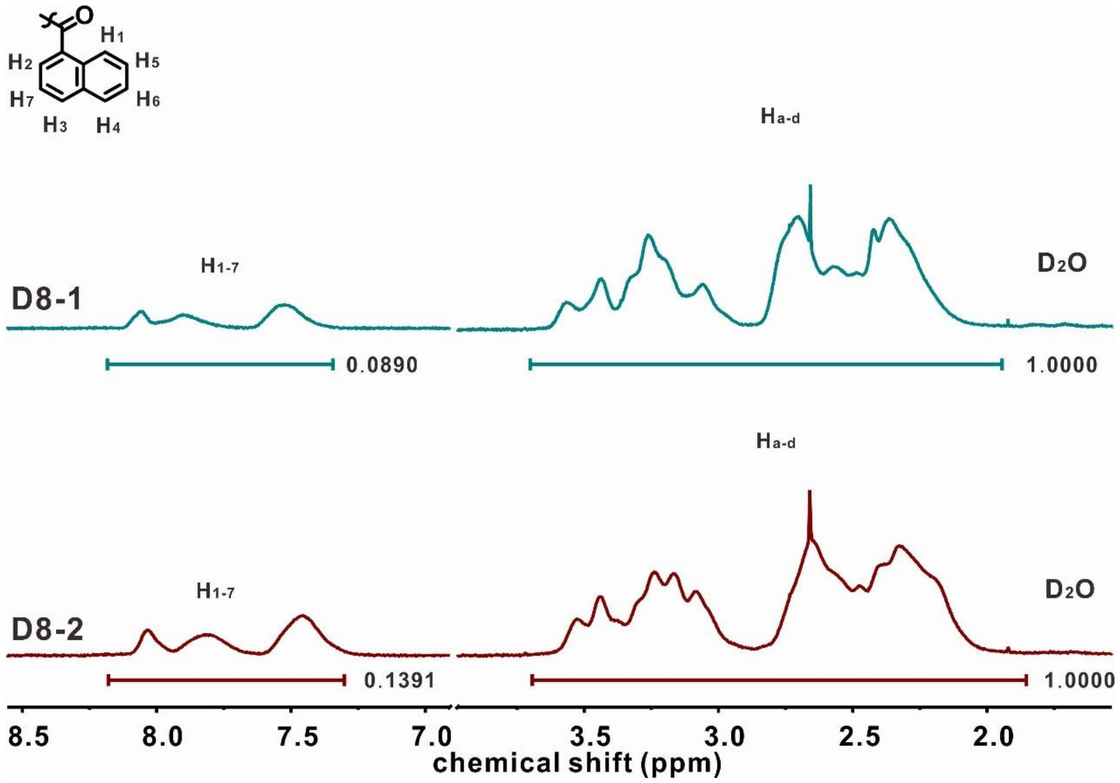
H1
H2
H5
H7
H6
H3
H4
Ha-d
H1-7
D2O
D8-1
0.0890
1.0000
Ha-d
H1-7
D2O
D8-2
0.1391
1.0000
8.5
8.0
7.5
7.0
3.5
3.0
2.5
2.0
chemical shift (ppm)

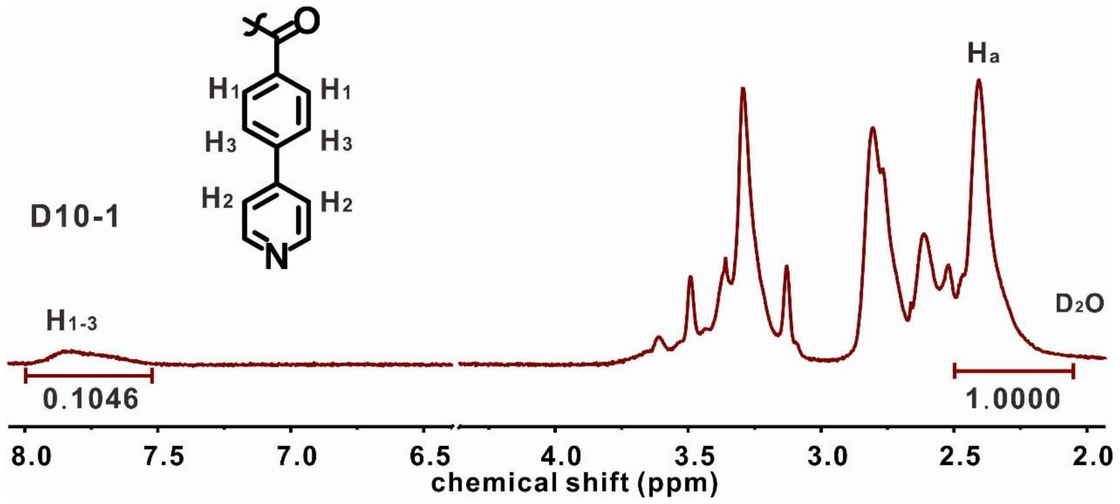
Ha
H1
H1
H3
H3
H2
H2
N
D10-1
H1-3
D2O
0.1046
1.0000
8.0
7.5
7.0
6.5
4.0
3.5
3.0
2.5
2.0
chemical shift (ppm)

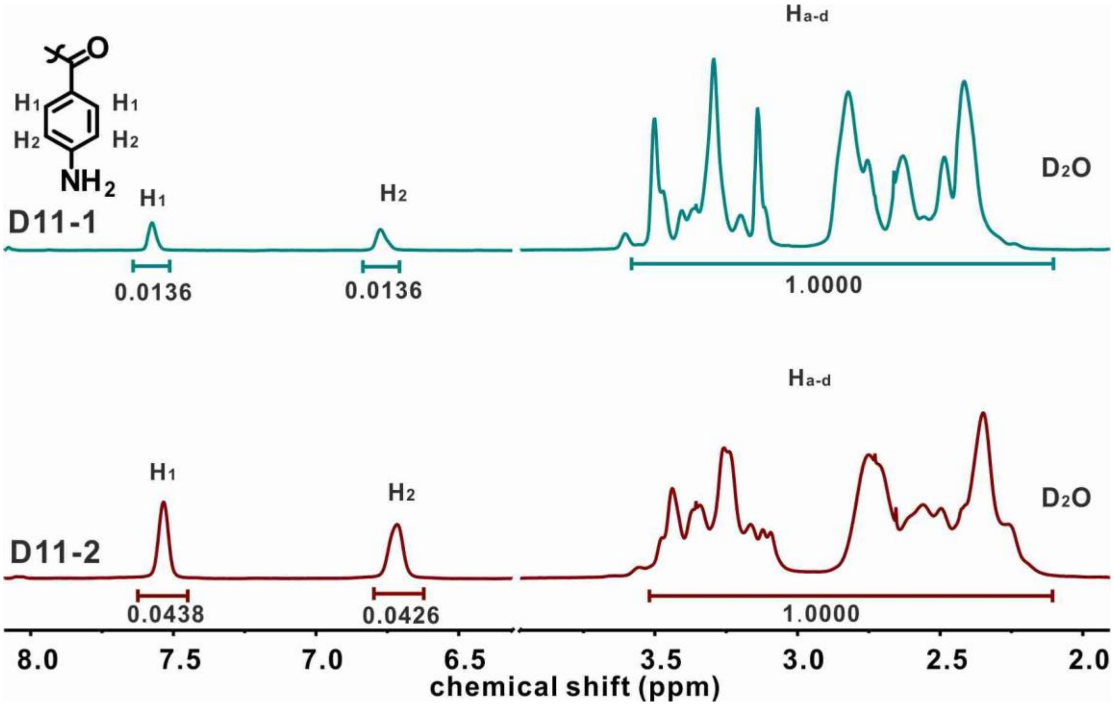

Ha-d
H1
H1
H2
H2
NH2
D2O
H1
H2
D11-1
0.0136
0.0136
1.0000
Ha-d
H1
H2
D2O
D11-2
0.0438
0.0426
1.0000
8.0
7.5
7.0
6.5
3.5
3.0
2.5
2.0
chemical shift (ppm)

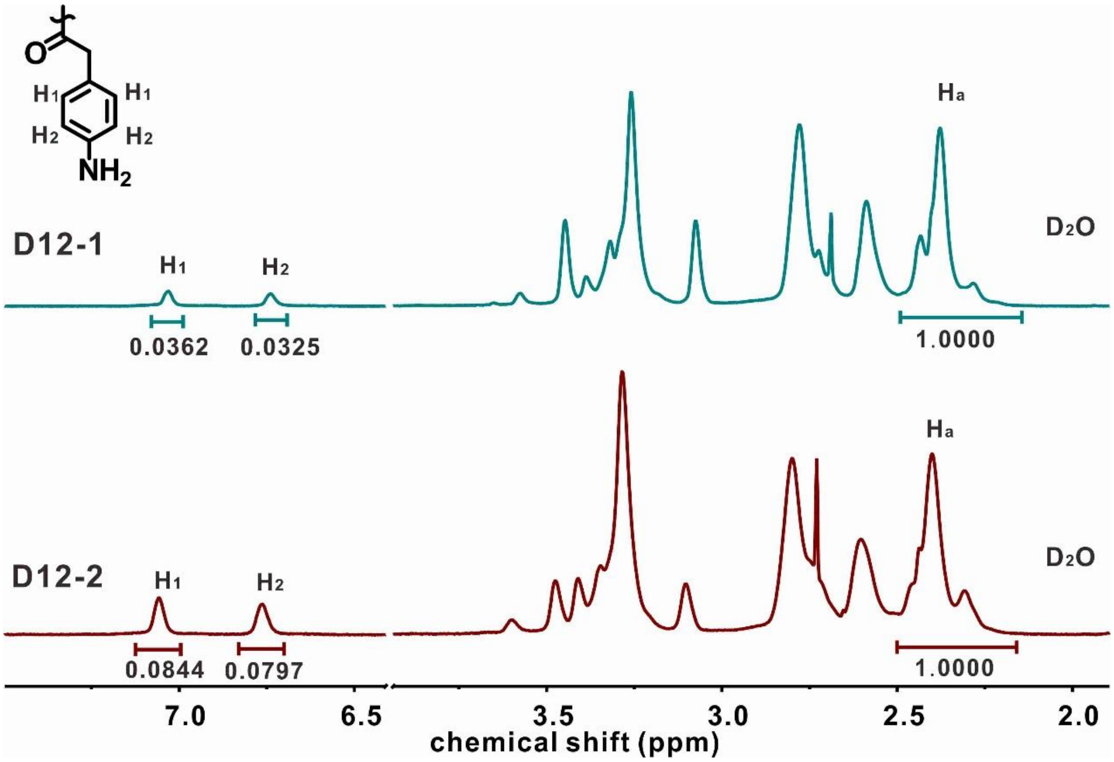

O
H1
H1
H2
H2
NH2
Ha
D2O
D12-1
H1
H2
0.0362
0.0325
1.0000
Ha
D2O
D12-2
H1
H2
0.0844
0.0797
1.0000
7.0
6.5
3.5
3.0
2.5
2.0
chemical shift (ppm)

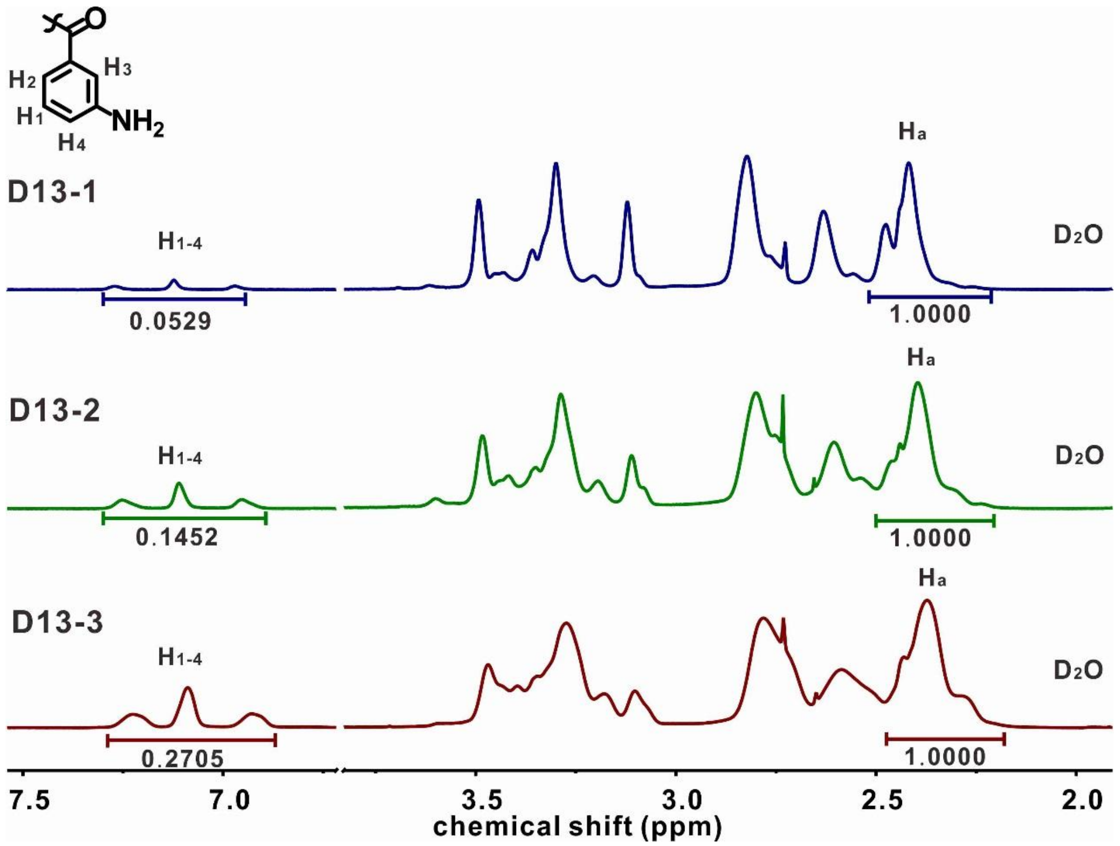

H2
H3
H1
NH2
H4
D13-1
H1-4
0.0529
Ha
1.0000
D2O
D13-2
H1-4
0.1452
Ha
1.0000
D2O
D13-3
H1-4
0.2705
Ha
1.0000
D2O
7.5
7.0
3.5
3.0
2.5
2.0
chemical shift (ppm)

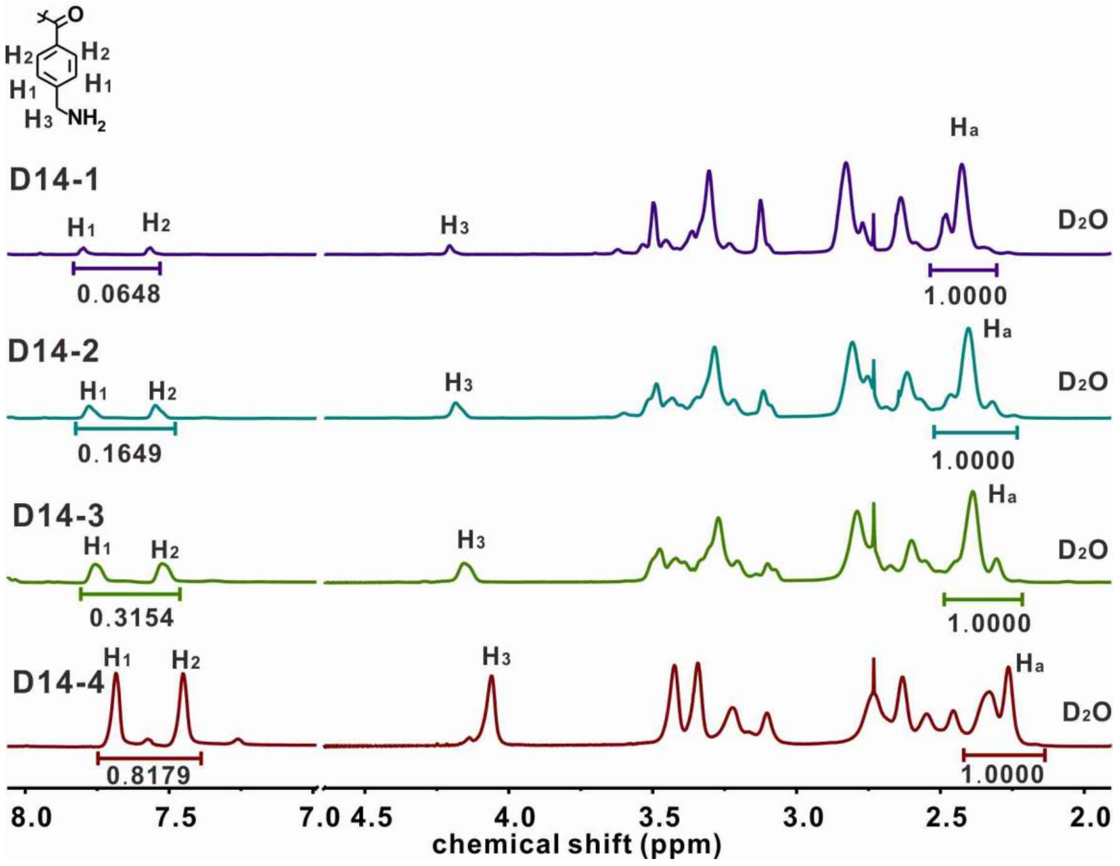

H2
H2
H1
H1
H3
NH2
D14-1
H1
H2
0.0648
H3
Ha
1.0000
D2O
D14-2
H1
H2
0.1649
H3
Ha
1.0000
D2O
D14-3
H1
H2
0.3154
H3
Ha
1.0000
D2O
D14-4
H1
H2
0.8179
H3
Ha
1.0000
D2O
8.0
7.5
7.0
4.5
4.0
3.5
3.0
2.5
2.0
chemical shift (ppm)

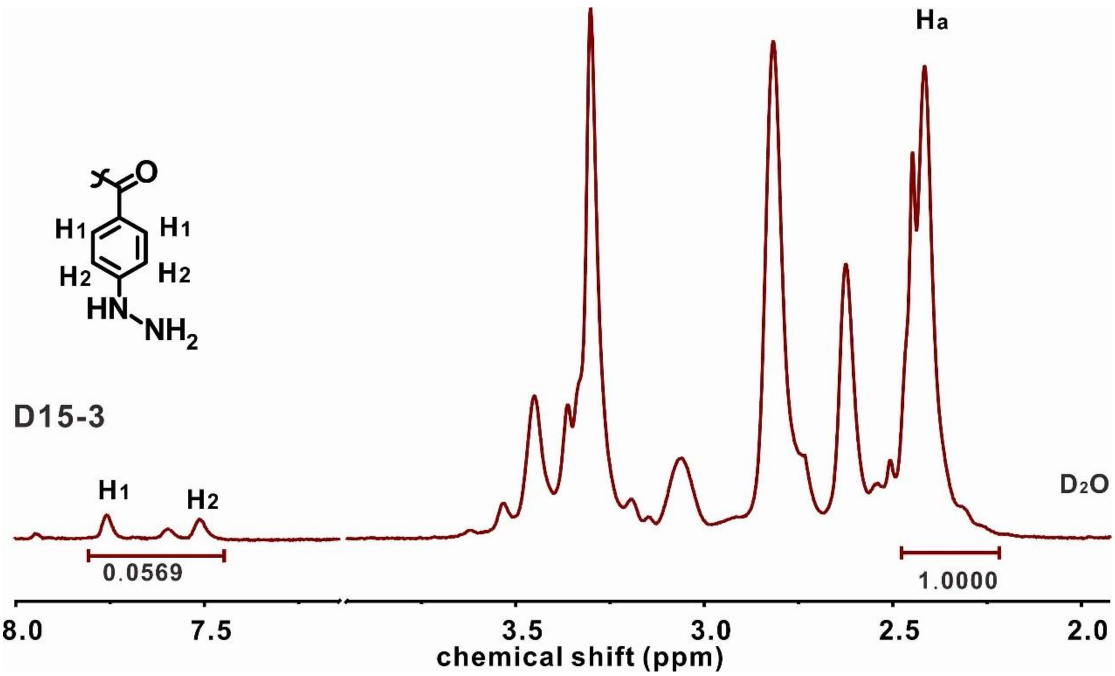
Ha
H1
H1
H2
H2
HN
NH2
D15-3
H1
H2
D2O
0.0569
1.0000
8.0
7.5
3.5
3.0
2.5
2.0
chemical shift (ppm)

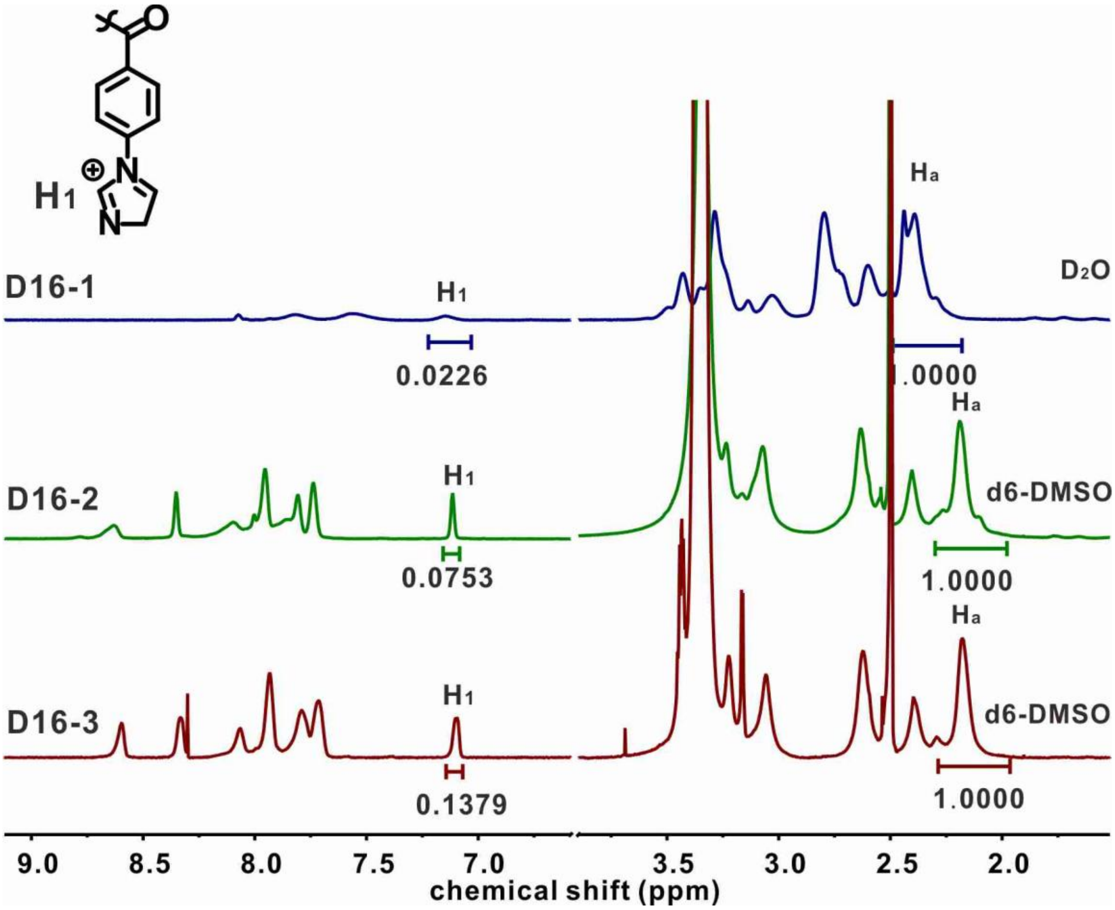
H1
N
N
Ha
D16-1
H1
D2O
0.0226
1.0000
Ha
D16-2
H1
d6-DMSO
0.0753
1.0000
Ha
D16-3
H1
d6-DMSO
0.1379
1.0000
9.0
8.5
8.0
7.5
7.0
3.5
3.0
2.5
2.0
chemical shift (ppm)

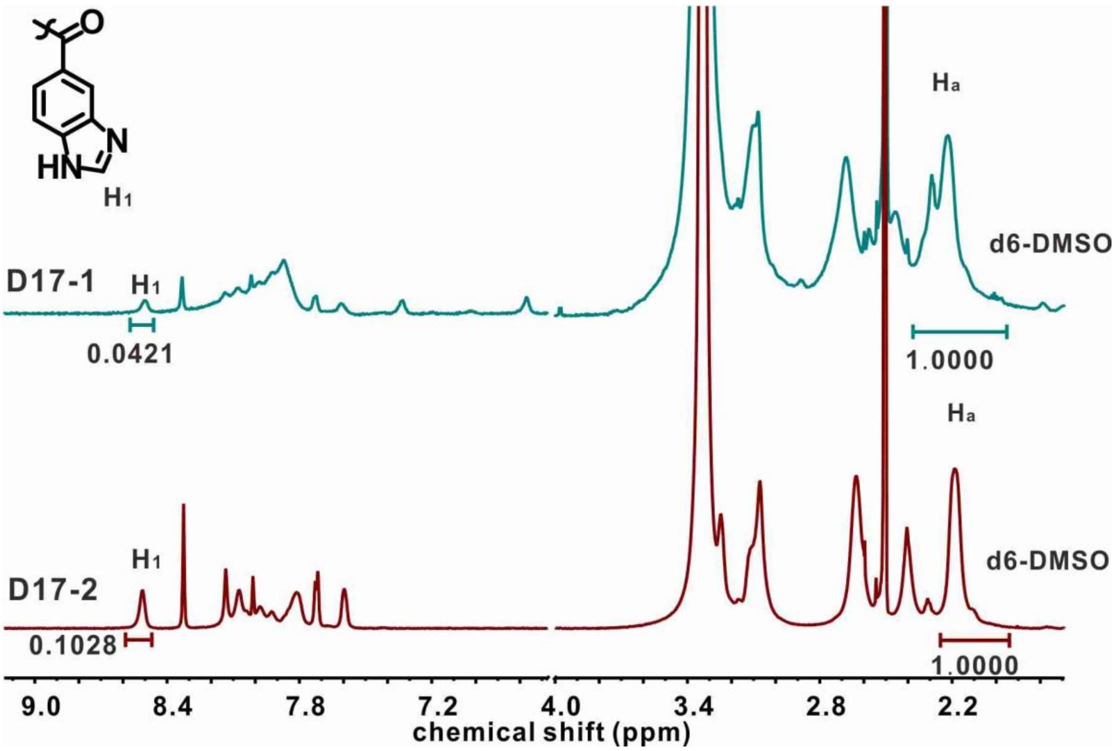
H1
Ha
D17-1
H1
d6-DMSO
0.0421
1.0000
Ha
D17-2
H1
d6-DMSO
0.1028
1.0000
9.0 8.4 7.8 7.2 4.0 3.4 2.8 2.2
chemical shift (ppm)

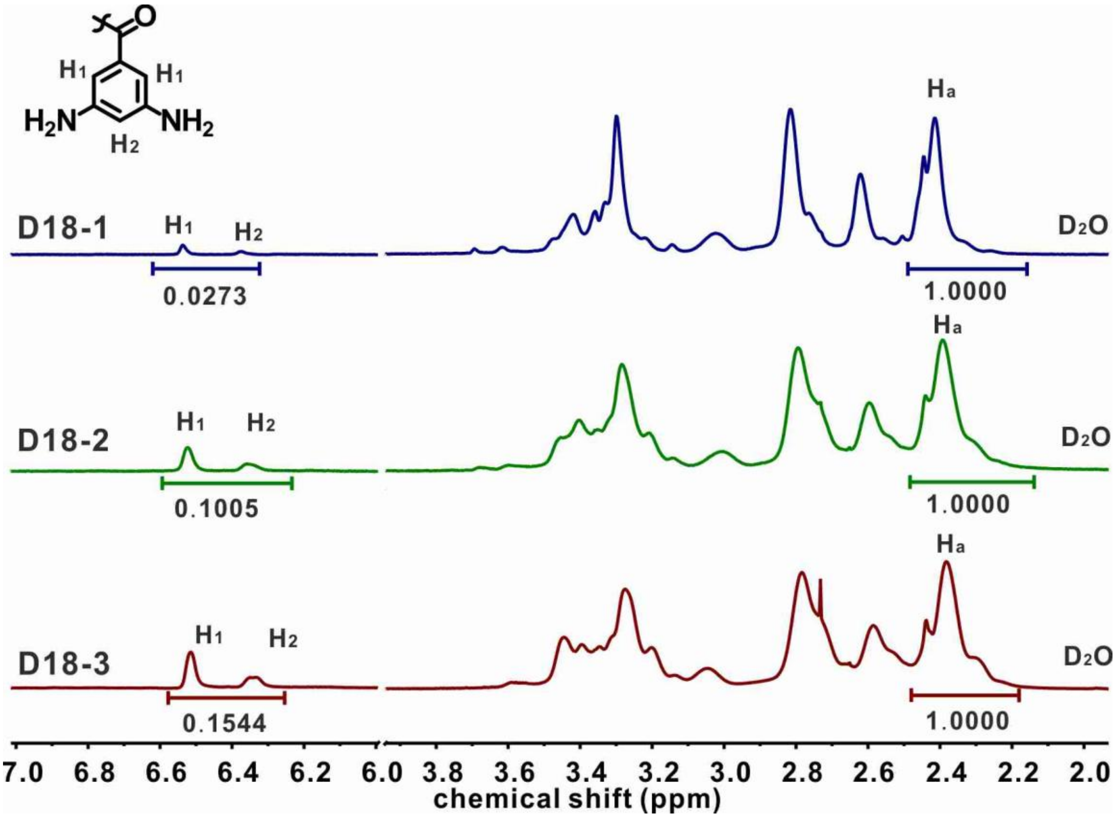
H1 H1
H2N NH2
H2
Ha
D18-1
H1 H2
D2O
0.0273
1.0000
Ha
D18-2
H1 H2
D2O
0.1005
1.0000
Ha
D18-3
H1 H2
D2O
0.1544
1.0000
7.0 6.8 6.6 6.4 6.2 6.0 3.8 3.6 3.4 3.2 3.0 2.8 2.6 2.4 2.2 2.0
chemical shift (ppm)

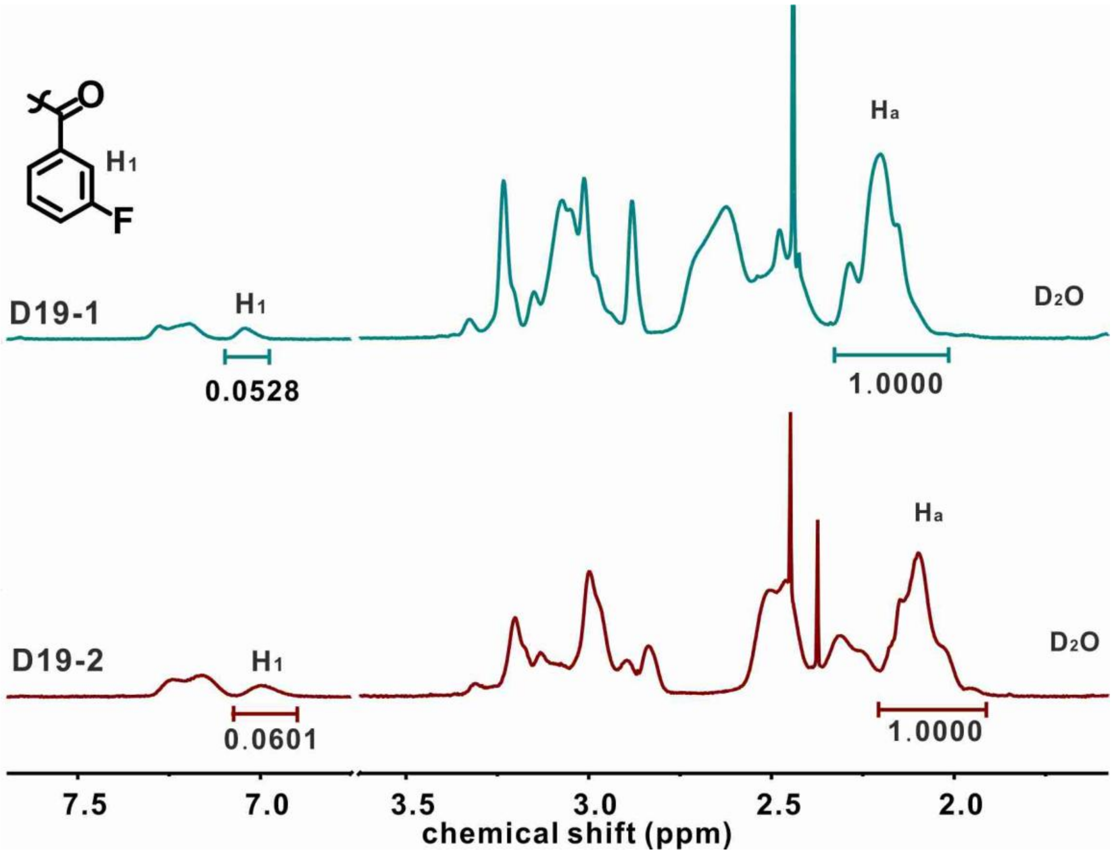
H1
F
D19-1
H1
0.0528
Ha
D2O
1.0000
D19-2
H1
0.0601
Ha
D2O
1.0000
7.5
7.0
3.5
3.0
2.5
2.0
chemical shift (ppm)

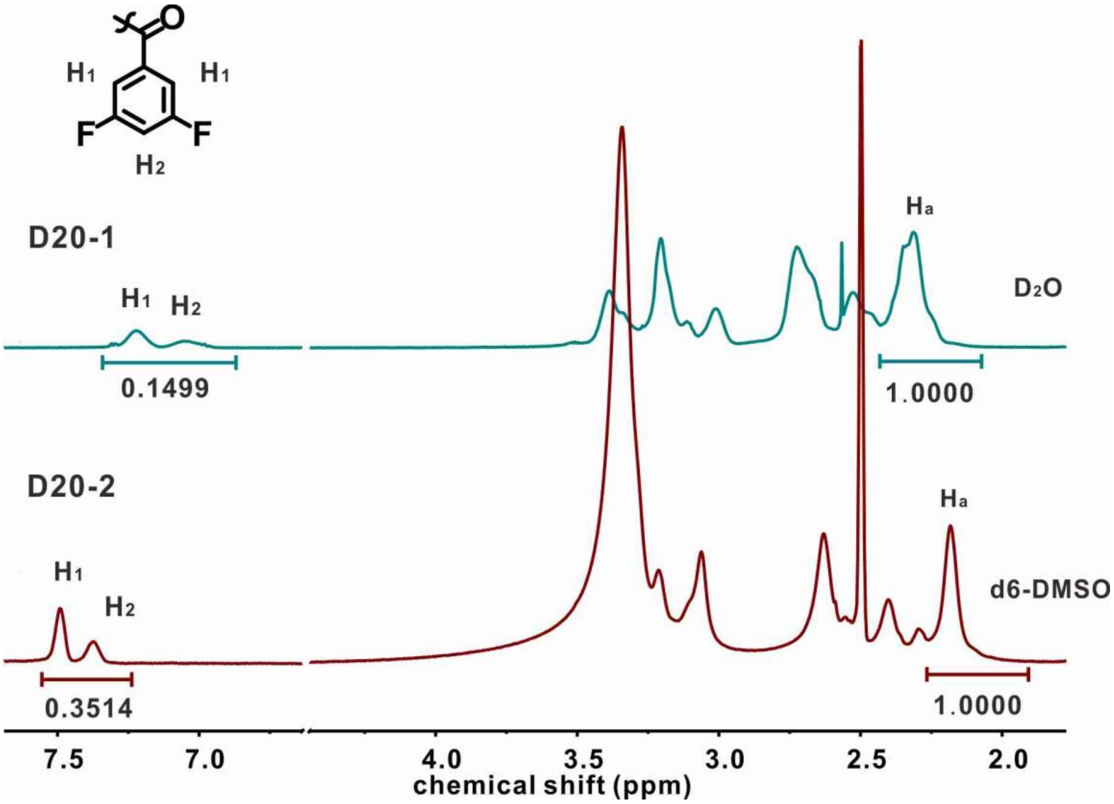
H1
H1
F
F
H2
D20-1
H1
H2
0.1499
Ha
D2O
1.0000
D20-2
H1
H2
0.3514
Ha
d6-DMSO
1.0000
7.5
7.0
4.0
3.5
3.0
2.5
2.0
chemical shift (ppm)

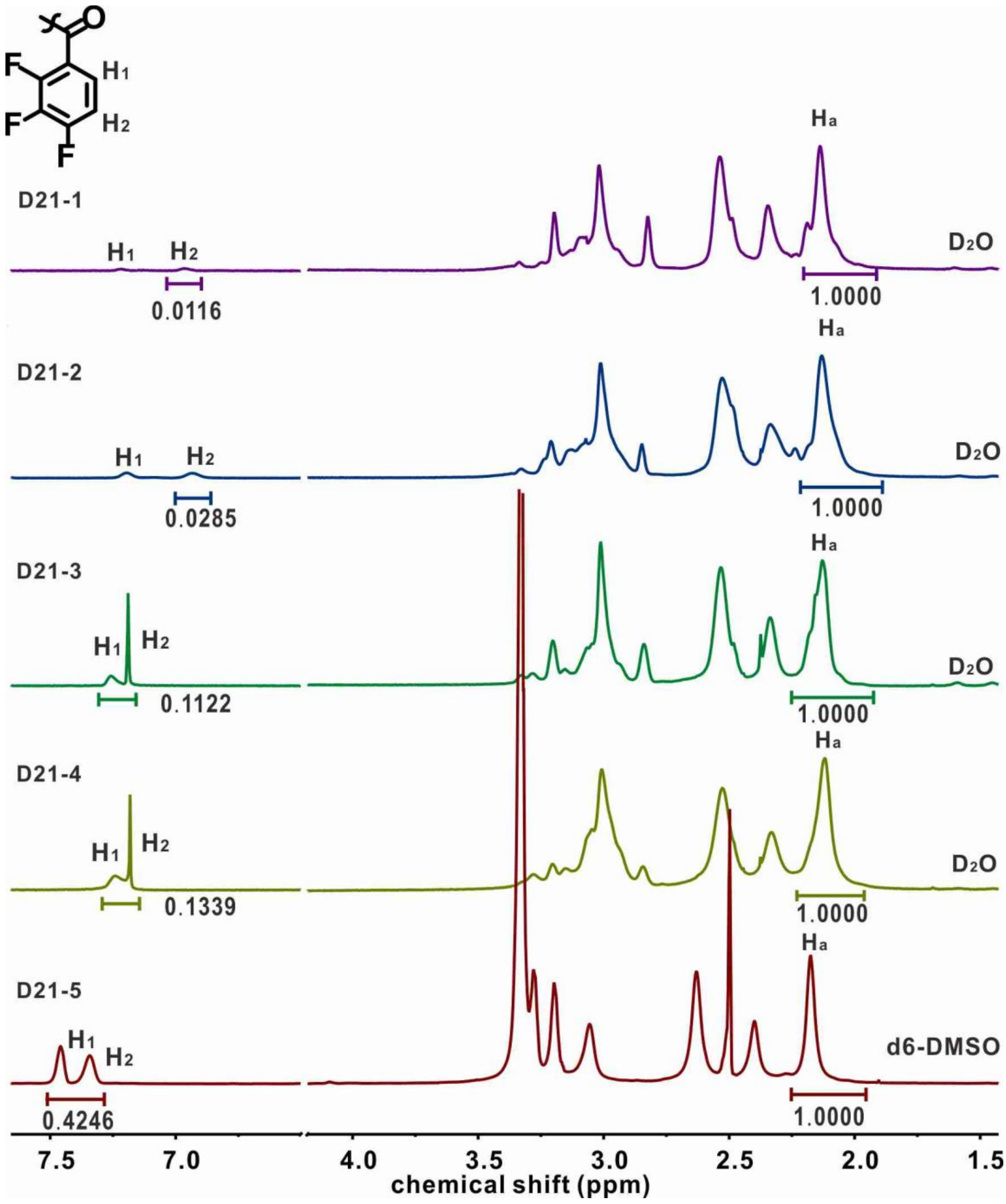

F
F
F
O
H1
H2
D21-1
H1
H2
0.0116
Ha
1.0000
D2O
D21-2
H1
H2
0.0285
Ha
1.0000
D2O
D21-3
H1
H2
0.1122
Ha
1.0000
D2O
D21-4
H1
H2
0.1339
Ha
1.0000
D2O
D21-5
H1
H2
0.4246
Ha
1.0000
d6-DMSO
7.5
7.0
4.0
3.5
3.0
2.5
2.0
1.5
chemical shift (ppm)

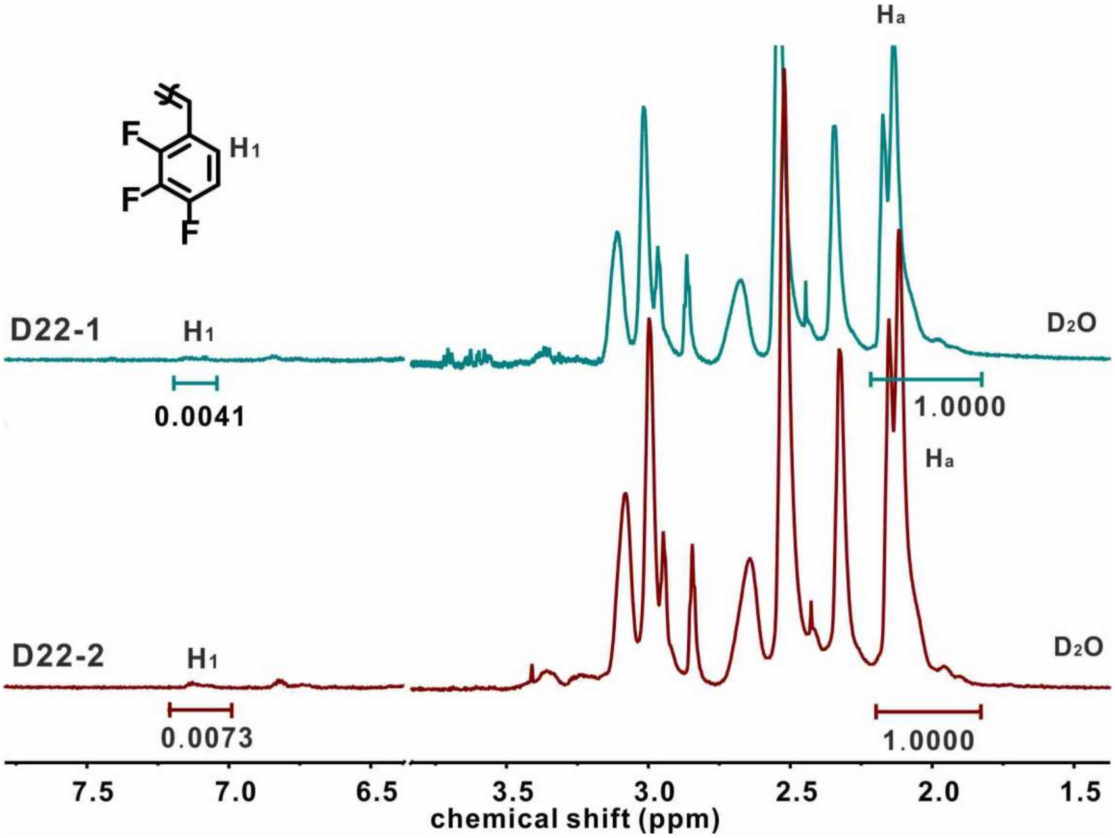

Ha
F
F
F
H1
D22-1
H1
D2O
0.0041
1.0000
Ha
D22-2
H1
D2O
0.0073
1.0000
7.5
7.0
6.5
3.5
3.0
2.5
2.0
1.5
chemical shift (ppm)

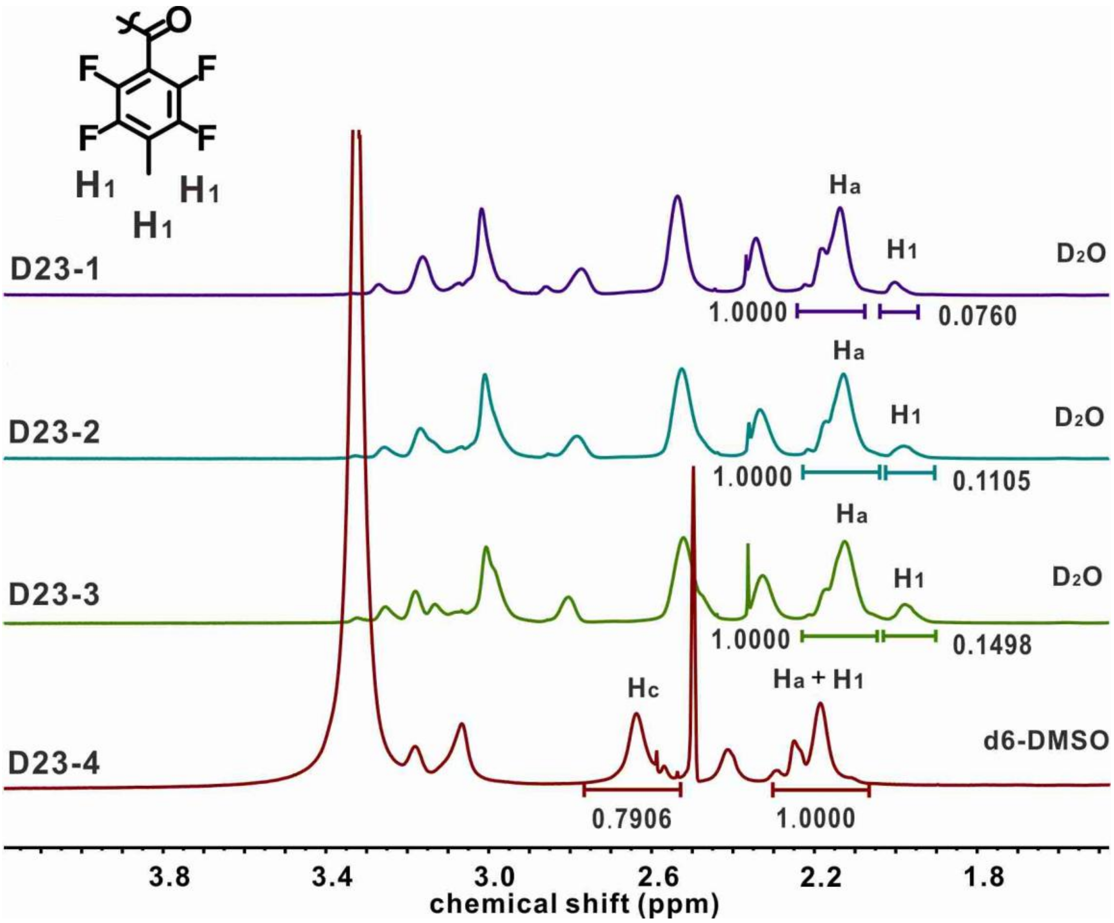

O
F
F
F
F
H1
H1
H1
Ha
D23-1
H1
D2O
1.0000
0.0760
Ha
D23-2
H1
D2O
1.0000
0.1105
Ha
D23-3
H1
D2O
1.0000
0.1498
Hc
Ha + H1
D23-4
d6-DMSO
0.7906
1.0000
3.8
3.4
3.0
2.6
2.2
1.8
chemical shift (ppm)

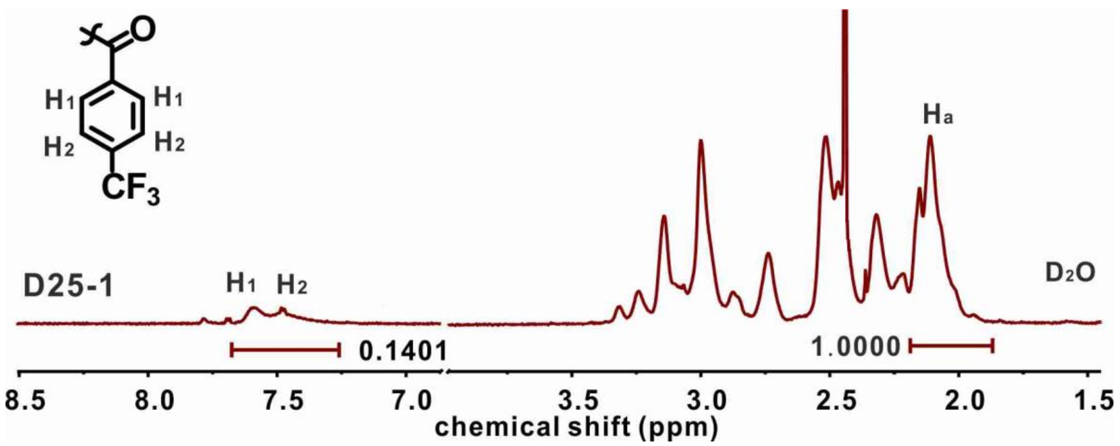

H1
H1
H2
H2
CF3
D25-1
H1 H2
Ha
D2O
0.1401
1.0000
8.5 8.0 7.5 7.0 3.5 3.0 2.5 2.0 1.5
chemical shift (ppm)

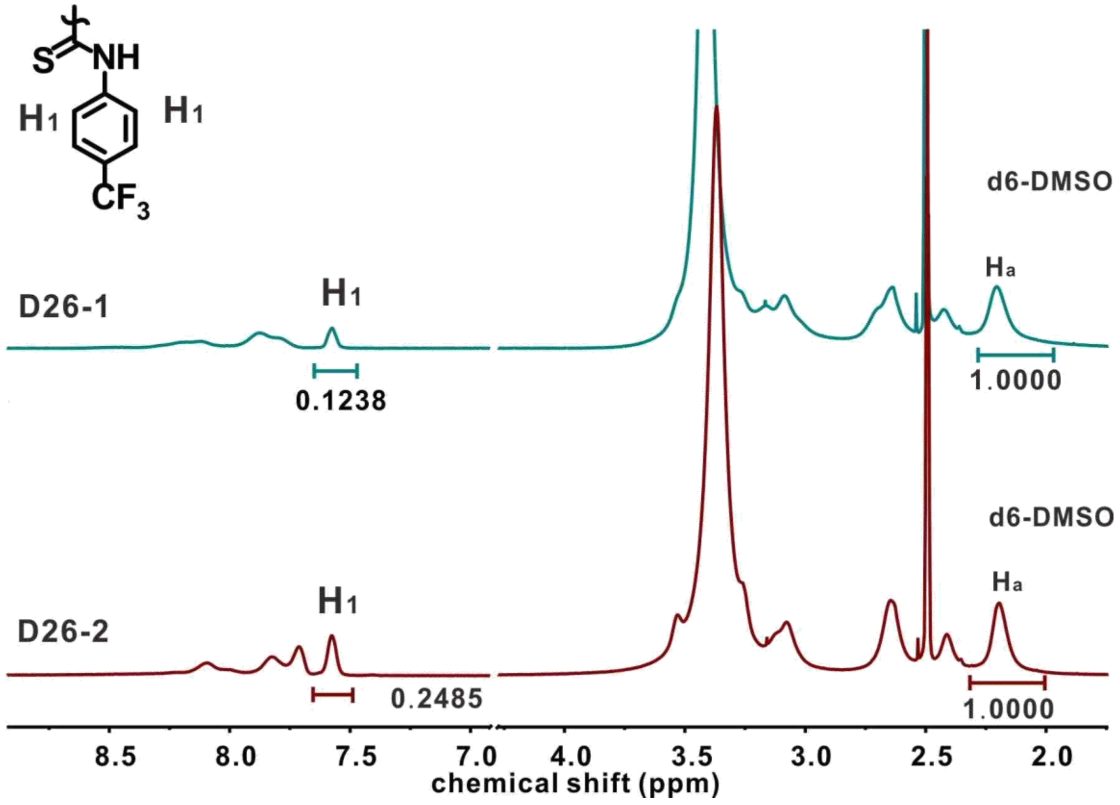

S
NH
H1
H1
CF3
d6-DMSO
D26-1
H1
Ha
0.1238
1.0000
d6-DMSO
D26-2
H1
Ha
0.2485
1.0000
8.5 8.0 7.5 7.0 4.0 3.5 3.0 2.5 2.0
chemical shift (ppm)

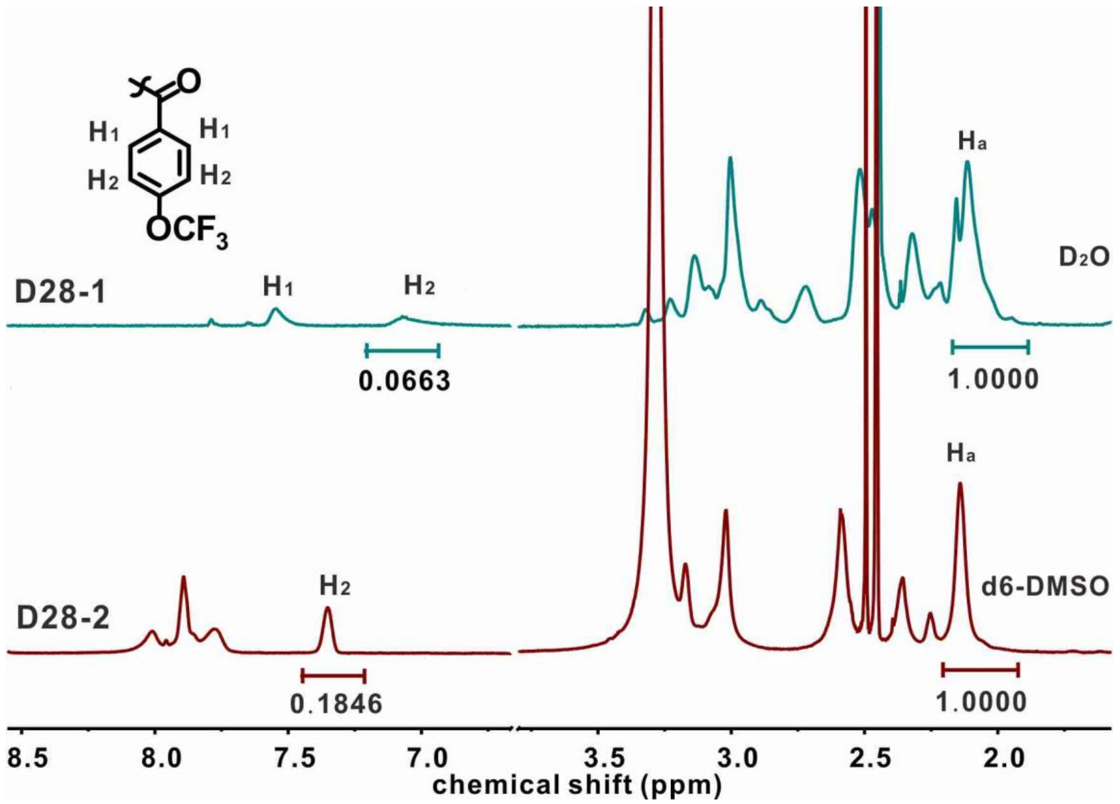

H1
H1
H2
H2
OCF3
D28-1
H1
H2
0.0663
Ha
D2O
1.0000
Ha
D28-2
H2
d6-DMSO
0.1846
1.0000
8.5 8.0 7.5 7.0 3.5 3.0 2.5 2.0
chemical shift (ppm)

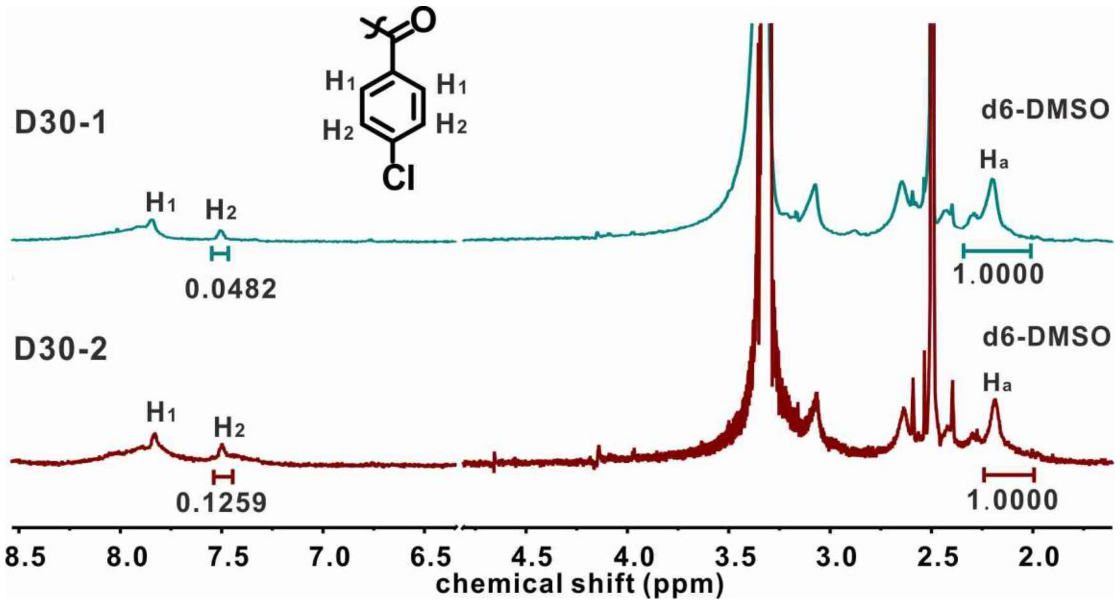

H1
H1
H2
H2
Cl
D30-1
d6-DMSO
H1
H2
Ha
0.0482
1.0000
D30-2
d6-DMSO
H1
H2
Ha
0.1259
1.0000
8.5 8.0 7.5 7.0 6.5 4.5 4.0 3.5 3.0 2.5 2.0
chemical shift (ppm)

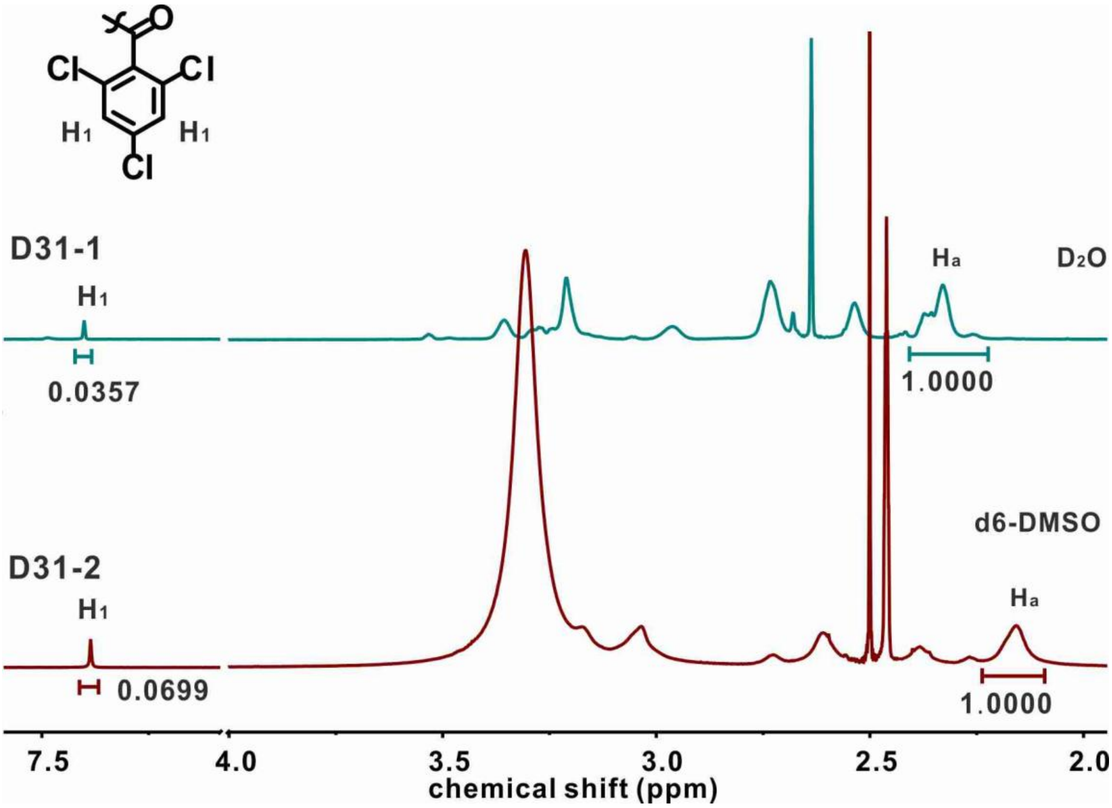

Cl
Cl
H1
H1
Cl
D31-1
H1
0.0357
Ha
D2O
1.0000
D31-2
H1
0.0699
d6-DMSO
Ha
1.0000
7.5
4.0
3.5
3.0
2.5
2.0
chemical shift (ppm)

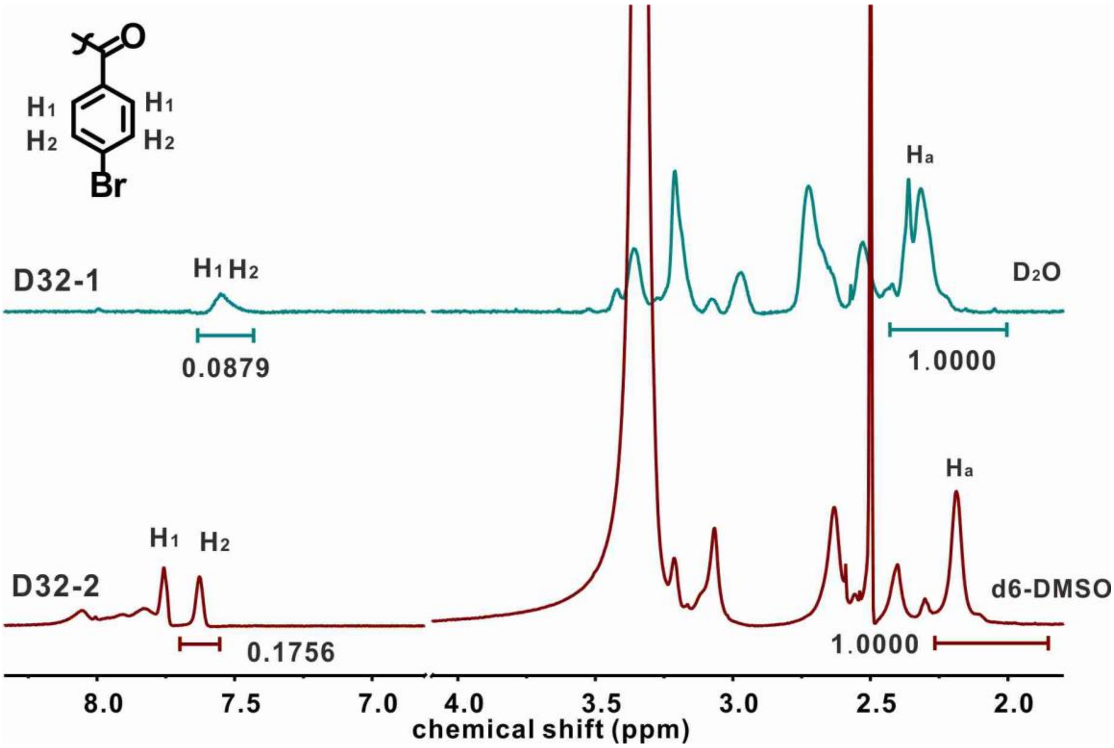

H1
H2
H1
H2
Br
D32-1
H1 H2
0.0879
Ha
D2O
1.0000
D32-2
H1
H2
0.1756
Ha
d6-DMSO
1.0000
8.0
7.5
7.0
4.0
3.5
3.0
2.5
2.0
chemical shift (ppm)

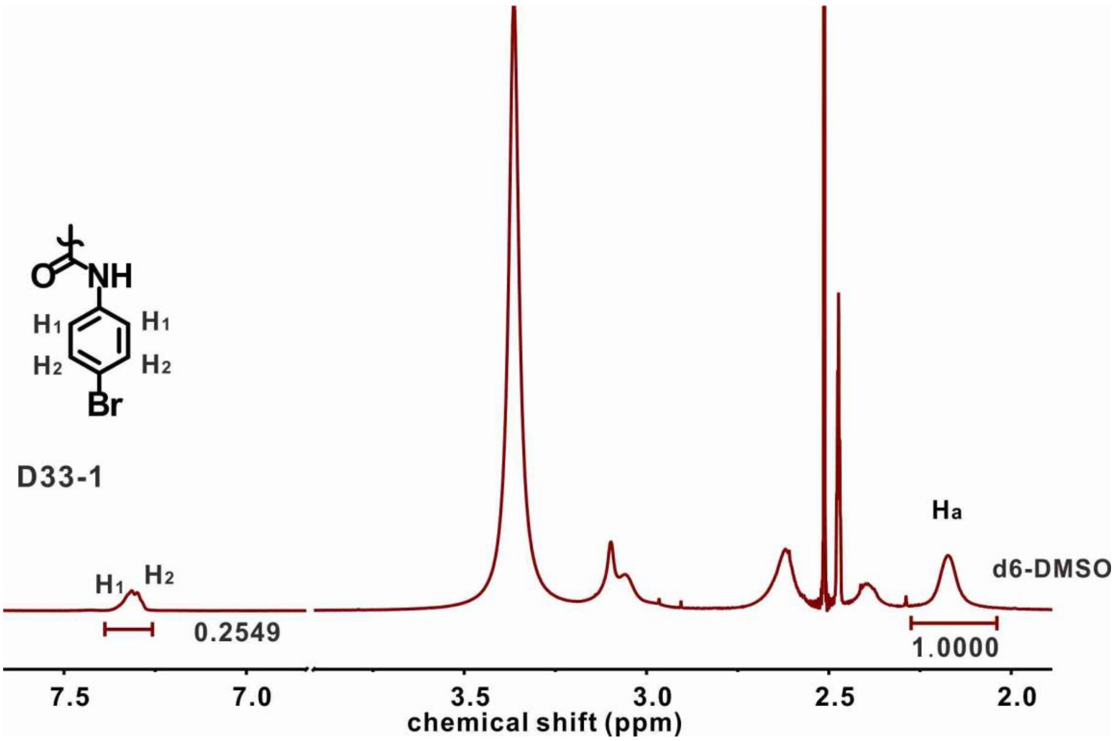
NH
O
H1
H1
H2
H2
Br
D33-1
H1
H2
0.2549
Ha
d6-DMSO
1.0000
7.5
7.0
3.5
3.0
2.5
2.0
chemical shift (ppm)

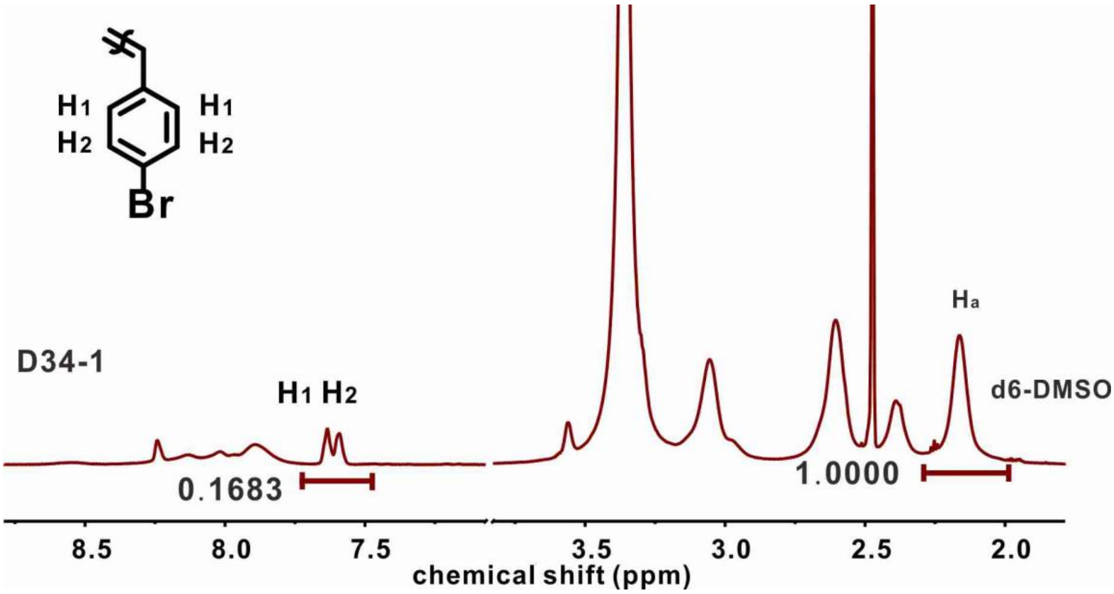
H1
H1
H2
H2
Br
D34-1
H1 H2
0.1683
Ha
d6-DMSO
1.0000
8.5
8.0
7.5
3.5
3.0
2.5
2.0
chemical shift (ppm)

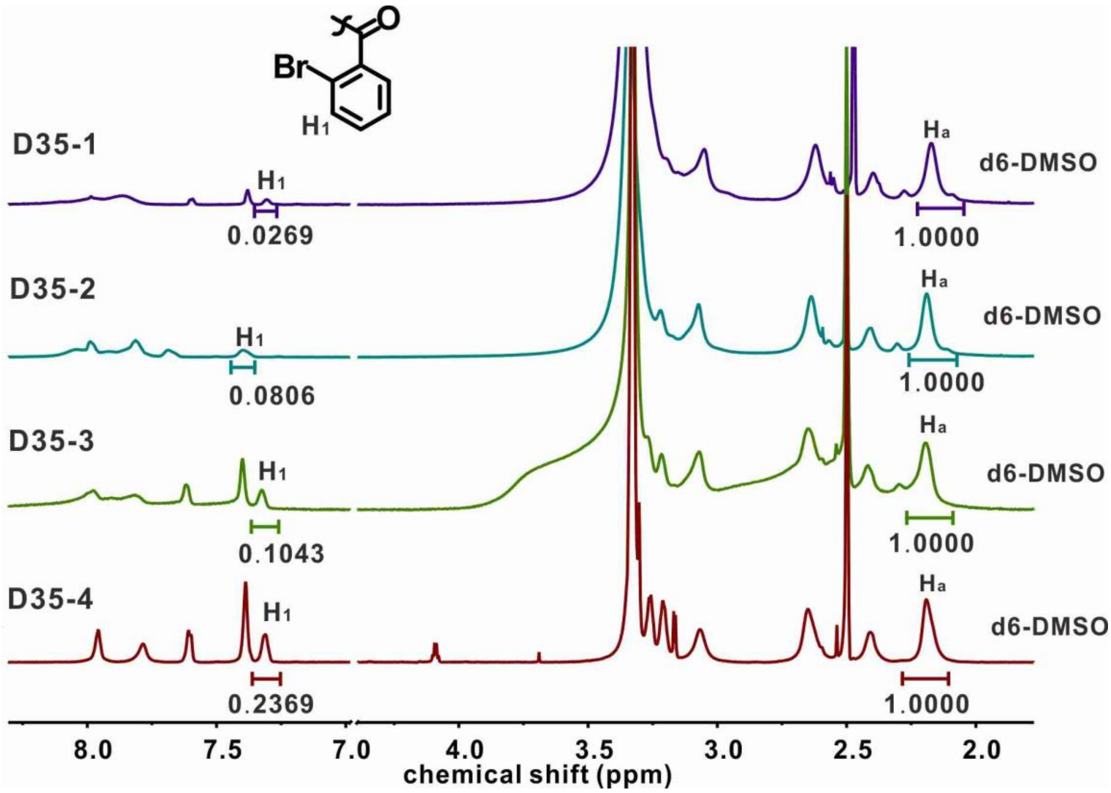

Br
H1
D35-1
D35-2
D35-3
D35-4
H1
Ha
d6-DMSO
0.0269
0.0806
0.1043
0.2369
1.0000
8.0
7.5
7.0
4.0
3.5
3.0
2.5
2.0
chemical shift (ppm)

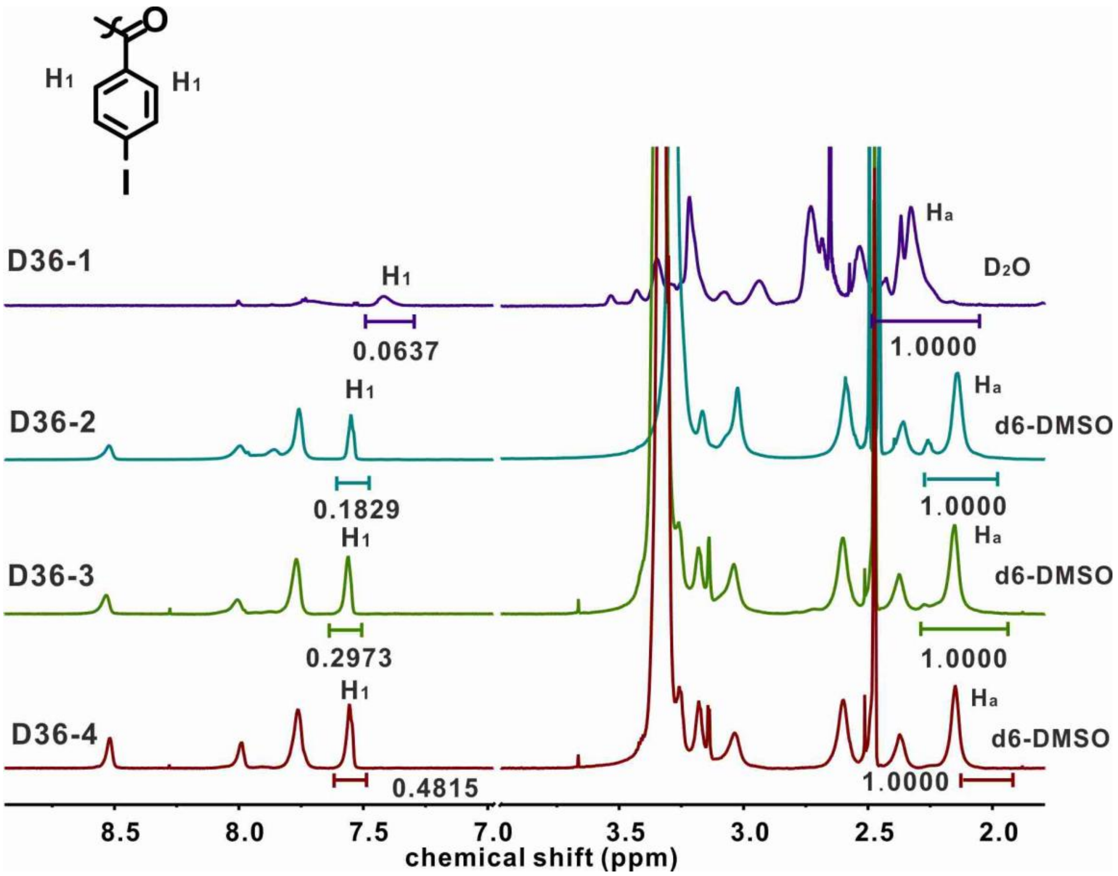

H1
I
D36-1
D36-2
D36-3
D36-4
Ha
D2O
d6-DMSO
0.0637
0.1829
0.2973
0.4815
1.0000
8.5
8.0
7.5
7.0
3.5
3.0
2.5
2.0
chemical shift (ppm)

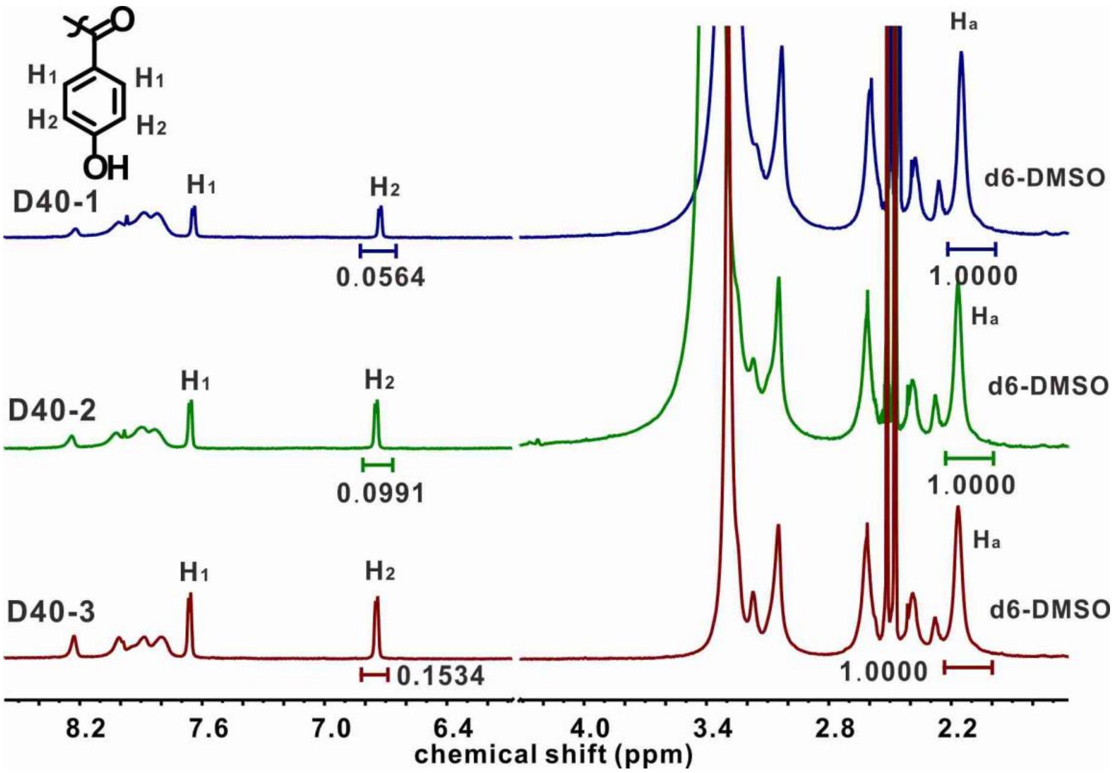
H1 H1
H2 H2
OH
D40-1
H1
H2
0.0564
Ha
d6-DMSO
1.0000
D40-2
H1
H2
0.0991
Ha
d6-DMSO
1.0000
D40-3
H1
H2
0.1534
Ha
d6-DMSO
1.0000
8.2 7.6 7.0 6.4 4.0 3.4 2.8 2.2
chemical shift (ppm)

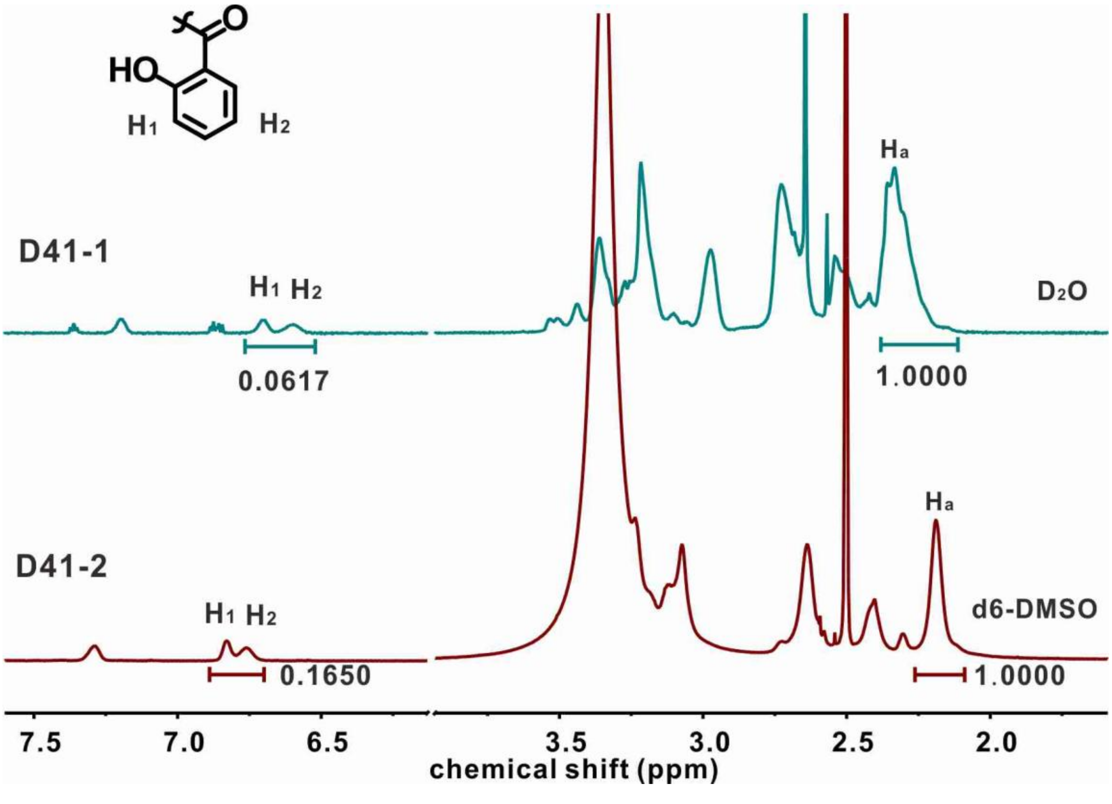
HO
H1 H2
D41-1
H1 H2
0.0617
Ha
D2O
1.0000
D41-2
H1 H2
0.1650
Ha
d6-DMSO
1.0000
7.5 7.0 6.5 3.5 3.0 2.5 2.0
chemical shift (ppm)

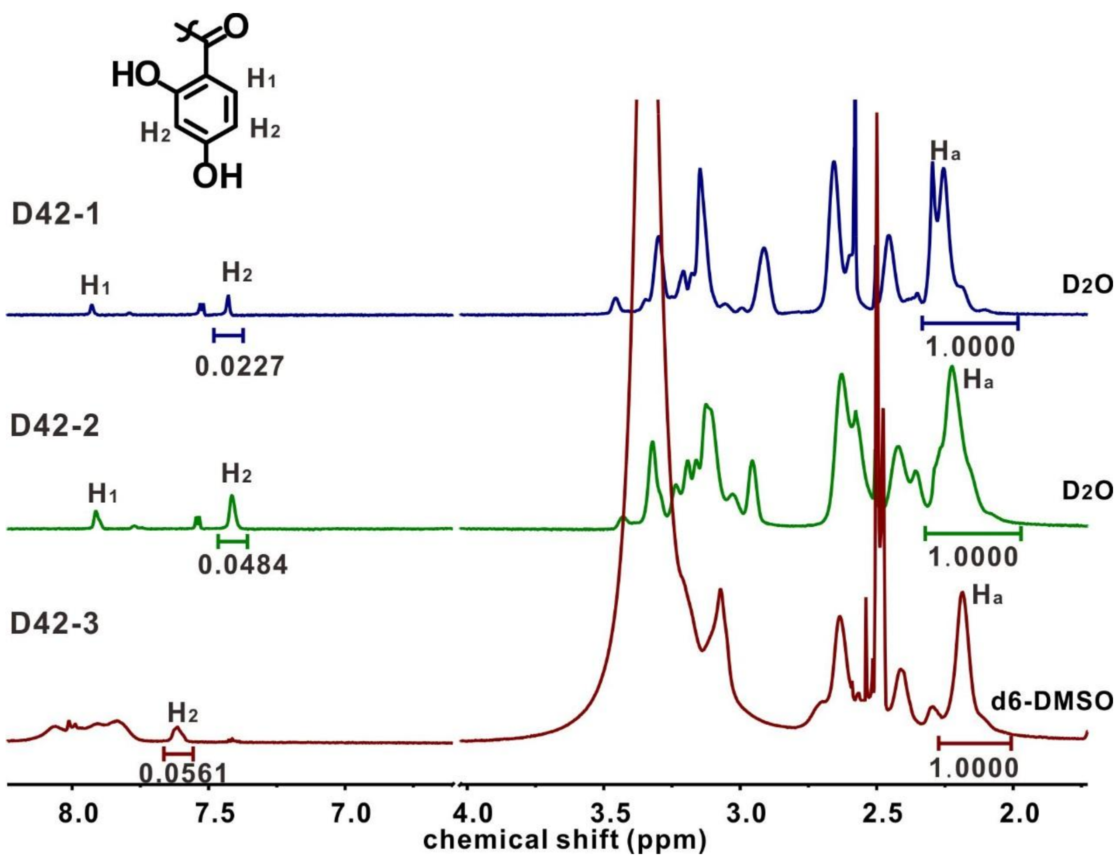
HO
OH
H1
H2
H2
D42-1
D42-2
D42-3
H1
H2
Ha
D2O
0.0227
1.0000
0.0484
d6-DMSO
0.0561
8.0
7.5
7.0
4.0
3.5
3.0
2.5
2.0
chemical shift (ppm)

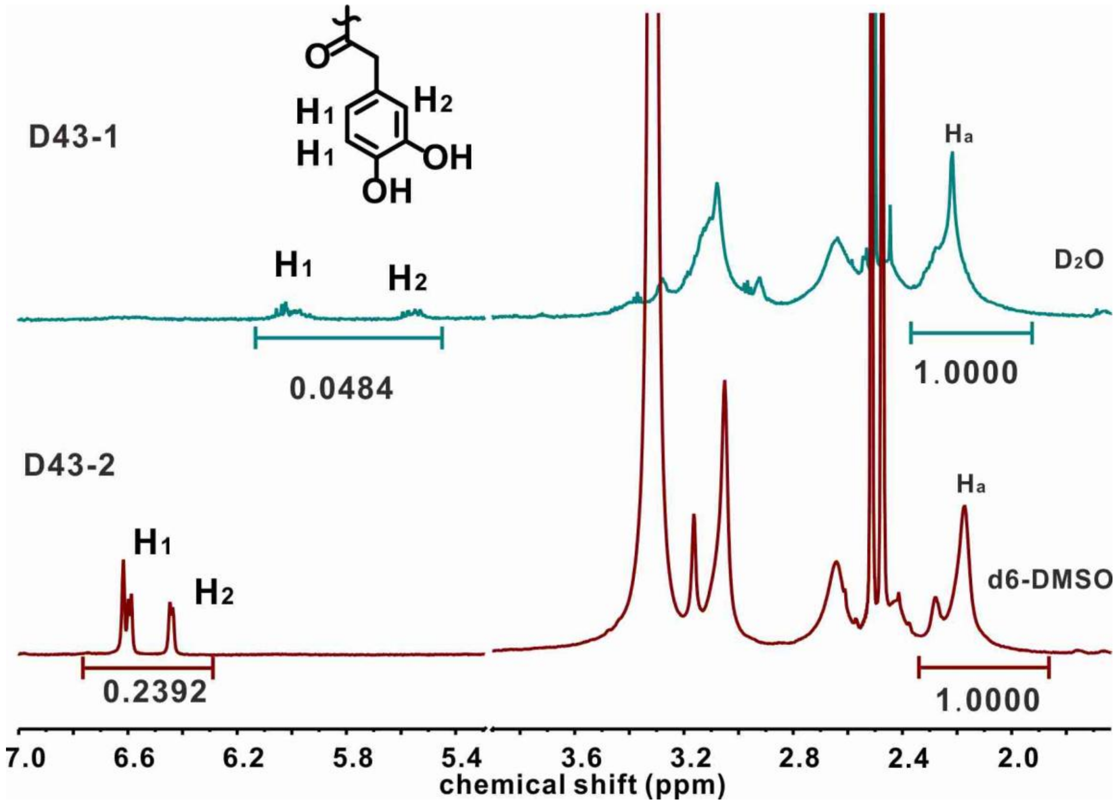
D43-1
D43-2
H1
H2
OH
Ha
D2O
d6-DMSO
0.0484
1.0000
0.2392
7.0
6.6
6.2
5.8
5.4
3.6
3.2
2.8
2.4
2.0
chemical shift (ppm)

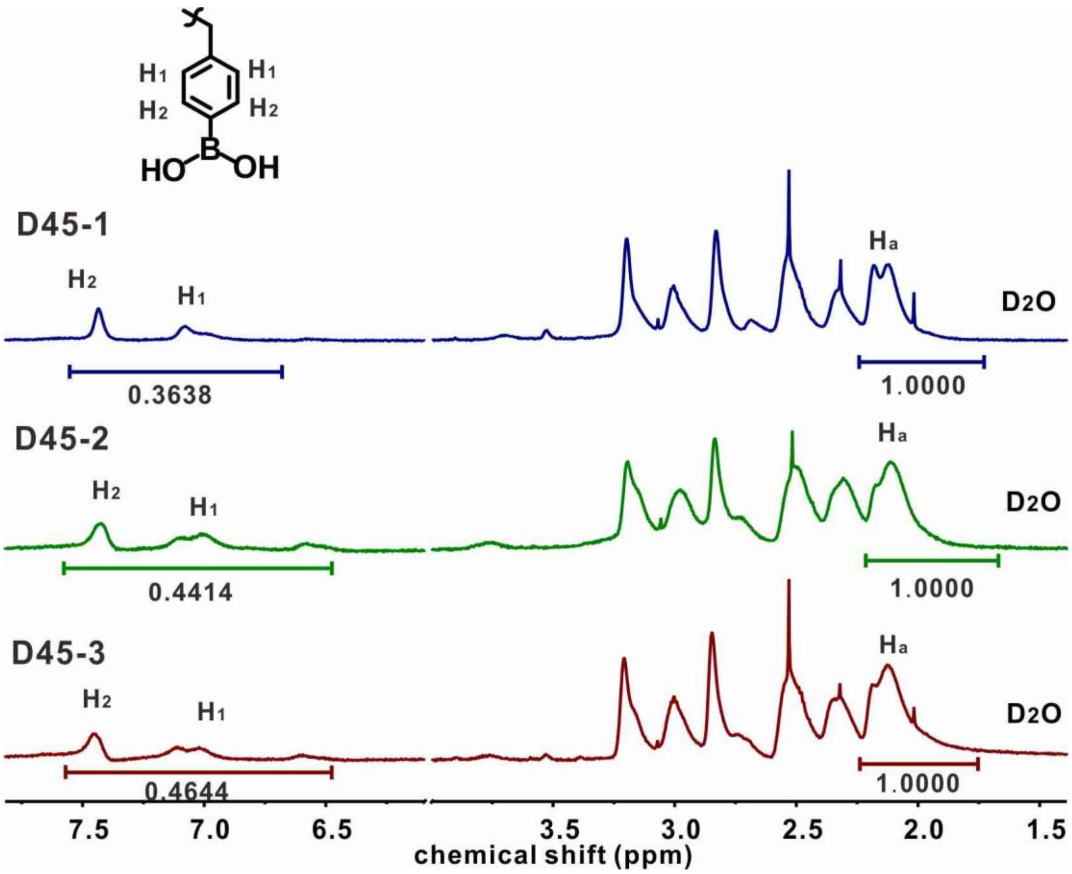
H1 H1
H2 H2
HO B OH
D45-1
H2 H1 Ha
D2O
0.3638 1.0000
D45-2
H2 H1 Ha
D2O
0.4414 1.0000
D45-3
H2 H1 Ha
D2O
0.4644 1.0000
7.5 7.0 6.5 3.5 3.0 2.5 2.0 1.5
chemical shift (ppm)

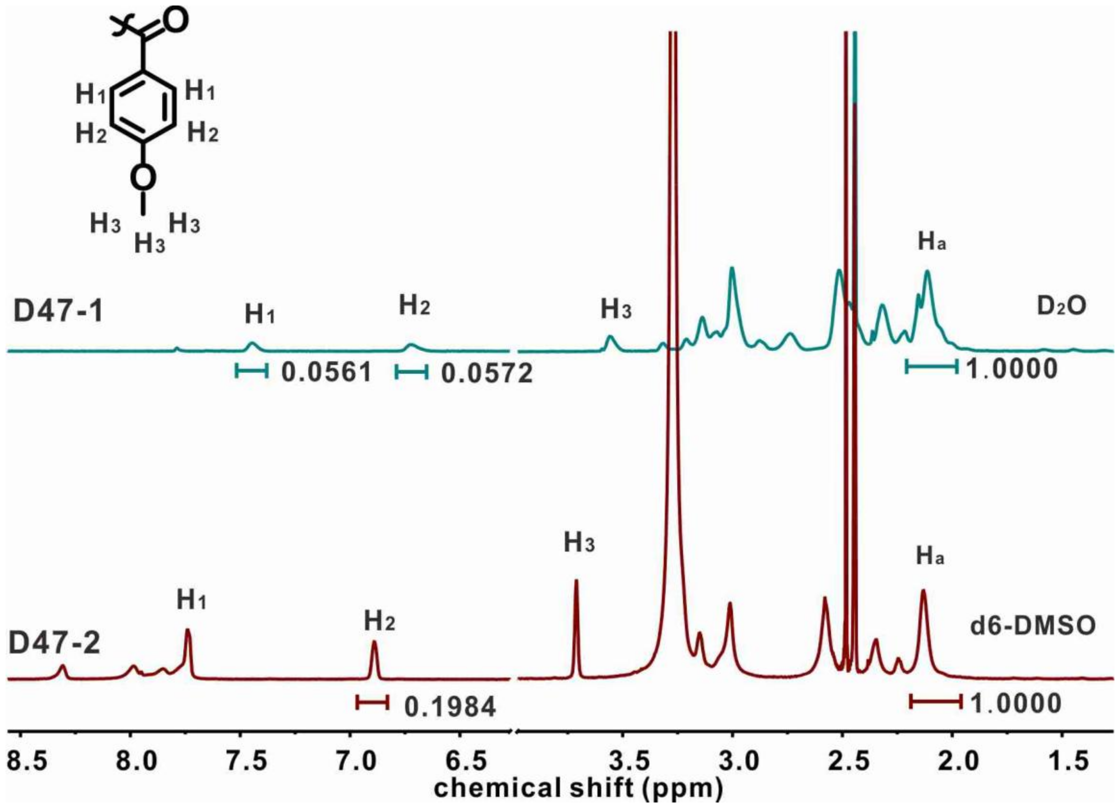
O
H1 H1
H2 H2
O
H3 H3 H3
D47-1
H1 H2 H3 Ha
D2O
0.0561 0.0572 1.0000
D47-2
H1 H2 H3 Ha
d6-DMSO
0.1984 1.0000
8.5 8.0 7.5 7.0 6.5 3.5 3.0 2.5 2.0 1.5
chemical shift (ppm)

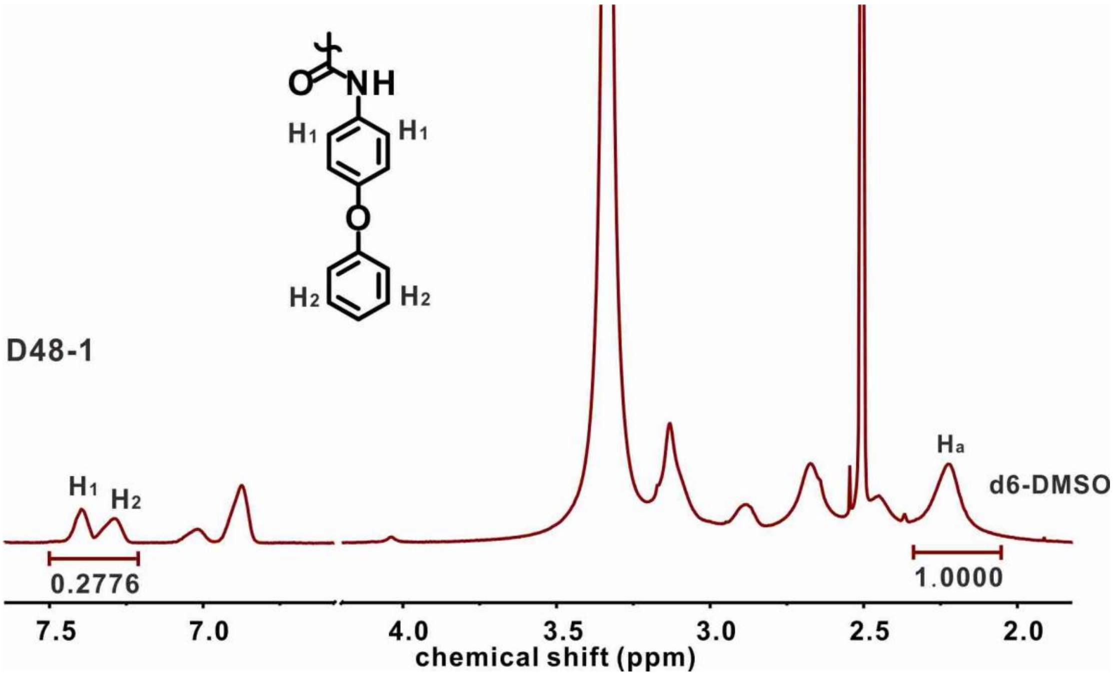

NH
O
H1
H1
O
H2
H2
D48-1
H1
H2
0.2776
Ha
d6-DMSO
1.0000
7.5
7.0
4.0
3.5
3.0
2.5
2.0
chemical shift (ppm)

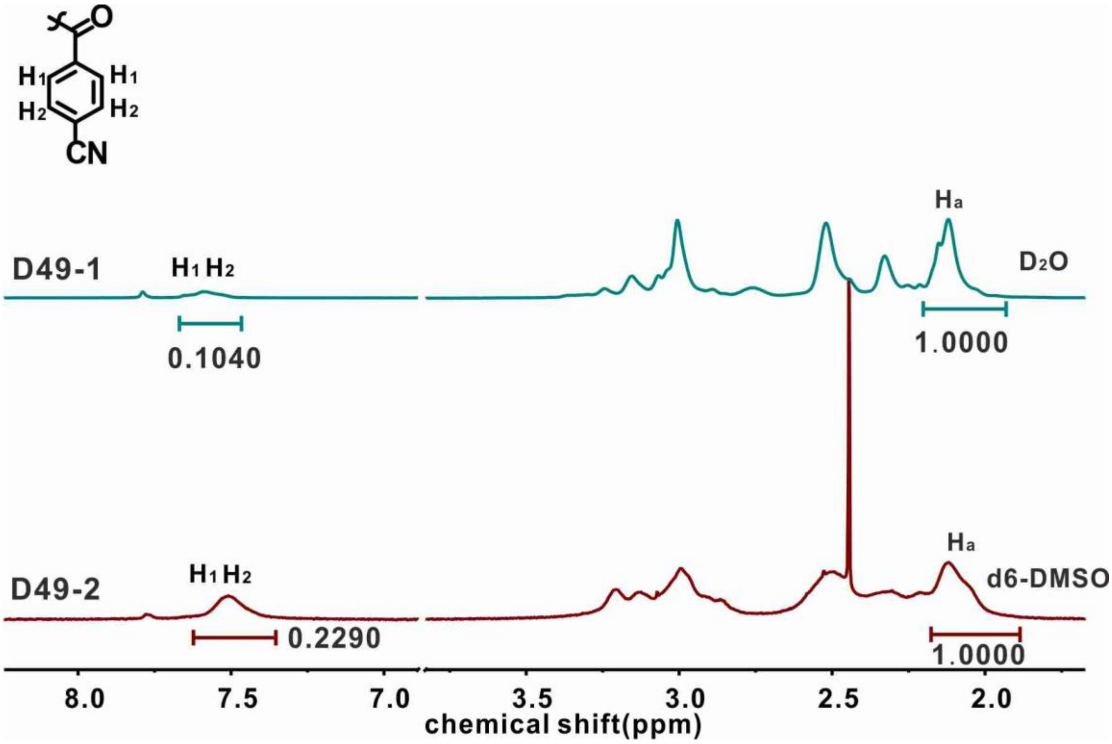

O
H1
H1
H2
H2
CN
D49-1
H1 H2
0.1040
Ha
D2O
1.0000
D49-2
H1 H2
0.2290
Ha
d6-DMSO
1.0000
8.0
7.5
7.0
3.5
3.0
2.5
2.0
chemical shift(ppm)

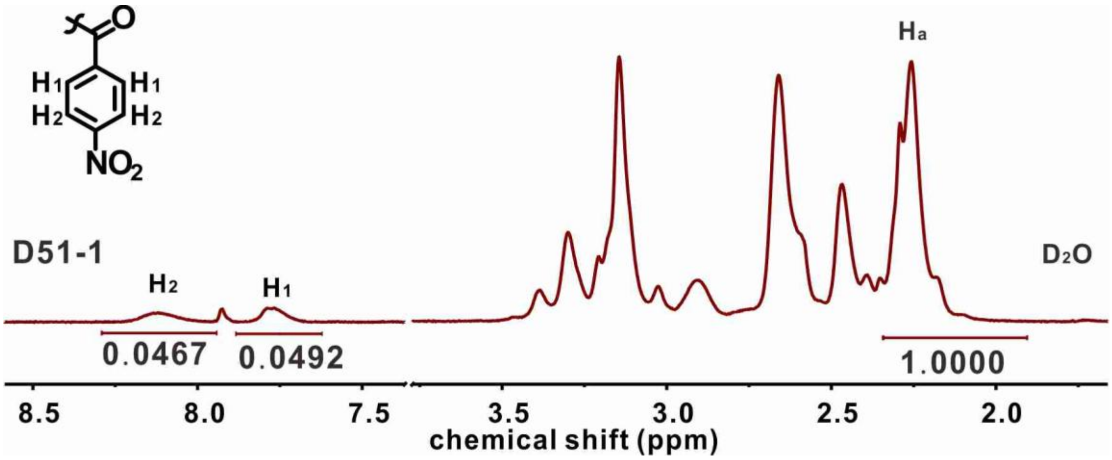

D51-1
H1 H1
H2 H2
NO2
Ha
H2
H1
D2O
0.0467
0.0492
1.0000
8.5 8.0 7.5 3.5 3.0 2.5 2.0
chemical shift (ppm)

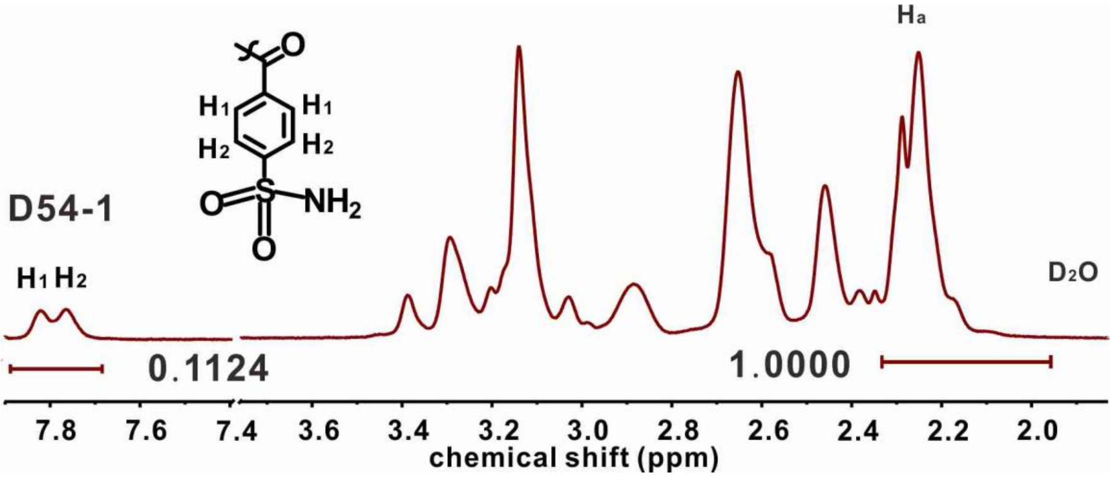

D54-1
H1 H1
H2 H2
NH2
Ha
H1 H2
D2O
0.1124
1.0000
7.8 7.6 7.4 3.6 3.4 3.2 3.0 2.8 2.6 2.4 2.2 2.0
chemical shift (ppm)

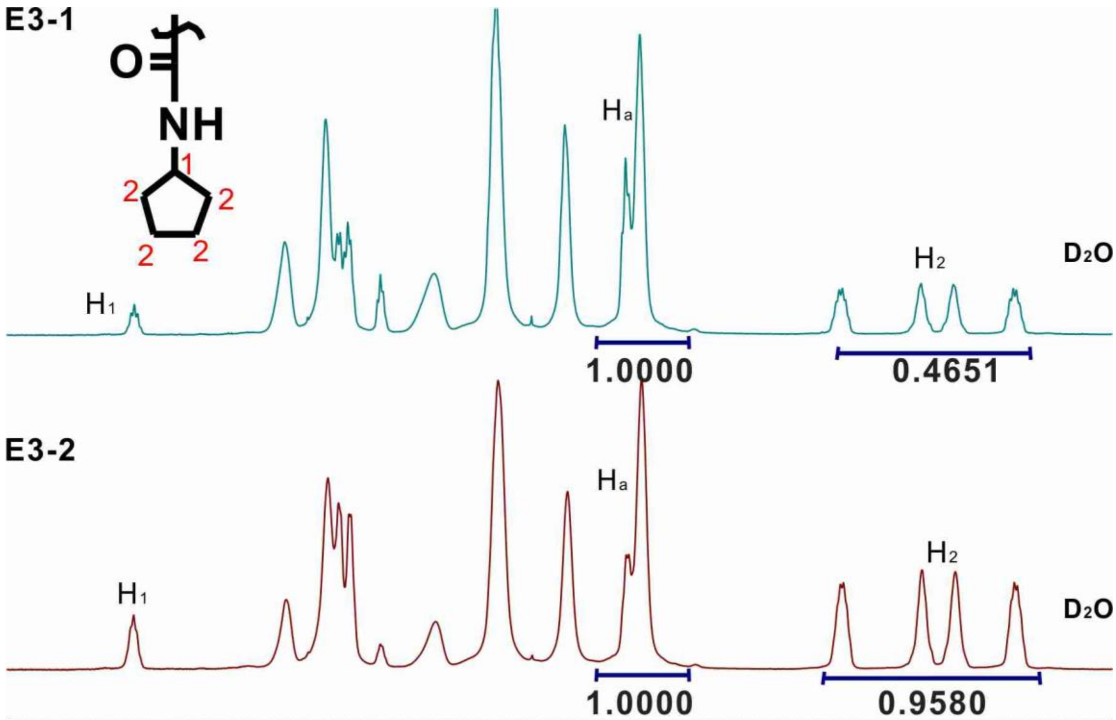

E3-1
O
NH
1
2 2
2 2
Ha
H1
H2
D2O
1.0000
0.4651
E3-2
Ha
H1
H2
D2O
1.0000
0.9580
3.9 3.6 3.3 3.0 2.7 2.4 2.1 1.8 1.5 1.2
Chemical shift (ppm)

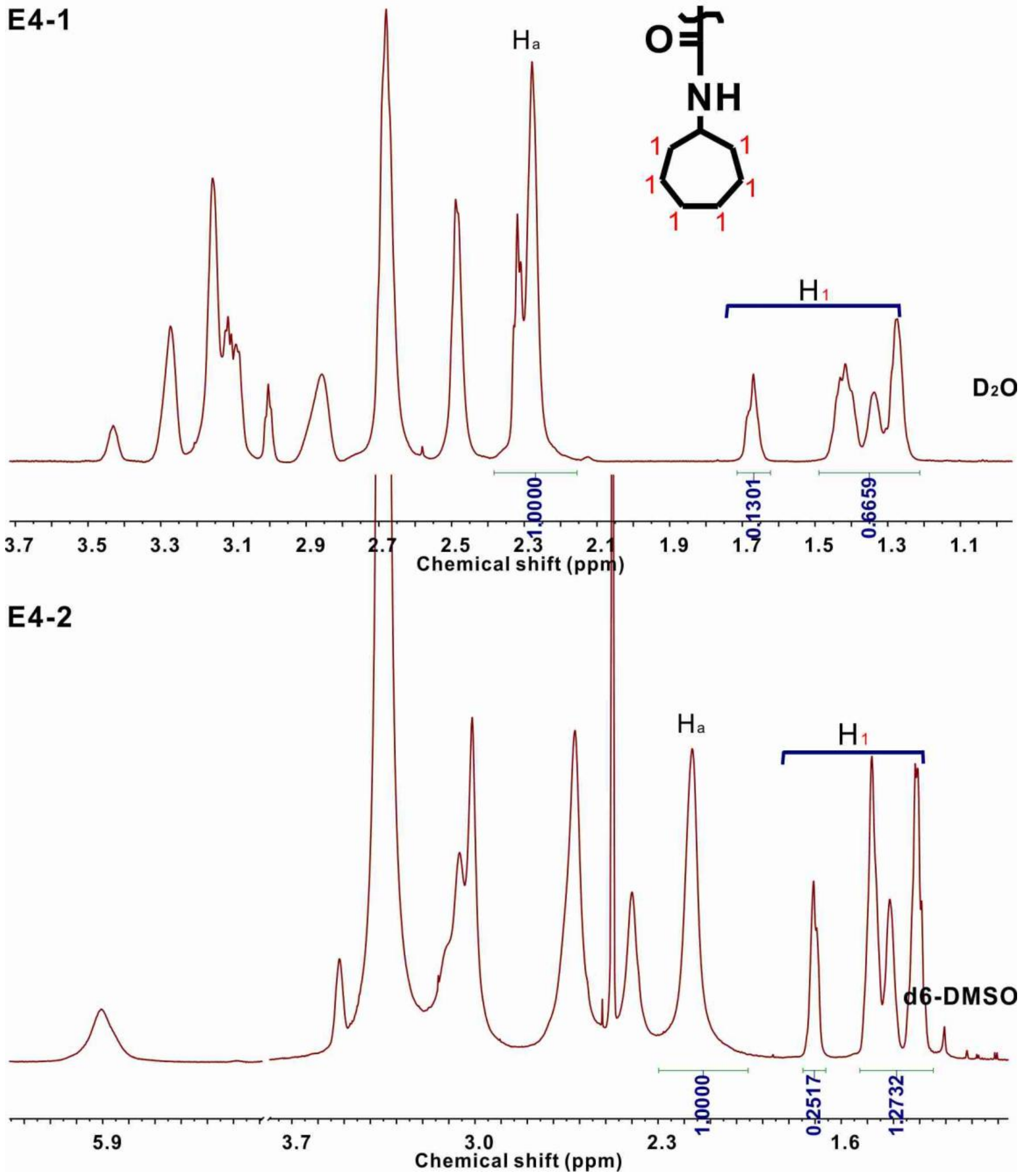
E4-1
Ha
O
NH
H1
D2O
1.0000
0.1301
0.6659
3.7 3.5 3.3 3.1 2.9 2.7 2.5 2.3 2.1 1.9 1.7 1.5 1.3 1.1
Chemical shift (ppm)
E4-2
Ha
H1
d6-DMSO
1.0000
0.2517
1.2732
5.9 3.7 3.0 2.3 1.6
Chemical shift (ppm)

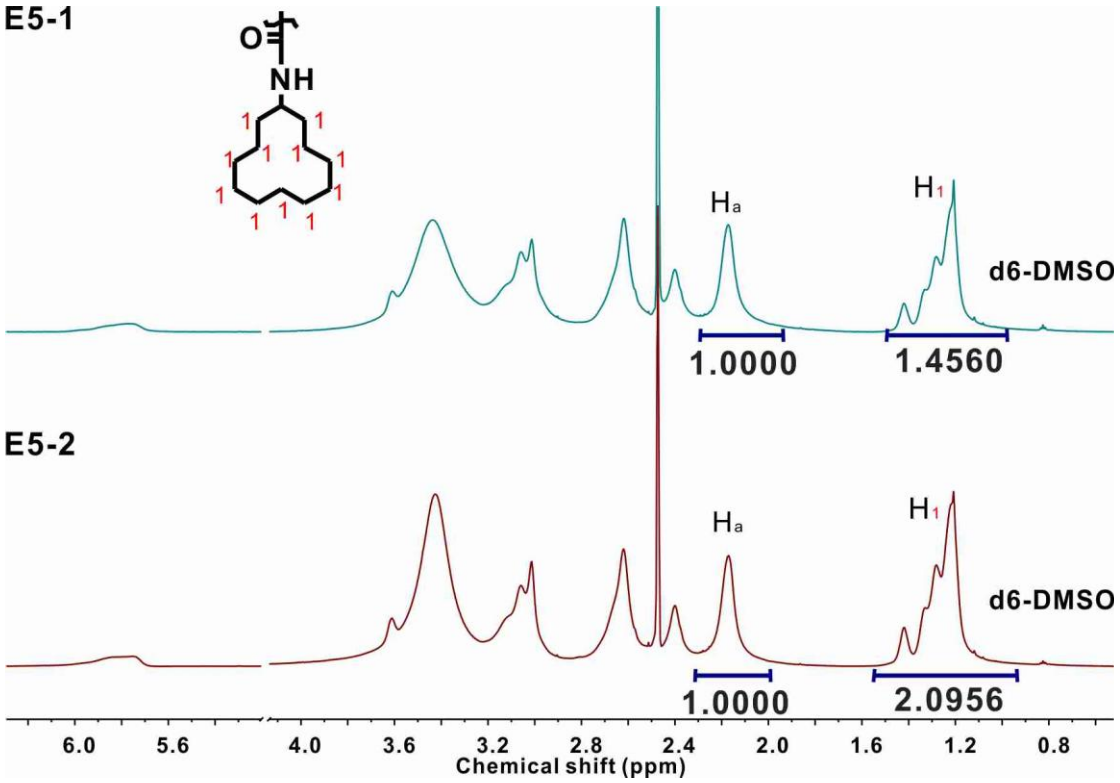

E5-1
O
NH
Ha
H1
d6-DMSO
1.0000
1.4560
E5-2
Ha
H1
d6-DMSO
1.0000
2.0956
6.0
5.6
4.0
3.6
3.2
2.8
2.4
2.0
1.6
1.2
0.8
Chemical shift (ppm)

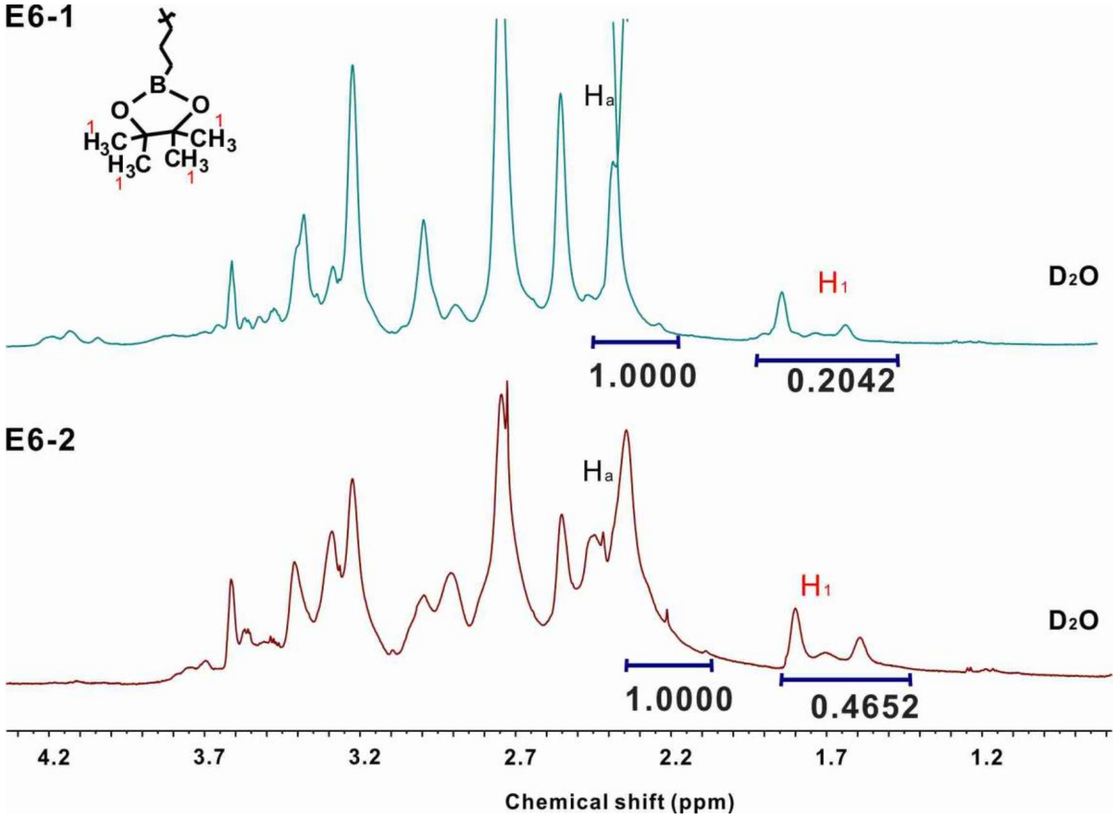

E6-1
B
O
O
H3C
CH3
H3C
CH3
Ha
H1
D2O
1.0000
0.2042
E6-2
Ha
H1
D2O
1.0000
0.4652
4.2
3.7
3.2
2.7
2.2
1.7
1.2
Chemical shift (ppm)

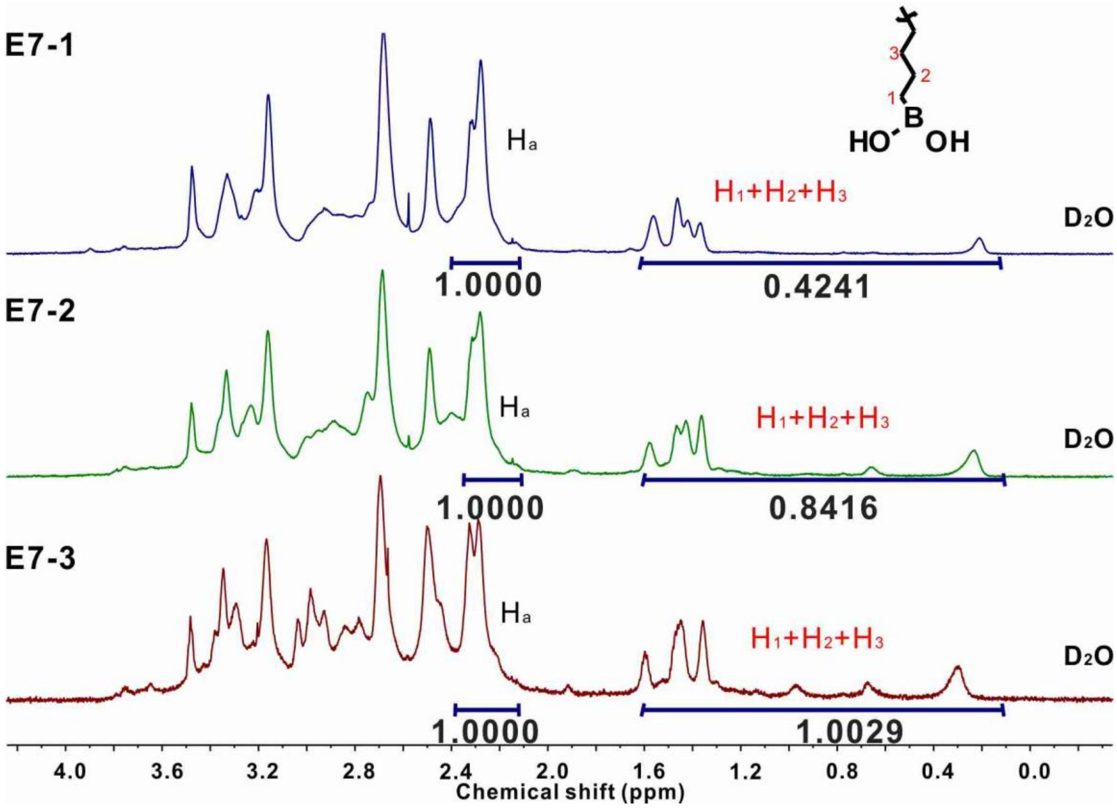
E7-1
E7-2
E7-3
Ha
H1+H2+H3
D2O
1.0000
0.4241
0.8416
1.0029
HO
OH
B
4.0
3.6
3.2
2.8
2.4
2.0
1.6
1.2
0.8
0.4
0.0
Chemical shift (ppm)

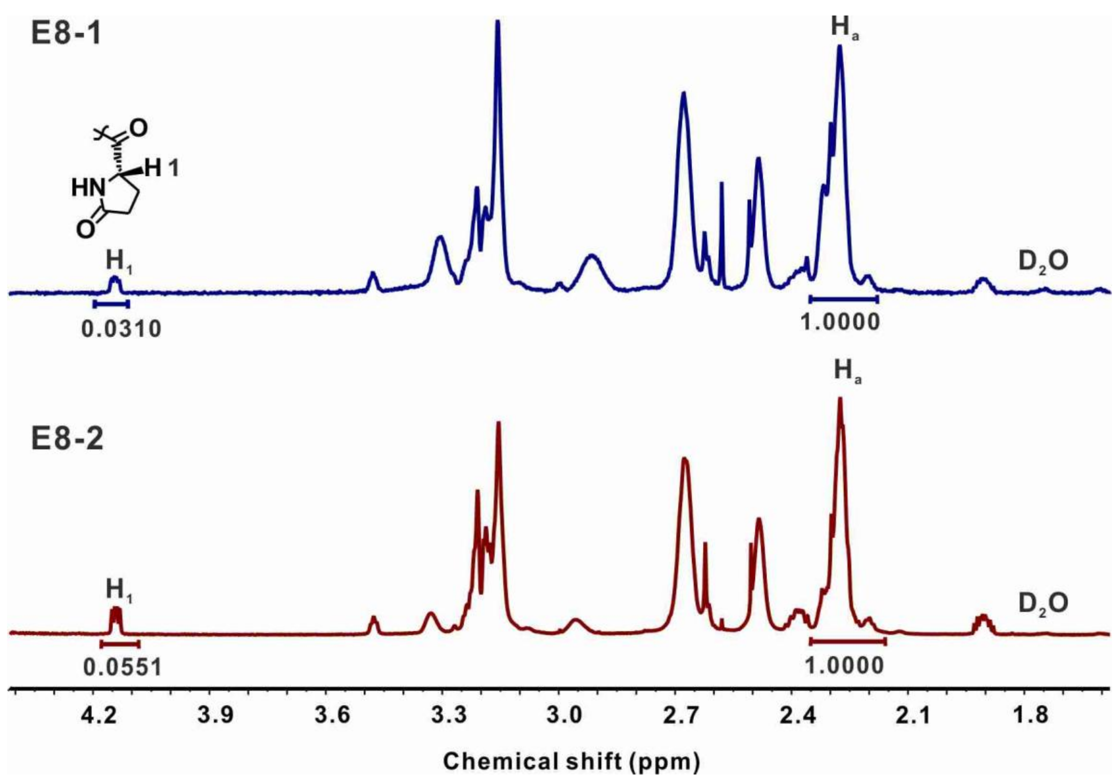
E8-1
E8-2
HN
O
H 1
H1
Ha
D2O
0.0310
0.0551
1.0000
4.2
3.9
3.6
3.3
3.0
2.7
2.4
2.1
1.8
Chemical shift (ppm)

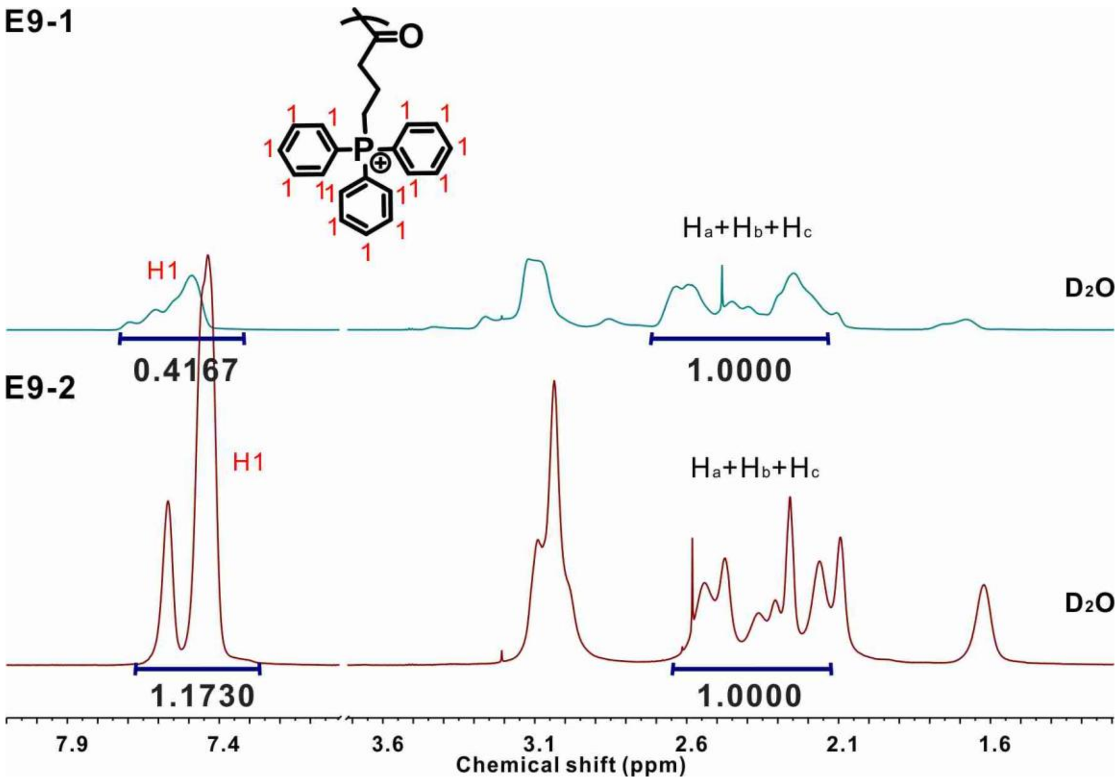
E9-1
H1
Ha+Hb+Hc
D2O
0.4167
1.0000
E9-2
H1
Ha+Hb+Hc
D2O
1.1730
1.0000
7.9 7.4 3.6 3.1 2.6 2.1 1.6
Chemical shift (ppm)

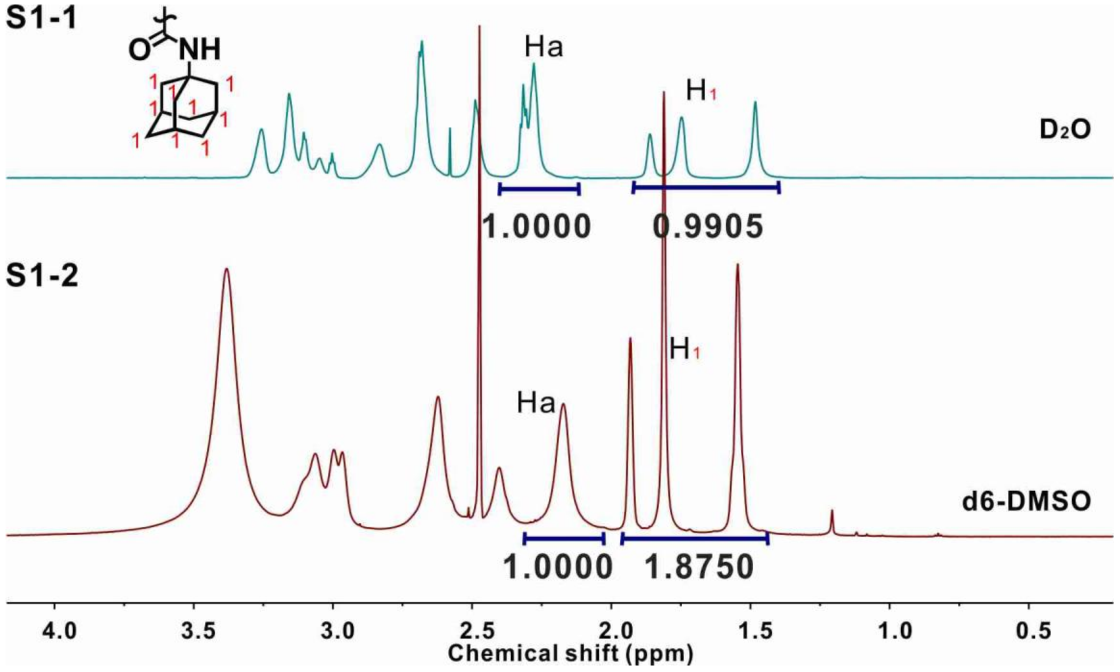
S1-1
NH
O
Ha
H1
D2O
1.0000
0.9905
S1-2
Ha
H1
d6-DMSO
1.0000
1.8750
4.0 3.5 3.0 2.5 2.0 1.5 1.0 0.5
Chemical shift (ppm)

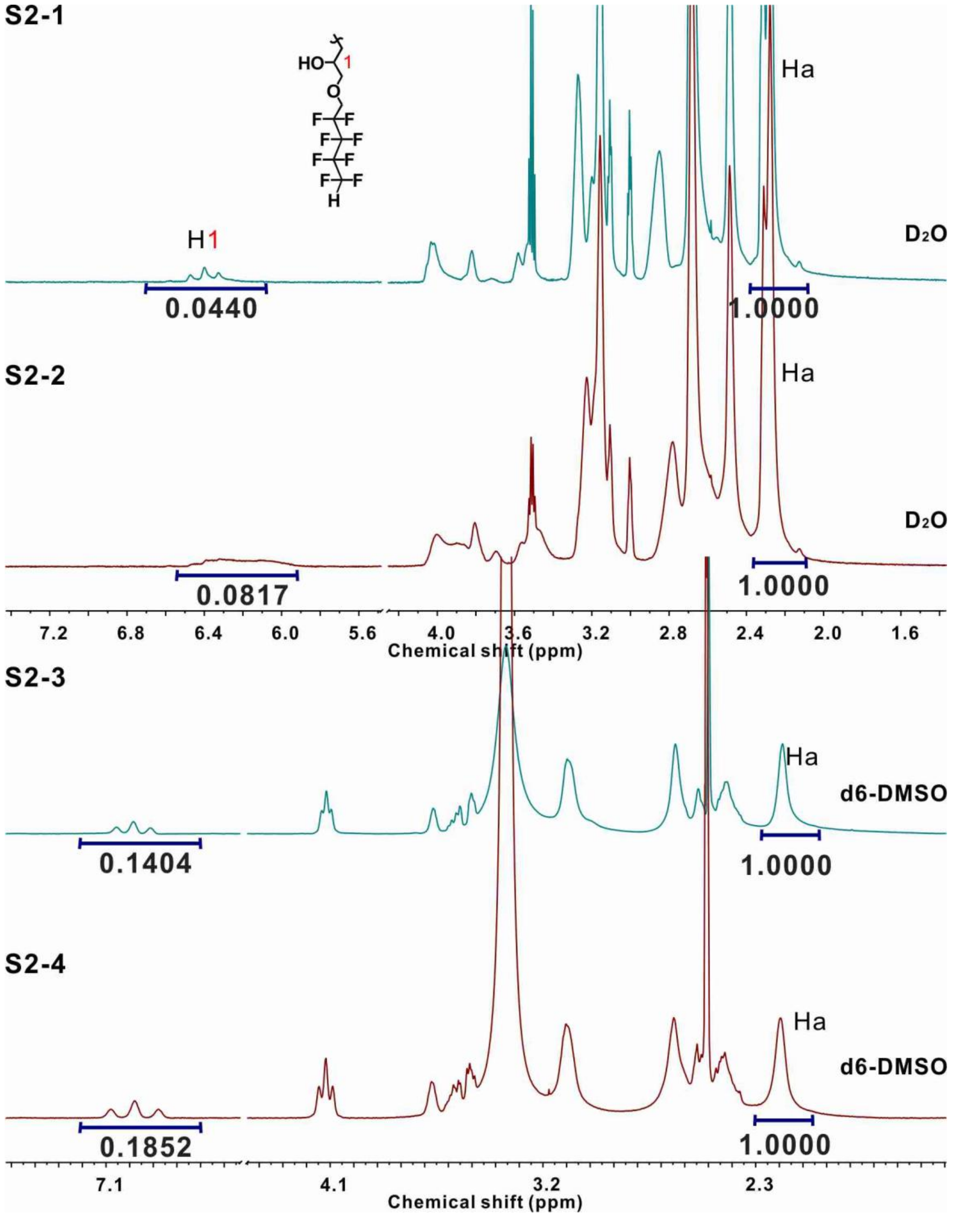
S2-1
HO
1
O
F F
F F
F F
F F
H
H1
Ha
D2O
0.0440
1.0000
S2-2
Ha
D2O
0.0817
1.0000
7.2 6.8 6.4 6.0 5.6 4.0 3.6 3.2 2.8 2.4 2.0 1.6
Chemical shift (ppm)
S2-3
Ha
d6-DMSO
0.1404
1.0000
S2-4
Ha
d6-DMSO
0.1852
1.0000
7.1 4.1 3.2 2.3
Chemical shift (ppm)

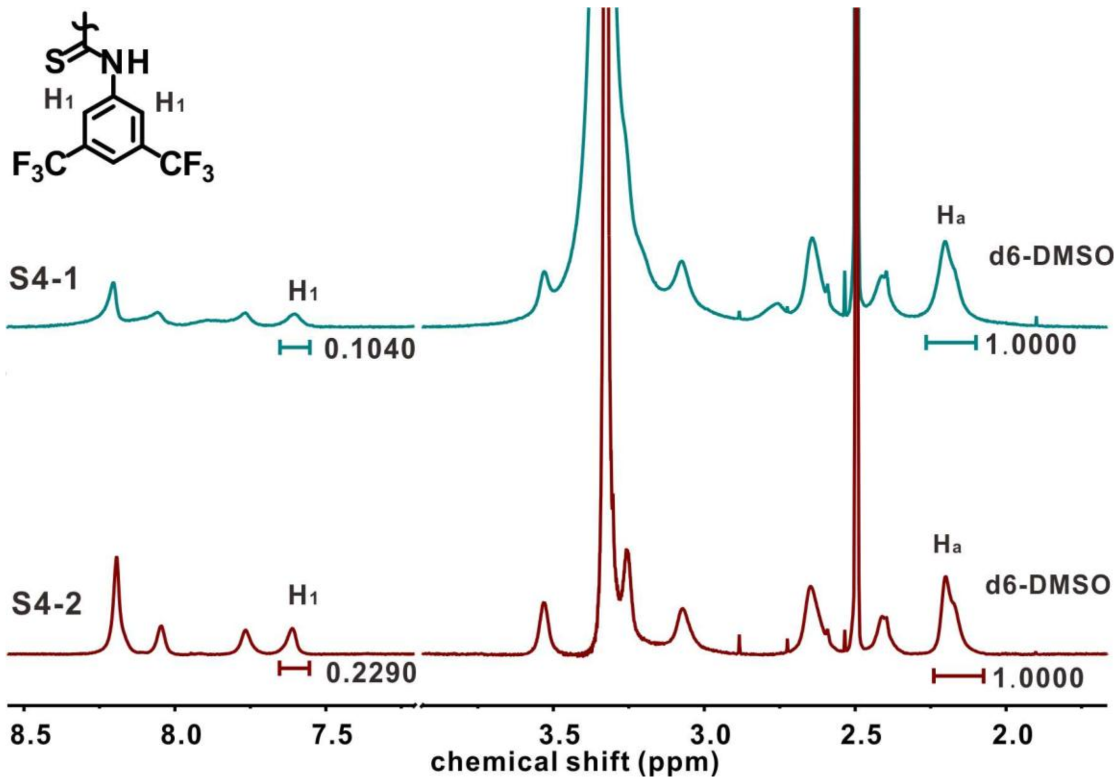
S
NH
H1
H1
F3C
CF3
S4-1
H1
Ha
d6-DMSO
0.1040
1.0000
S4-2
H1
Ha
d6-DMSO
0.2290
1.0000
8.5
8.0
7.5
3.5
3.0
2.5
2.0
chemical shift (ppm)

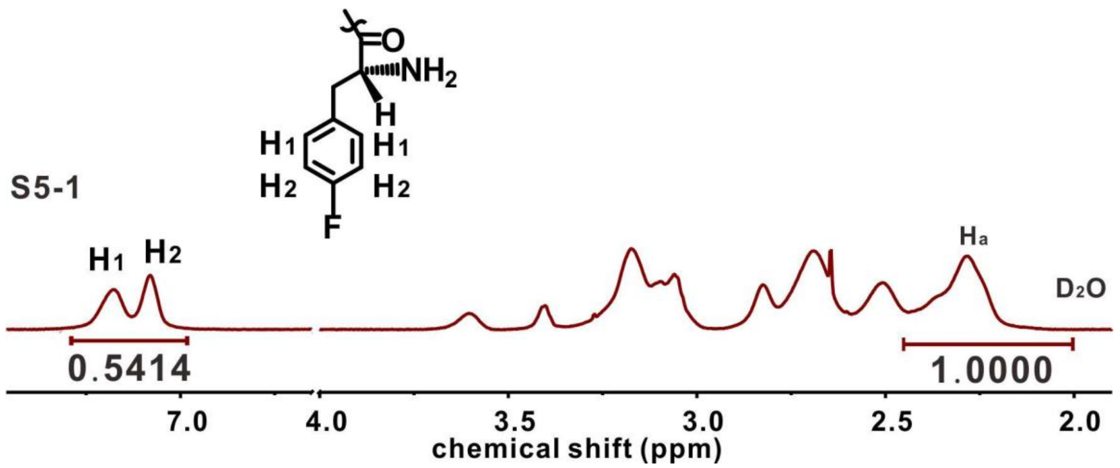
O
NH2
H
H1
H1
H2
H2
F
S5-1
H1
H2
Ha
D2O
0.5414
1.0000
7.0
4.0
3.5
3.0
2.5
2.0
chemical shift (ppm)

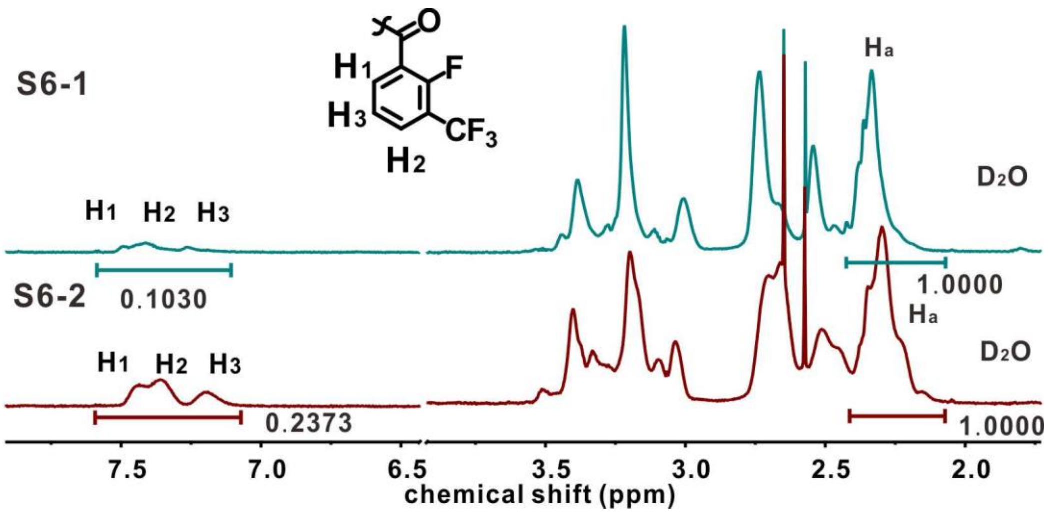
O
H1
F
H3
CF3
H2
S6-1
H1
H2
H3
Ha
D2O
0.1030
1.0000
S6-2
H1
H2
H3
Ha
D2O
0.2373
1.0000
7.5
7.0
6.5
3.5
3.0
2.5
2.0
chemical shift (ppm)

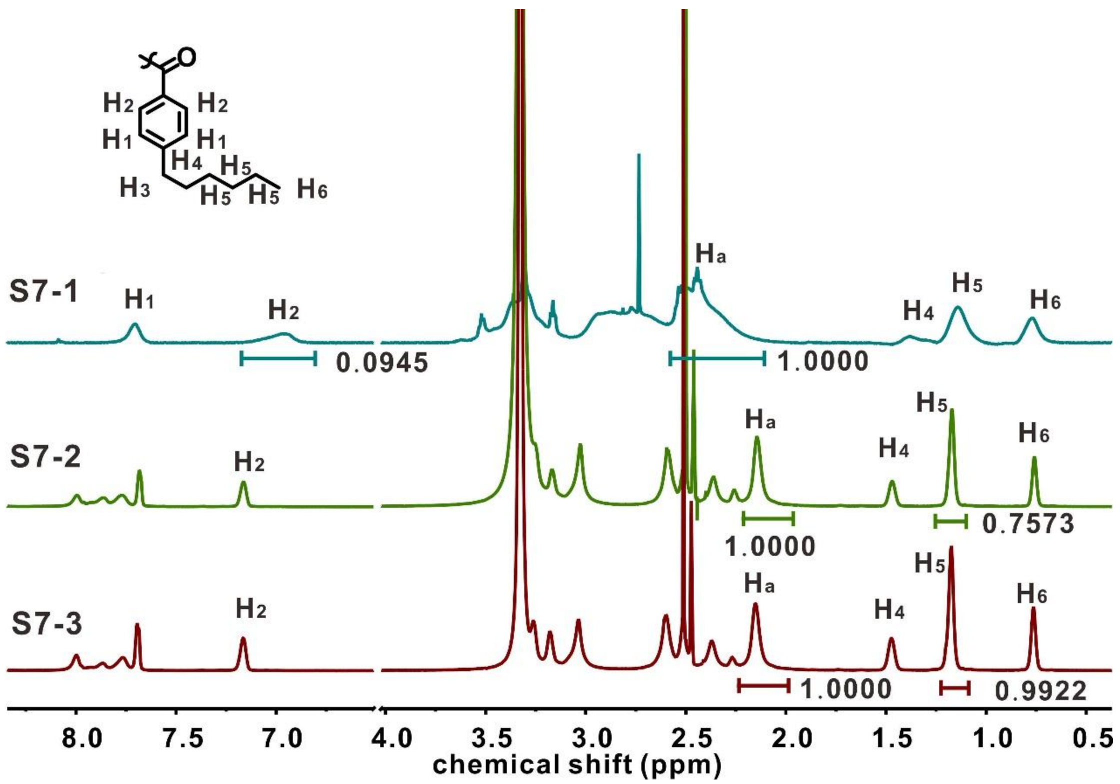

H2
H2
H1
H1
H4
H5
H3
H5
H5
H6
S7-1
H1
H2
Ha
H4
H5
H6
0.0945
1.0000
S7-2
H2
Ha
H4
H5
H6
1.0000
0.7573
S7-3
H2
Ha
H4
H5
H6
1.0000
0.9922
8.0 7.5 7.0 4.0 3.5 3.0 2.5 2.0 1.5 1.0 0.5
chemical shift (ppm)

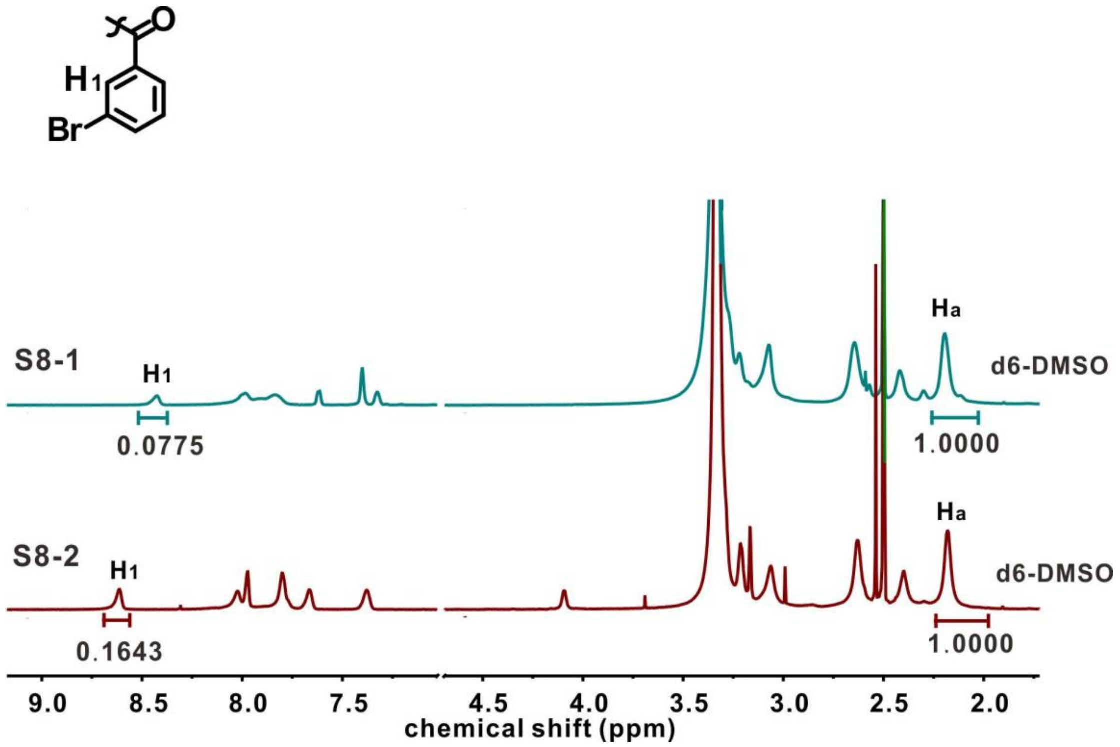

H1
Br
S8-1
H1
Ha
d6-DMSO
0.0775
1.0000
S8-2
H1
Ha
d6-DMSO
0.1643
1.0000
9.0 8.5 8.0 7.5 4.5 4.0 3.5 3.0 2.5 2.0
chemical shift (ppm)

Appendix B. Screening of efficient siRNA vectors in the library of surface-engineered dendrimers.

Gene silencing efficacy of the materials was tested with 50 nM siLuc in HeLa-Luc cells at different weight ratios (w/w=1-20) for 24 h. *Represents the optimal material dose for each material in the gene silencing experiments. “T” indicates that the material is toxic on the transfected cells. The optimal material dose is chosen at which the highest gene silencing efficacy is achieved and the polyplex is not toxic on the transfected cells, in addition, the relative protein mass in each well should be above 80% compared to the non-treated cells.

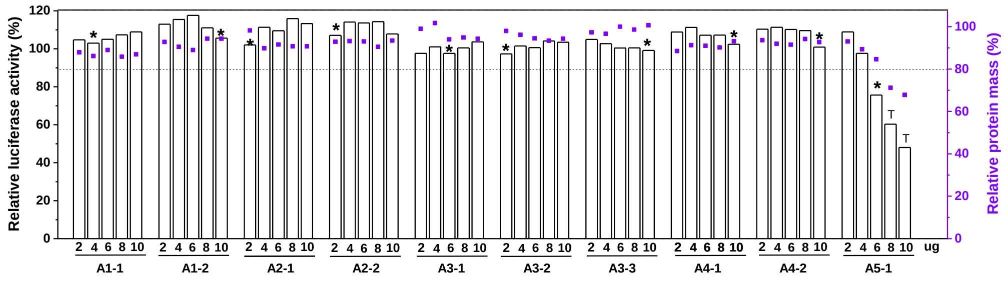

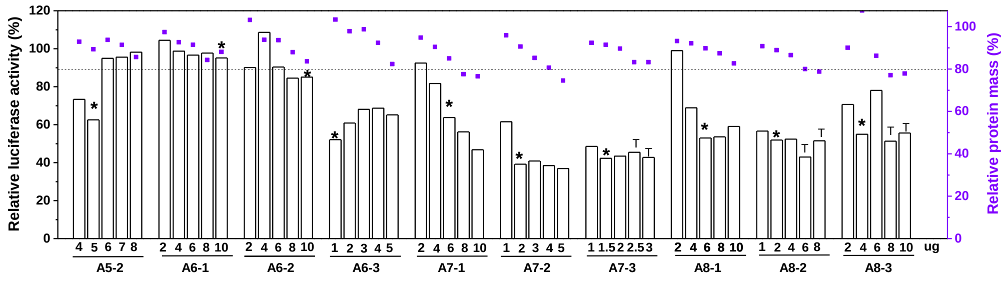

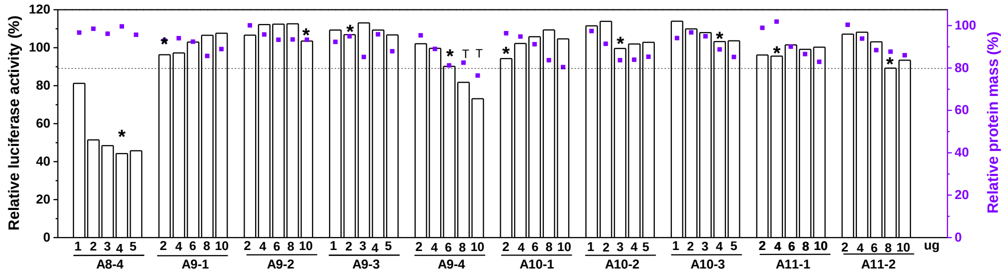

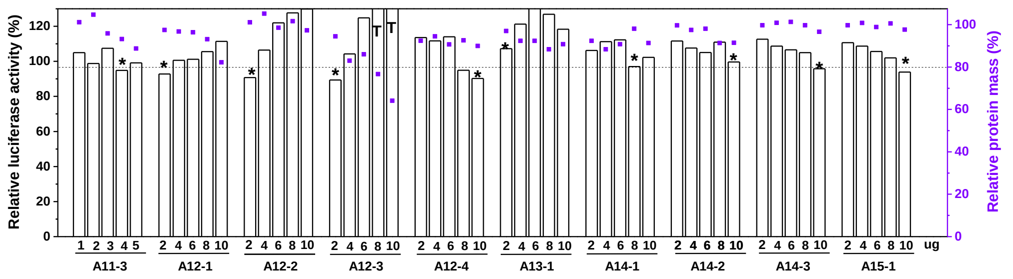
Relative luciferase activity (%)
Relative protein mass (%)
ug
A11-3
A12-1
A12-2
A12-3
A12-4
A13-1
A14-1
A14-2
A14-3
A15-1

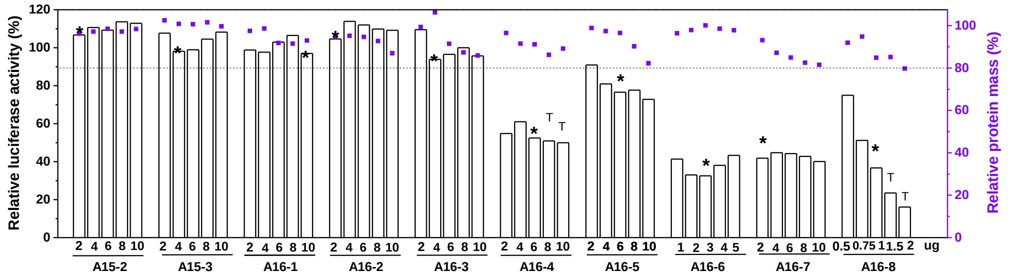
Relative luciferase activity (%)
Relative protein mass (%)
ug
A15-2
A15-3
A16-1
A16-2
A16-3
A16-4
A16-5
A16-6
A16-7
A16-8

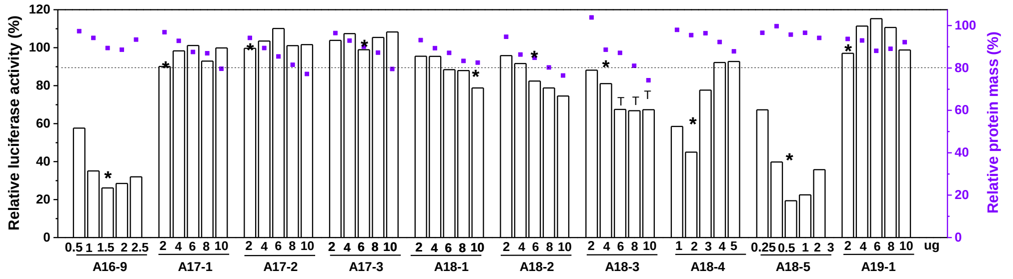
Relative luciferase activity (%)
Relative protein mass (%)
ug
A16-9
A17-1
A17-2
A17-3
A18-1
A18-2
A18-3
A18-4
A18-5
A19-1

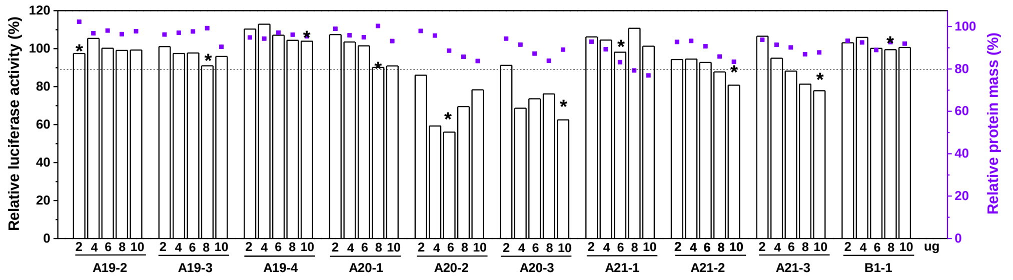
Relative luciferase activity (%)
Relative protein mass (%)
ug
A19-2
A19-3
A19-4
A20-1
A20-2
A20-3
A21-1
A21-2
A21-3
B1-1

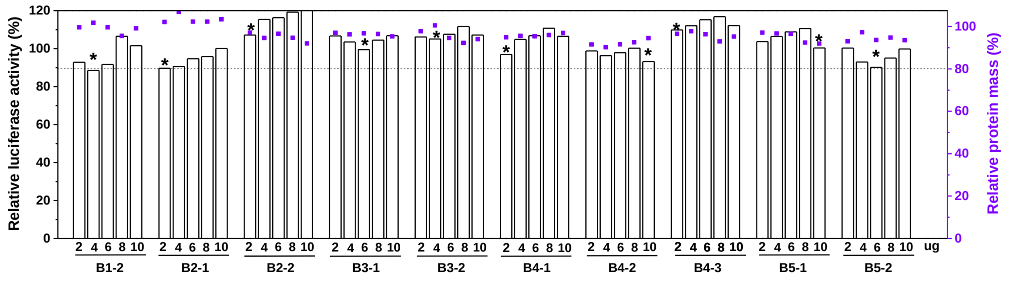
Relative luciferase activity (%)
Relative protein mass (%)
ug
B1-2
B2-1
B2-2
B3-1
B3-2
B4-1
B4-2
B4-3
B5-1
B5-2

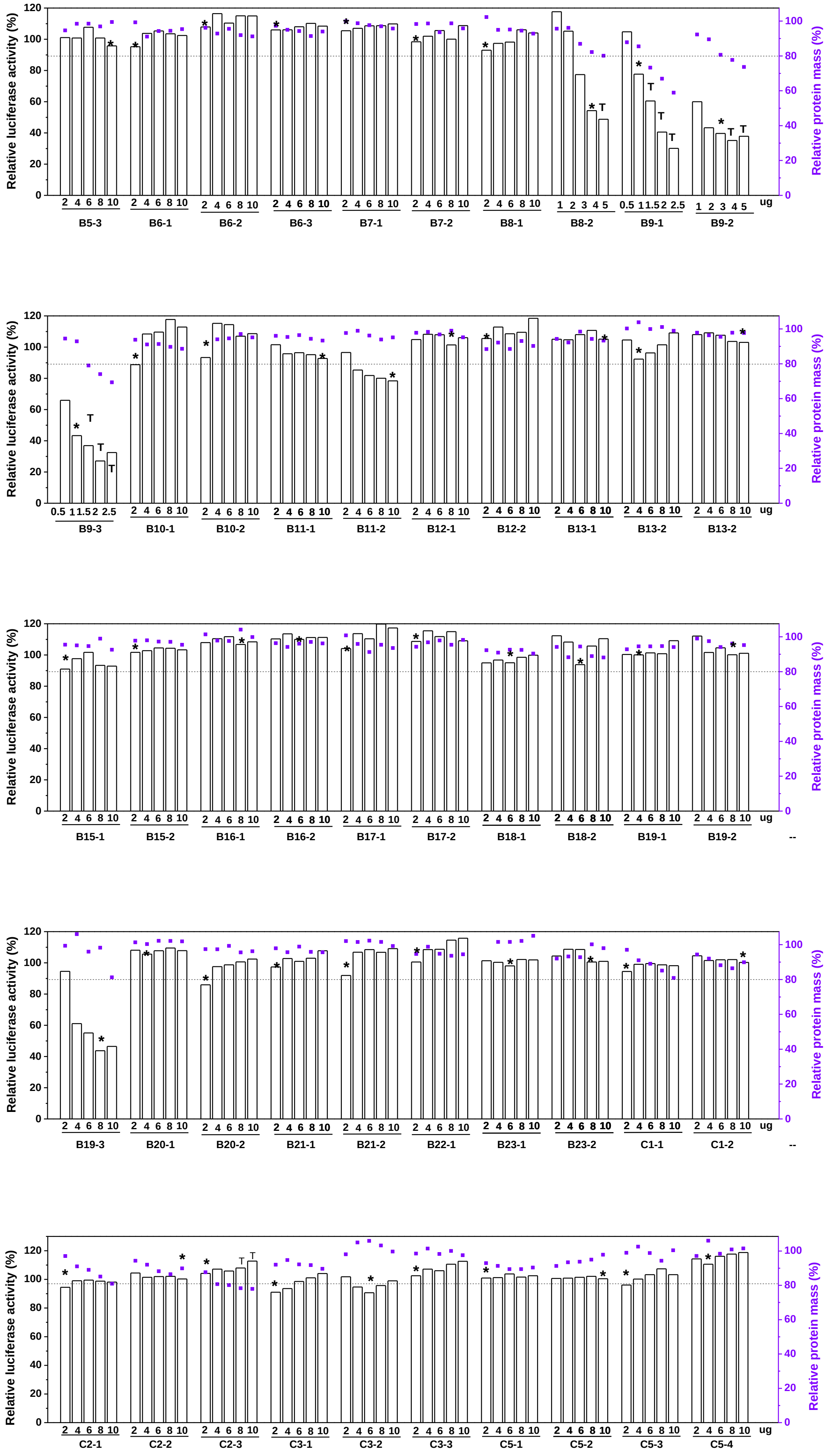

Relative luciferase activity (%)
Relative protein mass (%)
ug
B5-3 B6-1 B6-2 B6-3 B7-1 B7-2 B8-1 B8-2 B9-1 B9-2
B9-3 B10-1 B10-2 B11-1 B11-2 B12-1 B12-2 B13-1 B13-2 B13-2
B15-1 B15-2 B16-1 B16-2 B17-1 B17-2 B18-1 B18-2 B19-1 B19-2
B19-3 B20-1 B20-2 B21-1 B21-2 B22-1 B23-1 B23-2 C1-1 C1-2
C2-1 C2-2 C2-3 C3-1 C3-2 C3-3 C5-1 C5-2 C5-3 C5-4

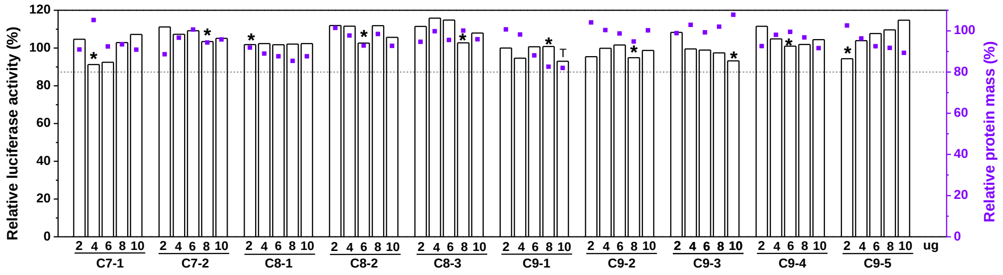
Relative luciferase activity (%)
Relative protein mass (%)
ug
C7-1 C7-2 C8-1 C8-2 C8-3 C9-1 C9-2 C9-3 C9-4 C9-5

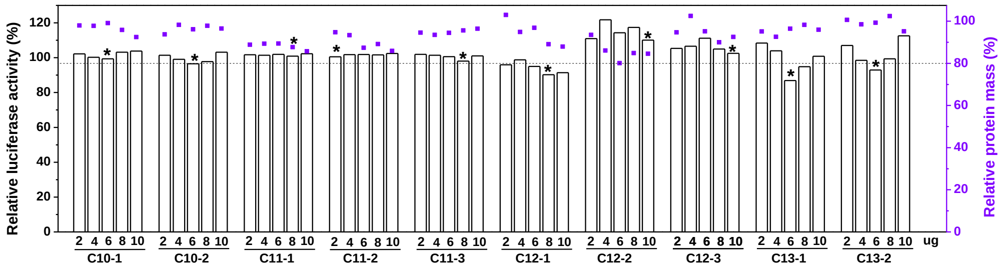
Relative luciferase activity (%)
Relative protein mass (%)
ug
C10-1 C10-2 C11-1 C11-2 C11-3 C12-1 C12-2 C12-3 C13-1 C13-2

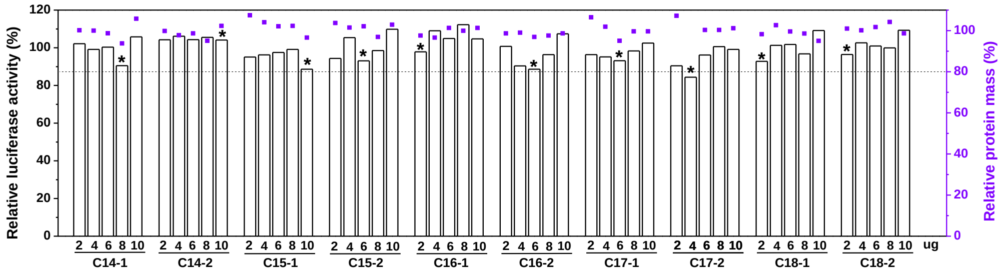
Relative luciferase activity (%)
Relative protein mass (%)
ug
C14-1 C14-2 C15-1 C15-2 C16-1 C16-2 C17-1 C17-2 C18-1 C18-2

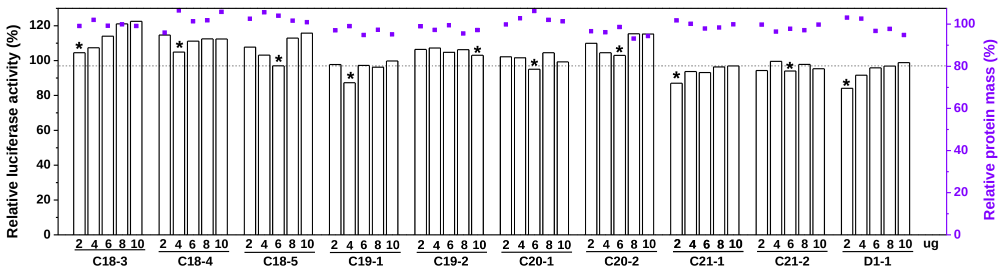
Relative luciferase activity (%)
Relative protein mass (%)
ug
C18-3 C18-4 C18-5 C19-1 C19-2 C20-1 C20-2 C21-1 C21-2 D1-1

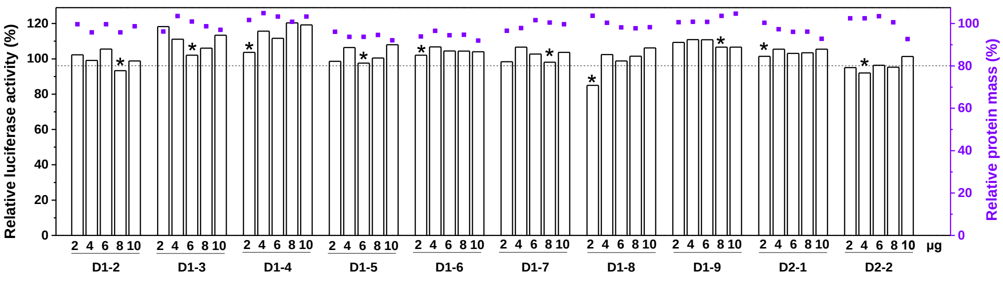
Relative luciferase activity (%)
Relative protein mass (%)
μg
D1-2 D1-3 D1-4 D1-5 D1-6 D1-7 D1-8 D1-9 D2-1 D2-2

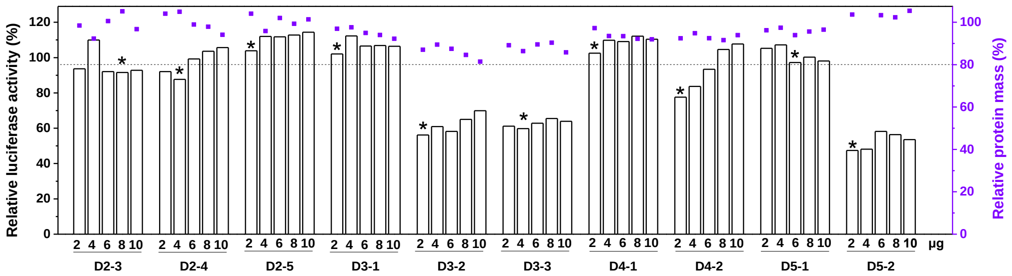
Relative luciferase activity (%)
Relative protein mass (%)
0 20 40 60 80 100 120
0 20 40 60 80 100
2 4 6 8 10 μg
D2-3 D2-4 D2-5 D3-1 D3-2 D3-3 D4-1 D4-2 D5-1 D5-2

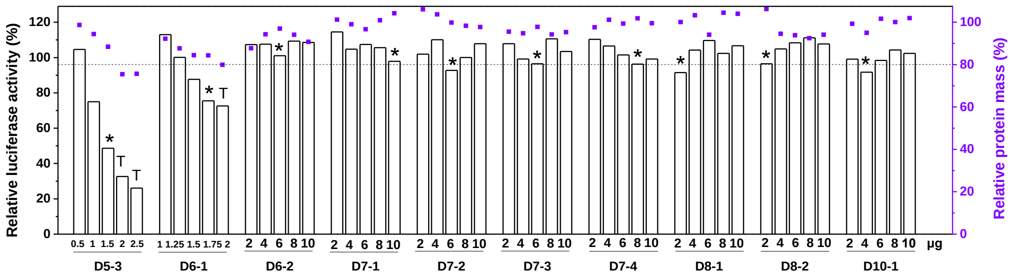
Relative luciferase activity (%)
Relative protein mass (%)
0 20 40 60 80 100 120
0 20 40 60 80 100
0.5 1 1.5 2 2.5
1 1.25 1.5 1.75 2
2 4 6 8 10 μg
T
D5-3 D6-1 D6-2 D7-1 D7-2 D7-3 D7-4 D8-1 D8-2 D10-1

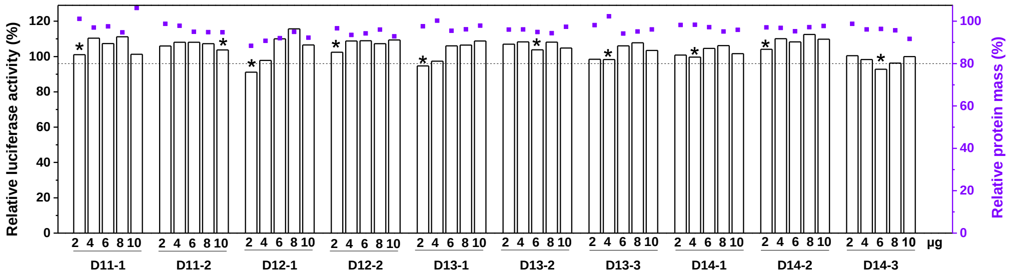
Relative luciferase activity (%)
Relative protein mass (%)
0 20 40 60 80 100 120
0 20 40 60 80 100
2 4 6 8 10 μg
D11-1 D11-2 D12-1 D12-2 D13-1 D13-2 D13-3 D14-1 D14-2 D14-3

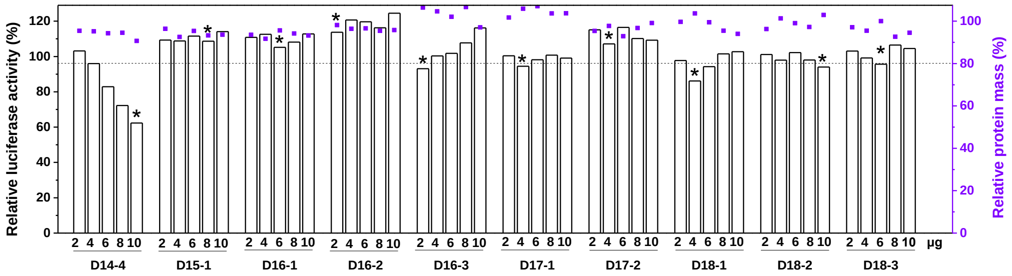
Relative luciferase activity (%)
Relative protein mass (%)
0 20 40 60 80 100 120
0 20 40 60 80 100
2 4 6 8 10 μg
D14-4 D15-1 D16-1 D16-2 D16-3 D17-1 D17-2 D18-1 D18-2 D18-3

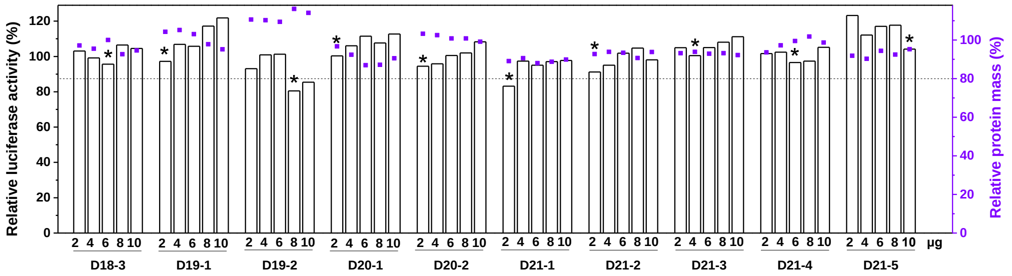
Relative luciferase activity (%)
Relative protein mass (%)
0 20 40 60 80 100 120
0 20 40 60 80 100
2 4 6 8 10 μg
D18-3 D19-1 D19-2 D20-1 D20-2 D21-1 D21-2 D21-3 D21-4 D21-5

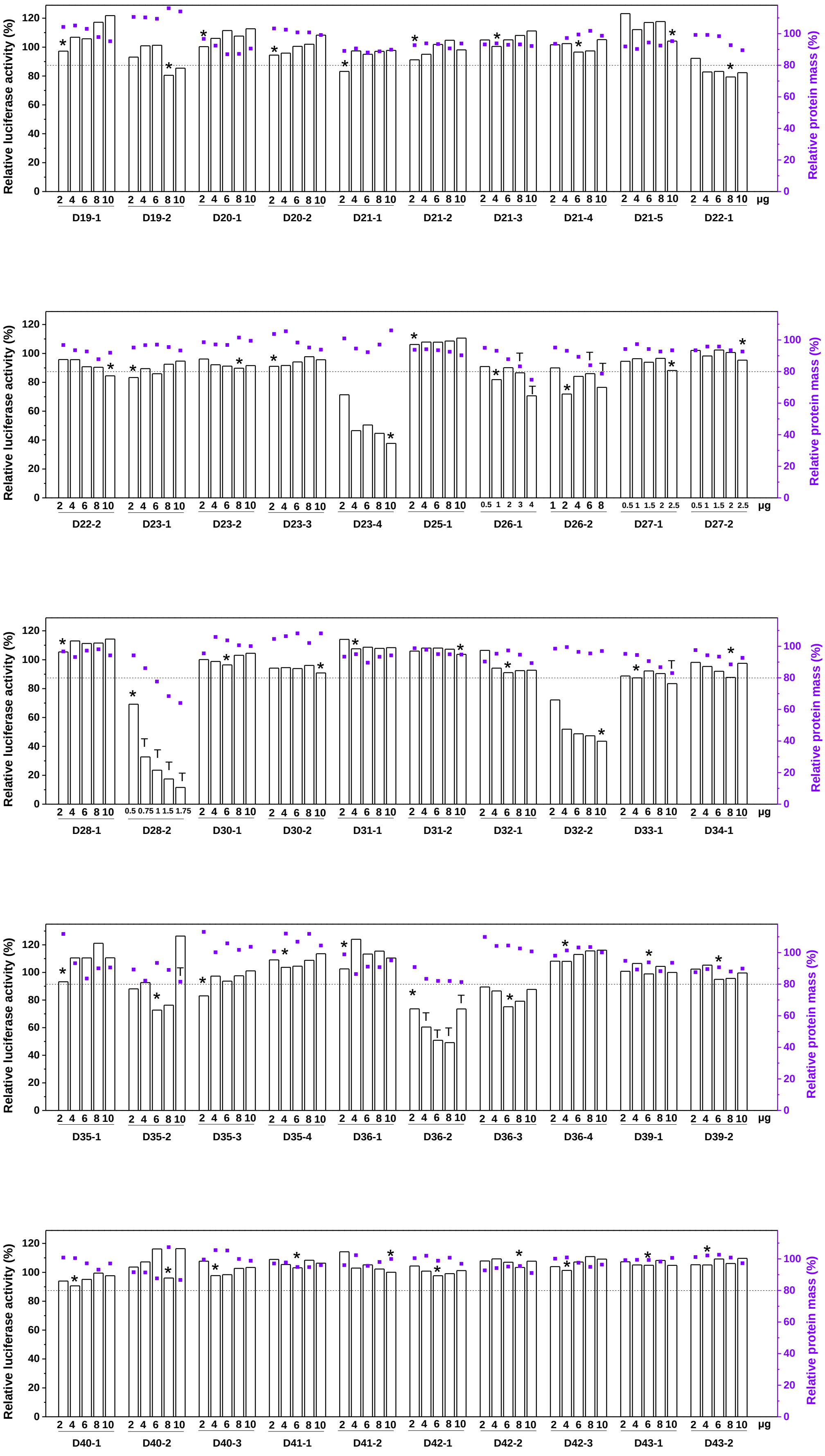
Relative luciferase activity (%)
Relative protein mass (%)
µg
D19-1 D19-2 D20-1 D20-2 D21-1 D21-2 D21-3 D21-4 D21-5 D22-1
D22-2 D23-1 D23-2 D23-3 D23-4 D25-1 D26-1 D26-2 D27-1 D27-2
D28-1 D28-2 D30-1 D30-2 D31-1 D31-2 D32-1 D32-2 D33-1 D34-1
D35-1 D35-2 D35-3 D35-4 D36-1 D36-2 D36-3 D36-4 D39-1 D39-2
D40-1 D40-2 D40-3 D41-1 D41-2 D42-1 D42-2 D42-3 D43-1 D43-2

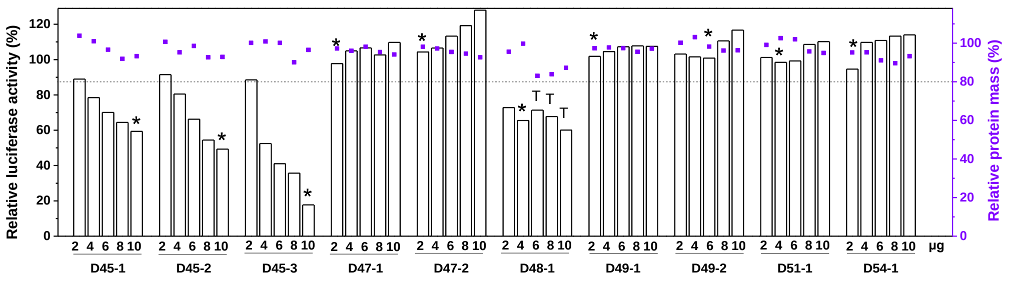
Relative luciferase activity (%)
Relative protein mass (%)
μg
D45-1
D45-2
D45-3
D47-1
D47-2
D48-1
D49-1
D49-2
D51-1
D54-1

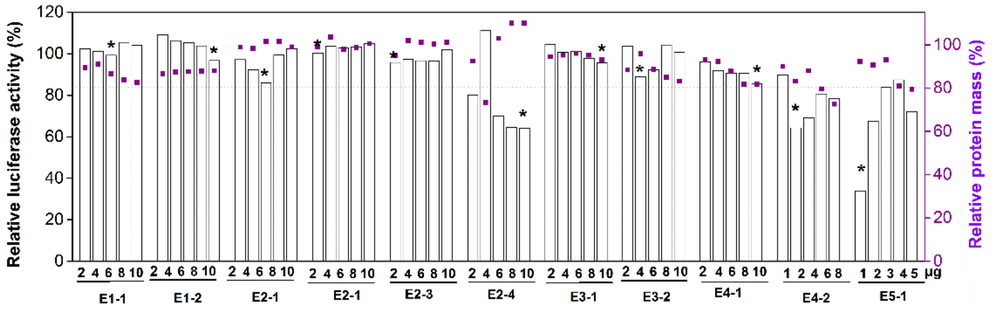
Relative luciferase activity (%)
Relative protein mass (%)
μg
E1-1
E1-2
E2-1
E2-1
E2-3
E2-4
E3-1
E3-2
E4-1
E4-2
E5-1

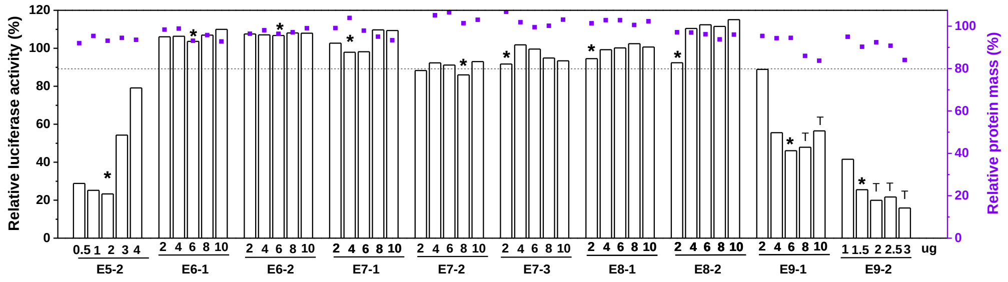
Relative luciferase activity (%)
Relative protein mass (%)
ug
E5-2
E6-1
E6-2
E7-1
E7-2
E7-3
E8-1
E8-2
E9-1
E9-2

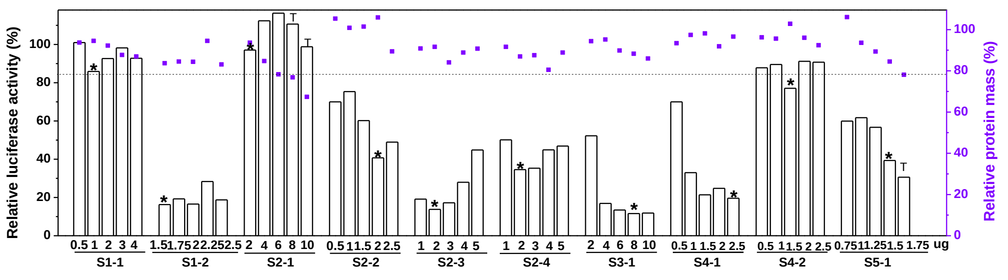
Relative luciferase activity (%)
Relative protein mass (%)
ug
S1-1
S1-2
S2-1
S2-2
S2-3
S2-4
S3-1
S4-1
S4-2
S5-1

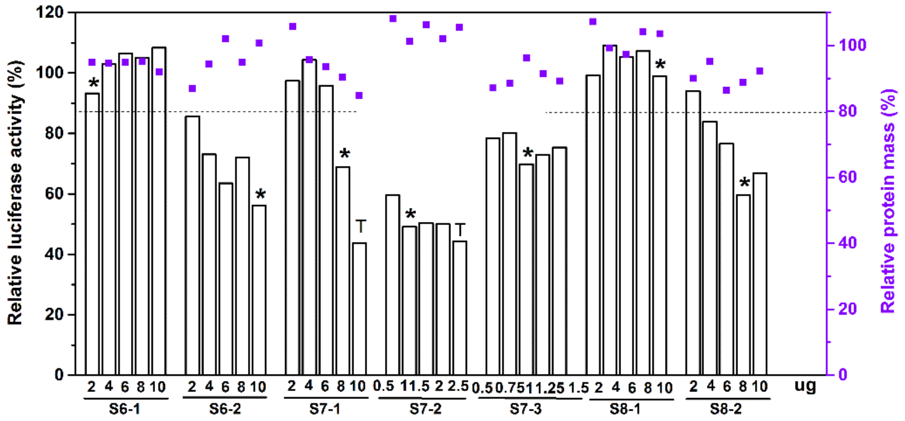

Relative luciferase activity (%)
Relative protein mass (%)
2 4 6 8 10
S6-1
S6-2
S7-1
0.5 1 1.5 2 2.5
S7-2
0.5 0.75 1 1.25 1.5
S7-3
S8-1
S8-2
ug
